# Soybean (*Glycine max*) SWEET gene family: insights through comparative genomics, transcriptome profiling and whole genome re-sequence analysis

**DOI:** 10.1186/s12864-015-1730-y

**Published:** 2015-07-11

**Authors:** Gunvant Patil, Babu Valliyodan, Rupesh Deshmukh, Silvas Prince, Bjorn Nicander, Mingzhe Zhao, Humira Sonah, Li Song, Li Lin, Juhi Chaudhary, Yang Liu, Trupti Joshi, Dong Xu, Henry T. Nguyen

**Affiliations:** National Center for Soybean Biotechnology and Division of Plant Sciences, University of Missouri, Columbia, MO 65211 USA; Department of Plant Biology and Forest Genetics and Linnean Center for Plant Biology, Swedish University of Agricultural Sciences, Uppsala, Sweden; Department of Computer Science, Informatics Institute, and Christopher S. Bond Life Sciences Center, University of Missouri, Columbia, MO 65211 USA; Current address: Agronomy College of Shenyang Agricultural University, Shenyang, China

**Keywords:** SWEET, Effluxer, Sugar transport, Sink, Whole genome re-sequencing, Soybean

## Abstract

**Background:**

SWEET (*MtN3_saliva*) domain proteins, a recently identified group of efflux transporters, play an indispensable role in sugar efflux, phloem loading, plant-pathogen interaction and reproductive tissue development. The SWEET gene family is predominantly studied in *Arabidopsis* and members of the family are being investigated in rice. To date, no transcriptome or genomics analysis of soybean SWEET genes has been reported.

**Results:**

In the present investigation, we explored the evolutionary aspect of the SWEET gene family in diverse plant species including primitive single cell algae to angiosperms with a major emphasis on *Glycine max*. Evolutionary features showed expansion and duplication of the SWEET gene family in land plants. Homology searches with BLAST tools and Hidden Markov Model-directed sequence alignments identified 52 SWEET genes that were mapped to 15 chromosomes in the soybean genome as tandem duplication events. Soybean SWEET (*GmSWEET*) genes showed a wide range of expression profiles in different tissues and developmental stages. Analysis of public transcriptome data and expression profiling using quantitative real time PCR (qRT-PCR) showed that a majority of the *GmSWEET* genes were confined to reproductive tissue development. Several natural genetic variants (non-synonymous SNPs, premature stop codons and haplotype) were identified in the *GmSWEET* genes using whole genome re-sequencing data analysis of 106 soybean genotypes. A significant association was observed between SNP-haplogroup and seed sucrose content in three gene clusters on chromosome 6.

**Conclusion:**

Present investigation utilized comparative genomics, transcriptome profiling and whole genome re-sequencing approaches and provided a systematic description of soybean SWEET genes and identified putative candidates with probable roles in the reproductive tissue development. Gene expression profiling at different developmental stages and genomic variation data will aid as an important resource for the soybean research community and can be extremely valuable for understanding sink unloading and enhancing carbohydrate delivery to developing seeds for improving yield.

**Electronic supplementary material:**

The online version of this article (doi:10.1186/s12864-015-1730-y) contains supplementary material, which is available to authorized users.

## Background

Photosynthesis fixes carbon in the leaves to make sugars as the primary transportable form of energy. Sugar production, status, and transport to the various tissues modulate the growth, productivity, and yield of plants [1]. In addition to their essential roles as substrates in carbon and energy metabolism, sugars also play an important role in signal transduction [1, 2]. In plants, sugars are accumulated in the form of simple sugars, carbohydrates, and starch. Stored sugars are then transported from leaves (source tissue) to the other plant parts (sink tissue) such as roots, modified leaves, and reproductive tissues (seeds). This transport from source to sink is modulated via phloem sap. Sucrose is synthesized in the cytosol and translocated to other non-photosynthetic tissues for direct metabolic use or for conversion to starch. Allocation of sucrose is facilitated by both short-distance transport systems and long-distance transport systems [3]. Short distance transport is achieved at the intra-cellular and inter-cellular levels, where sucrose is transported via diffusion/protoplasmic streaming and plasmodesmata, respectively [4, 5]. It then moves from cell to cell via plasmodesmata until it reaches the phloem parenchyma cells, and in the phloem parenchyma cells, processes related to long-distance transport initiate [6, 7]. Among the sugars, only a few are allocated to the phloem long-distance transport system and sucrose is the main form of carbon found in the phloem tissue followed by polyols, raffinose, etc. [8, 9]. Of the many different sugars found in plants, it is mainly sucrose that is transported in the phloem, where it is the most abundant carbonaceous compound [8].

The amount of sucrose available for transport to the sink tissues is very crucial for plant development [8, 10]. Metabolite transport efficiency influences photosynthetic productivity by relieving product inhibition and contributes to plant vigor by controlling source/sink relationships and biomass partitioning. The sucrose transport is controlled or facilitated by SUT (sucrose transporter) [11–13] and SWEET (sucrose effluxer) proteins [14–16]. SUT has been widely studied in many plant species [4, 11–14, 17, 18]. SUT proteins are expressed at low levels and display saturable sucrose transport kinetics, suggesting that additional transport proteins are responsible for sucrose allocation across the membrane [6]. The milestone efforts that identified the sucrose effluxer was led by Chen et. al. (2010) [15]. They identified the role of the SWEET (**S**ugars **W**ill **E**ventually be **E**xported **T**ransporters) gene family as sucrose effluxers based on their role in transporting glucose molecules across a membrane. SWEET proteins contains a *MtN3_slv* transmembrane domain that is essential for the maintenance of animal blood glucose levels, plant nectar production, and plant seed and pollen development [19, 20]. The first member of the SWEET family, *MtN3*, was identified as a nodulin-specific EST in the leguminous plant *Medicago truncatula* [21], and *MtN3_slv* was identified as an embryonic salivary gland specific gene in drosophila [22]. SWEET proteins function as uniporters, facilitate diffusion of sugars across cell membranes, and mediate sucrose efflux from putative phloem parenchyma into the phloem apoplasm [23–25]. In Arabidopsis, members of the SWEET gene family, *AtSWEET11* and −*12* were localized to the plasma membrane of the phloem parenchyma and are the main facilitators of sucrose flux. Mutations in *AtSWEET11, −12* genes led to defective phloem loading without affecting the phenotype [26]. Using optical sucrose sensors, SWEET proteins were identified as assisting movement of sucrose across cell membranes in preparation for long-distance transport. SWEET proteins are expressed in phloem parenchyma cells and are key to the export of sucrose from leaves [26].

SWEET transporters have diverse physiological roles and are essential for the maintenance of animal blood glucose levels, plant nectar production, and plant seed and pollen development [15, 23]. Arabidopsis *AtSWEET8* is essential for pollen viability, and the rice homologous *OsSWEET11* and *OsSWEET14* are specifically exploited by bacterial pathogens for virulence by means of direct binding of a bacterial effector to the SWEET promoter [27, 28]. Bacterial and fungal symbionts/pathogens induce the expression of different SWEET genes by secreting the effector protein that binds and activates SWEET genes, indicating that the sugar efflux function of SWEET transporters is targeted and hijacked by pathogens and symbionts for nutritional gain [5, 6, 15, 28, 29].

The sink organs, especially developing seeds which are mainly heterotrophic, depend on nutrients from their parent plants [30, 31]. Early development of the embryo is controlled by the maternal tissue and then during maturation it is controlled by the filial tissues [32]. Phloem unloading in most of the sink tissues follows symplasmic routes [30, 33]. In many dicot seeds, e.g. legumes [32] and Arabidopsis [31], the filial tissues are symplasmically isolated/interrupted by apoplast from the phloem in the maternal seed tissue. Transport of sucrose from phloem to the filial tissue is associated with the expression of sugar transporters, localized to the plasma membranes of filial cells. [5, 25, 33–36]. Ludewig et. al. [37] and Braun [24] have reviewed and discussed role of the SWEET family transporter as putative facilitator of phloem unloading or as the transporter mediating diffusion of sucrose in sink tissue. Similarly, it has been proposed that enhancing nutrient flow to the developing endosperm and embryo by overexpressing SWEET genes along with cell wall invertase and hexose symporter genes at seed maternal-filial interface can increase the seed yield [5, 38]. In *M. domestica*, the SWEET genes, including other sugar transporter genes, are involved in sugar accumulation in sink tissue and the concentration of sugars were positively correlated with the SWEET gene expression [39].

To date SWEET genes are well studied in Arabidposis [15, 26] and rice [20, 40] but no genome-wide exploration and characterization of the SWEET gene family has been performed in soybean. Studying the sucrose efflux system across different species and genera will lead us to understand the evolutionary aspects of the SWEET gene family. In this study, we first collected the SWEET gene family in a number of plant species, then focused on soybean where 52 putative SWEET genes were identified. The publicly available transcriptome datasets were explored and the expression pattern of 23 genes were analyzed using qRT-PCR in reproductive tissues. The wealth of whole genome re-sequencing resources in soybean provided an opportunity to explore natural variation in the soybean SWEET genes. The data presented here lays the foundation for further investigations into the biological and physiological processes of SWEET genes in soybean.

## Results

### Identification of SWEET genes in soybean and other species

**Fig. 1:**
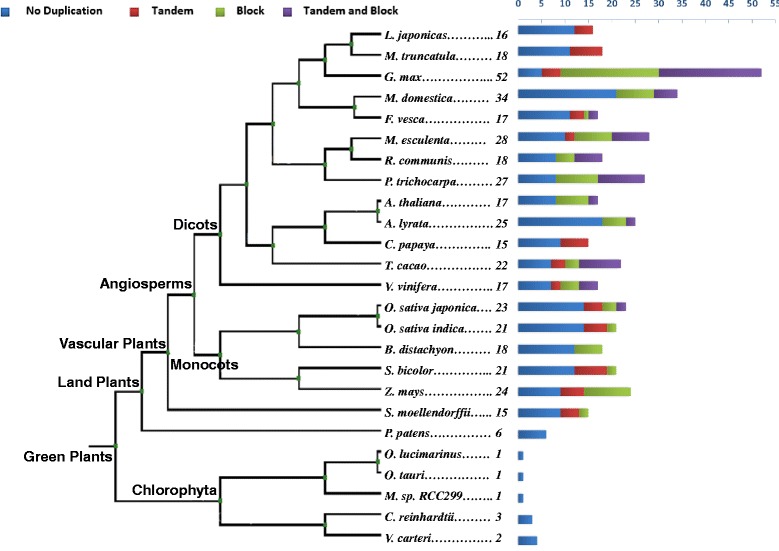
Distribution of SWEET genes and duplication events in 25 plant genomes. The total number of SWEET found in each genome is indicated in the bar. The numbers above horizontal axis suggest number of genes. The gene duplication analysis displays the fraction of block and tandem duplicates for a given set of genes

To find soybean SWEET homologues, BLAST and PFAM [41] searches were performed using *Arabidposis* and rice SWEET genes. This led to the identification of 52 genes with high homology (Fig. 1, Additional file 1). This is far higher than in the other 24 species used in this study. A number of genes with lower homology to SWEET were also found, but were not studied further. The 52 soybean SWEET genes identified in our study were designated as *GmSWEET1* to *GmSWEET*52. Similarly SWEET genes in other species were extracted from the Plaza comparative genomics platform [42] using BLASTN and BLASTP searches, 444 SWEET genes (including 33 outliers) were predicted across 25 genomes (Additional file 1). The details about other parameters, including nucleic acid and protein sequences, are provided in Table 1 and Additional file 1.

**Table 1:** List of 52 soybean SWEET genes and their sequence details (aa- amino acid)

Name	Glyma ID V1.0	Protein aa	Chr. No.	Intron	TMs	mRNA coordinates		Arabidopsis Orthologs
						Start	End	
*GmSWEET1*	*Glyma02g09710*	262	2	5	7	7672242	7674181	AtSWEET9
*GmSWEET2*	*Glyma03g36790*	316	3	8	7	43645675	43647218	AtSWEET9
*GmSWEET3*	*Glyma03g39430*	155	3	4	4	45507339	45508526	AtSWEET16/17
*GmSWEET4*	*Glyma04g37510*	259	4	5	7	43916099	43918975	AtSWEET10
*GmSWEET5*	*Glyma04g37520*	283	4	5	7	43926535	43929580	AtSWEET10
*GmSWEET6*	*Glyma04g37530*	277	4	4	6	43938391	43940184	AtSWEET11/12/13/14
*GmSWEET7*	*Glyma04g41680*	175	4	4	5	47528111	47529572	AtSWEET3
*GmSWEET8*	*Glyma04g42040*	248	4	5	7	47812561	47815829	AtSWEET1
*GmSWEET9*	*Glyma05g02070*	226	5	4	6	1492949	1494364	AtSWEET4
*GmSWEET10*	*Glyma05g25180*	283	5	3	7	31313674	31315182	AtSWEET15
*GmSWEET11*	*Glyma05g38340*	258	5	5	7	41712591	41715252	AtSWEET10
*GmSWEET12*	*Glyma05g38350*	276	5	6	6	41723915	41726765	AtSWEET11/12/13/14
*GmSWEET13*	*Glyma06g12740*	259	6	5	7	9947277	9952565	AtSWEET1
*GmSWEET14*	*Glyma06g13110*	255	6	5	7	10255499	10257429	AtSWEET3
*GmSWEET15*	*Glyma06g17520*	310	6	5	7	13868324	13870606	AtSWEET11/12/13/14
*GmSWEET16*	*Glyma06g17530*	261	6	5	7	13887645	13890535	AtSWEET10
*GmSWEET17*	*Glyma06g17540*	259	6	5	7	13901720	13904519	AtSWEET10
*GmSWEET18*	*Glyma06g21570*	244	6	7	5	18131992	18134116	AtSWEET16/17
*GmSWEET19*	*Glyma06g21640*	192	6	3	4	18206442	18207648	AtSWEET16/17
*GmSWEET20*	*Glyma08g01300*	295	8	5	7	771448	773996	AtSWEET11/12/13/14
*GmSWEET21*	*Glyma08g01310*	255	8	5	7	781403	783956	AtSWEET10
*GmSWEET22*	*Glyma08g02890*	274	8	4	7	1977779	1981412	AtSWEET15
*GmSWEET23*	*Glyma08g08200*	260	8	5	7	5861243	5863837	AtSWEET15
*GmSWEET24*	*Glyma08g19580*	281	8	5	7	14793461	14795629	AtSWEET15
*GmSWEET25*	*Glyma08g47550*	272	8	5	7	46378609	46380649	AtSWEET15
*GmSWEET26*	*Glyma08g47560*	274	8	5	7	46385711	46388217	AtSWEET15
*GmSWEET27*	*Glyma08g48280*	224	8	2	6	46926095	46926877	AtSWEET9
*GmSWEET28*	*Glyma09g04840*	245	9	5	7	3652169	3657426	AtSWEET16/17
*GmSWEET29*	*Glyma12g36300*	236	12	5	7	39418338	39419741	AtSWEET2
*GmSWEET30*	*Glyma13g08190*	256	13	5	7	8569038	8571904	AtSWEET3
*GmSWEET31*	*Glyma13g09140*	249	13	5	7	10118236	10121745	AtSWEET1
*GmSWEET32*	*Glyma13g10560*	258	13	4	7	12437340	12439924	AtSWEET6/7
*GmSWEET33*	*Glyma13g23860*	246	13	5	6	27171336	27175275	AtSWEET4
*GmSWEET34*	*Glyma13g33950*	236	13	5	7	35599596	35602840	AtSWEET2
*GmSWEET35*	*Glyma14g17810*	181	14	7	5	19873917	19875274	AtSWEET9
*GmSWEET36*	*Glyma14g27610*	250	14	5	7	33859148	33862494	AtSWEET1
*GmSWEET37*	*Glyma14g30740*	247	14	6	7	37447737	37449539	AtSWEET3
*GmSWEET38*	*Glyma14g30940*	255	14	5	7	37715242	37718176	AtSWEET3
*GmSWEET39*	*Glyma15g05470*	250	15	5	7	3856854	3858704	AtSWEET15
*GmSWEET40*	*Glyma15g16030*	246	15	5	7	12350866	12354851	AtSWEET16/17
*GmSWEET41*	*Glyma15g27530*	262	15	5	6	29768360	29770247	AtSWEET2
*GmSWEET42*	*Glyma15g27750*	236	15	5	7	30321666	30324324	AtSWEET2
*GmSWEET43*	*Glyma17g09840*	227	17	5	6	7343156	7345793	AtSWEET4
*GmSWEET44*	*Glyma18g53250*	263	18	5	7	61559244	61561078	AtSWEET9
*GmSWEET45*	*Glyma18g53930*	269	18	5	7	62184272	62186255	AtSWEET15
*GmSWEET46*	*Glyma18g53940*	272	18	5	7	62193884	62196748	AtSWEET15
*GmSWEET47*	*Glyma19g01270*	232	19	4	7	880980	884031	AtSWEET4
*GmSWEET48*	*Glyma19g01280*	247	19	5	6	886189	893470	AtSWEET4
*GmSWEET49*	*Glyma19g42040*	308	19	5	7	48123304	48127034	AtSWEET16/17
*GmSWEET50*	*Glyma20g01890*	160	20	3	1	1422950	1425797	AtSWEET16/17
*GmSWEET51*	*Glyma20g16160*	257	20	4	6	22458358	22460991	AtSWEET6/7
*GmSWEET52*	*Glyma20g21060*	213	20	4	4	29990001	29991796	AtSWEET16/17

### Soybean SWEET genes are highly conserved and points to duplication events in higher plants

Comparative genomics of SWEET genes were performed using 25 plant genomes encompassing monocots, dicots and lower plants with subsequent focus on the soybean SWEET family. According to a database of conserved protein families (PFAM), *MtN3-like* clan (http://pfam.xfam.org/clan/MtN3-like) contains five subfamilies: *MtN3_slv* (PF03083), PQ-loop (PF04193), MPC (PF03650), ER Lumen Receptor (PF00810), and Lab-N (PF07578). The SWEET genes belongs to *MtN3_slv* subfamily and serve function in sugar transport whereas other proteins have different roles, for example PQ-loop subfamily involved in amino acid transport [43]. SWEET gene (*MtN3_slv*) homologues from algae, moss and higher plants were collected from Genbank and Plaza 2.5 and 3.0 comparative genomics platforms [42]. Genome-wide distribution of the SWEET gene family showed that the unicellular plants and blue green algae have fewer copies (1–4) of SWEET genes, followed by 6 and 15 genes in *Physcomitrella patens* (non-vascular) and *Selaginella moellendorffii* (vascular) from lower plant group, respectively (Fig. 1).

**Fig. 2:**
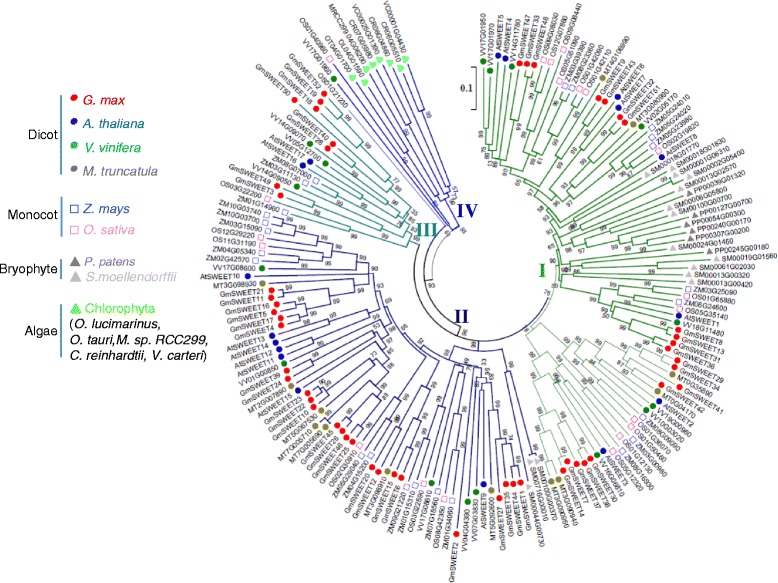
Phylogenetic relationship of SWEET gene family proteins in 13 different species. The phylogenetic tree was built using the neighbor-joining (NJ) method implemented in MEGA5.1. The roman numerals (I – IV) indicated with different colors, represents the clades associated with higher and lower plant groups. The numbers at the nodes represent bootstrap percentage values based on 1000 replications. Genes from each species are marked with different bullet point colors

To better understand the evolutionary relationship between different plant SWEET (*MtN3_slv*) homologues, we constructed a phylogenetic tree using 173 SWEET genes from 13 species representing major plant groups (Fig. 2, Amino acid sequences see Additional file 2). These 13 plant species represent; dicots (*Glycine max, Medicago truncatula, Vitis vinifera, Arabidopsis thaliana*), monocots (*Oryza sativa*, *Zea mays*), bryophytes (*Physcomitrella patens*, *Selaginella moellendorffii*), and algae (*Ostreococcus lucimarinus Ostreococcus tauri, Micromonas sp. RCC299, Chlamydomonas reinhardtii, Volvox carteri*). The phylogenetic clustering between different plant species reveal the evolutionary relationship among plant SWEET proteins. Four major clades were perceived, in which both monocots and dicots were distributed between clades I-III. The algal species were observed in clade number IV and the bryophytes (*P.patens* and S. *moellendorffii*) were predominantly observed in clade I. Interestingly, four algal species, those of the unicellular chlorophyta group (*O. lucimarinus*, *O. tauri, M. sp.RCC299, C. reinhardtii*) contain only 7- transmembrane domains (TMs) and not 3-TMs, which led us to speculate that the multicellular plants (bryophytes and flowering plants) might have acquired 3-TMs from symbiotic bacteria through horizontal gene transfer or might have evolved through internal duplication of 3-TMs within the gene.

The phylogeny of the soybean SWEET genes was compared to the Arabidopsis and rice SWEET genes since they have been functionally characterized and their duplication events represented by a whole-genome duplication. The lineage-specific arrangement of SWEET genes proposes that the genes may be expanded and then diversified after the monocot and dicot division. Soybean contains the highest number (52) of SWEET homologues as compared to other plant species included in the present study. To gain further insight into the structural diversity of *GmSWEET* genes, we compared intron/exon organization in the coding sequences of paralog pairs and found that most of the paralogs shared similar gene organization, consistent with the phylogenetic analysis (Additional file 3).

### Soybean SWEET gene family expansion

**Fig. 3:**
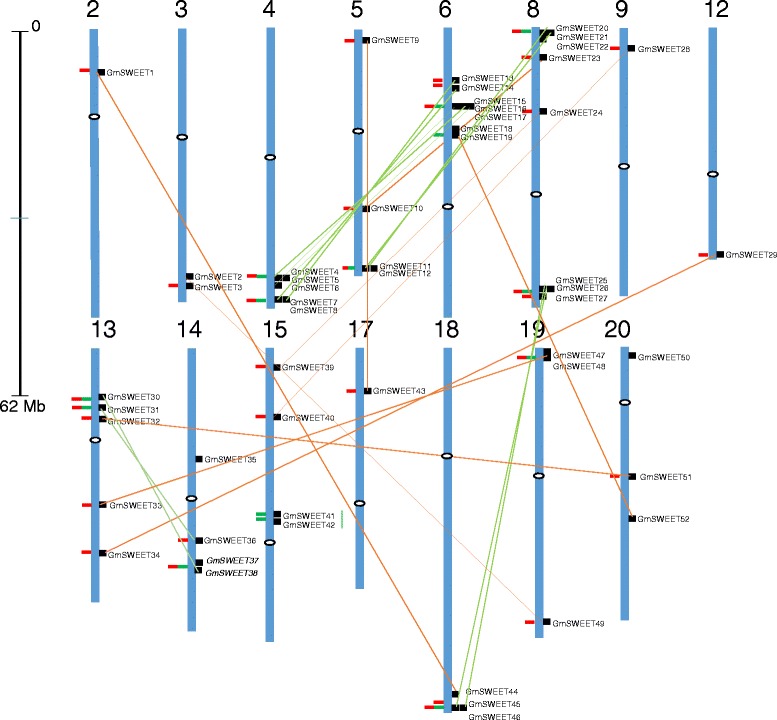
Chromosomal locations of soybean SWEET genes. The 52 SWEET genes were mapped to the 15 out of 20 chromosomes. Black boxes represent the gene position on the chromosome. The data used to generate the schematic diagram of the genome-wide chromosome organization was obtained from Phytozome and SoyKB genome browsers. Tandem and block duplications are marked with bold green and red boxes, respectively. Homologues were connected by orange (non-clustered genes) and light green (clustered genes) lines. Black scale line represents the length of chromosome. White dots on each chromosome represents centromere position

The phylogeny of the SWEET genes points to several duplication events. Out of 411 SWEET genes across 25 genomes, 56 tandem genes, 95 block duplication events, 72 genes were found to be both tandem and block duplication events (Fig. 1). The multiple sets of SWEET genes were first appeared in *S. moellendorffii* through duplication events. The non-vascular plant group (Chlorophyta and *P. patens*) did not show any gene duplication events. In soybean, 52 SWEET genes were mapped to 15 chromosomes and a majority were distributed in the more gene-dense euchromatic region near the chromosome ends (Fig. 3). The genes and clusters showed random distribution among the chromosomes. Chromosome numbers 2, 9, 12, and 17 contain only one SWEET gene, while chromosome 8 contains eight, the maximum number of SWEET genes per chromosome. It is known that polyploidy is a crucial force in plant evolution, and many angiosperms have experienced one or more episodes of polyploidization which subsequently resulted in gene duplication within the gene family [44, 45]. Soybean paralogs within a gene family were derived from genome duplications that occurred approximately 130 million years ago (MYA) (before the origin of rosids), 59 MYA (during legume genome duplication), and 13 MYA (duplication in the Glycine lineage) and nearly 75 % of the genes are present in multiple copies [44, 46]. In soybean, 21 *GmSWEET* sister pairs were identified with higher bootstrap values (<90 %) and the duplication of genes in soybean resulted in gene family expansion. Interestingly, we found clusters of five genes (*GmSWEET* 4 to 8 and *GmSWEET* 13 to 17) that were tandemly duplicated between chromosome 4 and 6. Similar tandem duplication clusters were observed between chromosome 5 and 8 and chromosome 8 and 18 (Fig. 3). The synonymous substitution rates (Ks), the non-synonymous substitution rates (Ka) and the Ka/Ks ratio for the 21 duplicated gene pairs revealed high similarities in their coding sequence alignments. The Ks values of these 21 genes ranged from 0.03 for gene pair *Glyma05G02070/Glyma17G09840* to 0.18 for pair *Glyma04G37520/Glyma06G17530* with an average Ks of 0.105 (Table 2), which is consistent with genes that emerged from the most recent genome duplication event 13 MYA [46, 47]. The history of selection performed on coding sequences can be measured by the Ka/Ks ratio and can be used to identify pairwise combinations of genes, where encoded proteins may have changed function [48]. Ka/Ks < 1 indicates that those genes underwent a purifying (stabilizing) selection and Ka/Ks > 1 at specific sites indicate genes that are under positive selection or Darwinian selection [47]. Table 2 summarizes the Ka/Ks for 21 duplicated pairs, in which 20 pairs were less than 0.9, indicating purifying selection and one pair (*Glyma05G02070/Glyma17G09840*) had a value of 1.79 indicating the positive selection. Based on the divergence rate of λ = 6.1×10^−9^ proposed for soybean [49], 20/21 SWEET paralogous pairs were estimated to have occurred between 4.95 to 14.9 MYA, except one pair at 2.88 MYA.

**Table 2:** Identification of substitution rates for homologues *GmSWEET* genes

Gene ID	No. of syn sites	No. of non-syn sites	Syno. substitution rate (Ks)	Non-syn substitution rate (Ka)	Ka/Ks	Duplication Date (MYA)
*GmSWEET29*	*214.9*	*490.1*	*0.0982*	*0.0229*	*0.2329*	8.05
*GmSWEET34*						
*GmSWEET41*	*179.6*	*489.4*	*0.1137*	*0.098*	*0.8627*	9.32
*GmSWEET42*						
*GmSWEET7*	*156.9*	*365.1*	*0.0983*	*0.011*	*0.1121*	8.06
*GmSWEET14*						
*GmSWEET30*	*210.8*	*551.2*	*0.1069*	*0.0225*	*0.2103*	8.76
*GmSWEET38*						
*GmSWEET8*	*207.2*	*533.8*	*0.1056*	*0.0112*	*0.1063*	8.66
*GmSWEET13*						
*GmSWEET31*	*209.6*	*534.4*	*0.0977*	*0.0346*	*0.3548*	8.01
*GmSWEET36*						
*GmSWEET1*	*218.2*	*567.8*	*0.1049*	*0.0259*	*0.2464*	8.60
*GmSWEET44*						
*GmSWEET6*	*222.4*	*614.6*	*0.09*	*0.0442*	*0.4912*	7.38
*GmSWEET15*						
*GmSWEET12*	*215.2*	*492.8*	*0.1059*	*0.0318*	*0.3001*	8.68
GmSWEET20						
*GmSWEET11*	*191.7*	*570.3*	*0.0866*	*0.0122*	*0.1408*	7.10
*GmSWEET21*						
*GmSWEET4*	*224.1*	*549.9*	*0.1265*	*0.0318*	*0.2513*	10.37
*GmSWEET17*						
*GmSWEET5*	*220.5*	*556.5*	*0.1823*	*0.0396*	*0.2172*	14.94
*GmSWEET16*						
*GmSWEET24*	*182.1*	*537.9*	*0.0955*	*0.0415*	*0.4342*	7.83
*GmSWEET39*						
*GmSWEET10*	*239.6*	*579.4*	*0.1145*	*0.0287*	*0.251*	9.39
*GmSWEET23*						
*GmSWEET26*	*234.6*	*569.4*	*0.0605*	*0.0108*	*0.178*	4.96
*GmSWEET45*						
*GmSWEET25*	*213.8*	*599.2*	*0.1066*	*0.0223*	*0.2094*	8.74
*GmSWEET46*						
*GmSWEET52*	*123.3*	*395.7*	*0.1523*	*0.1129*	*0.7409*	12.48
*GmSWEET19*						
*GmSWEET40*	*202.3*	*529.7*	*0.1572*	*0.0604*	*0.3845*	12.88
*GmSWEET28*						
*GmSWEET33*	*212.7*	*522.3*	*0.089*	*0.0198*	*0.2224*	7.30
*GmSWEET48*						
*GmSWEET9*	*200*	*478*	*0.0352*	*0.0633*	*1.7948*	2.89
*GmSWEET43*						
*GmSWEET32*	*205.6*	*562.4*	*0.1257*	*0.0259*	*0.2057*	10.30
*GmSWEET51*						

### Conserved domains

**Fig. 4:**
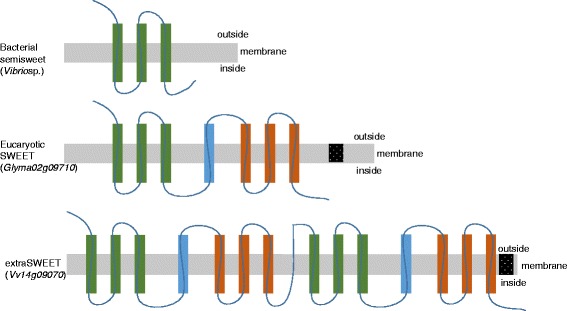
Conserved domain architecture of SWEET proteins. SWEET proteins classified into 3 major types based on number of 3-TM domains (Additional file 4). Proteins with single 3-TM domain classified as semiSWEET [50]; proteins with two 3-TM (7 α-helical) classified as SWEET genes [43]; and proteins with four 3-TMs were named as extraSWEET genes. Black box shows the associated protein domains, position of associated protein domains could be -N or -C terminal (Additional file 4). Not drawn to scale

The typical SWEET protein contains seven TM helices consisting of two tandem repeats of 3-TM units separated by a single TM unit [43]. Prokaryotes have homologues with only 3-TM units (semiSWEETs), which assemble into multiple 3-TM unit complexes to mediate sucrose transport [43, 50]. On the other hand, eukaryotes have both 7-TM and 3-TM SWEET genes. The eukaryotic 7-TMs have evolved by internal duplication of the 3-TMs [43] (see Fig. 4 for overall structural relationship of the sub-types). To understand the conservation of different domain within the gene family the protein sequences were aligned. On average, SWEET proteins in plants contain 5 exons that form a protein with an average of 248 amino acids. We found that out of 411 SWEETs, 140 were semiSWEET genes, each either missing the first or the second 3-TM domain, or they were present only in a partial form (Data not shown). In most SWEET genes, the second TM domain was found to be conserved rather than the first domain. A search for conserved domain architecture (using Conserved Domain Architecture Retrieval Tool [51]) resulted in three major types, as outlined in Fig. 4. These major types were further grouped into nine sub-types and they differed either in the position of *MtN3_slv* or they had regions with homology to other types of domains (e.g. receptor kinase, cuperdoxin, RNase H) and signal peptides (Fig. 4, Additional file 4).

As an interesting side finding, we found one SWEET protein from *V. vinifera* (*Vv14G09070*) that has duplication of 7-TM within the gene (Fig. 4, Additional file 4). This is a novel sub-type which we named extaSWEET. The extraSWEET gene could be another internal duplication of 7-TM, similar to the duplication of semiSWEET (3-TM) to evolved in SWEET gene (7-TM) [43]. *V.vinifera* accumulates high levels of sugar compounds in their berries and this extraSWEET gene might have a role to mediate more sucrose transport. It has been reported that sucrose (*VvSUC*) and hexose (*VvHT*) transporter genes are preferentially expressed during berry development in *V. vinifera* [52]. In addition to the *VvSUC* and *VvHT*, it would be interesting to see the expression sites and function of *VvSWEET* (*Vv14G09070*) for long distance sugar transport during flower and/or berry development in *V. vinifera*.

The protein architecture and TM domains in soybean were conserved showing 36 SWEET genes with 7-TMs (SWEET), and the rest had less than 6 TMs (partial/semiSWEET) (Additional file 5). In addition to this, conserved *cis-*elements in the proximal promoter region (2 Kb upstream) among 52 *GmSWEET* genes were identified using INCLUSive MotifSampler [53]. Identification and comparing the *cis*-motif consensus pattern and discovery of expression modules within gene co-expression networks are crucial to understand the common regulatory networks. The top five significant *cis*-motif patterns were sampled from *GmSWEET* genes (Additional file 6). Motifs such as TBP binding sites, GT-2 (Grass TF 2), ATHB1 (*A. thaliana* Homeobox 1), HAHB4 (*H. annuus* Homeobox 4) and TaMYB80 (*T. aestivum* MYB80) were identified in SWEET gene promoters, indicating differential regulation and also they might have a putative role of sugar signaling [54] (Additional file 6). Interestingly, *cis*-motif elements of GT-2 and GT-3 were significantly enriched in soybean SWEET genes (Additional files 6 and 7). GT-2, −3 are plant transcriptional activators in higher plants and are involved in seed development and other diverse functions in rice, Arabidopsis and soybean [55, 56]. Further functional characterization of these *cis*-regulatory motifs and TFs (Transcription Factor) binding sites in *GmSWEET* genes will be helpful to understand the precise roles in development.

### Soybean SWEET genes are highly expressed during reproduction and seed development

**Fig. 5:**
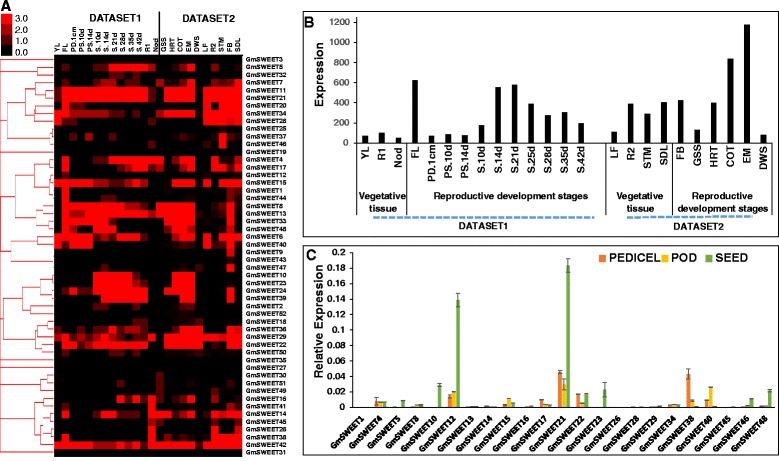
Expression profiles of soybean SWEET genes in different tissues. **a** Hierarchical cluster of expression profiles from two RNA-seq datasets in 24 tissues covering the whole life cycle of soybean (Williams 82). Sources of the samples are as follows: Dataset 1 - YL (young leaves), FL (flower), PD.1 cm (one cm pod), PS.10d (pod shell 10 Days After Flowering (DAF)), PS.14d (pod shell 14DAF), S.10d (seed 10DAF), S.14d (seed 14 DAF), S.21d (seed 21DAF), S.25d (seed 25DAF), S.28d (seed 28DAF), S.35d (seed 35DAF), S.42d (seed 42DAF), R1 (root), and Nod (nodule); Dataset 2 - GSS (globular stage whole seed), HRT (heart stage), COT (cotyledonary stage), EM (early maturation), DWS (dry whole seed), LF (leaf), R2 (root), STM (stem), FB (floral bud), and SDL (seedling). **b** Expression pattern of 52 *GmSWEET* genes. Bars show the expression of all genes in different developmental stages from both datasets. For simplicity, the datasets were marked with vegetative and reproductive stages. **c** qRT-PCR analysis of 21 selected *GmSWEET* genes in pedicel, pod, and seed tissues

To understand the roles of specific *GmSWEET* genes in different developmental stages, we compared the expression profiles of all soybean SWEET genes using two publicly available RNA-seq datasets. The first dataset contains 14 tissues including whole seed at 11 stages of reproductive tissue development (flower, pod, and seeds) and three vegetative tissues (leaves, root, and nodules) [57]. The second dataset contains 10 tissues including 6 reproductive tissues (floral buds, whole seeds at five stages of seed development i.e. globular, heart, cotyledon, early-maturation, dry), and four vegetative tissues (leaves, roots, stems, and seedlings) (GEO Accession GSE29163; Goldberg et. al. unpublished). Among all SWEET genes, *GmSWEET21* and *GmSWEET24* showed the highest expression in both of the datasets (Fig. 5a). The expression of 23 genes was either very low or undetectable in the datasets, hence they might be pseudo-genes or they might be expressed in certain tissues or conditions (Fig. 5a, Additional file 8). The gene expression pattern is varied in different developmental stages. Most of the genes were up-regulated during flower and seed development; several of them could be specific to these stages. It is noteworthy that the overall SWEET gene expression increased gradually during seed filling and then declined towards seed maturation (Fig. 5a, b). This suggests that the SWEET transporter plays a crucial role in nutrient unloading during seed development and seed filling. Overall results support earlier studies which concluded that most of the SWEET genes are related to reproductive development than other physiological processes [20, 58, 59].

In the present study 21 paralogous gene pairs for *GmSWEET* were identified (Fig. 3). The relationships between paralogous *GmSWEET* pairs with their expression pattern during development was compared. Nine out of 21 pairs showed a similar expression pattern and rest showed divergence in expression patterns (Additional file 9). For example, paralog pair *Glyma05g38340* (*GmSWEET11*) and *Glyma08g01310* (*GmSWEET21*) were up-regulated in cotyledonary tissue while simultaneously being down-regulated in leaf tissue. Similar expression levels of paralog genes suggests that they have retained the promoter element. Expression patterns of the remaining 12 paralog pairs has diverged (Fig. 5a), to either non-functionalization, neo-functionalization or sub-functionalization. Therefore, it would be interesting to see the expression pattern of those genes in soybean under different conditions. In soybean, SWEET genes are also associated with the iron deficiency [60]. Lauter et. al. (2014) observed the repression of two SWEET genes (*Glyma05g38340* and *Glyma08g01310*) and other sucrose transporter genes in the leaves one hour after iron stress and concluded that SWEET genes might play a role in regulation of the SnRK1/TOR (SNORKEL) signaling pathway in response to iron deficiency [60].

### Examination of SWEET gene expression in reproductive tissue by qRT-PCR

To confirm the expression patterns determined by the RNA-seq analysis, qRT-PCR was employed to analyze the expression patterns of 23 genes in three reproductive development tissues of soybean, Williams 82 (W82), specifically pedicel, pods, and developing seeds (Fig. 5c). The expression patterns (Fig. 5c) were largely consistent with those obtained by the RNAseq analysis (Fig. 5a), even though some smaller variations can be seen. GmSWEET 12 and 21 were highly expressed in all three developmental stages, but in the seeds they are so abundant that the total relative SWEET gene expression far exceeds that of the other tissues (Fig. 5c). The expression of GmSWEET genes 5, 10, 23, and 48 were also much higher in seeds than in the other tissues, and may be considered seed-specific. In pods, GmSWEET 12, 21, and 40 had comparatively higher expression, and in pedicels the expression of GmSWEET 12, 21, and 38 stands out.

### Exploring natural variation in *GmSWEET* genes using soybean whole genome re-sequencing data

The elucidation of the soybean SWEET genes gave us an unprecedented opportunity to obtain a comprehensive overview of the allelic variation in soybean whole genome re-sequencing data. The wealth of whole genome resources of soybean provides a unique angle to study natural variation in germplasm and further allows functional characterization of the particular gene [61–63]. Complete genome sequences for 106 soybean genotype, sequenced at approximately 15X coverage, were obtained from the Soybean Genetics and Genomics Laboratory at The University of Missouri (Valliyodan et. al. Unpublished) and analyzed for synonymous and non-synonymous SNPs, premature stop codon and haplotype variation in selected *GmSWEET* genes. In Arabidopsis, *AtSWEET11* and −*12* double mutants accumulated sucrose in the leaves and had lower levels in the phloem, identifying them as the long sought main sucrose effluxers in the leaf sugar export pathway [26]. It has been observed that when *AtSWEET17* expression is reduced, either by induced or natural variation, fructose accumulates in the leaves, suggesting an enhanced storage capacity [64]. Site directed mutagenesis of *AtSWEET1* at four conserved positions (P23T, Y57A, G58D, and G180D) led to abolishment of glucose transport activity in a yeast complementation assay. Also, SNP in the coding or promoter region can also abolish protein localization and function [43]. In the present study, wide natural variations were observed in non-synonymous SNPs and a total of 37 SNPs were observed in 21 (~40 %) *GmSWEET* genes (Table S5). *GmSWEET41* (*Glyma15g27530*) showed a premature stop codon in the 1st exon in 15 sequenced lines.

To understand and visualize the genetic variation in whole genome re-sequencing data for the SWEET genes, a cluster of genes (*GmSWEET15, 16, and 17*) including their 2 kb promoter region was examined. The haplogroup gave three major distinct clusters based on the SNP variation in promoter and coding regions similar or dissimilar to the soybean reference genome, W82 (Additional file 10). As sugar derivatives are associated with SWEET genes [8, 43], we further examined the association between the haplogroup cluster and different sugar content (sucrose, raffinose, and stachyose) in soybean seeds and observed a correlation between three SNP-haplogroups and average sucrose content. The SNP-haplogroup similar to reference genotype W82 showed intermediate sucrose concentration of average 5.26 ± 0.14 %. The other two groups were distinct from W82 haplogroup showing an average sucrose concentration of 4.8 ± 0.4 % and 5.5 ± 0.28 %, (Additional file 10). Out of 10 wild soybean lines (*G. soja*), seven lines were identified in the first haplogroup which showed a relatively lower sucrose content. No significant association was found for raffinose and stachyose concentrations. It has been reported that the transport of Raffinose family oligosaccharides (RFOs) are not detectable when associated with apoplastic loading [23, 65] and several higher plants accumulate RFO during the seed maturation process [66], hence SWEET genes might have no role in efflux for RFOs. However, to fully understand their roles, detailed functional characterization of the individual gene is needed.

## Discussion and conclusions

*In-silico* analysis and phylogenetic studies generate valuable information on the evolutionary and functional relationships between genes of different species, genomic complexity, and lineage-specific adaptations. Previous work on sugar transporter genes SWEET *(MtN3_slv*), along with the rapidly expanding availability of genomics sequence data has enabled us to examine the SWEET content of multiple plant genomes.

The SWEET gene family has been studied in Arabidopsis [15, 26], rice [20, 58, 59] and bacteria [43, 50]. However, this family has not previously been studied in soybean. Here, we explore these genes in soybean with an analysis of their phylogeny, gene structure, domain architecture, expression profiles and natural genetic variation. A total of 52 full-length SWEET genes were identified in the soybean genome, which is highest among the analyzed plants and implies a genome expansion. The exon/intron layouts and the TM motifs were quite conserved when compared to the paralogs. A phylogenetic tree was constructed (Fig. 2) to identify putative orthologous and paralogous SWEET genes and to study the pattern of the SWEET gene family expansion in the course of evolution.

The salt water living chlorophyta algae *O. tauri, O. lucimarius and Micromonos sp*. have only a single gene. On the other hand, the fresh water algae, *V. carteri and C. reinhardtii,* contain 2 and 3 SWEET genes, respectively. This leads us to suspect that during the transition phase to fresh water, a more involved mechanism for sugar transport was required by environmental conditions. The evolution to multi-cellularity led to further expansion of the SWEET gene family. Recent studies on the evolution of the SUT transporter family showed that divergence of different SUT types were likely associated with evolution of vascular cambium and phloem transport [34]. Higher plants evolved phloem for long-distance, source-to-sink transport. Although different phloem loading strategies are recognized, lineages that evolved apoplasmic phloem loading required a mechanism for efflux from phloem parenchyma and subsequent energized uptake into the companion cell/sieve element complex, SWEETs provided the former function [6]. *P. patens* is an early diverging land plant and many families of *P. patens* genes for metabolic enzymes (e.g. cytokinin [67], glutathione [68], pectin [69]) have large copy numbers. *P. patens* has only a primitive protophloem, and the increase in the SWEET genes here could be due to the recent genome duplication [70], without the new genes necessarily having acquired differentiated functionalities. *S. moellendorffii* does have a phloem, and the number of SWEET genes here approaches that of many angiosperms (Fig. 1).

The expansion of a gene family in higher plants indicates the differentiation of physiological function of each isoform in terms of the expression site and the regulatory manner which subsequently helps the organism to adapt in different environmental conditions. The internal duplication of the 3-TM (semiSWEET) gene must have happened early to give rise to new genes with 7-TMs (SWEET) which allow a more sophisticated sucrose transport [43, 50, 71]. Here we also report a novel gene in *V. vinifera* which has further duplicated the TM regions. Collectively, phylogenetic and domain studies imply that biological, physiological or environmental conditions forces particular gene families to evolve and expand. As evolution of the higher plants have progressed, some species have acquired further SWEET genes (Fig. 1). This suggests that sugar transport evolution has followed as new plant structures and adaptations to new ecological niches have arisen.

The SWEET genes play a diverse functional role during plant development which is evident from their expression patterns in other plant species [25, 40, 43, 50, 58] and soybean (this report). In rice and Arabidopsis, the expression of the SWEET genes were relatively higher in flower, pollen, embryo sac and seeds suggesting their roles in reproductive tissue development [15, 19, 20]. In rice two members of the SWEET gene family were highly expressed in panicles and anthers and were associated with fertility and seed size [20, 58, 59]. In Arabidopsis, *AtSWEET8* was expressed in the embryo sac suggesting that it might regulate female gametocyte development [72]. Developing seeds are the strongest sink tissues in many plants and they need a higher carbon source for development which implies that nutrient transporters including the SWEET genes might be key component for their development. In Arabidopsis, *AtSWEET11* and −*12* showed a higher expression in leaves and had important roles in leaf sucrose export [15]. The comparison of *AtSWEET11* and −*12* expression pattern with soybean orthologs *GmSWEET6,* and −*15* showed a relatively higher expression in leaves, suggesting that these genes also might have similar role in leaf sucrose export.

Yuan and Wang [20] and Chen [25] have reviewed the functional role of SWEET genes in different tissues, pathogen infestation, and environmental responses. Interestingly, *GmSWEET13*, *14* and *15* fall under the fungal disease resistance QTL on chromosome 6 in soybean [73]. It has been proven that fungal and bacterial symbionts induce SWEET gene expression for nutritional gain during pathogen infestation [15, 25, 40, 74, 75]. The statement that most of the reported SWEET genes are associated with reproductive development tissue is corroborated in this study using soybean transcriptome datasets. The transciptome and qRT-PCR data showed that multiple SWEET genes are expressed at higher levels in tissues involved in reproductive development. Relatively higher expression of *GmSWEET5*, −*10*, −*23* and −*48* in the seed tissue, suggest that collectively these genes might assist the movement of sucrose in the developing soybean seeds. Unloading of nutrient in the developing seeds occurs from the seed coat [32, 76]. In the developing legume seeds (*P. vulgaris* and *P. sativum*), a suite of sucrose transporters are expressed at a higher levels in seed coat tissue to facilitate the movement of sucrose [36]. Sugar availability, starch content, and cytokinin levels are involved in the regulation of abscission of soybean flowers, the delay of which hampers seed development and leads to yield loss in soybean [77–79]. Soybean flower abortion is primarily caused by deficiency in or competition for photo-assimilates and nutrients among growing organs.

Beside the expression level, the genetic variation (natural or induced) also enforces the functionality of SWEET genes and causes a variation in phenotype [43, 64]. Mutation in the SWEET gene or abolishing the activation of the SWEET promoter leads to resistance to bacterial pathogens in rice [5, 59, 80]. Identification of several non-synonymous SNPs and large effect SNPs in *GmSWEETs* are expected to affect the integrity of encoded proteins. Additionally, exploring SNP-haplotype diversity using whole-genome sequencing data mining provides a powerful resource for investigating diversity in a particular gene family [81–83]. The data presented here, using a cluster of genes on chromosome 6 (*GmSWEET13, −14* and −*15*), showed the association between the SNP-haplogroups and sucrose content in seeds. The allelic variation data presented in this study provides a valuable resource for association studies between the SNPs and important agronomic traits, although intensive studies with each candidate gene are required to examine this inference. Overall, the SWEET gene family signifies its role as a key component in reproductive tissue development, nutrient unloading and pathogen resistance. Manipulating SWEET expression in specific tissues (phloem sap, pedicel, and developing seeds) could enhance sugar delivery to developing seeds to increase yield.

## Methods

### Sequence and database search for SWEET gene family

SWEET (*MtN3_slv)* gene families were identified from 25 completely sequenced genomes representing the plant lineage (green plants) including members from unicellular green algae to multicellular plants (Fig. 1, Additional file 1). The protein BLAST search was performed using *AtSWEET11* as a query sequence in Plaza [42] (http://bioinformatics.psb.ugent.be/plaza/news/index) and Phytozome [84] (http://www.phytozome.org) databases and the sequences were retrieved from the corresponding plant genome annotation resources and analyzed. The multiple sequence alignment was performed using MUSCLE program [85] and partial and redundant sequences were excluded. All proteins were examined for presence of *MtN3_slv* related TM domains (IPR018179) using Interpro database [86] (http://www.ebi.ac.uk/). *Glycine max* SWEET genes were designated as *GmSWEET1* to *GmSWEET52*.

### Phylogenetic analysis

To understand the phylogenetic relationship, 173 SWEET genes from 13 species representing major clades were analyzed. Protein sequences were analyzed by the neighbor-joining (NJ) method [87] with genetic distance calculated by MEGA5.1 [88] (www.megasoftware.net/). The numbers at the nodes represent bootstrap percentage value based on 1,000 replications.

### Identification of conserved domains and *cis-*motif pattern

The Conserved Domain Architecture Retrieval Tool (CDART) [51] (http://www.ncbi.nlm.nih.gov/Structure/lexington/lexington.cgi) was searched using Arabidopsis *AtSWEET11* as a query protein sequence (Additional file 4). Several *MtN3_slv* TM domains were preceded which were grouped into three major architecture based on 3-TMs and associated proteins (Fig. 4). Identification of the exon/intron organization of SWEET genes was performed by aligning cDNAs with their corresponding genomic DNA sequences and were also obtained by using the Plaza comparative database. *Cis* regulatory elements were identified by searching 2 kb upstream of the 5’ translation start base for all of the soybean SWEET genes using INCLUSive MotifSampler [89]. 2 kb upstream sequences were annotated by similarity search (*p* value <0.05, motif score >5) with known plant transcription binding sites and motifs available in the Athamap database [90] (www.athamap.de, Additional files 6 and 7).

### Soybean SWEET gene chromosomal location and gene duplication

The location of soybean SWEET genes was determined based on their physical positions on chromosomes corresponding to their locus numbers in the SoyKB browser [91]. The duplication of SWEET genes on segmentally duplicated regions was determined using Plaza 2.5 whole genome mapping tool (http://bioinformatics.psb.ugent.be/plaza/versions/plaza_v2_5/genome_mapping/genome_mapping), and were visualized using genome search and synteny view tool (CViT) (http://comparative-legumes.org/) [92]. The comparative duplicate block representing homologous chromosome segments were anchored on 15 out of 20 soybean chromosomes and indicated by tandem/block duplication (Fig. 3).

### Calculation of Ka/Ks values

Non-synonymous (Ka) to synonymous (Ks) substitution rates were used to estimate the selection mode for all orthologous gene pairs of soybean SWEET family [48]. Subsequently, the PAL2NAL program (http://www.bork.embl.de/pal2nal/) was used to convert a multiple sequence alignment of proteins and the corresponding DNA (or mRNA) sequences into a codon alignment [93]. PAL2NAL automatically calculates Ks and Ka by the CODEML program in PAML. The divergence time (T) was calculated by T = Ks/(2 × 6.1 × 10^−9^)×10^−6^ MYA, where 6.1 × 10^−9^ is divergence rate in millions of years translated from Ks value [49].

### RNA-seq datasets and qRT-PCR analysis

Genome-wide public RNA-seq datasets (Reads/Kb/Million (RPKM) normalized data) for soybean developmental stages were downloaded from soybean RNA-seq Atlas [57] and Gene Expression Omnibus (GEO) database (accession number GSE29163) from Goldberg et. al. (Unpublished). Sources of the samples for first dataset are as follows: YL (young leaves), FL (flower), PD.1 cm (one cm pod), PS.10d (pod shell 10 Days After Flowering (DAF)), PS.14d (pod shell 14DAF), S.10d (seed 10DAF), S.14d (seed 14 DAF), S.21d (seed 21DAF), S.25d (seed 25DAF), S.28d (seed 28DAF), S.35d (seed 35DAF), S.42d (seed 42DAF), R1 (root), and Nod (nodule). Sources of the samples for second dataset are as follows: GSS (Globular stage whole seed), HRT (Heart stage), COT (Cotyledonary stage), EM (Early maturation), DWS (Dry whole seed), LF (Leaf), R2 (Root), STM (Stem), FB (Floral bud), and SDL (Seedling). Average linkage method provided in Cluster 3.0 was used to cluster gene and tissue types and visualized using TreeView software [94].

Total RNA was extracted from soybean pedicel, pod, and seed tissues using a Qiagen RNeasy mini kit (Qiagen, CA, USA). First strand cDNA from 1 μg of total RNA was synthesized by using Superscript III reverse transcriptase (Invitrogen) with oligo(dT) primer. Primers for quantitative reverse transcription PCR (qPCR) were designed using Primer3 (http://frodo.wi.mit.edu) (Additional file 11). Quantitative RT-PCR was performed using cDNA product in a 10 μl reaction volume using Maxima SYBR Green/ROX qPCR master mix (Thermo, USA) on ABI7900HT detection system (Life Technologies, NY, USA). Three biological replicates and two technical replicates were used for analysis. The PCR conditions were: 50 °C for 2 min., 95 °C for 10 min., then 40 cycles of 95 °C for 15 sec., and 60 °C for 1 min. To normalize the gene expression, Actin (*Glyma18g52780*) was used as an internal control.

### Analysis of sequence variants, non-synonymous SNP and haplotype variation

One hundred and six soybean lines with carbohydrate phenotypes (sucrose, stachyose, and raffinose) and whole genome re-sequencing (sequencing depth approximately 15X) data were obtained for soybean SWEET genes from Soybean Genetics and Genomics Laboratory at the University of Missouri (Valliyodan et. al. Unpublished). The processed data was aligned to the Williams 82 Gmax v9.0 from Phytozome as the reference genome [46]. SNPs were identified using an in-house built pipeline using with SOAP3 [95] and were analyzed for possible synonymous/non-synonymous SNP variation annotations using SnpEFF [96] and v9.0 gene models from Phytozome (Additional file 12). SNP haplotypes were examined by generating map and genotype data files using TASSEL 5.0 program [97] and then clustering pictorial output for a specific genic region was visualized using FLAPJACK software [98].

### Availability of supporting data

All supporting data of this article are included as additional files.

## Additional files

Additional file 1:
**SWEET gene family across 25 plant genomes.**
Additional file 2:
**Amino acid sequences of 173 SWEETs from 13 species.**
Additional file 3:
**Gene organization of soybean SWEET orthologs genes.**
Additional file 4:
**Conserved domain and associated protein architecture in SWEET (** ***MtN3_slv*** **) gene family.**
Additional file 5:
**Soybean SWEET protein Transmembrane Helix prediction obtained from TMHMM 2.0 tool [** 99 **].**
Additional file 6:
***cis*** **-motif analysis of soybean SWEET genes.** Conserved motifs identified in proximal promoter region of SWEET gene family using INCLUSive MotifSampler and its similarity with known motifs available in Athmap database. Similarity search performed using STAM tool (www.benoslab.pitt.edu/stamp). Additional file 7:
**Detailed information of** ***cis*** **-motifs in upstream promoter region of soybean SWEET genes.**
Additional file 8:
***GmSWEET*** **gene transcriptome profile in public datasets.**
Additional file 9:
**Expression profile of** ***GmSWEET*** **paralogue gene pairs.**
Additional file 10:
**SNP-Haplotype analysis of SWEET gene cluster on chromosome 6.** Hierarchical clustering showed the association between average sucrose content and SNPs haplogroups in 106 lines. Base position identical to reference (Williams 82) are light sky blue, black – different, gray-missing data. Blue colored bar on top showing approximate position of *GmSWEET15, −16* and −*17*. Additional file 11:
**Primer sequences of 23 selected** ***GmSWEET*** **genes for RT-qPCR analysis.**
Additional file 12:
**Identification of non-synonymous SNP in 52** ***GmSWEET*** **from 106 soybean re-sequencing data (Valliyodan et al. Unpublished).** The Seq_id with underline represents wild soybean lines (*G. soja*).
