# Supplementary material for: Soybean (Glycine max) SWEET gene family: insights through comparative genomics, transcriptome profiling and whole genome re-sequence analysis
Source: BMC Genomics. 2015 Jul 11;16(1):520. doi: 10.1186/s12864-015-1730-y (PMC4499210; doi:10.1186/s12864-015-1730-y)
Supplement: Additional file 1: — SWEET gene family across 25 plant genomes. [file 12864_2015_1730_MOESM1_ESM.pdf]

Additional file 1: SWEET gene family across 25 plant genomes.

	PLAZA ID (2.5)	No of intron	Amino acid	Outliers
A. lyrata	AL0G04140	7	294	
	AL0G06660	5	224	
	AL0G10280	3	182	
	AL1G22660	5	247	
	AL1G32720	0	143	Outlier
	AL2G14350	0	261	
	AL3G06610	4	227	
	AL3G06980	5	214	
	AL3G15550	5	237	
	AL3G17890	5	230	
	AL4G25490	5	258	
	AL5G07210	5	252	
	AL5G13860	5	285	
	AL5G17680	5	289	
	AL6G12880	5	295	
	AL6G33300	4	258	
	AL7G17110	5	280	
	AL7G18250	1	81	Outlier
	AL7G27640	7	241	
	AL7G37710	5	240	
	AL8G14940	5	289	
	AL8G14950	5	294	
	AL8G17790	5	260	Outlier
	AL8G18970	5	290	
	AL8G29370	5	240	
A. thaliana	AT1G21460	5	247	
	AT1G66770	0	261	
	AT2G39060	5	258	
	AT3G14770	5	236	
	AT3G16690	5	230	
	AT3G28007	5	251	
	AT3G48740	5	289	
	AT4G10850	4	258	
	AT4G15920	5	241	
	AT4G25010	5	281	
	AT5G13170	5	292	
	AT5G23660	5	285	
	AT5G40260	5	239	
	AT5G50790	5	289	

	AT5G50800	5	294	
	AT5G53190	5	263	
	AT5G62850	5	240	
B. distachyon	BD1G32920	4	251	
	BD1G62570	3	298	
	BD1G62890	5	312	
	BD2G11920	5	255	
	BD2G24850	5	256	
	BD2G32230	5	250	
	BD2G40537	5	238	
	BD2G42990	4	246	
	BD2G47540	5	231	
	BD2G56890	5	259	
	BD3G10270	4	251	
	BD3G41410	4	299	
	BD3G44260	3	309	
	BD4G07570	3	292	
	BD4G18350	3	300	
	BD4G19730	1	241	
	BD4G34780	4	291	
	BD5G08440	3	216	
C. papaya	CP00003G00570	5	252	
	CP00004G01330	5	292	
	CP00004G01350	5	292	
	CP00009G01680	5	278	
	CP00016G01990	1	87	Outlier
	CP00048G01020	5	246	
	CP00048G01030	4	262	
	CP00049G00770	5	246	
	CP00049G00780	5	240	
	CP00051G00690	5	265	
	CP00051G00750	0	64	Outlier
	CP00099G00590	5	278	
	CP35275G00010	2	235	
	CP36848G00010	2	140	Outlier
	CP36958G00010	1	83	
C. reinhardtii	CR06G04860	8	249	
	CR06G05510	6	231	
	CR07G05980	4	247	
	CR10G00940	4	200	Outlier
F. vesca	FV0G09350	5	310	
	FV0G09360	5	292	
	FV0G13510	4	276	

	FV0G27360	11	818
	FV0G38430	3	187
	FV0G38440	4	242
	FV0G38630	4	164
	FV0G46230	4	159
	FV2G08560	6	249
	FV2G23650	5	243
	FV3G19570	5	354
	FV4G13510	5	241
	FV5G06020	3	207
	FV5G09850	6	309
	FV5G35520	4	253
	FV7G20910	5	235
	FV7G36880	5	214
G. max	Glyma02g09710	5	262
	Glyma03g36790	8	316
	Glyma03g39430	4	155
	Glyma04g37510	5	259
	Glyma04g37520	5	283
	Glyma04g37530	4	277
	Glyma04g41680	4	175
	Glyma04g42040	5	248
	Glyma05g02070	4	226
	Glyma05g25180	3	283
	Glyma05g38340	5	258
	Glyma05g38350	6	276
	Glyma06g12740	5	259
	Glyma06g13110	5	255
	Glyma06g17520	5	310
	Glyma06g17530	5	261
	Glyma06g17540	5	259
	Glyma06g21570	7	244
	Glyma06g21640	3	192
	Glyma08g01300	5	295
	Glyma08g01310	5	255
	Glyma08g02890	4	274
	Glyma08g08200	5	260
	Glyma08g19580	5	281
	Glyma08g47550	5	272
	Glyma08g47560	5	274
	Glyma08g48280	2	224
	Glyma09g04840	5	245
	Glyma12g36300	5	236

	Glyma13g08190	5	256	
	Glyma13g09140	5	249	
	Glyma13g10560	4	258	
	Glyma13g23860	5	246	
	Glyma13g33950	5	236	
	Glyma14g17810	7	181	
	Glyma14g27610	5	250	
	Glyma14g30740	6	247	
	Glyma14g30940	5	255	
	Glyma15g05470	5	250	
	Glyma15g16030	5	246	
	Glyma15g27530	5	262	
	Glyma15g27750	5	236	
	Glyma17g09840	5	227	
	Glyma18g53250	5	263	
	Glyma18g53930	5	269	
	Glyma18g53940	5	272	
	Glyma19g01270	4	232	
	Glyma19g01280	5	247	
	Glyma19g42040	5	308	
	Glyma20g01890	3	160	
	Glyma20g16160	4	257	
	Glyma20g21060	4	213	
L. japonicus	LJ0G031270	0	120	Outlier
	LJ0G082400	1	88	Outlier
	LJ0G099080	4	203	
	LJ0G110350	0	160	
	LJ0G114260	3	203	
	LJ0G136970	1	171	
	LJ0G184500	1	99	Outlier
	LJ0G239110	2	210	
	LJ0G283440	2	130	Outlier
	LJ0G460670	4	261	
	LJ4G004280	5	278	
	LJ4G031400	4	284	
	LJ4G031410	5	247	
	LJ5G005970	3	173	
	LJ6G019470	4	213	
	LJ6G019640	1	83	Outlier
M. domestica	MD00G051960	4	193	
	MD00G112340	5	263	
	MD00G113720	5	295	
	MD00G192470	6	250	

	MD00G252980	2	147	Outlier
	MD00G309540	5	291	
	MD00G345840	3	222	
	MD00G395450	5	212	
	MD00G395950	5	243	
	MD00G419620	5	291	
	MD00G420470	5	305	
	MD00G442000	5	245	
	MD00G458620	6	281	
	MD00G479620	6	335	
	MD00G504430	5	243	
	MD02G017480	3	274	
	MD03G021290	5	196	
	MD04G019210	5	267	
	MD05G006340	6	218	
	MD05G006370	9	440	
	MD06G012000	7	412	
	MD06G014440	5	236	
	MD09G016880	4	265	
	MD10G021450	5	232	
	MD11G027500	5	300	
	MD12G024790	9	714	Outlier
	MD13G009640	5	305	
	MD14G007550	2	154	
	MD14G014520	5	299	
	MD14G014530	2	171	
	MD14G014540	5	295	
	MD14G018060	5	249	
	MD15G013630	4	261	
	MD16G007750	5	305	
M. esculenta	ME00341G00230	3	224	
	ME00784G00040	5	240	
	ME01551G03720	5	237	
	ME01701G00430	5	279	
	ME01701G00440	5	282	
	ME01701G00450	5	280	
	ME01701G00460	5	284	
	ME01701G00470	5	278	
	ME01945G00760	5	241	
	ME02831G00080	5	234	
	ME03175G00590	4	259	
	ME03219G00280	5	239	
	ME03614G05160	5	251	

	ME06089G00240	5	247	
	ME06688G00170	5	250	
	ME06711G00450	6	263	
	ME06875G00010	3	194	
	ME07238G00800	5	226	
	ME07520G00280	5	250	
	ME09120G00410	3	188	
	ME09876G00540	5	281	
	ME09876G00550	3	225	
	ME09876G00560	2	174	
	ME11668G00050	5	302	
	ME11668G00060	4	171	
	ME11994G00020	4	260	
	ME11998G01750	5	271	
	ME12794G01170	5	238	
Micromonas sp. RCC299	MRCC299_04G06200	0	254	Outlier
M. truncatula	MT0G04170	5	235	
	MT0G35890	5	236	
	MT2G007890	5	288	
	MT2G008140	1	68	Outlier
	MT3G080990	4	263	
	MT3G090940	5	250	
	MT3G090950	5	250	
	MT3G098860	1	75	Outlier
	MT3G098910	4	311	
	MT3G098930	5	270	
	MT4G106990	4	263	
	MT5G007080	2	119	Outlier
	MT5G067530	5	269	
	MT5G092600	5	252	
	MT6G034600	3	111	Outlier
	MT7G005650	1	147	Outlier
	MT7G005690	4	268	
	MT7G005710	1	161	
O. lucimarinus	OL04G01590	0	242	
O. sativa japonica	OS01G12130	5	252	
	OS01G21230	5	175	
	OS01G36070	5	243	
	OS01G40960	5	180	
	OS01G42090	3	254	
	OS01G42110	3	259	
	OS01G50460	5	230	
	OS01G65880	3	227	

	OS02G19820	4	259	
	OS02G30910	3	319	
	OS03G22200	2	254	
	OS03G22590	4	300	
	OS05G12320	5	246	
	OS05G35140	5	261	
	OS05G51090	3	237	
	OS08G42350	4	307	
	OS09G08030	1	206	
	OS09G08270	0	98	Outlier
	OS09G08440	5	375	
	OS09G08490	1	78	Outlier
	OS11G31190	4	303	
	OS12G07860	1	240	
	OS12G29220	5	296	
O. sativa indica	OSINDICA_01G11170	3	181	
	OSINDICA_01G19870	3	154	
	OSINDICA_01G33510	5	242	
	OSINDICA_01G39890	3	254	
	OSINDICA_01G39960	1	128	Outlier
	OSINDICA_01G39980	3	259	
	OSINDICA_01G62510	7	314	
	OSINDICA_01G62530	3	100	Outlier
	OSINDICA_02G18690	4	259	
	OSINDICA_02G29780	3	319	
	OSINDICA_03G21370	5	331	
	OSINDICA_03G21780	4	300	
	OSINDICA_05G10580	4	211	
	OSINDICA_05G31510	5	285	
	OSINDICA_08G40860	5	319	
	OSINDICA_09G05510	1	248	
	OSINDICA_09G06050	0	266	
	OSINDICA_09G06090	0	134	Outlier
	OSINDICA_11G22420	4	308	
	OSINDICA_12G06870	1	240	
	OSINDICA_12G18430	5	293	
Ostreococcus tauri	OT04G01700	0	250	
P. patens	PP00039G01320	4	251	
	PP00054G00300	5	257	
	PP00127G00700	3	253	
	PP00240G00170	4	247	
	PP00245G00180	3	191	
	PP00307G00200	4	243	

P. trichocarpa	PT00G09440	3	230	
	PT01G13220	5	282	
	PT01G34670	5	242	
	PT01G35790	3	169	
	PT01G38440	4	219	
	PT02G07160	5	250	
	PT02G07170	3	205	
	PT02G07180	5	259	
	PT03G13360	4	255	
	PT03G15570	5	287	
	PT05G02180	3	217	
	PT05G19830	4	294	
	PT08G21810	1	79	Outlier
	PT08G21820	4	162	
	PT11G09710	5	235	
	PT12G01210	5	255	
	PT12G09930	5	271	
	PT13G01350	5	293	
	PT13G01360	5	269	
	PT13G01400	5	239	
	PT15G02110	5	239	
	PT15G07610	5	237	
	PT15G10400	5	269	
	PT15G10410	5	283	
	PT15G10420	5	283	
	PT15G10430	5	276	
	PT19G04400	3	230	
R. communis	RC27613G00200	0	233	
	RC27985G00530	5	248	
	RC29475G00120	0	236	
	RC29579G00020	5	249	
	RC29647G00280	5	286	
	RC29726G01760	5	244	
	RC29822G00230	5	242	
	RC29822G00240	3	194	
	RC29929G01000	6	272	
	RC30026G00790	3	168	
	RC30068G00180	4	261	
	RC30128G02410	5	288	
	RC30147G03480	5	285	
	RC30147G03490	5	297	
	RC30147G03500	5	277	
	RC30147G03510	5	279	

	RC30147G06430	3	215
	RC30169G03000	5	251
S. bicolor	SB01G035490	4	313
	SB01G035840	4	329
	SB02G029430	4	273
	SB03G001520	5	259
	SB03G003470	3	213
	SB03G003480	3	242
	SB03G012930	3	166
	SB03G027260	5	244
	SB03G032190	5	231
	SB04G012910	4	250
	SB04G012920	4	252
	SB04G015420	4	250
	SB04G021000	3	336
	SB05G018110	5	291
	SB07G026040	3	309
	SB08G013620	4	304
	SB08G013840	4	302
	SB08G014040	4	302
	SB09G006950	3	171
	SB09G020860	5	256
	SB09G030270	2	239
S. moellendorffii	SM00001G06310	4	211
	SM00002G05400	4	263
	SM00006G05800	3	190
	SM00013G00320	3	186
	SM00013G00420	4	202
	SM00013G02570	4	244
	SM00018G01770	4	202
	SM00018G01830	5	246
	SM00019G01560	8	331
	SM00024G01450	3	190
	SM00044G00730	3	196
	SM00061G02030	8	362
	SM00100G00370	4	206
	SM00100G00700	3	184
	SM00716G00010	4	198
T. cacao	TC00G017860	4	180
	TC00G084710	5	235
	TC01G029640	5	274
	TC02G018550	5	287
	TC03G000950	5	227

	TC03G015440	5	280	
	TC03G015450	5	288	
	TC03G015460	5	301	
	TC03G017510	6	263	
	TC03G029390	4	255	
	TC04G000780	5	264	
	TC04G000790	4	135	Outlier
	TC04G028350	5	239	
	TC05G030360	5	239	
	TC05G030370	5	302	
	TC05G030380	3	229	
	TC05G030400	3	130	Outlier
	TC05G030410	3	208	
	TC05G030420	2	74	Outlier
	TC05G030430	5	293	
	TC06G013480	5	233	
	TC08G012800	5	252	
Volvox carteri	VC00001G04430	6	250	
	VC00009G02590	4	274	Outlier
	VC00025G01390	5	315	
V. vinifera	VV01G00850	5	289	
	VV02G05170	4	260	
	VV04G04390	5	278	
	VV05G12700	5	233	
	VV07G03830	6	341	
	VV10G03020	5	232	
	VV14G09050	5	298	
	VV14G09070	13	655	
	VV14G11780	5	254	
	VV16G06810	5	249	
	VV17G01950	5	235	
	VV17G01960	3	127	Outlier
	VV17G01970	5	234	
	VV17G08600	5	270	
	VV17G08610	5	288	
	VV18G11480	5	248	
	VV19G00960	5	235	
Z. mays	ZM01G14960	5	317	
	ZM01G15310	4	306	
	ZM01G34060	3	310	
	ZM02G42570	5	344	
	ZM03G00980	6	327	
	ZM03G11130	5	238	

ZM03G15090	3	160	
ZM03G25090	3	155	
ZM03G39390	5	244	
ZM04G05340	5	293	
ZM04G15200	3	304	
ZM05G23980	4	252	
ZM05G24010	4	261	
ZM05G24020	3	320	
ZM05G29040	3	333	
ZM06G24600	5	250	
ZM07G18560	3	266	
ZM08G07000	2	106	Outlier
ZM08G09090	5	243	
ZM08G16930	5	238	
ZM08G23360	5	243	
ZM09G21220	5	305	
ZM10G03700	4	301	
ZM10G03740	4	302	
