# Supplementary material for: Soybean (Glycine max) SWEET gene family: insights through comparative genomics, transcriptome profiling and whole genome re-sequence analysis
Source: BMC Genomics. 2015 Jul 11;16(1):520. doi: 10.1186/s12864-015-1730-y (PMC4499210; doi:10.1186/s12864-015-1730-y)
Supplement: Additional file 2: — Amino acid sequences of 173 SWEETs from 13 species. [file 12864_2015_1730_MOESM2_ESM.pdf]

Additional file 2: Amino acid sequence of 173 SWEETs from 13 species

>GmSWEET1
MVSISDHELVLIFGLLGNIVSFMVFLAPLPTFYTIYKKKSSEGFQSIPYAVALLSALLLLYYGFIK
TNATLIITINCIGCVIEVSYLTMYIIYAPRKQKISTLVMILIADIGGFGLTMLITTFAVKGINRVHA
VGWICAIFNIAVFAAPLSIMRRVIKTKSVEFMPFSLSLFLTLCATMWFFYGFFDKDDFIMFPNVL
GFIFGISQMILYMIYKNSKKNGETNCTEQQESEGTVNSKQHSCDGNKLDFPSLVEMKENQLNQ
V
>GmSWEET2
METHHDVFVLVFGVLGNIVNSLIYLAPMVTIYDTFQEQTKQHYNAIPYSLSLFTASLMLYYAH
LKGNEEALLLITINSIGCTMEVAYLIICYIYANFRAKTVIVRWVFLFNGATYLVILFLTSLVSPLS
NRLKVVGWICATFSVGVYVTSLINPMMRTVVRTKCISMPLLISLTLSSIVWFFYGFFSHDYFIV
MPNVLHFWLGVAQMILCFIYRNGGADERERVQSETGEINNENDEILETIEMQIAEAKNTNVDH
QTDNDDIKKKENGSSSRVELEPCEIVHLQNNIVVVTRNGTRIVCNEIQSVAEPEIVEIPG
>GmSWEET3
MASLTFAVGIIGNKTFCRVVKKKSTENYKGAPYITTFLCTSLWTSYGVLKPGGFQIAIVNGAGA
VFRCMYIILFLVYSPQDQKVKTALLVAILDVGFLGTVISVTLFRSPWSDPAYKMVIQTKSVEYM
PFLLSFFMFLNWRLDIVFFPRQRFLHW
>GmSWEET4
MAISHETWAFIFGLLGNVISFMVFLAPLPTFYQIYKKKSSEGFQSLPYVVALFSSMLWIYYALV
KKDASLLLITINSFGCVIETIYLAIFLVYAPSKTRLWTIKLLLMLNVFGFGGMLLSTLYLTTGSK
RLSVIGWICLVFNISVFAAPLCIMKRVIKTRSVEFMPFSLSLSLTINAVMWFFYGLLLKDYYIAL
PNTLGFLFGIIQMVLYLVYRNAKPQTLEEPTKVQELNGHIIDVVKPNHATKNGHVPVIEIASSV
>GmSWEET5
MAIHHETWAFVFGLLGNVISFMVFLAPLPTFYQIYKKKSSEGFQSLPYVVALFSSMLWIYYAFV
KKDASLLLITINSFGCVIETIYLAIFLVYAPSKTRLWTIKLLLMLNVFGFGAMLLSTLYLTTGSK
RLTVIGWICLVFNISVFAAPLCIMKRVIKTKSVEFMPFSLSFSLTINAVMWFFYGLLLKDYYIAL
PNTLGFLFGIIQMVLYLIYRNAKKDEPMKLEELNSHIINVGKLSRMEPSEPNHATKNGTVTEITI
EDPNGKETEEGNLKNIMNSASNV
>GmSWEET6
MSSHSHLSFAFGVLGNIASFVCFLAPLPTFYRVCKKKSTEGFQSIPYVAALFSAMLWIFYAYVK
TGEMLLITINAFGCVIETIYLAVFITYCPKKARTNRGCMVNFVKKFQRVVIRTKSVEFLPFPLSL
LLLISAIMWLLYGISLKDIYVTLPNVVGLTFGVIQIGLYAMYRNNKPVKDQKLPEHKGDIVDNN
NESVIAPTVNGEKQEQEVKPQGGIIETGEKKEENNKQDQQQPEENKKFDQVVHEQTKLNNKN
TNNINDDDNNKTGERVISCEV
>GmSWEET7
MAETIRLGVAVLGNAASVALYAAPMVTFRRVIRKKSTEEFSCFPYIIGLLNCLLFTWYGLPIVS
YKWENFPLVTVNGVGILLELSYVLIYFWYASAKGKVKVAMTAIPVLLVFSIIAAVSAFAFHDN
HHRKLLVGSIGLGVSVAMYGSPLIVMKKVIQTKSVEFMPLPLSMCSF
>GmSWEET8
MDVAHFLFGIFGNASALFLFLAPVITFKRIIKNRSTEKFSGIPYVMTLLNCLLSAWYGLPFVSPH
NILVSTVNGTGSLIEIIYVLIFIVLAPRKEKAKILGLFTFVLSVFSAVVFVSLFALHGNSRKLFCGF
AAAIFSIIMYGSPLSIMRLVIKTKSVEFMPFFLSLFVFLCGTSWFIFGLLGRDPFVAVPNGVGSAL
GTTQLILYFIYRDNKGVTGKQAPTEEESMEIGDVKPQQGKQSNANGIQG
>GmSWEET9
MVDTGAIRTVIGVIGNVISFCLFMSPVPTFISIWKSKSVQNFKPDPYIATILNCGMWSIYGMPFV
TEDNTLVVTINGFGFFLEIFYALIFFVYSTWSKRRKIILIFLGELVFLAVVIFLIMTFLHSAKQRKV
IVGPICIVFNILMYFAPLTVMRQVIRTKSVKYMPFLLSFANFANGVIWTTYALLKWDPFIVIPNG
IGTVSGLVQLILYAMYYRTTKWDEEIDSV

>GmSWEET10
MAIFNGHNHLALGFGMLGNVISFMVYLAPLPTFYRIYKKKSTEGFQSLPYLVALFSSMLWLYY
ASLKPADATLLLITINSLGCVIEIVYIIMFTIYATKDARNLTVKLFMVMNVGSFALIFLVTYFAMH
GSLRVQVVGWVCVSIAVGVFAAPLSIVAQVIRTKNVEFMPFNLSLFLTISAVMWFFYGLLLKDI
CIAIPNILGFTLGLLQMLLYAIYRNGKTNNKEVVTKEEHALEAMKNVVVVNPLGTCEVYPVIG
KEINNNGQGIEGAEEKEKGVELGKECPV
>GmSWEET11
MTMHRESWAFVFGVMGNIISFGVFLAPLPTFYQIYKKKSTEGFQSLPYVVALFSAMLWIYYAF
VKREAALLLITINTFGIVVESIYLAIFLLYAPRKPRLTTIKLLLLLNVFGFGAMLLSTLYLSKGAK
RLAIIGWICLVFNISVFAAPLFIIRRVIKTRSVEYMPFTLSMFLTINAVMWFFYGLLLRDYYVALP
NTLGFVFGIIQMGMYLMYRNATPVALEEPVKAQELNGHIIDVGKMGTMEPNHAATAGAVGK
V
>GmSWEET12
PTFYRVCKKKTTEGFQSLPYVAALFTSMLWIFYAYIKTGEILLITINAFGCFIETVYLVIYIIYCPK
KARMIFLFNVGVIFLVVLLTHVLAKERTARIELLGWICVVLSTSVFAAPLSIIKVVIRTKSVEFM
PITLSLLLTVSATMWMAYGILLRDIYVTLPNFVGITFGTIQIVLYLIYRKSKPVKDQKLPEHKNH
VVNDENASTAVSELVPKSYSKVLLHEPIESARIVSIFLFVNKKSEKKIGLQLTRWEVVDMDRVH
GNGGPETETIASTL
>GmSWEET13
MDVAHFLFGIFGNASALFLFLAPVYALHSCFLPSLITFKRIIKNRSTEKFSGIPYVMTLLNCLLSA
WYGLPFVSPHNILVSTVNGTGSFIEIIYVLIFIVLAPRKEKAKILGLFTFVLSVFSAVVFVSLFALH
GNSRKLFCGFAAAIFSIIMYGSPLSIMRLVIKTKSVEFMPFFLSLFVFLCGTSWFIFGLLGRDPFV
AVPNGVGSALGTMQLILYFIYRDNKGVPRKQAPTEEESMEMGDAKPQQGKQSNANGIQG
>GmSWEET14
MAETIRLAVAVLGNAASVALYAAPMVTFRRVIRKKSTEEFSCFPYIIGLLNCLLFTWYGLPVVS
YKWENFPLVTVNGVGIVLELSYVLIYFWYASAKGKVKVAMTAIPVLLVFSIIAAVSAFAFHDN
HHRKLLVGSIGLGVSVTMYGSPLIVMKKVIQTKSVEFMPLPLSMCSFLATVLWLIYGLLIRDIF
VAGPSAVGTPLGILQLVLYCKYRKGSVVEDPSKGDLEKGNLEKVEMEIGKVEMNVTNHMNG
HS
>GmSWEET15
MSHSHLSFAFGILGNIASFVCFLAPLPTFYRVCKKKSTEGFQSIPYVAALFSAMLWIFYAYVKT
GETLLITINAFGCVIETIYLAVFITYCPKKARMSTLRMIVLLNFGGFCTIVLLTHLLAKGEEARV
KLLGWICVVFATSVFAAPLSIIRVVIRTKSVEFLPFPLSLLLLISAIMWLLYGISLKDIYVTLPNVV
GLTFGVIQIGLYAMYRNNKPIKDQKLPEHKGDIVESENVIAPTGNGEKQEEEVKPQGGDIEIGE
KKEENNKQDQQQSVENKKLDQVAHDQTELNKNNINKNNNKTEERVSCEV
>GmSWEET16
MAINHETWAFVFGLLGNVISFMVFLAPLPTFYQIYKKKSTEEFQSLPYVVALFSSMLWIYYALV
KKDASLLLITINSFGCVIETIYLAIFLIYAPSKTRLWTIKLLLMLNVFGFGAMLLSTLYLTTGSKR
LTVIGWICLVFNISVFAAPLCIIKRVIKTKSVEFMPFSLSFFLTINAVMWFFYGLLLKDYYVALP
NTLGFLFSIIQMVLYLIYRNAKTPDLPMKLQELNSHTIDVGKLSRMEPSEPNHLTKNGTLTEREI
>GmSWEET17
MAINHETWAFIFGLLGNVISFMVFLAPLPTFYQIYKKKSTDGFQSLPYIVALFSSMLWIYYALV
KKDASLLLITINSFGCVIETIYLAIFLIYAPSKTRLWTIKLLLMLNVFGFGAMLLSTLYLTTGSKR
LSVIGWICLVLNISVFAAPLCIMKRVIKTKSVEFMPFSLSFFLTINAVMWFFYGLLLKDYYIALP
NTLGFLFGIIQMVLYLIYRNAKPQGLEEPTKVQELNGHIIDVVKPNHVTKNGPVPVIETASNV
>GmSWEET18
MADASFFVGVIGNIISILMFLSPVIDYSWKNMIYPCLQIHVLSLVRPHQKLPHLFYHLEAPAHHK
IKKHGSTEDFSSLPYICTLLNCSLWTYYGIIKAREYLVATADGFGIVVETIYVILFLIYAPKGIRQ

RKSICRTVILVVILDVAISTIAVVTTQLALQREARGGVVGVMGAGLNIVMYFSPLSCHGKYIIYL
LSDKKLMCNLVCVSFRLVCVDSVYGLVLIRFSFLYSLCHLLKHCLYH
>GmSWEET19
PTFWKIKKHGSTEDFSSLPYICTLLNCSLWTYYGIIKAREYLVATVDGFGIVVETIYVILFLIYAP
KGIRGRTVILAVILDVAISTVAVVTTQLALQREARGTQLALQREAHGGGVGVMGACLNIVMY
FSPLSAMCLCRKLSFKKQGIFSRTLQTIIAFRLVCADSVYGLVLIRFSFLYSLCHLLKHCLYH
>GmSWEET20
MAHANPMIFVVGILGNLVSFCCFLAPVPTFYRVCKKKTTEGFQSLPYVAALFTSMLWIFYAYI
KTGEILLITINAFGCFIETVYLVIYITYCPKKARFFTFKMIFLFNVGVIFLVVLLTHVLAKERTARI
ELLGWICVVLSTSVFAAPLSIIKVVIRTKSVEFMPITLSLLLTVSAMMWMAYGILLRDIYVTLPN
FVGITFGTIQIVLYLIYRKNKPVKDQKLPEHKDDVANDENVNTAVSGENRGANATGFVDIEIGE
KKQVQEQADKKQDQQAVNARDQTEHNNNSNKTREG
>GmSWEET21
MTMHRESWAFVFGVMGNIISFGVFLAPLPTFYQIYKKKSTEGFQSLPYVVALFSAMLWIYYAF
VKRETALLLITINTFGIVVESIYLSIFLIYAPRKPRLTTIKLLLLLNVFGFGAMLLSTLYLSKGAKR
LAIIGWICLVFNISVFAAPLFIIRRVIKTRSVEYMPFTLSMFLTINAVMWFFYGLLLRDYYVALP
NTLGFVFGIIQMVMYLMYRNATPVALEEPVKAQELNGHIIDVVKIGTMEPNHGGAVGKV
>GmSWEET22
MALNSHNHLALAFGMLGNVISFMVYLAPLPTFYRIYKKKSTEGFQSLPYLVALFSSMLWLYY
ASLKPADATLLLITINSLGCVIEIVYIVMFTIYATKDARNLTVKLFMVMNVGSFALIFLVTYFAIH
GSLRVQVVGWVCVSIAVGVFAAPLSIVAQVIRTKNVEFMPFNLSLFLTLSAVMWFFYGLLLKD
ICIAIPNILGFTLGLLQMLLYAIYRNGKTNNKEVATKEEKALEAIMKNVVVVNPLGTCEVYPVI
NKENNNGQGIEGAKEKECPV
>GmSWEET23
NVISFMVYLAPLPTFYRIYKKKSTEGFQSLPYLVALFSSMLWLYYASLKPADATLLLITINSLGCV
IEIVYIVMFTIYATKDARNLTVKLFMVMNVGSFALIFLVTYFAIHGSLRVQVVGWVCVSIAVG
VFAAPLSIVAQVIRTKNVEFMPFNLSLFLTLSAVMWFFYGLLLKDICIAIPNILGFTLGLLQMLL
YAIYRNGKTNNKEVATKEEKALEAIMKNVVVVNPLGTCEVYPVINKENNNGQGIEVTSNCTM
ARSF
>GmSWEET24
MGSHNALAATFGILGNIISVMVYLAPVPTFYRIYKKKCTDGFHSLPYLLSLMSSMLWLYYAFL
KIHDGVVPLITINSIGCVIELIYILTYIKYAHKDARNLTYTLFAAMNIGFLALVLSSRFALNGSHR
VKVIGWICDAVSLSVFASPLSIMAKVIRTKSVQFMPFYLSFFLTLNAITWFVYGLSMQDKCIYIP
NVGGFALGLVQMVLYGIYRKGSESEKEQGLGEGVINIVVVNPLGPAEVFPIAVDDNKVKDLV
VVDDVTVSQQEKEKNVEAKDDCPV
>GmSWEET25
MVITHHTLAFTFGMLGNVISFLVFLAPVPTFYRIYKKKSTESFQSLPYLVALFSSMLWLYYALL
KRDAVLLITINSFGCVIEIIYIVLYITYATRDARNLTIKLFSAMNMTSFAVILLVTHFGVHGPLRV
QVLGWICVSISVSVFAAPLSIVAQVVRTKSVEFMPFNLSFTLTLSAIMWFGYGLFLKDICIALPN
VLGFVLGLLQMLLYTIYRKGNKKTNTNEKSLSVKPLKNIAVVNPLGTGEVFPVEEDEQAAKKS
QGDGEDKKAEDCLV
>GmSWEET26
MAISHSTLAFAFGMLGNVISFLVFLAPITTFYRIFKKKSTEGFQSLPYLVALFSSMLWLYYALLK
KDAMLLLTINSFGCVIEIIYIILYITYATGDARNLTLKLFFAMNVGAFALILLVTHFAVHGSLRV
QVLGWICVSLSISVFAAPLSIVAQVVRTKSVEFMPFNLSFTLTLSAIMWFGYGLFLKDICIALPN
VLGFALGLLQMLLYAIYRNGNKKVDKILEKKAPLEPLKSVVIETGEVFLVEEKQQGKKSKESS
EEKEKSDEPNNDCAV
>GmSWEET27

QSIPYVVALLSALLLLYYGFIKTNATLIITINCIGCVIEVSYLSMCIIYAPRKQKISTLVMILIADIG
GLALTMLIIITFAVKAINRVHAVGWICAISSIAVFAAPLSKMRRVIKTSSVEFMPFSLSLFLTLCPI
MWFFYGFFDKDDFIMIPNVLGFLFGISQMILYMIYKNAKKNGETNCTEPARKGGHSELQTAQL
QWQQIRFPISGGNERESTQSSLMLC
>GmSWEET28
MAEASFFVGVIGNIISILMFLSPVPTFWKIKKHGSTEDFSSLPYICTLLNCSLWTYYGIIKAGEYL
VATVNGFGILMETIYIILFLIYAPKGIRGRTAILALILDVVILTAIIIITQLALEGETRSGAVGVMGA
GLNIVMYSSPLSVMKTVVTTKSVEYMPFLLSFFFFFNGAVWLLYAVLVRDVILGVPNGTGFLL
GAMQLVLYAIYRNGKRVSNNRLEEGLQHEPLISEPNNESHQSEDRPI
>GmSWEET29
MSLFAAFSICKVAKDAAGVAGNVFAFGLFVSPIPTFRRIIRNGSTEMFSGLPYIYSLLNCLICMW
YGTPLISADNLLVTTVNSIGAVFQFVYTIIFLMYAEKAKKVRMVGLLLAVLGMFAIVLVGSLQI
DDVIMRRFFVGFLSCASLISMFASPLFIIKLVIQTKSVEFMPFYLSLSTFLMSTSFLLYGLFNDDA
FIYVPNGIGTILGMIQLILYFYFESKSRESSREPLIVSYA
>GmSWEET30
MAETLRMVVAVIGNVASVSLYAAPTVTFKRVIRKKSTEEFSCIPYIIALLNCLLFTWYGLPVVS
NKWENFPLVTVNGVGILFELSYVLIYFWFSTPKGKVKVAMTAVPVLIVFCVIAVVSAFVFPDH
RHRKLLVGSIGLGVSIAMYASPLVVMKKVIQTKSVEFMPLPLSFCSFLASVLWLTYGLLIRDIF
VAGPSVIGTPLGILQLVLHCKYWKRRVTEEPTKVELQKGNNAEKLDLENGHGKECVTVPSNF
NS
>GmSWEET31
MDVAHFIFGIFGNASGLFLFLAPIVTFWRIVSNKSTEKFSGVPYPMTLLNCLLSAWYGLPFVSPN
NLLVTIINGTGAGIEIIYVFIFIYFAPKKEKTKIIGLFSFVVAVFSVVVLVSLFALQGNARKLFCGF
AAAIFSIVMYGSPLSIMRLVIKTKSVEFMPFFLSLFVFLCGTSWFIYGLLGRDPFVAVPNGVGSA
LGTAQLILYFIYRDNKSDPKKIPRTEEEAMEMGTANKNPISNSNGIQEGRV
>GmSWEET32
MVTADIARTVVGIIGNIISGCLFLSPVPTFVRIWKKGSVEQYSAVPYLATLMNCMVWTLYGLP
MVHPHSLLVVTINGAGCVIEIIYVTLFLLYSDRTKRLRVFLCLFSELIFITLLTLLTFTLIHSIKHRS
AIVGTICMLFNIAMYASPLSVMKLVITTKSVEYMPFFLSLASFGNGVSWTTYALIPFDPFIAIPN
GIGTTFSVAQLILYATYYKSTKKQIAAARNAKEVNLSEVVVGNSTVQDPNNNKISAAPYGL
>GmSWEET33
MVSAAIARNVVGVIGNIISFGLFFSPAPTFYGIVKKKTVEEFKPDPYIATVLNCAFWVFYGMPF
VHPNSILVVTINSVGLAFEFVYLTIYYVYATSKGRKKLLIFLLIEAVFFAAVVLITMLALHGTRQ
RSLVVGVLSDIFNVMMYVSPLTIMAKVIKTKSVKYMPFWLSLANFLNGVSWTTYALIHPFDLY
VLISNGIGAISGLIQLILYACYCSCKSENDEGGDQDLKPSGVQLSNLNGRAAV
>GmSWEET34
MALFAAFSICKVAKDAAGVAGNVFAFGLFVSPIPTFRRIIRNGSTEMFSGLPYIYSLLNCLICMW
YGTPLISADNLLVTTVNSIGAVFQFVYIIIFLMYAEKAKKVRMIGLSLAVLGIFAIILVGSLQIDDI
IMRRFFVGFLSCASLISMFASPLFIIKLVIQTKSVEFMPFYLSLSTFLMSTSFLLYGLFNDDAFIYV
PNGIGTILGLIQLILYFYFEGKSRVNSREPLIVSYA
>GmSWEET35
MVSFSDHELVLIFGLLGNIVSFMVFLAPLSNFYTIYKKKSSEGFQSIPYVVALLSALLLLYYDFIK
TKATLIITINCIGCVIEVLYLTMYIIYAPRKQKAINRVHAVGWSCAIFNIAVFVAPLSIMLHSIFNY
SLFMPFSLSLFLTLCAIMWFLYGFFDKDDFIMGAANTAMLSDANEVKN
>GmSWEET36
MDVAHFIFGIFGNVSGLFLFLAPIVTFWRIIKNKSTEKFSGVPYPMTLLNCLLSAWYGLPFVSPN
NILVTIINGTGAGIEIIYVFIFIYFAPKKEKAKILGLFSFVVAVFSVVVLVSLFALHGNARKLFCGF
AAAIFSIIMYGSPLSIMRLVIKTKSVEFMPFFLSLFVFLCGTSWFIYGLLGRDPFVAVPNGVGSAL
GTAQLILYFIYRDKKGDQKKKPRTEEEEAMEMGTANKNPISNSKGAQEGHV

>GmSWEET37
MAISLRMVVAVLGNVASMSLYAAPSVTFKRVIRKKSTEEFSSIPYIIALLNSLLYTWYGLPIISN
KWENFPLVTVNGAGIPFELSYVLIYFWFSSPKGKAIVMCSVSHASHHYMVAITTVTILAVFCFI
AFVSAFAIPGHRYRKLLVGSIGLAVSIALYASPLVAMKKVIQTKSVEFMPLPLSLSSLLASLLW
MTYGLLIGDIFVAGPNVVGTPLGILQIVLYCKYWKKIVTEEPNKVELQKGNTEK
>GmSWEET38
MAETLRMVVAVIGNVASVSLYAAPTVTFKRVIRKKSTEEFSCMPYIIALLNCLLFTWYGLPVV
SNKWENLPLVTVNGVGILFELSYVLIYIWFSTPKGKVKVAMTAVPVLIVFCVIAIVSAFVFPDH
RHRKLLVGSIGLGVSIAMYGSPLVVMKKVIQTKSVEFMPLPLSFCSFLASVLWLTYGLLIRDIF
VAGPSLIGTPLGILQLVLHCKYWKRRVMEEPNKVELQKGNNTEKLDLEMGHGKECVTVPSNC
N
>GmSWEET39
MPTHHASAAIFGIIGNMISVMVYLAPVPTFYQIYKKKCTDGFHSLPYLLSLMSSMLWLYYAFL
KIHDGVVPLITINSIGCVIELIYILTYIKYAHKDARNLTYTLFAAMNIAFLTLVLSSHFALHGSHR
VKVIGWICDAVSLSVFASPLSIMAKVIRTKSVQFMPFYLSFFLTLNAITWFVYGLSIQDKCIYVP
NVGGFGLGLVQMVLYGIYRNGGESEKEQALAEGAINIVVVNPLGPAEVFNRRGSG
>GmSWEET40
MADASFFVGVIGNIISILMFLSPVPTFWKIKKQGSTEDFSSLPYICTLLNCSLWTYYGIINAREYL
VATVNGFGIVVETIYVILFLIYAPKGRRGRTAILAVILDVAILAAAVVITQLAFQGKARSGAVGV
MGAGLNIVMYFSPLSAMKTVVKTKSVEYMPFLLSFFFFLNGGVWLLYAVLVRDVILGVPNGT
GFLLGAMQLVLYAIYRNGKPSSNNRLEEGLQHEPLISQPNKESHQIREDRPI
>GmSWEET41
MEHLCFWVVCVLKSFGSNSKITLQSHLKSNFFFFFYIQMISANNLIPKTTEIWHLSMSPIPIFRRII
KNGSTKMFSGLPYIYSLLNCLICLWYGTPLISPDNLLVTTVNSIGAAFQLVYILFLMYAEKARK
VRMVGLLLTVLGIFVIILVGSLQVDDSTMRGMFVRFLSCASLISTFASPLFIIKLVIQTKSVEFMP
FYLSISTFLMSISFFLYGFLSDDAFIYVPNGIGTVLGMIQLVLYFYYKGSTSEECREPLIVSYE
>GmSWEET42
MSLFGAYSICEVGKDAAGVAGNIFAFGLFVSPIPTFRRIIRNGSTEMFSGLPYIYSLLNCMICLW
YGTPLISPDNLLVTTVNSIGAAFQLVYIILFLMYAEKARKVRMVGLLLAVLGIFVIILVGSLQID
DSAMRRMFVGFLSCASLISMFASPLFIIKLVIRTKSIEFMPFYLSLSTFLMSFSFFLYGLLSDDAFI
YVPNGIGTVLGIIQLVLYFYYKGSSSEECREPLIVSYE
>GmSWEET43
MVDPGTIRTVVGVIGNVISFCLFMSPIPTFISIWKSKSVQNFKPDPYIATILNCAMWSFYGMPFV
TEDNTLVVTINGFGFFLEMFYTLIFFIYSTWSKRRKILLIFLGEIVFLALVVILLMTFLHSAKQRK
VIVGPICIVFNILMYFAPLTVMRRVIQTKSVKYMPFLLSFANFANGIIWTTYALLKWDPFIVIPNS
IGAVSGLTQLVLYAMYYKTTNWDEEIEQV
>GmSWEET44
MVLFSDHELVLIFGLLGNIVSFMVFLAPWPTFYTIYKNKSSEGFQSIPYVVALLSALLLLYYGFI
KTNATLIITINCIGCVIEVSYLAMYIIYAPRKQKISTLVMILIADIGGFGLTMLITTFAVKGINRVH
AVGWICAIFNIAVFAAPLSIMRRVIKTKSVEFMPFSLSLFLTLCATMWFFYGFFDKDNFIMLPNV
LGFLFGISQMILYMIYKNAKKNGEINCTEQQERDGTVNSKQHSCNGNKLDFSSLVEMKENQLN
QV
>GmSWEET45
MAISHSTLAFAFGMLGNVISFLVFLAPMTTFYRIFKKKSTEGFQSLPYLVALFSSMLWLYYALL
KKDAMLLLTINSFGCVIEVIYIILYITYATRDARDLTLKLFFAMNVGAFALILLVTHFAVHGSLR
VQVLGWICVSLSISVFAAPLSIVAQVVRTKSVEFMPFNLSFTLTLSAIMWFGYGLFLKDICIAVP
NVLGFALGLLQMLLYAIYRNGNKKVDKIMEKKAPLEPLKTVVIETGLEEKQQGKKSKENSEE
KEKSDEPNDCAV
>GmSWEET46

MVISHHTLAFTFGMLGNLISFLVFLAPVPTFYRIYKKKSTESFQSLPYLVALFSSMLWLYYAML
KRDAVLLITINSFGCVIEIIYIVLYITYATRDARDLTIKLFSAMNMSSFALILLVTHFAVHGPLRV
QVLGWICVSISVSVFAAPLSIVAQVVRTKSVEFMPFNLSFTLTLSAIMWFGYGLFLKDICIAVPN
VLGFVLGLLQMLLYTIYRKGNKKTKTNEKSPVEPLKSIAVVNPLGTGEVFPVEEDEQAAKKSQ
GDGDDKKGQDCLV

>GmSWEET47

MVSAAIARNVVGIIGNVISFGLFLSPAPTFYKIIKNKAVEEFKPDPYIATVLNCAFWVFYGMPFI
HPHSILVVTINGIGLVFEFVYLTIFFTYATNKGRKKLLICLLIEAIFFAAIVLITMLAVHGKHRSL
MIGVICDFFNIMMYVSPLTIMFKVIKTKSVKYMPFWLSLTNFLNGACWTTYALIHPFDLFVLIS
NSVGVVSGFVQLILYACYCCRENNDDGDDDGAVQKKTTGVPVSESPRDVEA

>GmSWEET48

MVSAAIARNVVGVIGNIISFGLFFSPAPTFYGIVKKKTVEEFKPDPYIATVLNCAFWVFYGMPF
VHPNSILVVTINSVGLAFEFVYLTIYYVYATNKGRKKLLIFLLIEVVFFAAVALITMLALHGTRQ
RSLVVGVLSDIFNVMMYVSPLTIMAKVIKTKSVKYMPFWLSLANFLNGACWTTYALIHPFDL
YVLISNGIGAISGLIQLILYACYCSCKSKNDEDGDQDLKPSGFQLSNLNGRAAVV

>GmSWEET49

MSNSSMASLTFAVGIIGTVLSLLVFASPIKTFCRVVKKKSTENYKGAPYITTFLCTSLWTSYGVL
KPGGFQIAIVNGAGAVFHCTYIILFLVYSPQDQKVKTALWVAILDVGFLGTVISVTLFALHGTIQ
LSVLGMFCSGLTIIMYASPLLSMKMVIQTKSVEYMPFLLSFFMFLNAGVWALYSFLVKDFFIGI
PNLIGLILGSTQLTVYVVVYKKKQPEATKGPRVGLSLGKGASNYEEAQLKDETVKVVVVEKAL
KKVKSLPKPVLNHEHILKKTLSFGVNNLPSTFWSTKPQQEDVAVDAEEAQV

>GmSWEET50

MRPTFWKIKKHESTEDFSSLPYICTLLNCSLWTYYGIIKAREYLVATVNGFGIVVETIYVILFLIY
APKGIRDCHFGCDFGCRNFGRSSNYFSISIARRSSWWCCWCYGSRLKHCHVFLTSLCHDIIWK
DRTRKYRLIIFVEGDYQFRVVACVEMLEPS

>GmSWEET51

MVTADIARTVVGIIGNIISGCLFLSPVPTFVRIWKKGSVEQYSAVPYLATLMNCMVWTLYGLP
MVHPHSLLVVTINGAGCVIEIIYVTLFLLYSDRTKRLKVFLWLFLELVFIAVLTFVTFTLIHSVK
KRSAVVGTICMLFNVAMYASPLSVMKLVITTKSVEYMPFFLSLASFGNGVSWTTYALIPFDPFI
AIPNGIGTTFSVAQLILYATYYKSTKKQIAARNAKEVNLSEVVVGNSTVQDPNNNKISAAPNGL

>GmSWEET52

FYLDNLGNIEHDLSLSSNTCSISGSTPLETSTLILSFGSASTSCRPMFWKIKKHGSTEDFSSLPYIC
TLLNCSLWTYYGIIKAREYLVATVDGFGIVVETIYVILFLIYAPKGIRGRTLILAVILDVAISAVA
VVTTQLALQREAHGGVVGVMGAGLNIVMYFSPLSAMLKSKDSHCTLQTIIAFRFVCVDSVYG
LILIRFSFLYSLCHLLKH

>AtSWEET1

MNIAHTIFGVFGNATALFLFLAPSITFKRIIKNKSTEQFSGIPYPMTLLNCLLSAWYGLPFVSKDN
TLVSTINGTGAVIETVYVLIFLFYAPKKEKIKIFGIFSCVLAVFATVALVSLFALQGNGRKLFCGL
AATVFSIIMYASPLSIMRLVVKTKSVEFMPFFLSLFVFLCGTSWFVYGLIGRDPFVAIPNGFGCA
LGTLQLILYFIYCGNKGEKSADAQKDEKSVEMKDDEKKQNVVNGKQDLQV

>AtSWEET6

MVHEQLNLIRKIVGILGNFISLCLFLSPTPTFIHIVKKKSVEKYSPLPYLATLLNCLVRALYGLPM
VHPDSTLLVTISGIGITIEIVFLTIFFVFCGRQQHRLVISAVLTVQVVFVATLAVLVLTLEHTTDQ
RTISVGIVSCVFNAMMYASPLSVMKMVIKTKSLEFMPFLLSVVGFLNAGVWTIYGFVPFDPFL
AIPNGIGCVFGLVQLILYGTYYKSTKGIMEERKNRLGYVGEVGLSNAIAQTEPENIPYLNKRVS
GV

>AtSWEET9

MFLKVHEIAFLFGLLGNIVSFGVFLSPVPTFYGIYKKKSSKGFQSIPYICALASATLLLYYGIMKT
HAYLIISINTFGCFIEISYLFLYILYAPREAKISTLKLIVICNIGGLGLLILLVNLLVPKQHRVSTVG

WVCAAYSLAVFASPLSVMRKVIKTKSVEYMPFLLSLSLTLNAVMWFFYGLLIKDKFIAMPNIL
GFLFGVAQMILYMMYQGSTKTDLPTENQLANKTDVNEVPIVAVELPDVGSDNVEGSVRPMK
>AtSWEET2
MDVFAFNASLSMCKDVAGIAGNIFAFGLFVSPMPTFRRIMRNKSTEQFSGLPYIYALLNCLICL
WYGTPFISHSNAMLMTVNSVGATFQLCYIILFIMHTDKKNKMKMLGLLFVVFAVVGVIVAGS
LQIPDQLTRWYFVGFLSCGSLVSMFASPLFVINLVIRTKSVEFMPFYLSLSTFLMSASFLLYGLF
NSDAFVYTPNGIGTILGIVQLALYCYYHRNSIEEETKEPLIVSYV
>AtSWEET16
MADLSFYVGVIGNVISVLVFLSPVETFWRIVQRRSTEEYECFPYICTLMSSSLWTYYGIVTPGEY
LVSTVNGFGALAESIYVLIFLFFVPKSRFLKTVVVVLALNVCFPVIAIAGTRTLFGDANSRSSSM
GFICATLNIIMYGSPLSAIKTVVTTRSVQFMPFWLSFFLFLNGAIWGVYALLLHDMFLLVPNGM
GFFLGIMQLLIYAYYRNAEPIVEDEEGLIPNQPLLA
>AtSWEET4
MVNATVARNIAGICGNVISLFLFLSPIPTFITIYKKKKVEEYKADPYLATVLNCALWVFYGLPM
VQPDSLLVITINGTGLAIELVYLAIFFFFSPTSRKVKVGLWLIGEMVFVGIVATCTLLLFHTHNQ
RSSFVGIFCVIFVSLMYIAPLTIMSKVIKTKSVKYMPFSLSLANFLNGVVWVIYALIKFDLFILIG
NGLGTVSGAVQLILYACYYKTTPKDDEDEEDEENLSKVNSQLQLSGNSGQAKRVSA
>AtSWEET11
MSLFNTENTWAFVFGLLGNLISFAVFLSPVPTFYRIWKKKTTEGFQSIPYVVALFSATLWLYYA
TQKKDVFLLVTINAFGCFIETIYISMFLAYAPKPARMLTVKMLLLMNFGGFCAILLLCQFLVKG
ATRAKIIGGICVGFSVCVFAAPLSIIRTVIKTRSVEYMPFSLSLTLTISAVIWLLYGLALKDIYVAF
PNVLGFALGALQMILYVVYKYCKTSPHLGEKEVEAAKLPEVSLDMLKLGTVSSPEPISVVRQA
NKCTCGNDRRAEIEDGQTPKHGKQSSSAAAT
>AtSWEET7
MVFAHLNLLRKIVGIIGNFIALCLFLSPTPTFVRIVKKKSVEEYSPIPYLATLINCLVWVLYGLPT
VHPDSTLVITINGTGILIEIVFLTIFFVYCGRQKQRLIISAVIAAETAFIAILAVLVLTLQHTTEKRT
MSVGIVCCVFNVMMYASPLSVMKMVIKTKSVEFMPFWLSVAGFLNAGVWTIYALMPFDPFM
AIPNGIGCLFGLAQLILYGAYYKSTKRIMAERENQPGYVGLSSAIARTGSEKTANTNQEPNNV
>AtSWEET17
MAEASFYIGVIGNVISVLVFLSPVETFWKIVKRRSTEEYKSLPYICTLLGSSLWTYYGIVTPGEY
LVSTVNGFGALVETIYVSLFLFYAPRHLKLKTVDVEAMLNVFFPIAAIVATRSAFEDEKMRSQS
IGFISAGLNIIMYGSPLSAMKTVVTTKSVKYMPFWLSFFLFLNGAIWAVYALLQHDVFLLVPNG
VGFVFGTMQLILYGIYRNAKPVGLSNGLSEIAQDEEEGLTSRVEPLLS
>AtSWEET14
MVLTHNVLAVTFGVLGNIISFIVFLAPVPTFVRICKKKSIEGFESLPYVSALFSAMLWIYYALQK
DGAGFLLITINAVGCFIETIYIILFITYANKKARISTLKVLGLLNFLGFAAIILVCELLTKGSNREK
VLGGICVGFSVCVFAAPLSIMRVVIRTKSVEFMPFSLSLFLTISAITWLFYGLAIKDFYVALPNIL
GAFLGAVQMILYVIFKYYKTPLVVDETEKPKTVSDHSINMVKLSSTPASGDLTVQPQTNPDVS
HPIKTHGGDLEDQMDKKMPN
>AtSWEET15
MGVMINHHFLAFIFGILGNVISFLVFLAPVPTFYRIYKRKSTESFQSLPYQVSLFSCMLWLYYAL
IKKDAFLLITINSFGCVVETLYIAMFFAYATREKRISAMKLFIAMNVAFFSLILMVTHFVVKTPP
LQVSVLGWICVAISVSVFAAPLMIVARVIKTKSVEYMPFTLSFFLTISAVMWFAYGLFLNDICIA
IPNVVGFVLGLLQMVLYLVYRNSNEKPEKINSSEQQLKSIVVMSPLGVSEVHPVVTESVDPLSE
AVHHEDLSKVTKVEEPSIENGKCYVEATRPETV
>AtSWEET12
MALFDTHNTWAFVFGLLGNLISFAVFLSPVPTFYRICKKKTTEGFQSIPYVVALFSAMLWLYY
ATQKKDVFLLVTINSFGCFIETIYISIFVAFASKKARMLTVKLLLLMNFGGFCLILLLCQFLAKG
TTRAKIIGGICVGFSVCVFAAPLSIIRTVIKTKSVEYMPFSLSLTLTISAVIWLLYGLALKDIYVAF

PNVIGFVLGALQMILYVVYKYCKTPSDLVEKELEAAKLPEVSIDMVKLGTLTSPEPVAITVVRS
VNTCNCNDRNAEIENGQGVRNSAATT

>AtSWEET8

MVDAKQVRFIIGVIGNVISFGLFAAPAKTFWRIFKKKSVEEFSYVPYVATVMNCMLWVFYGLP
VVHKDSILVSTINGVGLVIELFYVGVYLMYCGHKKNHRRNILGFLALEVILVVAIILITLFALKG
DFVKQTFVGVICDVFNIAMYGAPSLAIIKVVKTKSVEYMPFLLSLVCFVNAGIWTTYSLIFKIDY
YVLASNGIGTFLALSQLIVYFMYYKSTPKEKTVKPSEVEISATERV

>AtSWEET10

MAISQAVLATVFGILGNIISFFVCLAPIPTFVRIYKRKSSEGYQSIPYVISLFSAMLWMYYAMIKK
DAMMLITINSFAFVVQIVYISLFFFYAPKKEKTLTVKFVLFVDVLGFGAIFVLTYFIIHANKRVQ
VLGYICMVFALSVFVAPLGIIRKVIKTKSAEFMPFGLSFFLTLSAVMWFFYGLLLKDMNIALPN
VLGFIFGVLQMILFLIYKKPGTKVLEPPGIKLQDISEHVVDVVRLSTMVCNSQMRTLVPQDSAD
MEATIDIDEKIKGDIEKNKDEKEVFLISKN

>AtSWEET13

MALTNNLWAFVFGILGNIISFVVFLAPVPTFVRICKKKSTEGFQSLPYVSALFSAMLWIYYAMQ
KDGTAFLLITINAFGCVIETIYIVLFVSYANKKTRISTLKVLGLLNFLGFAAIVLVCELLTKGSTR
EKVLGGICVGFSVSVFAAPLSIMRVVVRTRSVEFMPFSLSLFLTISAVTWLFYGLAIKDFYVALP
NVLGAFLGAVQMILYIIFKYYKTPVAQKTDKSKDVSDHSIDIAKLTTVIPGAVLDSAVHQPPAL
HNVPETKIQLTEVKSQNMTDPKDQINKDVQKQSQV

>AtSWEET3

MGDKLRLSIGILGNGASLLLYTAPIVTFSRVFKKKSTEEFSCFPYVMTLFNCLIYTWYGLPIVSH
LWENLPLVTINGVGILLESIFIFIYFYYASPKEKIKVGVTFVPVIVGFGLTTAISALVFDDHRHRK
SFVGSVGLVASISMYGSPLVVMKKVIETRSVEYMPFYLSFFSFLASSLWLAYGLLSHDLFLASP
NMVATPLGILQLILYFKYKNKKDLAPTTMVITKRNDHDDKNKATLEFVVDVDRNSDTNEKNS
NNASSI

>AtSWEET5

MTDPHTARTIVGIVGNVISFGLFCAPIPTMVKIWKMKSVSEFKPDPYVATVLNCMMWTFYGLP
FVQPDSLLVITINGTGLFMELVYVTIFFVFATSPVRRKITIAMVIEVIFMAVVIFCTMYFLHTTKQ
RSMLIGILCIVFNVIMYAAPLTVMKLVIKTKSVKYMPFFLSLANFMNGVVWVIYACLKFDPYIL
IPNGLGSLSGIIQLIIYITYYKTTNWNDDDEDKEKRYSNAGIELGQA

>CR06G04860

MGASGNMLLDTVVPGMGAVISILMYLSPLKAVLKAQREKHLGDLNPIPFSITIANCIAWLGYG
LLKKDPFVCAPNAPGVLIGTYMSLTAHGLADEGAKERIRFVVCLAAAIFPFLGVYTSFFAPSAV
VQQGVWGMAGNIVCLVYYAAPLSTMWDVIRTRNSSSILVPLTMMNTLNAALWTTYGVAVA
DPYIWAPNGIGLALSVMQIALRLVFPARAASALPSHAHHSGGSGASKYARLDEEVPLGGAGH

>CR06G05510

MTAWTGRRALLDDDDEFDFKKLFLHHLAPGLGCIIAFLMFVSPLKTVLQIRANKHLGDLNPLP
LVAIIANCAAWLIYGCINADPYVITANEPGLLLGIFMTVSCYGFADPKARDVMLKALMFFAVL
LSAVGIAIALFIEEDETASKTAGYTAVFILLCYYGAPLSTMAEVLRSRSSASLFWPTSLMNTING
LLWVAYGTAVSDPFIAVPNAIGAAFGVIQIGLINIYPAKK

>CR07G05980

MGVFTEHVVPIFGNILACAMLVSPFPAVLRLRAAGKLGDINPLPYPMTVVNAAGWVAYGFAV
ANPYIFPANVVGFLAGVFFTFTAYAAAPKQVQDRITGIMVAASAHYIMLGLIACFALSHTAGA
RMWGTSAVVILMLYYFVPLSTMVQIVKTRNAASIYPPLAITAIANGLMWSIYGFAIMDINLWL
PNLFGSIVGVIQLLLRLVYGAKPTAAAAGGGALAVGAGAVAADDEETAAFAKTGAEPSG

>ZM01G14960

MADPSFLVGIVGNVISILVFASPIATFRRIVRSRSTEDFRWLPYVTTLLSTSLWTFYGLLKPGGLL
VVTVNGAGAALEAAYVALYLVYAPRETKAKMAKVVVAVNVAFLAAVVAVALLALHGGAR
LFAVGLLCAALTVGMYAAPLGAMRTVVKTRSVEYMPFSLSFFLFLNGGVWSIYSLLVKDYFIG

VPNAIGLVLGTAQLLLYLAYRKAPASKDDDEEAAAAASGDDGDDEEEEEGLTHLMGQQVEM
AQRGRLRLHKGQSLPKPPPGGPLSSPRHGFGSIIKSLSATPVELHSVLYQHARFQPVKKDDDDV
EAND
>ZM01G15310
MITVGHPVVFAVGILGNILSFLVTLAPVPTFYRVYKKKSTESFQSVPYVVALLSAMLWLYYAL
LSVDLLLLSINTIACVVESVYLAIYLTYAPKPAMAFTLKLLCTMNMGLFGAMVAFLQFYVDGQ
RRVSIAGGVGSAFAFAVFVAPLTIIRQVIRTKSVEFMPFWLSFFLTVSAVAWFFYGLLMKDFFV
AMPNVLGLLFGLAQMALYFVYRNRNPKKNGAVSEMQQAAAVQADAEKEQQLRQADADAD
ADGKAATTDDDGGQTAVVVDIMPPPPLLPAERAPPLPLPPHPAMVMTTAHQTAVEVV
>ZM01G34060
MAGGFFSMAHPAVTLSGIAGNIISFLVFLAPVATFLQVYRKKSTGGFSSVPYVVALFSSVLWIF
YALVKTNSRPLLTINAFGCGVEAAYIVLYLAYAPRRARLRTLAYFFLLDVAAFALVVAVTLFA
VREPHRVKFLGSVCLAFSMAVFVAPLSIIVKVVKTKSVEFLPISLSFCLTLSAVAWFCYGLFTK
DPFVMYPNVGGFFFSCVQMGLYFWYRKPRPAAKNNAVLPTTTDGANAVQVQGQVIELAPNT
VAILSVSPIPIVGVHKIEVVEQQHKEAAVAAETRRMAAANPDGAMPEVIEIVPAAAAV
>ZM02G42570
MAGLSLQHPMAFAFGLLGNIISFMTYLAPLPTFCRIYRNKSTEGFQSVPYVVALFSAMLWIYYA
LLKSNEFLLITINSAGCVIETLYIATYLLYAPNKAKLFTAKILLLLNVGVFGLILLLTLLLSAGPH
RVVVLGWVCVAFSVSVFVAPLSIIRQVVRTRSVEFMPFSLSFSLTASAVVWFLYGLLIKDKYV
ALPNVLGFTFGVVQMGMYALYRNATPRVPAAKEAAAAADDGNTFNFKAPGEHVVTIAKLTA
AAPATAAELIIKARDDAQHPPEEEAAAAKAAPAKSKLLIPLPEHAYACMCIIRSGSHHKLGRAC
LLGTSTRPPACLPARMIQSSCYIRKG
>ZM03G00980
MVPDTVRVAVGILGNAASMLLYTTPILTFRWVIRKGNVEEFSCVPYILALLNCLLYTWYGLPV
VSSGWENLPVATINGLGILLEVAFIAIYLRFAPAEKKRFALQLVLPALALFGLTAALSSFAARTH
RSRKAFVGSVGLVASVSMYTSPMVAAKRVIATKSVEFMPFSLSLFSFLSSALWMAYGLLGRDL
FIASPNFIGVPVGVLQLLLYCIYRRDHGAAAGAEAQAHGPAAAADQEKGMKAAAPVAVQPQE
NPLCVVSVCEVNVSLSPSAAQAQHRTGLSKSNEIEGLALGLYGHIAATQLLRTTYTDQQIHLW
RVWFMKSLYTS
>ZM03G11130
MDSTLFIIGVIGNIISVLVFISPIKTFWRIVRSGSTEEFEPAPYVFTLLNALLWLYYGATKPDGLLV
ATVNGFGAAMEAIYVVLFIVYAANHATRVKTAKLAAALDIGGFGVVFVATTFAINELNMRIM
VIGMICACLNVLMYGSPLAAMKTVITTKSVEFMPFFLSFFLFLNGGIWATYAVLDRDIFLGIPN
GIGFILGTIQLIIYAIYMNSKVSQSSKEIASPLLASSQEEAASHV
>ZM03G15090
MAGLSLQHPWAFTFGLLGNVISFMTFLAPIPTFYRIYKSKSTEGFQSVPYVVALFSAMLWIFYA
LIKSNETFLITINAAGCVIETVYVVMYFVYATKKGRMFTAKIMLLLNVGAFGAILLLTLLLFKG
DKRVVMLGWICVGFSVSVFVAPLSIMRVYIHV
>ZM03G25090
MEHIARFFFGVSGNVIALFLFLSPVVTFWRVIRKRSTEDFSGVPYNMTLLNCLLSAWYGLPFVS
PNNILVSTINGTGSVIEAIYVVIFLIFAVDRRARLSMLGLLGIVASIFTTVVLVSLLALHGNARKV
FCGLAATIFSICMYASPLSIMVRER
>ZM03G39390
MISPDAARNVVGIIGNVISFGLFLSPVLTFWRIYKAKDVEEFKPDPYLATLLNCMLWVFYGIPV
VHPNSILVVTINGIGLVIEAVYLTIFFLYSDSQKRKKAFAILAVEILFMVAVVLGVILGAHTHEK
RSMIVGILCVIFGSMMYASPLTIMSRVIKTKSVEYMPFLLSLVSFLNGCCWTAYALIRFDLYVTI
PNALGAFFGLVQLILYFCYYKSTPKKEKNVELPTVSSNVGGGNVTVSVER
>ZM04G05340

MAGLSLLHPMAFAFGLLGNIISFMTYLAPLPTFYRIYKNKSTEGFQSVPYVVALFSAMLWIYYA
LLKSNELLLITINSAGCVIETLYIAMYLLYAPKKAKLFTAKILLLLNVGVFGLILLLTLLLSAGQR
RVVVLGWVCVAFSVSVFVAPLSIIRQVVRTRSVEFMPFSLSLSLTVSAVVWFLYGLLIKDKYV
ALPNVIGFSFGVVQMGLYALYRNATPRVPAKDVADDASKDKAPGEHVVVTIAKLTAATTAPA
AAVAEDLVKVHDGHPEEAAKGAAKPAENGAGRSDAEQV

>ZM04G15200

MAFLNMEQQTWAFTFGILGNIVSLMVFLSPLPTFYRVYRNKSTEGFQSTPYVVTLFSCMLWIL
YALLKPGAELLVTINGVGCVVETVYLAMYLVYAPKAARVLAAKMLLGLNVAVFGLVALVT
MLLSDAGLRVHVLGWICVSVSLSVFAAPLSIMRQVIRTKSVEFMPISLSFFLVLSAVVWFAYGA
LKKDVFVAFPNVLGFVFGLAQMALYMAYRKPAAALVIIPEQSKEEVAEGKASCGGAEVHPIDI
AEVHDLQTVVVDVDVEPVTYAAASGMVDGSVGRPRAPEELVIKPDMVTVIAAEA

>ZM05G23980

MISPDTIRTAIGVIGNGTALVLFLSPVPTFIRIWKKGSVEQYSPIPYVATLLNCMMWVLYGLPAV
HPHSMLVITINGTGMAIQLTYVALFLLYSVGAARRKVVLLLAAEVGFVGAVAALVLSLAHTH
ERRSMVVGILCVLFGTGMYAAPLSVMKMVIQTKSVEYMPLFLSLASLVNGICWTAYALIRFDL
YITIPNGLGVLFAVAQLVLYAIYYKSTQEIIEARKRKADQIAMTGVVVDGGKTNNQAGAGQY

>ZM05G24010

MVSADTIRTAIGVIGNGTALVLFLSPVPTFVGIWKKRAVEQYSPIPYVATLLNCMMWVLYGLP
LVHPHSMLVVTINGTGMLIQLTYVALFILCSAGAVRRRVVLLFAAEVAFVVALAALVLTLAHT
HERRSMLVGIVSVFFGTGMYAAPLSVMKLVIQTKSVEYMPLFLSLASLANSICWTAYALIRFD
LYITIPNGLGVLFALGQLGLYAMFYKNTKQIMEARRRKADQQSTMMEVVTDASATPPPPPNN
NNGGGGGNGY

>ZM05G24020

MSRYSSTQSIIYVHVRINFVEAGLLIFLPICCLFIYRTNLPQIKVQLVNFFLFVATKERTGYIYYIL
TFGFLFWYGCLAGNGTALVLFLSPVPTFIRIWKKGSVEQYSPIPYVATLLNCMMWVLYGLPLV
HPHSMLVITINGTGMLIQLTYVALFLVYSAGAARRKVSLLLAAEVAFVGAVAALVLALAHTH
ERRSMVVGILCVLFGTGMYAAPLSVMKMVIQTKSVEYMPLFLSLASLVNGICWTAYALIRFDL
YITIPNGLGVLFALAQLLLYAIYYKNTQKIVEARKRKAGQVAMTEVVVDGSRASNNNNNGGS
GTY

>ZM05G29040

MAFLNMEQQTWAFTFGILGNIISLMVFLSPLPTFYRVYRKKSTEGFQSTPYVVTLFSCMLWIFY
ALLKSGAELLVTINGVGCVIEAAYLAAYLVYAPKAARALTAKMLLGLNVGVFGLAALATMV
VSSAGLRVRVLGWICVSVALSVFAAPLSIMRQVVRTKSVEFMPISLSFFLVLSAVIWFAYGALK
RDVFVAFPNVLGFVFGVAQIALYMAYRNKEPAAVTVEEAKLPEHAKEVVVAAAAAEARASC
GAEVHPIDIDIEATPTPVEEVHEPQVVVVVDVDVEPVTCAGAAEAAAGAGADASGVADGGVP
GPMAPPEQLAIKPDMAISVEA

>ZM06G24600

MEDVVKFVFGVSGNVIALFLFLSPVPTFWRIIRRKSTEDFSGVPYSMTLLNCLLSAWYGLPFVS
PNNMLVSTINGAGAAIEAVYVVIFLAFASSQRTRLRMLGLASAVSAAFAAVALASMLALHGQ
GRKLMCGLAATVCSICMYASPLSIMRLVVKTKSVEYMPFLLSLAVFLCGTSWFVYGLLGRDPF
VAIPNGCGSFLGAVQLVLYAIYRDSNSGGKQQAGDDVEMASDAKSSKKVADDVGGKEDRLV

>ZM07G18560

MGLCFWHSRPTFLRVYRKKSTEGFSSVPYVVALFSCTLWILYALVKTNSSPLLTINAFGCVVEA
AYILLYLVYAPRGARLRALASFLLLDVAAFSLVAVVTVVLVAEPHRVRVLGSVCLAFSMAVF
VAPLSVIFVVIRTKSAEFMPFTLSFFLTLSAVAWFLYGLFTKDPYVTLPNVGGFFFGCIQMVLY
CCYRKRKPASVVVLPTTTAAAAVAQQLEAEMELPLAAHQHQLAVAVLPTCAAPVLAELQKL
EEAMGSPRKGGVKAI

>ZM08G07000

MDSTLFIIGVIGNIISVLVFISPIKTFWRIVRGGTTEEFEPAPYVLTLLNALLWLYYGLTKPDGFL
VATVNGFGAVMEAIYVVLFIVYAANHATRVRGRSSWGQYI

>ZM08G09090

MDWDAPALTSFVADLSFRHLCCYGAGIAGNAFAFVLFVSPLPTFKRIVRNGSTEQFSCTPYIYS
LLNCLICMWYGLPFVSYGVVLVATVNSIGAVFQLAYTAVFIAFADAKQRLKVSALLAAVFLVF
GLIVFVSLALLDHKARQVFVGYLSVASLVCMFASPMSIVNLVIRTKSVEYMPFYLSLSMFLMS
ASFVIYGVLLGDGFIYIPNGIGTILGIVQLLLYAYIRKGSSEEAKLPLLITHT

>ZM08G16930

MVTSIRVIVGIIGSVVCVLLYAVPVLTFKRVVKEASVGEFSCVPYILALFSAFTWGWYGFPIVSD
GWENLSLFGTCAVGVLFEASFVVVYVWFAPRDKKKSVVLMVSLVVATLCVIVSLSSFVFHTH
HMRKQFVGSIGIVTSISMYSAPLVAVKQVILTKSVEFMPFYLSLFSLLTSFTWMLYGILGRDPYL
TAPNGAGCLTGLLQIAVYCIYSRCNRPPKAVNGATTSREDANDCKV

>ZM08G23360

MISPDAARNVVGIIGNVISFGLFLSPVLTFWRICKARDVEEFKPDPYLATLLNCMLWVFYGIPV
VHPNSILVVTINGVGLVIEAIYLTIFFLYSDGPKRRKAFGILAVEILFMVAVVLGVILGAHTHEK
RSMIVGILCVIFGSMMYASPLTIMSRVIKTKSVEYMPFLLSLVSFLNGCCWTAYALIRFDLYVTI
PNALGAFFGLIQLILYFCYYKSTPKEKNVELPTVSSNAGGGNVTVSVER

>ZM09G21220

MITVGHPVAFAVGILGNILSFLVILAPVPTFYRVYAKKSTESFQSVPYVVALLSATLWLYYALL
STDLLLLSINTVACVAESVYLAVYLAYAPGPAKAFTLKLLCAINMGLFGAMVAFLQFYVVDT
QRRVSIAGGVGAAFALAVFVAPLAIIRRVMRTKSVEFMPFWLSFFLTVSAVVWFFYGLLIKDFF
VAMPNVLGLLFGLAQMVLFFVYRNRNPKKNGAVSEMQQAAVQADAEKERRSHANADGEAD
VRTVIVDIMPPPPAMMRHADREARGGAGTGRRAAAREQGGARRREDREALGGGGI

>ZM10G03700

MAGLSLEHPWAFAFGLLGNVISFMTFLAPIPTFYRIYKSKSTEGFQSVPYVVALFSAMLWIFYA
LIKSNETFLITINAAGCVIETIYIVMYFVYAPKKAKLFTAKIMALLNGGVFGVILLLTLLLFKGSK
RVVLLGWICVGFSVSVFVAPLSIMRRVIQTKSVEYMPFSLSLSLTLSAVVWFLYGLLIKDKYVA
LPNVLGFIFGVVQMVLYVFYMNKTPVAAAVGKDAGKLPSAADEHVLVNIAKLNPALPERTSG
MHPVTQMAAVPARSCAAEAIAPAMLPNRDVVDVFVSRHSPAVHVV

>ZM10G03740

MAGMSLQHPWAFAFGLLGNVISFMTFLAPIPTFYRIYKSKSTEGFQSVPYVVALFSAMLWIFY
ALIKSNETFLITINAAGCVIETIYVVMYFVYAPKKAKLFTAKIMVLLNGGVFGVILLLTLLLFKG
SKRVVLLGWICVGFSVSVFVAPLSIMRRVIQTKSVEYMPFSLSLSLTLSAVVWFLYGLLIKDKY
VALPNILGFTFGVVQMVLYVLYMNKTPVAATAEGKDAGKLSSAADEHVLVNIAKLSPALPER
SSGVHPVTQMAGVPVRSCAAEATAPAMLPNRDVVDVFVSRHSPAVHVA

>VV01G00850

MAMAMANHHTLGLIFGILGNIISFLVYFAPAPTFYRIYKRKSAEGFHSLPYIVALFSAMLWLYY
ALLKKDAFLLITINSFGCAIESFYILLYFFYAPMQAKKQTLKVVISLNVGVFSILVVLIQFLLKGS
NRINVFGWICASFSVAVFAAPLSIVAKVIRTKSVEFMPFSLSFFLTLSAIMWFAYGLLKNDPCVA
IPNILGVILGLVQMVLYGFYRNAGKEKMEKKLPEHIIDMVMLSTLGTSDIHPIGAQQNGIKKSG
SEDVKDDEETGNREKSTENSGELQPNGSTV

>VV02G05170

MSSTEVARTAVGILGNIIALFLFLSPVPTFISIWKKGSVEQYSPVPYLATFINCMVWVLYGLPMV
HPHSTLVVTINGTGFVIELVYLILFIVFSNRGNRLRVIMIALVEIIFVAIVALLTLTMVHTTDRRS
MIVGTICILFNIMMYASPLSVMKMVIRTKSVEYMPFFLSLAAFGNGIAWTTYALIRFDLFITVPN
GLGTLFAAAQLTLYAMFYKSTKRQLAERKQGKVEMDLAQVVVTAEPMDKAQNGGGGVHE
VRRT

>VV04G04390

MAVVTVKQLAFIFGLLGNLVSFMVYLSPVPTFFKIYKRKTSEGYQALPYSVGLLCASLFLYYA
LLQSGKFLILSINTIGSTIQATYLVLFIIYSPRAGKVATLKMILILNVASLGLVLLLTTLFSKGKTRI
QVVGWISAGVNIGTFVAPLSIIKRVIETRSVEYMPFNLSFFLTICATMWFFYGIFVRDFFIAIPNV
VGFVFGIAQMFLYIIYKYMMKSDETTLEQLEETTERPLYVPTANHEPSGQELKAVTITSPRQVD
YFTEHHPMFMERDEYLS

>VV05G12700

MESLSFFAGVIGNIISVLVFLAPIGTFWRIVKHRSTQDFESLPYVCTLLNSSLWTYYGIIKPGEILV
ATVNGFGVVVEAAYVTLFLIYAPAKMRAKTVALVSLLDVGFLAAAILVTRLALQGDTRIDAL
GFICSGLNIVMYGSPLAAMKTVVTTKSVEFMPFFLSFFLFLNGGIWTIYAVLVRDYFLAVPNGT
GLVLGTAQLVLYAIYRNSKPSNKFSIEDGSQEEHLIASSS

>VV07G03830

MAMLTVPHMAFAFGILGNIVSFLVYLSPLPTFYRIYKRKSTEGFQSIPYSVALFSAMLLLYYAFL
KTDNQIMLITINSVGTCIEATYLLVYMIYAPRTAKIYTAKLLLLFNTGVYGAIVLSTFFLSKGHR
RAKIVGWVCAAFSLCVFAAPLSIMRLVIRTKSVEYMPFPLSFFLTICAVMWFFYGLLIRDFYIAF
PNILGFAFGIAQMILYTIYKNAKKGVLAEFKLQELPNGLVFPTLKKAENTDTNPNDQPEDTAM
TEGGARDKAVEPSGELKHNSSSLVVRFCLRALRLSFHHVSFSRIIAYTNQRNTVNTMRVYLLYI
AMYENKSILVFITLFSQIL

>VV10G03020

MSSVYSVCCDAAGIAGNLSAFVLFVSPIPTFRRIIRNGSTEQFSGLPYIYALLNCLICLWYGMPL
VSPGIILVATVNSVGAIFQLIYIGIFITFAEKAKKMKMSGLLTAIFGIYAIIVFASMKLFDPHARQL
FVGYLSVASLISMFASPLFIINLVIRTRSVEYMPFYLSLSTFLMSLSFFTYGMFKHDPFIYVPNGI
GTILGVVQLVLYAYYSRTSTEDLGLRESFIESYA

>VV14G09050

MASLSFIIGIIGNVISILVFASPIGTFRRVVKKKSTENYKGIPYITTLLSTSLWSFYGILKPGGLLVL
TVNGAGAIMQFIYVTLFLIYAPRDVKIKSMKVAAVLDVGFLGAVIALTLLAFHGSSRLICVGIF
CAGLTIVMYASPLSAMRMVIKTKSVEFMPFFLSFFLFLNGGVWSVYAVLVTDFFIGVPNAVGF
VLGSAQLILYAVYRNKSRPSATSEERVEEEGSAHTVKRAVEMQVSKDDGKASPKNHSLSKGR
SLPMPFISRQYSLQKIMRTLSWSPCELQDRQQDKDIEKGDI

>VV14G09070

MEGLSFFVGVIGNIISVTVVLSPIKTFLRIVKHRSTEDFESFPYVIALLGTSLWCYYGVIKPGGFIL
ATTNGLGIIIELVYVTLFIIYAPLRVRAKTAIYLGILNVAVPAIVILITLFTMHGDLRIDVLGFVCA
GLSIVMYGSPLVVVKRVLTTKSVEYMPFLLSFFFFLNGGIWTVYAILVKDFFLGVPNGIGFLLG
TAQMVLYAMYWKSKSSQNISEECQMGLDFFLEQLRWCSMPYTGNLSHPKIFQSWRMDGNTSI
SYPKTVLKTRCLTFMEERKEKNWNIISVLYMLSPVPTFSRIVKHRSTEEFESLPYVSSLATSSLW
VFYGLMKSGGLLIATVNGFGIIIELVYVILFLIFAPTRMRAKTAILVVTLNVGFPAGVVLITLIVM
DGDLRLDVLGIVCAVLNILMYGSPFTAMKKVVMTKSVEYMPFLLSFFLLLNGAIWTFYAILVK
DFFVGVPNGIGFILGAAQIVLYAMYWKSKTSQNLSDKLKGRSMDSATSQRLSISLAATLSLILIH
LTLVNSFTEIPTAYEVLEDYNFPVGLLPEEVVTGYKLNHRTGEFSAYLNDSCSVYEGGYRLKY
EPTIKGYISNGKISSLEGVSVMFFHKWRKIVEILRRDNHIHFSAGVARDRFHIKDFEESPQCVCS
MNLLDLSH

>VV14G11780

MTGADTARTVIGIIGNVISFALFASPSPTFWRIWKKRSVEEFSPDPYLATVMNCMFWIFYGLPV
VHPNSTLVVTINSIGLAVELIYLTIYFVFAPNKGRLKVIGVLCLELAFMAAVVVVTLTKLHTHA
SRSNLVGIFCVVFGVLMYASPLTVMKKVITTKSVEYMPFYLSLTNFLNGVIWLTYALIQFDLYI
TIGNGLGAVSGAIQLILYACYYKSTPKDKEGKEKGKSSEVELASPKRLNKPTPQATAANTAA

>VV16G06810

MGDRLHLAIGVMGNAASLLLYTAPILTFARVMRKKSTEEFSCIPYIIALLNCLLYTWYGLPVVS
YRWENFPVVTINGLGILLEFSFILIYFWFTSPRGKIKVVGTVVPVVTVFCITAIISSFVLHDHHHR

KMFVGSVGLVASVAMYGSPLVVVRQVILTKSVEFMPFYLSFFSFLTSFLWMAYGLLGHDLLL
ASPNLVGSPLGILQLVLYCKYRKRGIMEEPNKWDLEGNDEKSKQLQPVINNDSNGKI
>VV17G01950
MVSKDTARTIVGIIGNIISFGLFASPIPTFKKIYHEKTVGGFKPDPYLATVLNCSLWVLYGLPFVH
PDSVLVITINGIGLVMEIIYVSIFFTYSDWAKRKKIVMALLCIVIFVAAVAGITMGAFHTHHDRS
MFVGILCVVFNVVMYASPLTVMRRVIRTRSVKYMPFFLSLANLMNGIVWLIYALIKIDAYIVIP
NALGTISGLVQMVLYAAFYKSTPREEEEVKKTQEVQLSGI
>VV17G01960
MDFVLYPIHPSQQPSYHHNQWWWLCHRGHLCDQCDHLFYICRCTKTWKKIVLWFVIGMIFIGI
LATITMLFFHGTLKRSLFIGLFCVAFNITMYTAPPTIMILIGINMESLKKVKAINLKANFLSPN
>VV17G01970
MVNPDTIRTIVGIIGNVISFGLFASPIPTFIQIVKKKTVGEFKPDPYLATVLNCMMWVLYGLPFV
RPDSLLVITINGGGLVIELIYVTIFFVYADSLKRKKIALWLLFEVIFMAIIAAITMLLFHGTKNRSL
FVGLLCVVFNVIMYASPLTVMRQVIRTKSVKYMPFTLSLANFANGIVWSIYALIKFDPYILIPNG
LGSLSGAVQLILYATYYKSTPKDEEDKKPPEVQLSGM
>VV17G08600
MALFPIHHPLVFIFGILGNLISFMVYLAPLPTFYQIYKRKSTEGFQSVPYVVALFSAMLWIYYAF
LNTDASLLITINSVGCVIETSYIVMFLVYAPKKARITTVKLVFLMNICGFGSILLLTLLLAEGANR
VRILGWVCLVFSLSVFLAPLCIMRQVIRTKSVEYMPFLLSFFLTLSAVMWFFYGLMLKDFYIAG
PNILGFVFGIVQMVLYLIYRNRKKVLENEKLPELSEQIIDVVKLSTMVCSEVNLTNQQHSNEGH
GTTGLEVIVAL
>VV17G08610
MAMFTVGHHPWVFASGILGNLMSFLVYLAPIPTFTRVIKKKSTEGFQSVPYVIALFSAMLWMY
YGLVNTNASFLLSVNGFGCFIEIIYISIYLIFAPRRARILTLRLLLLINLGAFCLILIVTNFMVKRPH
RVKAVGWVCLIFAVSVFAAPLSIMASILYRLVIRTKSVEFMPLPLSICLTLSAVGWFFYGILQM
DLYIAMPNTLGFVFGLIQMILYAMYRNSTPVTKEPKLPEQVIDIVKLNTNSTPEVHPVSTLQPN
CVENEGGNGQNARKETEHAEESMGGSNRV
>VV18G11480
MDAHHALHFTFGIFGNATALFLFLAPLITFKRIIKSKSTEQFSGIPYVMTLLNCLLSAWYGLPFV
SKNNILVSTINGTGAAIEIIYVLIFIAYSIKKERAKILGLFIFVLSVFGVVVFVSLFALHGHSRKLFC
GLAATIFSIIMYASPLSIMRMVIKTKSVEYMPFFLSLFVFLCGTSWFVFGLLGKDPFVAVPNGFG
CGLGAMQLILYAIYCKKGKSKNLAAADKPVDMELGKPQQEKQSRAQNGNV
>VV19G00960
MSRSLLLPVNTICKDAAGVAGNIFAFGLFVSPIPTFRRIARNRSTESFSGLPYIYALLNCLVTLW
YGTPLVSYNNIMVTTVNSMGAAFQLVYIILFITYTDKRKKVRMFGLLMVDIVLFLVIVVGSLEI
SDFTIRRMVVGFLSCAALISMFASPLFVINLVIQTRSVEFMPFYLSLSTFLMSASFLAYGILNNDP
FVYVPNGAGTVLGIVQLGLYSYYKRTSAEESREPLIVSYG
>VC00001G04430
MAMRRLLDDHDDMDFKEVLLKHIAPGLGCIIAFLMFVSPLKAVLQVRASKHLGDLNPLPLVAI
IANCAAWLLYGCINADPYVILANEPGLLLGVFMTVSSYGFADPRARDLMLKALLFFTVIISGAG
ITIALFVERDHTASLISGYTAVFVLLCYYGAPLSTISEVVRSRSSASLFWPISVMNTVNGLLWVA
YGTAVEDLFIAVPNAIGATFGLIQLVLIQCYPAKKAVVAVGGDRGDSDPLLQDSKHVA
>VC00025G01390
MGAFTETAVPIFGNILATAMLLSPFPAVLRLRQTGKLMDINPLPYPMTCINAAGWVAYGYAVA
NPYIFPANIIGFLAGMFFTLTAFSCAPQKLQDLITGLLVAGSGYFIMLGLISCFGLAQTESQRMW
GISAVAILMCYYFVPLSTMVSIVRTRNAASIYPPLAATAIANGSMWTIYGLAVKDINLWLPNMF
GAVIGAVQLILRLVYGARSVGDAPAVTVADEEAFVVVHKGAGAPVEDRMDSGTNLLRPGHV
RSSGQGATGPTGPGGAAEPTANSGATSRHWSDDASAASPASPSDPGQSGTAQPAGGPLTAAP
>SM00001G06310

MAIAATIIGVAGNVVAALMFLSSILTFIRIAKKKSTESFSSVPYIASLLNCILWVLYGSPINKNAM
LVVTINGLGTVLNVIYVFLFLFYARKSPKALKRTSLYTFSCLALVAAVGFGISLGIHSKDTRITIF
GVLCIVLNIAMYWSPLSVMYRIFKTKSVEFLPFYLCLTVFINSALWFAYALLKHDIYILVPNVL
GLAGGAVQLFCHYIY
>SM00002G05400
MAIAATIIGVAGNVVAALMFLSSILTFIRIAKKKSTESFSSVPYIASLLNCILWVLYGSPINKNAT
LVVTINGLGTVLNVIYVLLFLFYARKSPKALKRASLYTFSCLAIMAAVGFGISLGIHSKDTRITIF
GVLCIVLNIAMYWSPLSVMYRIFKTKSVEFLPFYLCLTVFINSALWFAYALLKHDIYILVPNVL
GLAGGAVQLFCHYIYYKPGNLLTWQVPDEKEAEESESPDLESGIELPKQNGKFVDVALNTSPT
ATQS
>SM00006G05800
PTFWEIVRSKSTQEYSGLPYVCTLFNCMLWILYGMPFVKPHSMLIITINAAGCAIELVYTALYLS
YATRAKMVKVLKMLGAVAVAFGLITLTTVKLADTHDERITVVGSVCVAVAIAMYISPLTVMK
LVIQTRSVQYMPFLLSLFVFLNSLVWTFYAVVTRDIFIAIPNGLGCLSGIAQLSLYAIYRNSS
>SM00013G00320
PTFWRIYKNKSVEKFSWLPYATGLLYAAYWGCYALPFITEHNMLLFTVSVAQAVLELIYLIIFL
VYSSPKQRASVAGAIFGVAASVAATIAVAKSAMHKRPERCMFAGLPAAIVTVAMYASPLTVM
RLVIKTKSVEYMPFLLSFSIFVNSVAWTIYGVLQLDYFILISEGLGAILGTSQLVLYALY
>SM00013G00420
FVSTGNITTILSSLAPIPTFYRIYKRKDTENFSVLPYITTILCNLFWAWYALPFITSQNLLLFIISAIQ
VVLQSIYVIMFFIYAPPERKSRTTVMVVTTVILFAMDIIITMAFLRQSKRETFAGVIATISSILAYA
APLSIMGLVIRTRSVEYMPFLLSLAIFCSGFTWTVYGILGPDIFVIISDGLGFLLSTLQLILYAVY
>SM00013G02570
MGVADTIIGICGNIAALVLFLVPAKTFNTIRKKKSTLDFSGIPYVTTLLNCLLWVLYGLPVNKG
NVLVMTINSSGIVIQTVYILLFLYYASSWAARRKILGIFVFDIVATAALGAGVILGVHSKATRITI
LGISCVVLNIGMYYAPLSVMWLVIKTKSNEYMPFLLSLMVLINSSFWTIYAFLLMDIYIIIPNTL
GLAGGIFQMILYFCYRKPAQQVEGDTRSTSKADVEIGRMEQKQNSTRTF
>SM00018G01770
GNVIAFGLFMSPLPTFYKVIRLKDTEQFSGVPYVATLLNCLLWTLYGLPFVTPNSLLVVTINGIG
TALESTYLCVYLFYAPNKPRAKVLKMLAVVLTFFAAVALMVMTITHVHKTRQLIVGVLCVIV
GTAMYASPMSVMVRKLVIQTKSVKYMPFLLSLTAFLNGLTWTAYAFLGKIDPFIVVPNAIGTC
LATTQLILYAIY
>SM00018G01830
MTLAGAIRTVMGIIGNVIAFGLFLSPAPTFRSIVKNHTTGDFSGAPYVATLFNCLLWVLYGLPF
VTSNSVLVITINTIGCVIESVYLGIFLFYASKRIEKARVAGMISIVLTVYLGIVLAVFMASKDHHT
RRKFAGICCAVVTIAMYASPLSIMRTVISTKSVQYMPLLPLVAGLFNGATWTAYGFLGQPHDY
YIVVPNLVGACLAVIQLILYGFYSRTGKPRPIVKDLWPRIEHHAGCCNQQAAV
>SM00019G01560
MPSQRNNNKKETSQMAPSNATTKRLFHGKAAAFPTLLIDEANRFHDWSLCPKEQHHLRNLQD
YFFKETNTANVILCSSEDIRAVEGARSILDLRRSTTRSVGCIERERRHSTAWCNTSSSQLLVSWY
KGQYPLVIHFHDVNCAGSSRKKEGIDIMNLLFSLFVVTPSKMLLEILGLATLIVSGKQWQMSTC
SWESLVEKNQGNVTAMVMFLSPTPTFWRIINSRDTGSFSPVPYACTLLNCLLWFFYGLPAVTS
NNTLIVTINAAGIILECIYLIVFFTFAPATHRGYLSMLLVGVAGFFAAAIAVTLTAFQQEQRAKF
VGASWYQSYSSS
>SM00024G01450
RPTFSIIYKQKDTGRFSAFPYVCTLMNCLLWFFYGLPIISENNILVLTINGAGIVIEAVYLVIFIYY
AAWPVKVRSIARVLLLFVIFFCAITFAITLGAFEGDDRTTFLGSINVIINTMMYAAPLSVMKMVI
ETKSVEYMPFMLSLCSFVNATIWALYGILKQDKFIIIPNGLGVLLGALQLGLYAKYRK
>SM00044G00730

GNATAIAVYASPIPTFSIISRKKSTEMFSVVPYVLTLLTAALGLYYGMMKSGGGLLIVTVNCVG
CVFELAYIIIFYKYASKASRRKIWKLLGVELFILCSLILITLFATRGKLRIIVIGSVASAIAIAMYAS
PLSVMRTVIRTKNVEAMPLTLTIFLLINGILWSGFAFFTKDIFIGVSSRSNQKKKTKDPTLTPS
>SM00061G02030
MVRNNNKRETSQMASSHSTTKRLFHGKAAAFPTLLIDEANRFHEWSLCPKEQHHLRNLQDYF
LMLTKETNTANVILCSSEDIRRVAGTCEQASWRLEKEAFLSSVLFDVETMSQASFQRRHSTAW
CNTSSSQLLVSGLDVVQRPISSVTISKIVKVYLALTSYRSAPKMSTCSWESLVEENQGNVTAMV
MFLSPTPTFWRIINSRDTGSFSPVPYACTLLNCLLWFFYGLPAVTSNNTLIVTINAAGIILECIYLI
VFFTFAPAAHRGYLSVLLVGVAGFFAAAIAVTLTAFQQEQRAKFVGAVCVVVGTLMYASPLS
VMKLVIATRSVEYMPFSLSLCSLINALLWTIYGVLKHDKFLIVS
>SM00100G00370
LFSLPGNITSIMAYASPVPTFWYIFKKKSTEYFSALPYVCTLLTVLLGLYYGCIRPNGMLIITINIV
GITFEATYLAIFITYATKFSRIKTVKLVLLDLAVFGVAVLLTMFLSHGKLRVMLVGSMCSAVAI
SMYAAPLSVMRMVIRTKNVEFMPITLSAFLAVNASLWSAYSFFSRDIFIGIPSALGSLLAIAQVL
LYLFYRNASK
>SM00100G00700
SVDGGVGNITAVALFISPAPTFWRILRMKSTQDYSGLPYVCTLFNCMLWVFYGMPFVKTNGM
LIITINAAGCAIETVYLLIYLIYAPKLAKMKVLRMLGAVLAAFAMVVALTMLLAHTHDARTTI
VGSVCVVVAVAMYVSPLSVMKLVIQTRSVEYMPFLLSLFVLINSLVWMLYAVATKDIFI
>SM00716G00010
LFSLPGNITSIMAYASPVPTFWYIFKKKSTECFSALPYVCTLLTVLLGLYYGCIRPNGMLIITINIV
GITFEATYLAIFITYATKFSRIKTVKLVLLDLAVFGVAVLLTMLLSHGKLRVMLVGSMCSAVAI
SMYAAPLSVMRMVIRTKNVEFMPITLSAFLAVNASLWSAYSFFSRDIFIGIPSALGSLLAIAQVL
LY
>PP00039G01320
MRVAGNITASFLFLSPVPTFWRIVKSRKVDDFSGMPYLTAALNTCLWTLYGLPFVSFQVLVVT
VNAAGAGLEISYIIIYLMYSEGKARMRVVKFFAVMVCGFILMTGLVLGLVDSVDTRKTILGVM
GAFLGSLMYAAPLTVMRMVIQTKSVEFMPFLLSLFVFLNSTTWTIYAGVPETDLYILIPNGLGL
LLGTTQLVLYAMYRGSTPRKPSLPTFSYKLAVETPPKFAPAPDSKANRPLGPGNQKAPENV
>PP00054G00300
MGHVDFKVILGVLGNITAICLFASPIPTFINIVKKKSVGDYSGIPYVCTLLNCLLWVVYGLPVVE
YQVLVVTINAAGCIIELIYLALYLKNAHKSIRMKVMKVLLAVLILFTLVTVIVLELIHDKKKRK
LVIGTLCAVFAVGMYVSPLTVMRMVIRTRSVEYMPFLLSLFNFINGLVWFGYAFIGGLDIFIAIP
NGLGALSGVAQLSLYAFYRNATPVVRDRDDVEKAKHMKPNTDSVYVQMGQNGHPPQSEAN
GAH
>PP00127G00700
MLSVRVSCNFYSPTFVDIVKRKSVGDYSGIPYICTLLNCLLWVVYGLPVVELQVLVVTINAAG
VVIEMIYIGLYLKNAQRSVRVKVMKVLLAVLILFTAIAVLVFVLIHDRKTRKLLVGTLCAVFG
VGMYISPLAVMRLVIWTRSVEYMPFLLSLFNFINGLVWFGYAVIGHLDIFIAIPNCLGALSGVA
QLSLYAYFRPATPTVRDRNEEKGNSMKWVSSSVSILVEQNDHPPLNQPCGSIEALQICEKASN
>PP00240G00170
GNITAICLFTSPIPTFIKIVKKKTVADYSGFPYVCTLLNCLLWVVYGLPVVEFQVLVVTINAAGC
FIEFLFLTLYLLNAEKKIRMKVMKLLMLVLVSFIAVTVLVLELIEDKKKRKTVIGTLCAVFAVG
MYASPLSIMRMVIQTRSVKYMPFLLSLFNFINGLVWFGYAFIGGVDIYIAIPNGLGAASGIAQLA
LYAFYRNATPRDGDEKGNPTKATNNNFASIELEKNGAQKQSSHVSKSQTNEIV
>PP00245G00180
GNVFSFIMFFSPLPTFWTIIKRRETGQFSVVPYVATLLNCLMWLFYGTSSVAGLMLVLTINAAG
VVIESIYIIIHVLFGDFESRKRTGCYFLGIMVLYTIVLCCVTQAVEVNDRVTVVGAICVVIGSIMY
SAPMTVIAQVIRDKNVANMPLFLSASSLINSVVWTTYGILVEDVFVIVSNAFNVDTLNVFF

>PP00307G00200
MFCPVCCWSGNITAICLFTSPVPTFSKIVKKKTVAEFSGIPYVCTLLNCLLWVVYGLPIVEFQVL
VISINAAGCLIEFTYLALYLTYAQKSIRMKVMKVLMAVLITFIAVTILVLELVHDKKKRKLIIGT
LCAVFAVGMYVSPLTVMKMVIQTRSVKYMPFLLSLFNFINGLVWFGYAFFGGIDIFIAIPNGLG
ALSGIAQLALYAFYRNATPRDEDEKDGPTKPTNNSIEMEKNDTYKQSNV
>OT04G01700
MPSAAEAVTLYVAPALGTVLAMFMFGSPLPEISRSREKGTIGSLNPTPYPIVAANCASWMMYG
AISGNYWVYCPNFTGLLAGAYYSGVSYALSERHRPVLEKLSGGLIFLVSLIGMVLSCVMRGSS
ENSRLMVAGIQANTILAVYYVSPMSTMSEVVRTRDSKSMHFPLVVTNFLNGLCWFAFGIGLN
DWWLAAPNLFGACVSVVQIGLIMVFPNSERRAGISTTPSTDGLMELNPTSSFSESPRSSTAV
>OS01G12130
MVSNTIRVAVGILGNAASMLLYAAPILTFRRVIKKGSVEEFSCVPYILALFNCLLYTWYGLPVV
SSGWENSTVSSINGLGILLEIAFISIYTWFAPRERKKFVLRMVLPVLAFFALTAIFSSFLFHTHGL
RKVFVGSIGLVASISMYSSPMVAAKQVITTKSVEFMPFYLSLFSFLSSALWMIYGLLGKDLFIAS
PNFIGCPMGILQLVLYCIYRKSHKEAEKLHDIDQENGLKVVTTHEKITGREPEAQRD
>OS01G21230
MASVVDLIESSFPIATKILNKTTIDRTIKWAVGLDIGLSGAVLAVATFAISQLQLRIRVIGIICACF
NVLMYASPLTAVINVIQHENVDAMPFWLSFFLFLNGGVWLVYGIIDRDMLIGIPNGIGFLLGTI
QLIVYAIYANFIHCRRLRLFLRGLVGRQALVAPLLPNAVEGQEA
>OS01G36070
MMNALGLSVAATSTGSPFHDVCCYGAGIAGNIFALVLFISPLPTFKRIVRNGSTEQFSAMPYIYS
LLNCLICLWYGLPFVSYGVVLVATVNSIGALFQLAYTATFIAFADAKNRVKVSSLLVMVFGVF
ALIVYVSLALFDHQTRQLFVGYLSVASLIFMFASPLSIINLVIRTKSVEYMPFYLSLSMFLMSVSF
FAYGVLLHDFFIYIPNGIGTVLGVIQLVLYGYFRKGSREDSLPLLVTHT
>OS01G40960
MWPSFFLPPLSLLSLSLPFFPFCRHAGWQQERAAAGGEAGAAAVGGRAARSSGGALRRRGSC
ARRESKRRRKRTPTLDTAQPGAALGGESERRRQVTTFKRILKAKSTERFDGLPYLFSLLNCLIC
LWYGLPWVANGRLLVTTVNGTGAVFQLAYICLFIFYADSKKTSVILPILHLILS
>OS01G42090
MISPDAARNVVGIIGNVISFGLFLSPVPTFWRICKRKDVEQFKADPYLATLLNCMLWVFYGIPIV
HPNSILVVTINGIGLIVEGTYLFIFFLYSPNKKRLRMLAVLGVELVFMLAVILGVLLSAHTHKKR
SMIVGILCVFFGSIMYFSPLTIMGKVIKTKSVEYMPFFLSLVCFLNGVCWTAYALIRFDIYVTIPN
GLGAIFGAIQLILYACYYRTTPKKTKAAKDVEMPSVISGPGAAATASGGSVVSVTVER
>OS01G42110
MISPDAARNVVGIIGNVISFGLFLAPVPTFWRICKRKDVEEFKADPYLATLLNCMLWVFYGIPV
VHPNSILVVTINGIGLLVEGTYLLIFFLYSPNKKRLRMCAVLGVELVFMLAVILGVLLGAHTHE
KRSMIVGILCVFFGSIMYFSPLTIMGKVIKTKSVEYMPFFLSLVCFLNGVCWTAYALIRFDIYVT
IPNGLGALFGAIQLILYACYYRTTPKKTKAAKDVEMPSVVVSGTGAAAAAGGGNTGGGSVSV
TVER
>OS01G50460
MDSLYDISCFAAGLAGNIFALALFLSPVTTFKRILKAKSTERFDGLPYLFSLLNCLICLWYGLPW
VADGRLLVATVNGIGAVFQLAYICLFIFYADSRKTRMKIIGLLVLVVCGFALVSHASVFFFDQP
LRQQFVGAVSMASLISMFASPLAVMGVVIRSESVEFMPFYLSLSTFLMSASFALYGLLLRDFFI
YFPNGLGLILGAMQLALYAYYSRKWRGQDSSAPLLLA
>OS01G65880
MTLLNCLLSAWYGLPFVSPNNILVTTINGTGSVIEAIYVVIFLIFAERKARLKMMGLLGLVTSIF
TMVVLVSLLALHGQGRKLFCGLAATIFSICMYASPLSIMRLVIKTKSVEFMPFLLSLSVFLCGTS
WFIYGLLGRDPFIAIPNGCGSFLGLMQLILYAIYRNHKGATPAAAAGKGDAADEVEDAKKAA
AAVEMADAKTNKVVADDADADADGKSADDKVASQV

>OS02G19820
MVSPDTIRTAIGVVGNGTALVLFLSPVPTFIRIWKKGSVEQYSAVPYVATLLNCMMWVLYGLP
AVHPHSMLVITINGTGMAIELTYIALFLAFSLGAVRRRVLLLLAAEVAFVAAVAALVLNLAHT
HERRSMIVGILCVLFGTGMYAAPLSVMKMVIQTKSVEYMPLFLSLASLVNGICWTAYALIRFD
LYITIPNGLGVMFAVAQLILYAIYYKSTQQIIEARKRKEADHVAMTDVVVDSAKNNPSSGAAA
AAANGRY
>OS02G30910
MAFMSMERSTWAFTFGILGNLISLMVFLSPLPTFYRVYRKKSTEGFQSTPYVVTLFSCMLWMY
YAFVKSGAELLVTINGVGCVIETVYLAMYLAYAPKSARMLTAKMLLGLNIGLFGVIALVTLLL
SRGELRVHVLGWICVAVSLSVFAAPLSIIRLVIRTKSVEFMPFSLSFFLVLSAVIWFLYGLLKKD
VFVALPNVLGFVFGVAQMALYMAYRSKKPLVASSSSAVVAAGLEIKLPEHVKEVQAVAKGA
VAAAPEGRISCGAEVHPIDDVMPSEVVEVKVDDEETNRTDEMAGDGDHAMVRTEQIIKPDMA
IVVEV
>OS03G22200
MLGVADRSTVKREEFNRGDQAKMVKVVLAVNVGALAAVVAVALVALHGGVRLFVVGVLC
AALTIGMYAAPMAAMRTVVKTRSVEYMPFSLSFFLFLNGGVWSVYSLLVKDYFIGIPNAIGFA
LGTAQLALYMAYRRTKKPAGKGGDDDEDDEEAQGVARLMGHQVEMAQQRRDQQLRKGLS
LSLPKPAAPLHGGLDRIIKSFSTTPIELHSILHQHHGGHHHHHRFDTVPDDDDEAVAAGGTTPA
TTAGPGDRH
>OS03G22590
MVQALVFAVGIVGNILSFLVILAPVPTFYRVYKKKSTESFQSVPYAVALLSAMLWLYYALLTS
DLLLLSINSIGCLVESLYLTVYLLYAPRQAMAFTLKLVCAMNLALFAAVVAALQLLVKATDRR
VTLAGGIGASFALAVFVAPLTIIRQVIRTKSVEFMPFWLSFFLTLSAVVWFFYGLLMKDFFVAT
PNVLGLLFGLAQMVLYVVYKNPKKNSAVSEAAAAQQVEVKDQQQLQMQLQASPAVAPLDV
DADADADLEAAAPATPQRPADDDAIDHRSVVVDIPPPPQPPPALPAVEVA
>OS05G12320
MFPDIRFIVGIIGSVACMLLYSAPILTFKRVIKKASVEEFSCIPYILALFSCLTYSWYGFPVVSYG
WENMTVCSISSLGVLFEGTFISIYVWFAPRGKKKQVMLMASLILAVFCMTVFFSSFSIHNHHIR
KVFVGSVGLVSSISMYGSPLVAMKQVIRTKSVEFMPFYLSLFTLFTSLTWMAYGVIGRDPFIAT
PNCIGSIMGILQLVVYCIYSKCKEAPKVLHDIEQANVVKIPTSHVDTKGHNP
>OS05G35140
MEDLAKFLFGVSGNVIALFLFLSPVPTFWRIIRRKSTEDFSGVPYNMTLINCLLSAWYGLPFVSP
NNILVSTINGAGAVIETAYVVVFLVFASTHKTRLRTLGLAAAVASVFAAVALVSLLALHGQHR
KLLCGVAATVCSICMYASPLSIMRLVIKTKSVEYMPFLMSLAVFLCGTSWFIYGLLGRDPFVTI
PNGCGSFLGAVQLVLYAIYRNNKGAGGGSGGKQAGDDDVEMAEGRNNKVADGGAADDDST
AGGKAGTEV
>OS05G51090
MVMNPDAVRNVVGIIGNLISFGLFLSPLPTFVTIVKKKDVEEFVPDPYLATFLNCALWVFYGLP
FIHPNSILVVTINGTGLLIEIAYLAIYFAYAPKPKRCRMLGVLTVELVFLAAVAAGVLLGAHTY
DKRSLIVGTLCVFFGTLMYAAPLTIMKQVIATKSVEYMPFTLSLVSFINGICWTIYAFIRFDILITI
PNGMGTLLGAAQLILYFCYYDGSTAKNKGALELPKDGDSSAV
>OS08G42350
MAGGFLSMANPAVTLSGVAGNIISFLVFLAPVATFLQVYKKKSTGGYSSVPYVVALFSSVLWI
FYALVKTNSRPLLTINAFGCGVEAAYIVLYLVYAPRRARLRTLAFFLLLDVAAFALIVVTTLYL
VPKPHQVKFLGSVCLAFSMAVFVAPLSIIFKVIKTKSVEFMPIGLSVCLTLSAVAWFCYGLFTK
DPYVMYPNVGGFFFSCVQMGLYFWYRKPRNTAVLPTTSDSMSPISAAAAATQRVIELPAGTH
AFTILSVSPIPILGVHKVEVVAAEQAADGVAAAAAADKELLQNKPEVIEITAAV
>OS09G08030

MVSPDMIRNVVGIVGNVISFGLFLSPVPTFWQIIKNKNKNKKKMEVVLAAEALFMAAVALGV
LLGVHTHQRRSLIVGILCVIFDTIMYSSPLTVMSQVVKTKSVEYMPLLLSVVSFLNGLYWTSYT
LIRFDIFITIPNGLGVLFAAVQLILYVIYYRTTPKKQNKNLELPTVTPVAKDTSVGPISKDNDLNG
STASHVTIDITIQP

>OS09G08440

MVSPDLIRNMVGIVGNIISFGLFLSPVPTFYRIIKNKDVQDFKADPYLATLLNCMLWVFYGLPIV
HPNSILVVTINGIGLVIEAVYLTIFFLFSDKKNKKKMGVVLATEALFMAAVVLGVLLGAHTHQ
RRSLIVGILCVIFGTIMYSSPLTIMSQVVKTKSVEYMPLLLSVEAESYERKVPEILPRQAVEGKG
RGAARVLLNCDILLLNCDILSHFCFHSISDQSEISLPENELQALLLSIMFNLFEITSRSLREESNHN
SQKHRSVSLATQQMTVANANEVQSLTSTTPALAKEHPLQVQPTKNFESNSTEFVIDIEGPYDAE
DITGHTTDKTKFILVNYSNSSEEHKSQDPTQDESDNIPNKSTNVSNIYHAF

>OS11G31190

MAGMSLQHPWAFAFGLLGNIISFMTYLAPLPTFYRIYKSKSTQGFQSVPYVVALFSAMLWIYY
ALLKSDECLLITINSAGCVIETIYIAVYLVYAPKKAKMFTAKLLLLVNVGVFGLILLLTLLLSAG
DRRIVVLGWVCVGFSVSVFVAPLSIIRLVVRTKSVEFMPFSLSFSLTISAVVWFLYGLLIKDKYV
ALPNVLGFSFGVIQMGLYAMYRNSTPKAVLTKEVEAATATGDDDHSAAGVKEHVVNIAKLS
AAVDVVKTREVHPVDVESPPAEAPPEEDDKAAAATAAAVAGAGEKKVAA

>OS12G07860

MVSPDLIRNVVGIVGNVISFGLFLSPVPIFWRIIKNKNVQNFKADPILVVTINGISLVIEAVYLTIF
FLFSDKKNKKKMGVVLATEALFMAAVAVGVLLGAHTHQRRSLIVGILCVIFGTIMYSSPLTIM
VVKTKSVEYMPLLLSVVSFLNGLCWTLYALIRFDIFITIPNGLGVLFAIMQLILYAIYYRTTPKK
QDKNLELPTVAPIAKDTSIVAPVSNDDDVNGSTASHATINITIEP

>OS12G29220

MAGLSLQHPWAFAFGLLGNLISFTTYLAPIPTFYRIYKSKSTEGFQSVPYVVALFSAMLWIFYA
LIKSNEALLITINAAGCVIETIYIVMYLAYAPKKAKVFTTKILLLLNVGVFGVILLLTLLLSHGEQ
RVVSLGWVCVAFSVSVFVAPLSIIKRVIQSRSVEYMPFSLSLTLTLSAVVWFLYGLLIKDKYVA
LPNILGFTFGVVQMGLYVFYMNATPVAGEGKEGKGKLAAAEELPVVVNVGKLAAATPDRST
GAVHVHPVPRSCAAEAAAAEPEVLVDIPPPPPPRAVEVAAV

>OL04G01590

MGDTRDALTLWFAPALGSALAQVMFLSPFPEIERCKTKRSLGHLNALPYPFVAANCAAWMIY
GGISGNYWVYIPNFTGYFCGTYYSFVAYALDEKIRGTMERIVAVLIILVSFIGMVVSCVMKNSS
ESARLVVAGILANLILVVYYSAPLSTMAEVVRTKDSKSMHFPLVFCNGLNGLCWTTYGIALND
WWIAAPNLFGSVLSIVQVVLIFLYPSSERLRSRITPTPSVEGLVSMSSDSSPL

>MT0G04170

MSLFNAYSICEIGKDAAGIAGNIFAFGLFVSPIPTFRRIMRNGSTELFSGLPYIYSLLNCLICLWY
GTPLISCDNLLVTTVNSIGAAFQLVYIFLFLIYAEKPKKVRMFGLLLAVLGIFVIILVGSLKITDSS
IRRILVGCLSCASLISMFASPLFIIKLVIRTKSVEFMPFYLSFSTFLMSISFFLYGLLSDDAFIYVPN
GIGTVLGMIQLILYFYYKRSSSDDSTEPLIVSYG

>MT0G35890

MSVFASLAICKVAKDAAGVAGNIFAFGLFVSPIPTFRRIIRNGSTEMFSGLPYIYSLMNCLICMW
YGTPLISHDNILVTTVNSIGAVFQFVYIILFMMSAEKEKKVKMLAWLMGVLGIFAIILIGSLQID
DIVMRRLFVGILSCASLISMFASPLFIIKLVIQTKSVEFMPFYLSLSTFLMSTSFLVYGLLSDDIFIY
VPNGIGTILGMTQLILYFYYESKSRRMDAEEPLIVSYA

>MT2G007890

MAMISMNHHFLVIAFGLLGNIISCMVYLAPLPTFIQIYKKKSTECFQSLPYLVALFSSMLWLYY
GIQTNAIFIVSINAFGCVIEIIYCIMYIAYATKDARKLTIKLCAALNVVSFVLIFLIIQFSIPENHRV
QVLGWICTSISISVFAAPLSIVVRVVKTKSVEFMPFNLSLFLTLSAVVWFLYGFVKRDICIYLPN
VVGFILGIIQMVLYGYYSKYSVEKEKEQAVINIVVVNPLGSSEVFPIPLDENKESIEDVINQQFQ
VKKVGEEDAKEKHDNNVEAIEFQCVV

>MT3G080990
MSTAEIARTAVGIIGNVIAGCMFLSPVPTFVGICKKGSVEQYSPVPYLATLMNCMVWTLYGLP
MVHPHSFLVVTINGAGCVVEIIYITLFLIYSDRKKRLKVFLGLLLELIFIFLLSFVSLTMLHTVNK
RSAVVGTICMLFNIGMYASPLSIMKLVIKTKSVEFMPFFLSLASFGNGVSWTIYALIPFDPFIAIP
NGIGTMFAVVQLILYASYYKSTQEQIAARKNNGKGEMNLSEVVVGMSNATVQDNKKITAIDH
SSPSAK
>MT3G090940
MSETLRLAVAVLGNAASVSLYAAPMVTFKRVIRKKSTEEFSCIPYIIGLLNCLLFTWYGLPIVSY
KWENFPLVTVNGVGIALELSYVLIYFWYSSPKGKVKVAMIMTPVLLVFCIVAAVSAFSFHDTA
HRKLLVGSIGLGVSVALYGSPLVAMKKVIETKSVEFMPLPLSLCAFSASACWLVYGILVRDVF
VAGPSVVGTPLSILQLVVYFKYRKARVVEEQKIGDLEKGSIELEKVVKVEKIVTNCEQC
>MT3G090950
MSNTLRLAVAVLGNAASVSLYAAPMVTFKRVIRKKSTEEFSCIPYIIGLLNCLLFTWYGLPIVS
YKWENFPLVTVNGVGIALELSYVLIYFWYSSPKGKVKVAMITTPVLLVFCITVAVSTFFLHDTT
HRKLLVGSIGLVVSVALYGSPLVAMKKVIQTKSVEFMPLPLSLCAFSASVFWLAYGILVRDVF
VAGPSLVGTPLSILQLVIYFKYRKERVMEESKIGDLEKGSIELEKVVKVEKIVTNCEQC
>MT3G098910
MSSHHSHLSFAFGVLGNISSFVCFLAPLPTFYRICKKKSTEGFQSIPYVAALFSAMLWMFYAYT
KKGETLLITINAFGCVIETIYLAVFVTYCPKKVRMSTLRMIVLMNFVGFGTIVLLTHFLAKQEE
GRIKLLGWICVVFATSVFAAPLSIIRVVIRTKSVEFLPFPLSVLLLISAVMWLLYGLSLRDIYVTL
PNVVGLTFGIVQITLYAMYRNSKPVIDEKLPEHKGDIVDKEIENVVVPSKTTNDEKKLEVSVVD
MVIVEKKEEKQDEEHDEKEKKQDQVTQDKTKVKNENDNININKTEEKDSGCEV
>MT3G098930
MALFYSEYWAFVFGVIGNVISCMTFLAPLPTFYRIYKKKSTEGFQSVPYVTALLSAMLWIYYA
HVKNKATLLLLTINIYGFGIEAIYIIIFLLYASNKARLSTIKLLFLTVCGYGTMVILTTYLTKGSKR
LSIIGWICMVFNICVFASPLFILKQVIKTKSVAFMPLNLSFFLTLNAIVWFFYGLLIDDFYIAIPNT
LGFVFGIVQMVIYLIYKDAIPLESTKLQKPNDHVLNICEDVPNGALQPDPNQVVKSGAPAVAVI
GDEDPNNGK
>MT4G106990
MARMQVRRSALHTCCGQELKHHPNLDKCPNTYLWPTFIKICKAKSVQDFKPDPYVVTILNCA
MWSFYGMPFISKSNTLVLTINGFGFFIEIIYTSIFFVYSNGSKRVRNISNLLIKLQSIFPFNVLKIEL
KKKILLALLAEVVFLVLVVFIVMYFVTNLKERRFIVGVICIIFNILMYFSPLTVMRQVIRSKSVK
YMPFLLSLANFANGLIWTTYALLRWDPFVVIPNGLGALSGLAQLILYAVYYRTTKWDDDAPPS
SVNNV
>MT5G067530
MDPHDHDRLAFIFGILGNIISSMVYLAPLPTFYRIWKKKSTEGFQSLPYLVALFSSMLWLYYGF
VKKHAFLLITINSAGCVIETIYIVTYLIYATKDARILTIKLFMAMNVACSVLIVLTTQLAMHGKL
RVHVLGWICTSFAICVFAAPLTIMAKVIRTKSVEFMPINLSFFLTLSAIVWFFYGLLLHDICIAIP
NVLGFILGLLQMLLYAIYNKSVKEEYALEPMTNIVIVNPLGIPCEVFSLPVIDNVNKIEKEGAEE
MEKSVENLT
>MT5G092600
MFPFSNLKMVLLFGFLGIVTFMSFLAPLPTFYSIYKKKSSEGFHSIPYVVTLLSTLLFVYYGFLK
TNAIFLITINSIGCVMEVAYLIMYITYAPKKLKISTLVLILIVDMGGFGLTMIITTFIVKGSFHVQV
VGMICTIFNIGMFAAPLSIMKKVIKTRSVEYMPFPLSLFLTICATMWFFYGFFDKDKYIMLPNGL
GFLLGVSQMILYLIYKNAKNNVEASSTNQLQEHGCDGGNNQIFPTVVEMKEINIV
>MT7G005690
MAISHNTLAFAFGMLGNVISFMVFLAPMTTFYRIYKKKSTEGFQSLPYLVALFSSMLWLYYAF
LKKDEFLLITINSFGCVVELIYIILYIIYATKDARKLTIKLLLAMNIGSFGLILLVTKYAVHGPIRV
QVLGWICVSISVSVFAAPLTIVAQVVRTKSVEFMPFNLSFTLTLSAIMWFGYGLFLKDICIALPN

VLGFALGLVQMILYCIYRNGDKKKANSKAALKSVVIESSLGGTGEVFQVEKNDGEEEEEKKK
TIEETEYDSKV

>MT7G005710

MNIGSFGLILLVTKYAVHGPIRVQVLGWICVSISVSVFAAPLTIVAQVVRTKSVEFMPFNLSFTL
TLSAIMWFGYGLFLKDICIALPNVLGFALGLVQMILYCIYRNGDKKKANSKAALKSVVIESSLG
GTGEVFQVEKNDGEEEEEKKKTIEETEYDSKV
