# Supplementary figures and images for: Soybean (Glycine max) SWEET gene family: insights through comparative genomics, transcriptome profiling and whole genome re-sequence analysis
Source: BMC Genomics. 2015 Jul 11;16(1):520. doi: 10.1186/s12864-015-1730-y (PMC4499210; doi:10.1186/s12864-015-1730-y)

### Additional file 3: Gene organization of soybean SWEET orthologs.

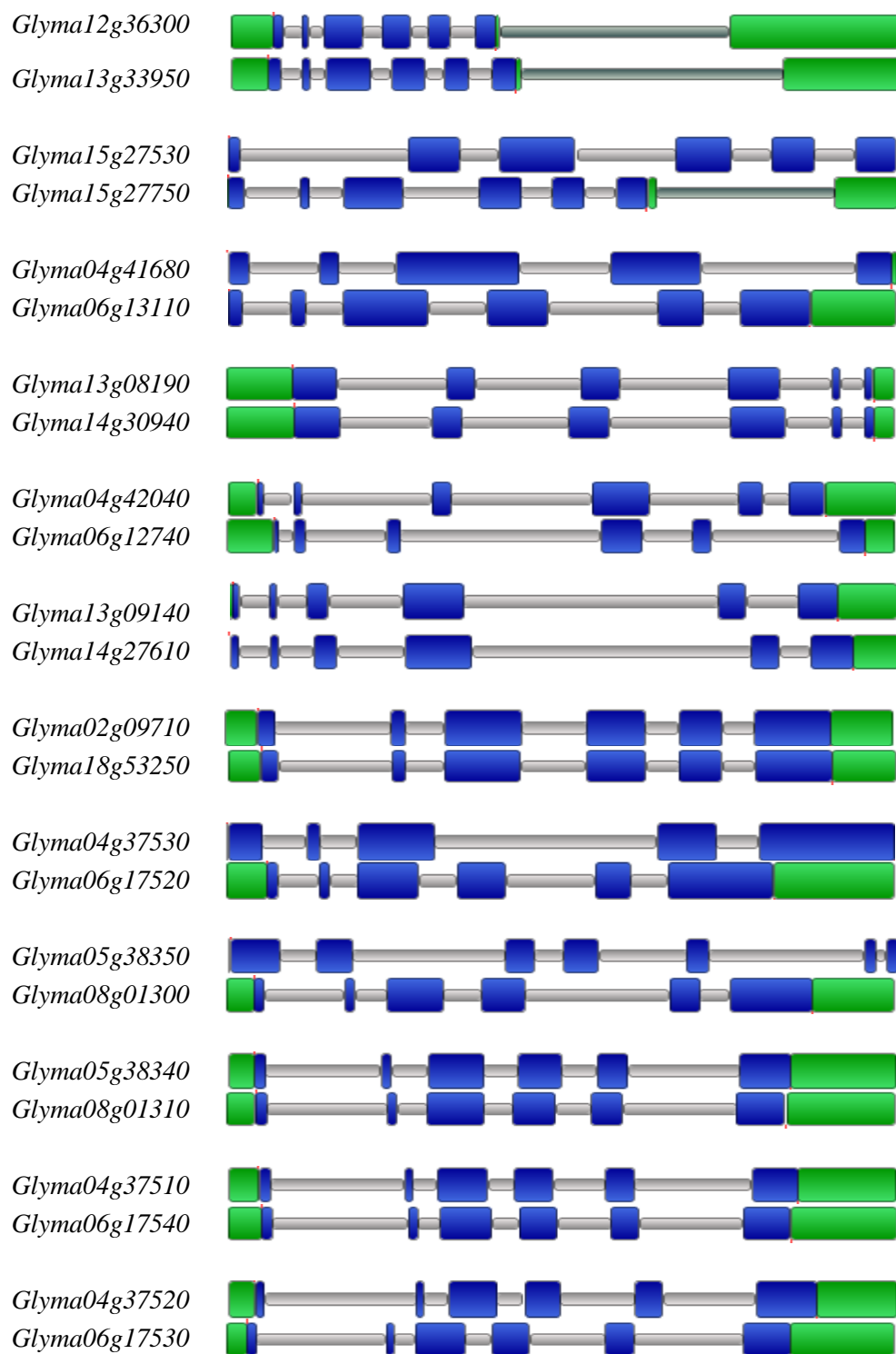

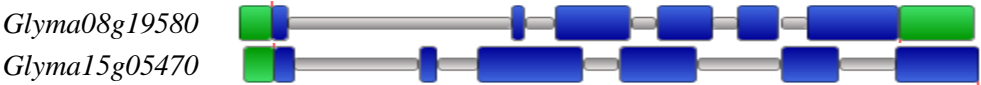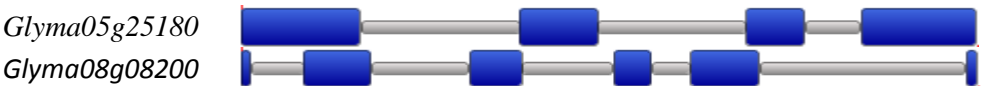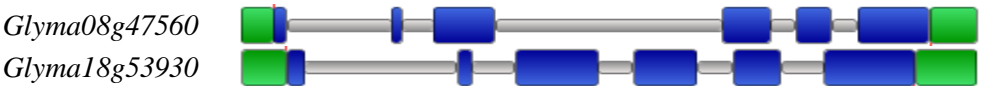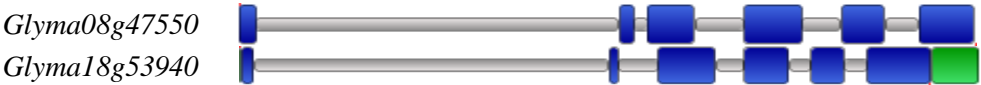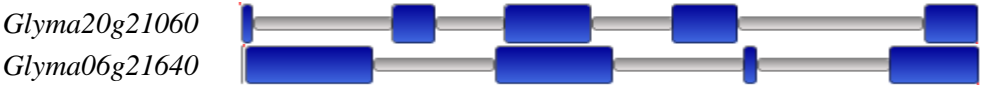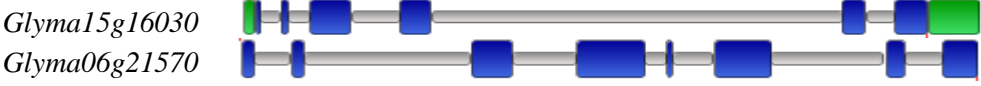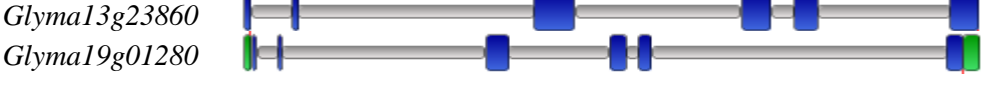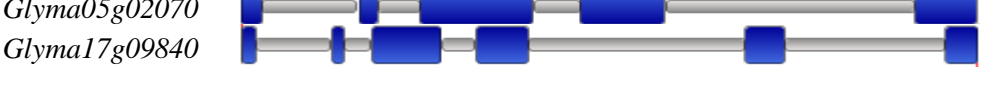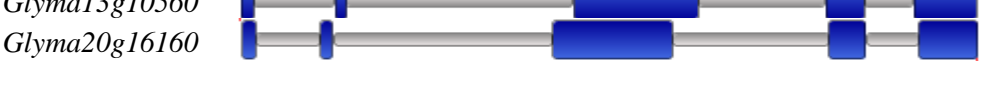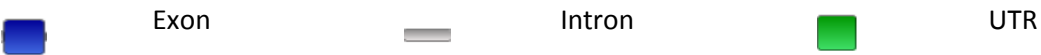

Supplement: Additional file 3: — Gene organization of soybean SWEET orthologs genes. [file 12864_2015_1730_MOESM3_ESM.pdf]
