# Supplementary material for: Soybean (Glycine max) SWEET gene family: insights through comparative genomics, transcriptome profiling and whole genome re-sequence analysis
Source: BMC Genomics. 2015 Jul 11;16(1):520. doi: 10.1186/s12864-015-1730-y (PMC4499210; doi:10.1186/s12864-015-1730-y)
Supplement: Additional file 4: — Conserved domain and associated protein architecture in SWEET ( MtN3_slv ) gene family. [file 12864_2015_1730_MOESM4_ESM.pdf]

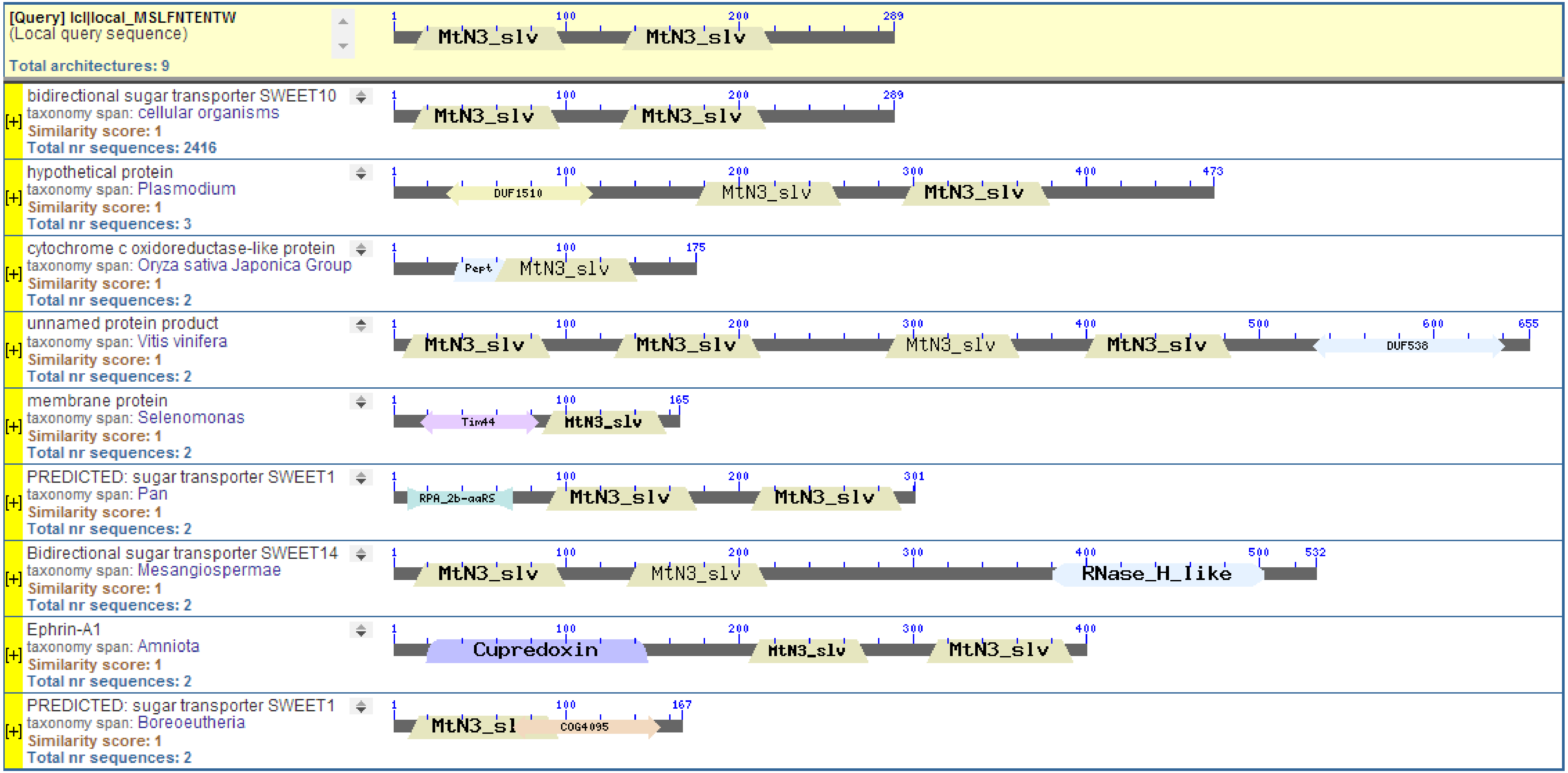
[Query] lcl|local_MSLFNTENTW
(Local query sequence)
Total architectures: 9
1 100 200 289
MtN3_slv MtN3_slv
bidirectional sugar transporter SWEET10
taxonomy span: cellular organisms
Similarity score: 1
Total nr sequences: 2416
1 100 200 289
MtN3_slv MtN3_slv
hypothetical protein
taxonomy span: Plasmodium
Similarity score: 1
Total nr sequences: 3
1 100 200 300 400 473
DUF1510 MtN3_slv MtN3_slv
cytochrome c oxidoreductase-like protein
taxonomy span: Oryza sativa Japonica Group
Similarity score: 1
Total nr sequences: 2
1 100 175
Pept MtN3_slv
unnamed protein product
taxonomy span: Vitis vinifera
Similarity score: 1
Total nr sequences: 2
1 100 200 300 400 500 600 655
MtN3_slv MtN3_slv MtN3_slv MtN3_slv DUF538
membrane protein
taxonomy span: Selenomonas
Similarity score: 1
Total nr sequences: 2
1 100 165
Tim44 MtN3_slv
PREDICTED: sugar transporter SWEET1
taxonomy span: Pan
Similarity score: 1
Total nr sequences: 2
1 100 200 301
RPA_2b-aaRS MtN3_slv MtN3_slv
Bidirectional sugar transporter SWEET14
taxonomy span: Mesangiospermae
Similarity score: 1
Total nr sequences: 2
1 100 200 300 400 500 532
MtN3_slv MtN3_slv RNase_H_like
Ephrin-A1
taxonomy span: Amniota
Similarity score: 1
Total nr sequences: 2
1 100 200 300 400
Cupredoxin MtN3_slv MtN3_slv
PREDICTED: sugar transporter SWEET1
taxonomy span: Boreoeutheria
Similarity score: 1
Total nr sequences: 2
1 100 167
MtN3_sl COG4095
