# Supplementary material for: Soybean (Glycine max) SWEET gene family: insights through comparative genomics, transcriptome profiling and whole genome re-sequence analysis
Source: BMC Genomics. 2015 Jul 11;16(1):520. doi: 10.1186/s12864-015-1730-y (PMC4499210; doi:10.1186/s12864-015-1730-y)
Supplement: Additional file 5: — Soybean SWEET protein Transmembrane Helix prediction obtained from TMHMM 2.0 tool [ 99 ]. [file 12864_2015_1730_MOESM5_ESM.pdf]

TMHMM result

HELP with output formats

```
# GM02G09710 Length: 262
# GM02G09710 Number of predicted TMHs:  7
# GM02G09710 Exp number of AAs in TMHs: 154.88708
# GM02G09710 Exp number, first 60 AAs:  38.89945
# GM02G09710 Total prob of N-in:        0.09574
# GM02G09710 POSSIBLE N-term signal sequence
GM02G09710      TMHMM2.0        outside      1     9
GM02G09710      TMHMM2.0        TMhelix     10    32
GM02G09710      TMHMM2.0        inside      33    42
GM02G09710      TMHMM2.0        TMhelix     43    65
GM02G09710      TMHMM2.0        outside     66    68
GM02G09710      TMHMM2.0        TMhelix     69    91
GM02G09710      TMHMM2.0        inside      92   102
GM02G09710      TMHMM2.0        TMhelix    103   125
GM02G09710      TMHMM2.0        outside    126   129
GM02G09710      TMHMM2.0        TMhelix    130   152
GM02G09710      TMHMM2.0        inside     153   163
GM02G09710      TMHMM2.0        TMhelix    164   186
GM02G09710      TMHMM2.0        outside    187   190
GM02G09710      TMHMM2.0        TMhelix    191   213
GM02G09710      TMHMM2.0        inside     214   262
```

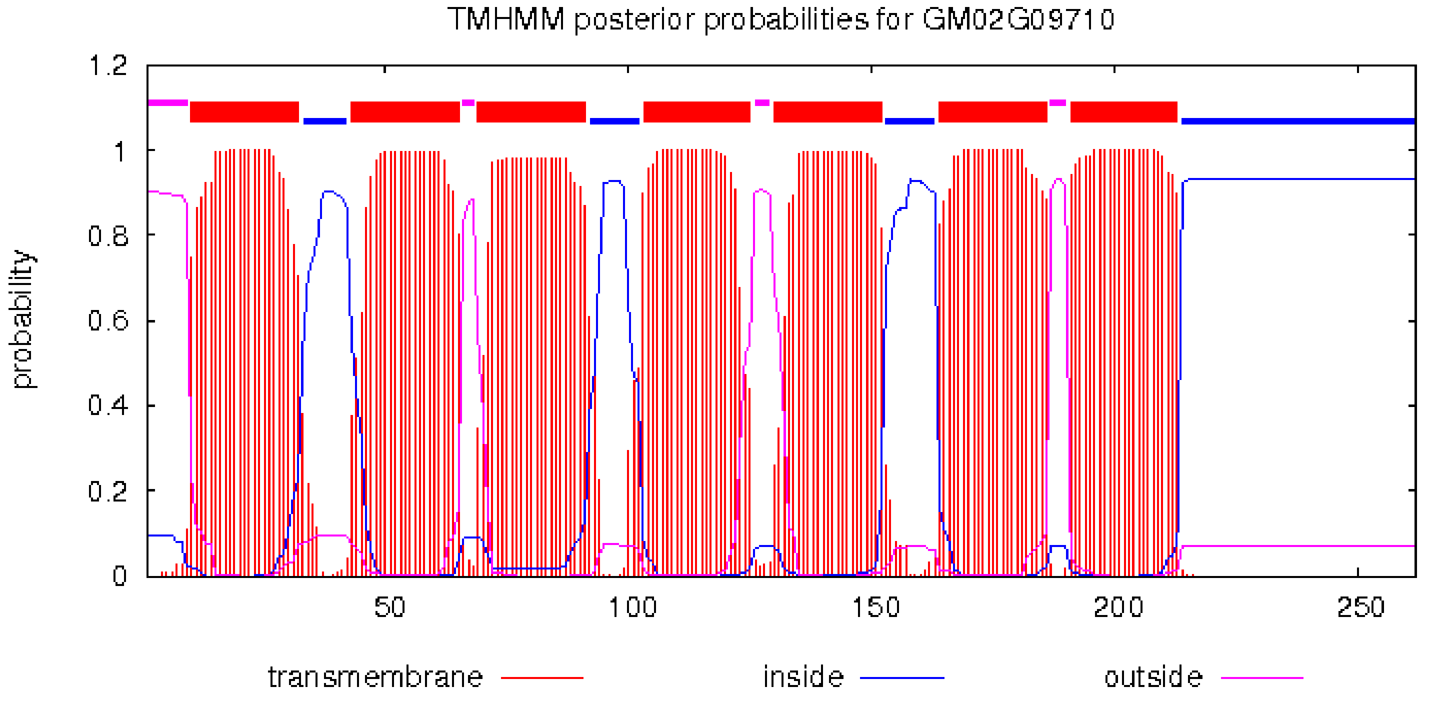

plot in postscript, script for making the plot in gnuplot, data for plot

---

```
# GM03G36790 Length: 336
# GM03G36790 Number of predicted TMHs:  7
# GM03G36790 Exp number of AAs in TMHs: 151.67854
# GM03G36790 Exp number, first 60 AAs:  22.33592
# GM03G36790 Total prob of N-in:        0.00878
# GM03G36790 POSSIBLE N-term signal sequence
```

GM03G36790	TMHMM2.0	outside	1	29
GM03G36790	TMHMM2.0	TMhelix	30	52
GM03G36790	TMHMM2.0	inside	53	64
GM03G36790	TMHMM2.0	TMhelix	65	83
GM03G36790	TMHMM2.0	outside	84	92
GM03G36790	TMHMM2.0	TMhelix	93	115
GM03G36790	TMHMM2.0	inside	116	121
GM03G36790	TMHMM2.0	TMhelix	122	144
GM03G36790	TMHMM2.0	outside	145	153
GM03G36790	TMHMM2.0	TMhelix	154	171
GM03G36790	TMHMM2.0	inside	172	183
GM03G36790	TMHMM2.0	TMhelix	184	206
GM03G36790	TMHMM2.0	outside	207	210
GM03G36790	TMHMM2.0	TMhelix	211	233
GM03G36790	TMHMM2.0	inside	234	336

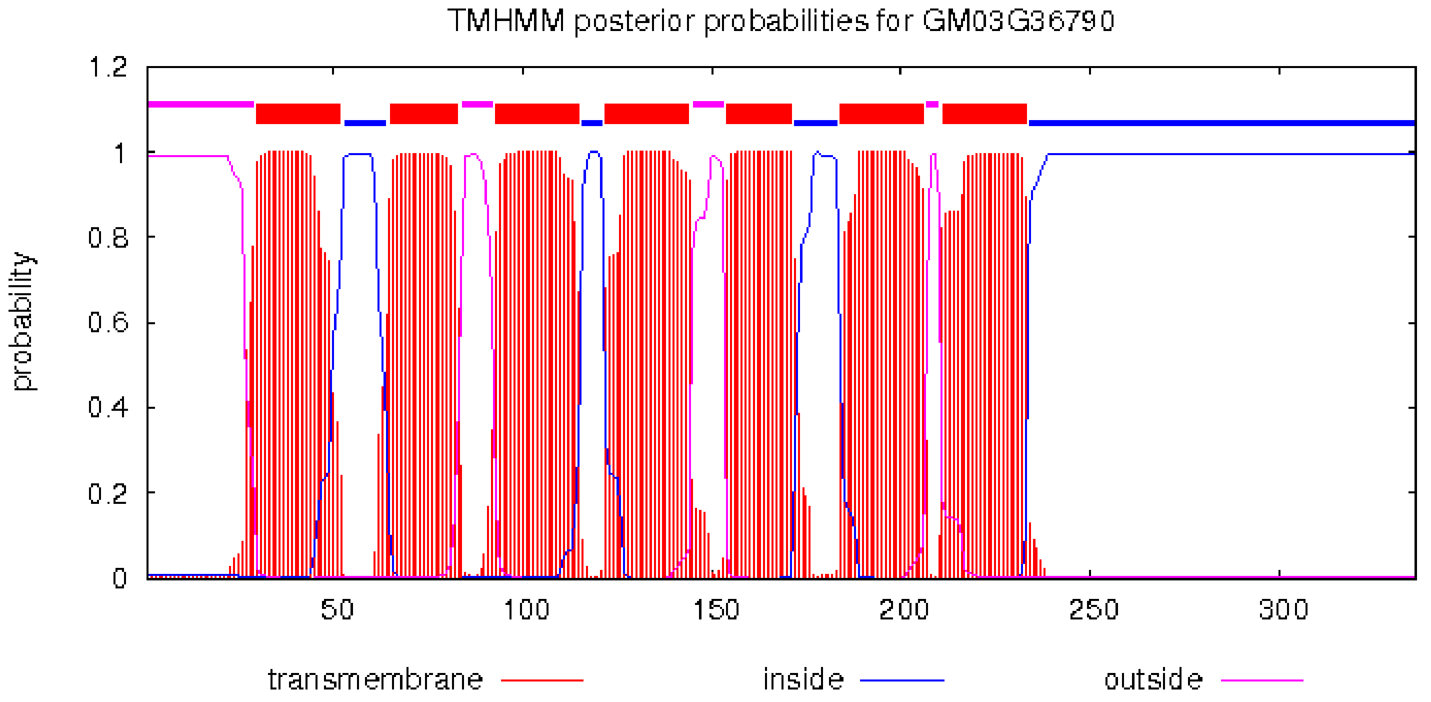

plot in postscript, script for making the plot in gnuplot, data for plot

---

```
# GM04G37510 Length: 258
# GM04G37510 Number of predicted TMHs:  7
# GM04G37510 Exp number of AAs in TMHs: 153.36579
# GM04G37510 Exp number, first 60 AAs:  39.44796
# GM04G37510 Total prob of N-in:        0.02029
# GM04G37510 POSSIBLE N-term signal sequence
```

GM04G37510	TMHMM2.0	outside	1	9
GM04G37510	TMHMM2.0	TMhelix	10	32
GM04G37510	TMHMM2.0	inside	33	44
GM04G37510	TMHMM2.0	TMhelix	45	62
GM04G37510	TMHMM2.0	outside	63	71
GM04G37510	TMHMM2.0	TMhelix	72	94
GM04G37510	TMHMM2.0	inside	95	100
GM04G37510	TMHMM2.0	TMhelix	101	123
GM04G37510	TMHMM2.0	outside	124	132
GM04G37510	TMHMM2.0	TMhelix	133	152
GM04G37510	TMHMM2.0	inside	153	163
GM04G37510	TMHMM2.0	TMhelix	164	186
GM04G37510	TMHMM2.0	outside	187	190

GM04G37510	TMHMM2.0	TMhelix	191	213
GM04G37510	TMHMM2.0	inside	214	258

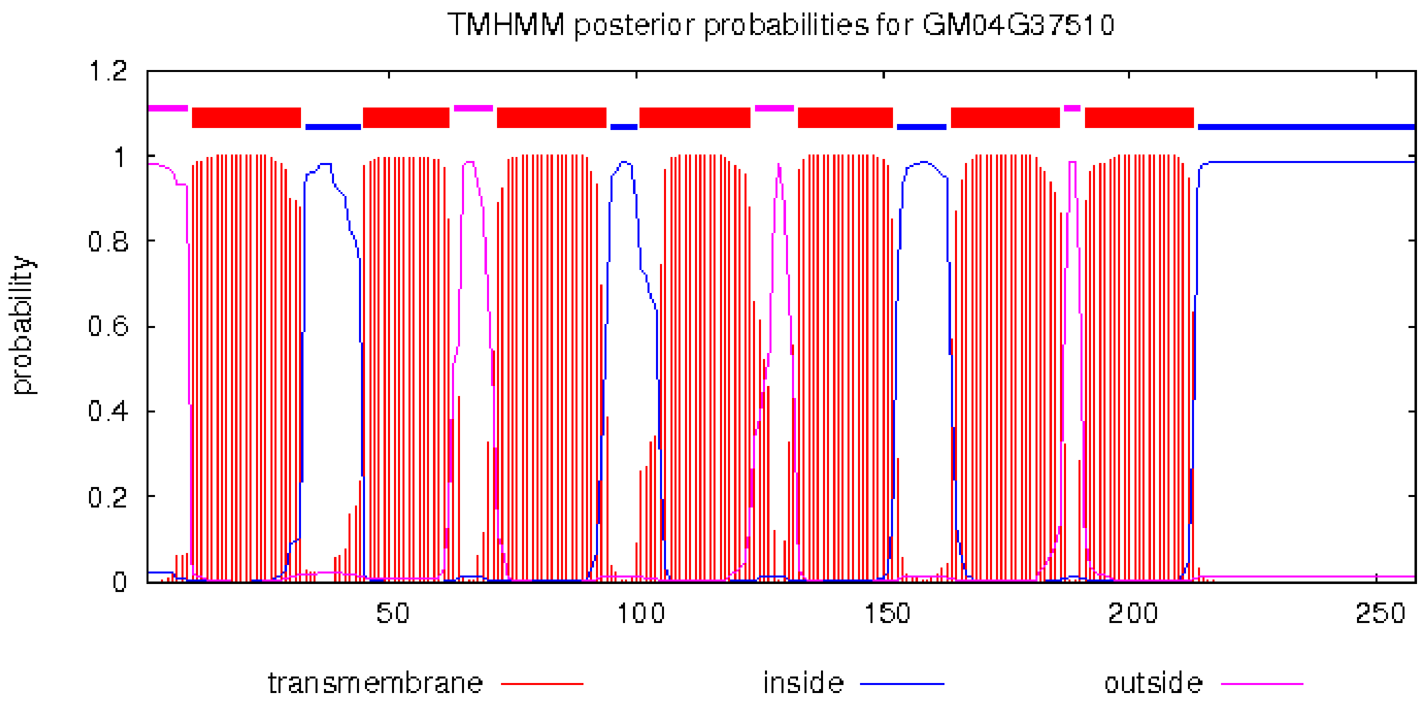

plot in postscript, script for making the plot in gnuplot, data for plot

---

GM04G37520 Length: 282
GM04G37520 Number of predicted TMHs: 7
GM04G37520 Exp number of AAs in TMHs: 153.97121
GM04G37520 Exp number, first 60 AAs: 39.39724
GM04G37520 Total prob of N-in: 0.01799
GM04G37520 POSSIBLE N-term signal sequence

GM04G37520	TMHMM2.0	outside	1	9
GM04G37520	TMHMM2.0	TMhelix	10	32
GM04G37520	TMHMM2.0	inside	33	44
GM04G37520	TMHMM2.0	TMhelix	45	62
GM04G37520	TMHMM2.0	outside	63	71
GM04G37520	TMHMM2.0	TMhelix	72	94
GM04G37520	TMHMM2.0	inside	95	100
GM04G37520	TMHMM2.0	TMhelix	101	123
GM04G37520	TMHMM2.0	outside	124	132
GM04G37520	TMHMM2.0	TMhelix	133	152
GM04G37520	TMHMM2.0	inside	153	163
GM04G37520	TMHMM2.0	TMhelix	164	186
GM04G37520	TMHMM2.0	outside	187	190
GM04G37520	TMHMM2.0	TMhelix	191	213
GM04G37520	TMHMM2.0	inside	214	282

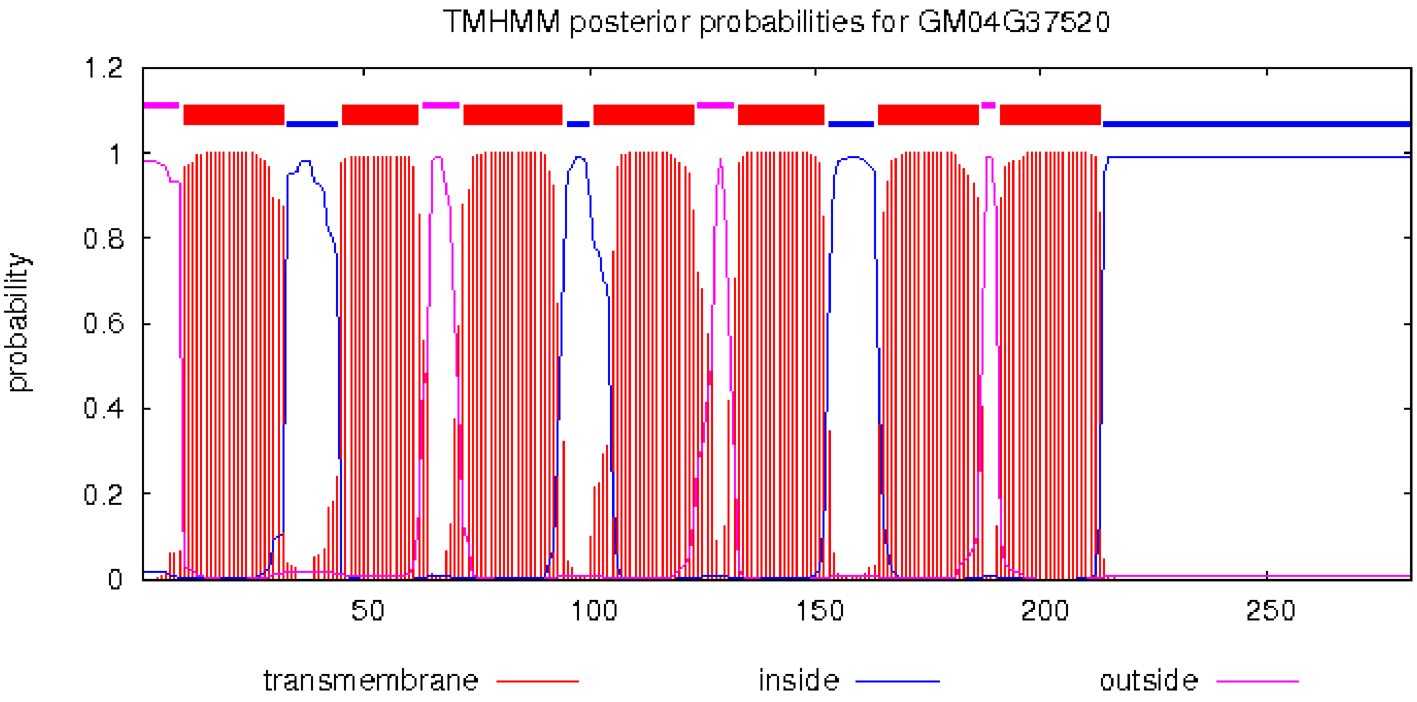

plot in postscript, script for making the plot in gnuplot, data for plot

---

```
# GM04G37530 Length: 287
# GM04G37530 Number of predicted TMHs:  6
# GM04G37530 Exp number of AAs in TMHs: 132.68201
# GM04G37530 Exp number, first 60 AAs:  40.55874
# GM04G37530 Total prob of N-in:        0.87504
# GM04G37530 POSSIBLE N-term signal sequence
GM04G37530      TMHMM2.0        inside       1     6
GM04G37530      TMHMM2.0        TMhelix      7    29
GM04G37530      TMHMM2.0        outside     30    43
GM04G37530      TMHMM2.0        TMhelix     44    63
GM04G37530      TMHMM2.0        inside      64    69
GM04G37530      TMHMM2.0        TMhelix     70    92
GM04G37530      TMHMM2.0        outside     93   101
GM04G37530      TMHMM2.0        TMhelix    102   124
GM04G37530      TMHMM2.0        inside     125   132
GM04G37530      TMHMM2.0        TMhelix    133   155
GM04G37530      TMHMM2.0        outside    156   159
GM04G37530      TMHMM2.0        TMhelix    160   182
GM04G37530      TMHMM2.0        inside     183   287
```

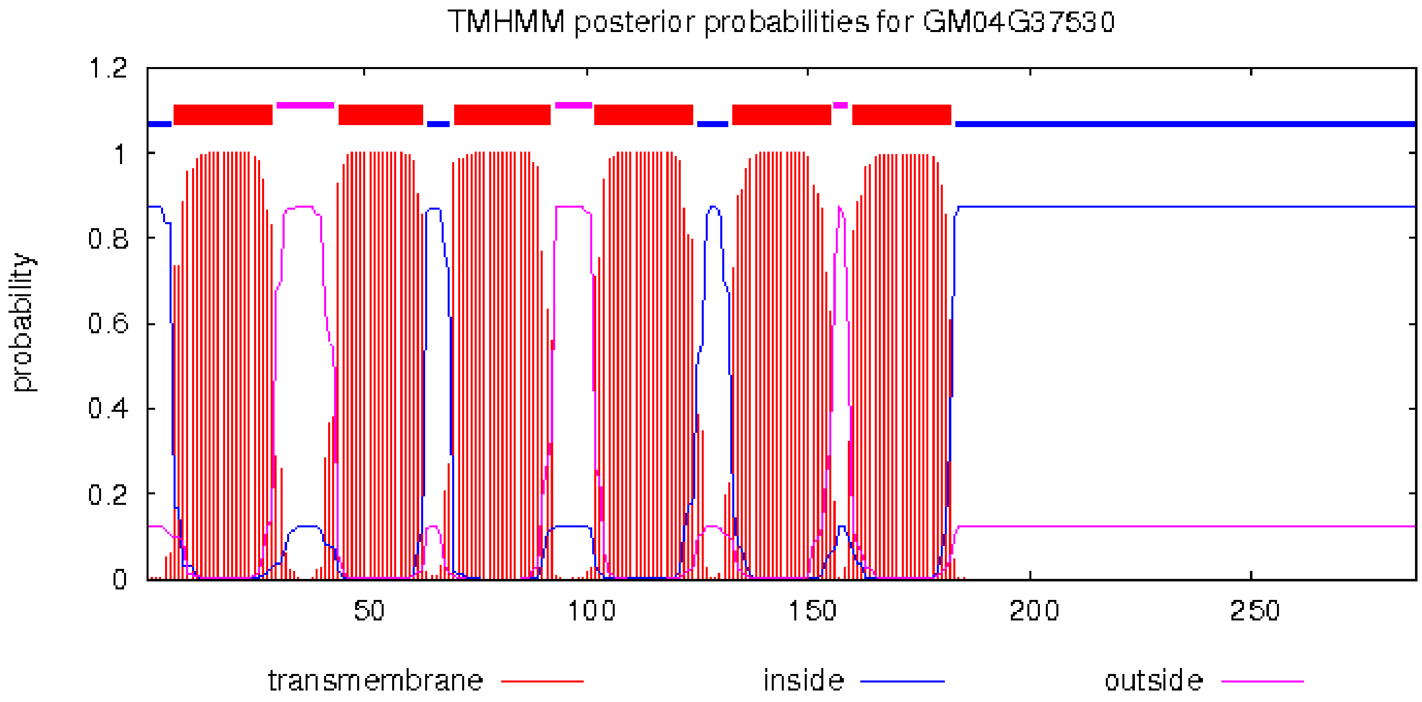

plot in postscript, script for making the plot in gnuplot, data for plot

---

```
# GM04G41680 Length: 174
# GM04G41680 Number of predicted TMHs:  5
# GM04G41680 Exp number of AAs in TMHs: 108.40534
# GM04G41680 Exp number, first 60 AAs:  40.63107
# GM04G41680 Total prob of N-in:        0.50086
# GM04G41680 POSSIBLE N-term signal sequence
GM04G41680      TMHMM2.0        inside       1     6
GM04G41680      TMHMM2.0        TMhelix      7    28
GM04G41680      TMHMM2.0        outside     29    42
GM04G41680      TMHMM2.0        TMhelix     43    65
GM04G41680      TMHMM2.0        inside      66    69
GM04G41680      TMHMM2.0        TMhelix     70    92
GM04G41680      TMHMM2.0        outside     93   101
GM04G41680      TMHMM2.0        TMhelix    102   124
GM04G41680      TMHMM2.0        inside     125   130
GM04G41680      TMHMM2.0        TMhelix    131   153
GM04G41680      TMHMM2.0        outside    154   174
```

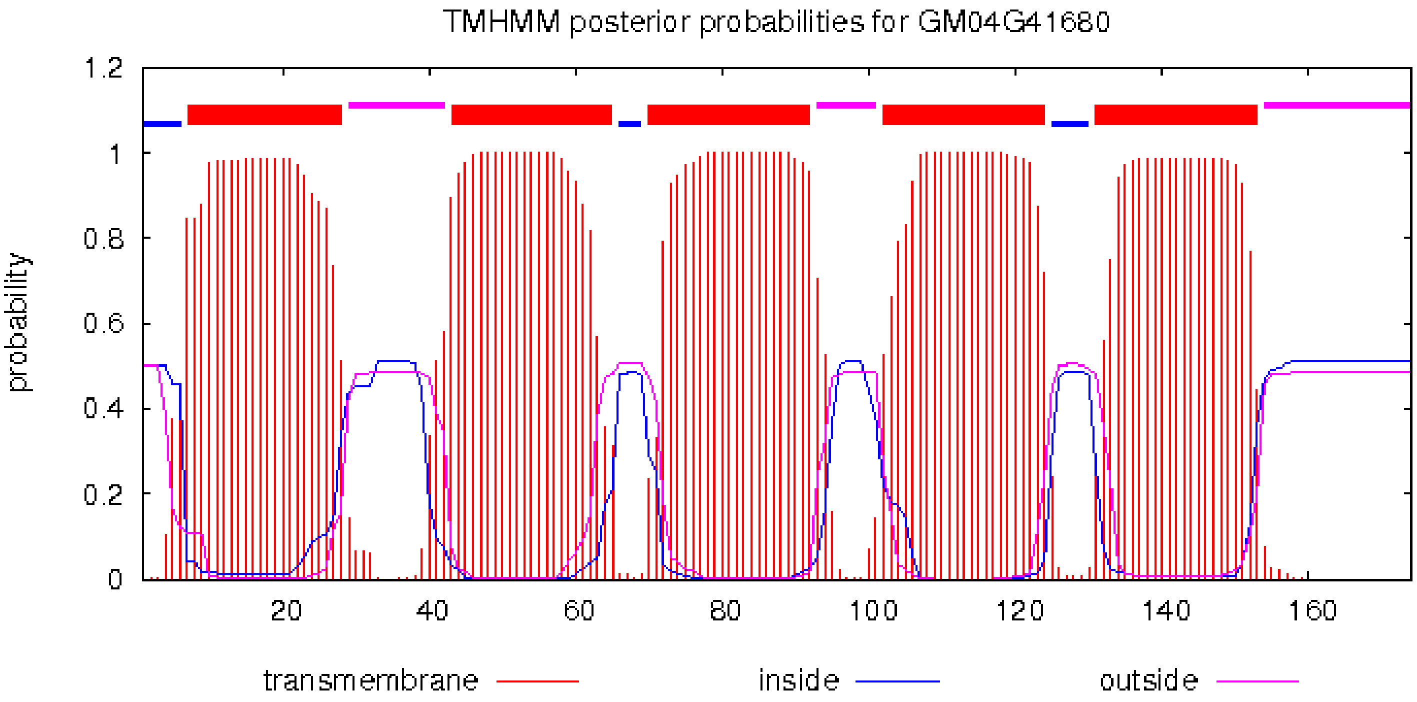

plot in postscript, script for making the plot in gnuplot, data for plot

---

```
# GM04G42040 Length: 247
# GM04G42040 Number of predicted TMHs:  7
# GM04G42040 Exp number of AAs in TMHs: 156.69269
# GM04G42040 Exp number, first 60 AAs:  43.36065
# GM04G42040 Total prob of N-in:        0.00095
# GM04G42040 POSSIBLE N-term signal sequence
GM04G42040      TMHMM2.0        outside      1     4
GM04G42040      TMHMM2.0        TMhelix      5    27
GM04G42040      TMHMM2.0        inside      28    38
GM04G42040      TMHMM2.0        TMhelix     39    58
GM04G42040      TMHMM2.0        outside     59    67
GM04G42040      TMHMM2.0        TMhelix     68    90
GM04G42040      TMHMM2.0        inside      91    98
GM04G42040      TMHMM2.0        TMhelix     99   121
GM04G42040      TMHMM2.0        outside    122   130
GM04G42040      TMHMM2.0        TMhelix    131   153
GM04G42040      TMHMM2.0        inside     154   159
GM04G42040      TMHMM2.0        TMhelix    160   182
GM04G42040      TMHMM2.0        outside    183   186
GM04G42040      TMHMM2.0        TMhelix    187   209
GM04G42040      TMHMM2.0        inside     210   247
```

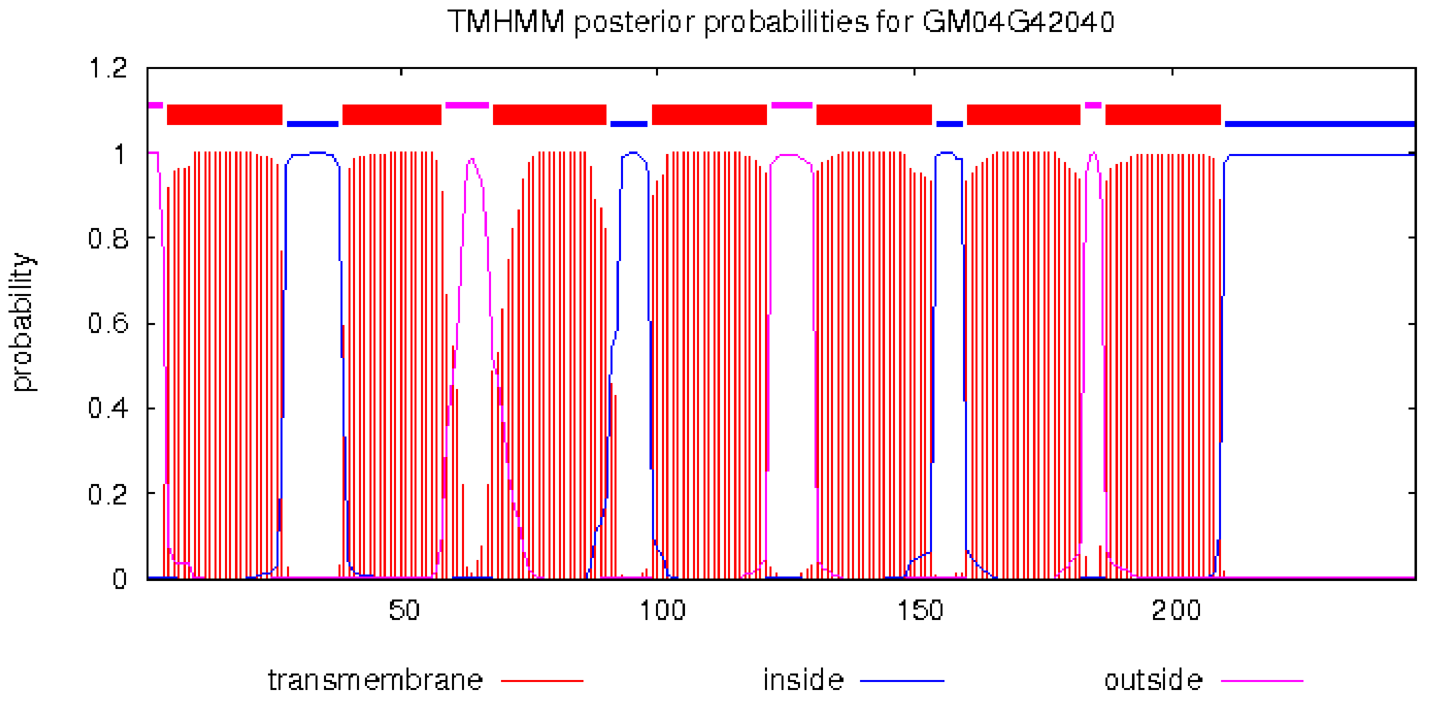

plot in postscript, script for making the plot in gnuplot, data for plot

---

```
# GM05G02070 Length: 226
# GM05G02070 Number of predicted TMHs:  6
# GM05G02070 Exp number of AAs in TMHs: 135.71057
# GM05G02070 Exp number, first 60 AAs:  25.4069
# GM05G02070 Total prob of N-in:        0.84115
# GM05G02070 POSSIBLE N-term signal sequence
GM05G02070      TMHMM2.0        inside       1    11
GM05G02070      TMHMM2.0        TMhelix     12    34
GM05G02070      TMHMM2.0        outside     35    71
GM05G02070      TMHMM2.0        TMhelix     72    94
GM05G02070      TMHMM2.0        inside      95   100
GM05G02070      TMHMM2.0        TMhelix    101   123
GM05G02070      TMHMM2.0        outside    124   132
GM05G02070      TMHMM2.0        TMhelix    133   152
GM05G02070      TMHMM2.0        inside     153   163
GM05G02070      TMHMM2.0        TMhelix    164   186
GM05G02070      TMHMM2.0        outside    187   190
GM05G02070      TMHMM2.0        TMhelix    191   213
GM05G02070      TMHMM2.0        inside     214   226
```

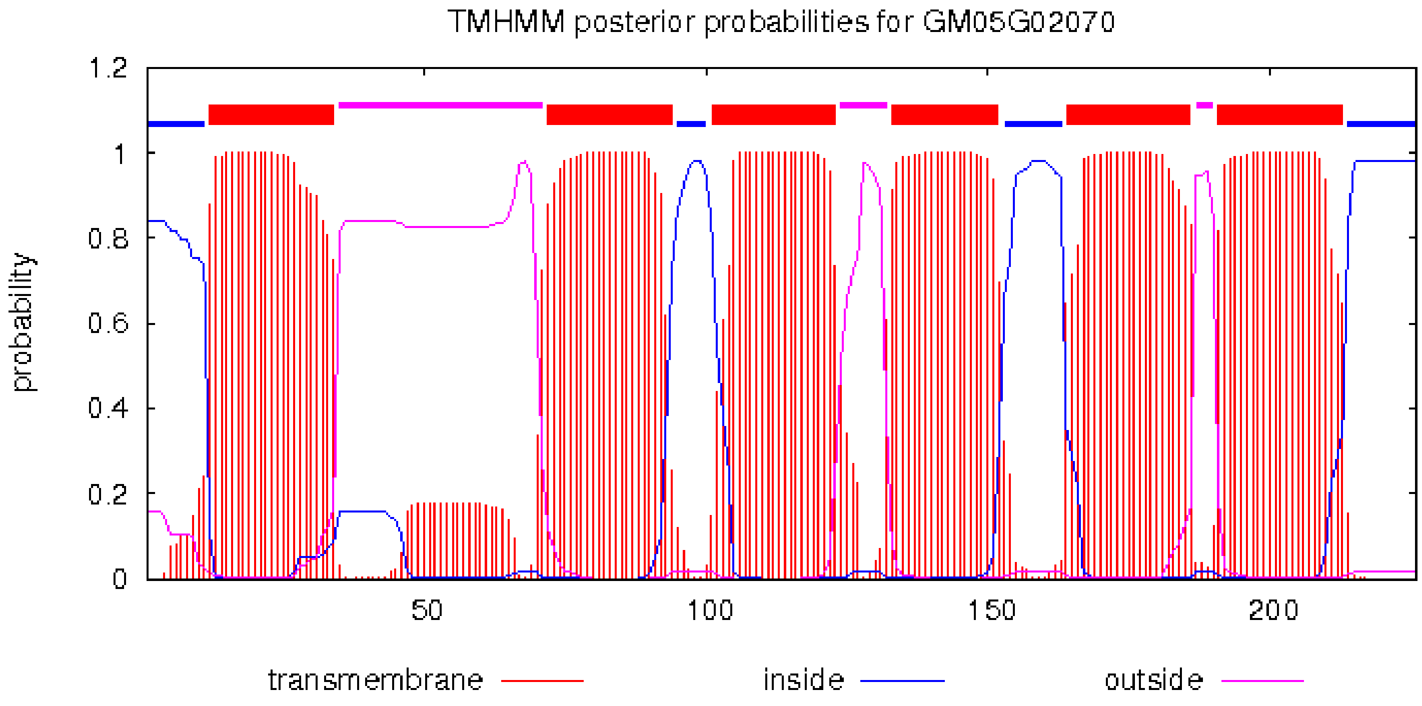

plot in postscript, script for making the plot in gnuplot, data for plot

---

```
# GM05G25180 Length: 283
# GM05G25180 Number of predicted TMHs:  7
# GM05G25180 Exp number of AAs in TMHs: 153.2209
# GM05G25180 Exp number, first 60 AAs:  38.18565
# GM05G25180 Total prob of N-in:        0.00488
# GM05G25180 POSSIBLE N-term signal sequence
GM05G25180      TMHMM2.0        outside      1     14
GM05G25180      TMHMM2.0        TMhelix     15     37
GM05G25180      TMHMM2.0        inside      38     43
GM05G25180      TMHMM2.0        TMhelix     44     63
GM05G25180      TMHMM2.0        outside     64     72
GM05G25180      TMHMM2.0        TMhelix     73     95
GM05G25180      TMHMM2.0        inside      96    106
GM05G25180      TMHMM2.0        TMhelix    107    129
GM05G25180      TMHMM2.0        outside    130    133
GM05G25180      TMHMM2.0        TMhelix    134    156
GM05G25180      TMHMM2.0        inside     157    168
GM05G25180      TMHMM2.0        TMhelix    169    188
GM05G25180      TMHMM2.0        outside    189    191
GM05G25180      TMHMM2.0        TMhelix    192    214
GM05G25180      TMHMM2.0        inside     215    283
```

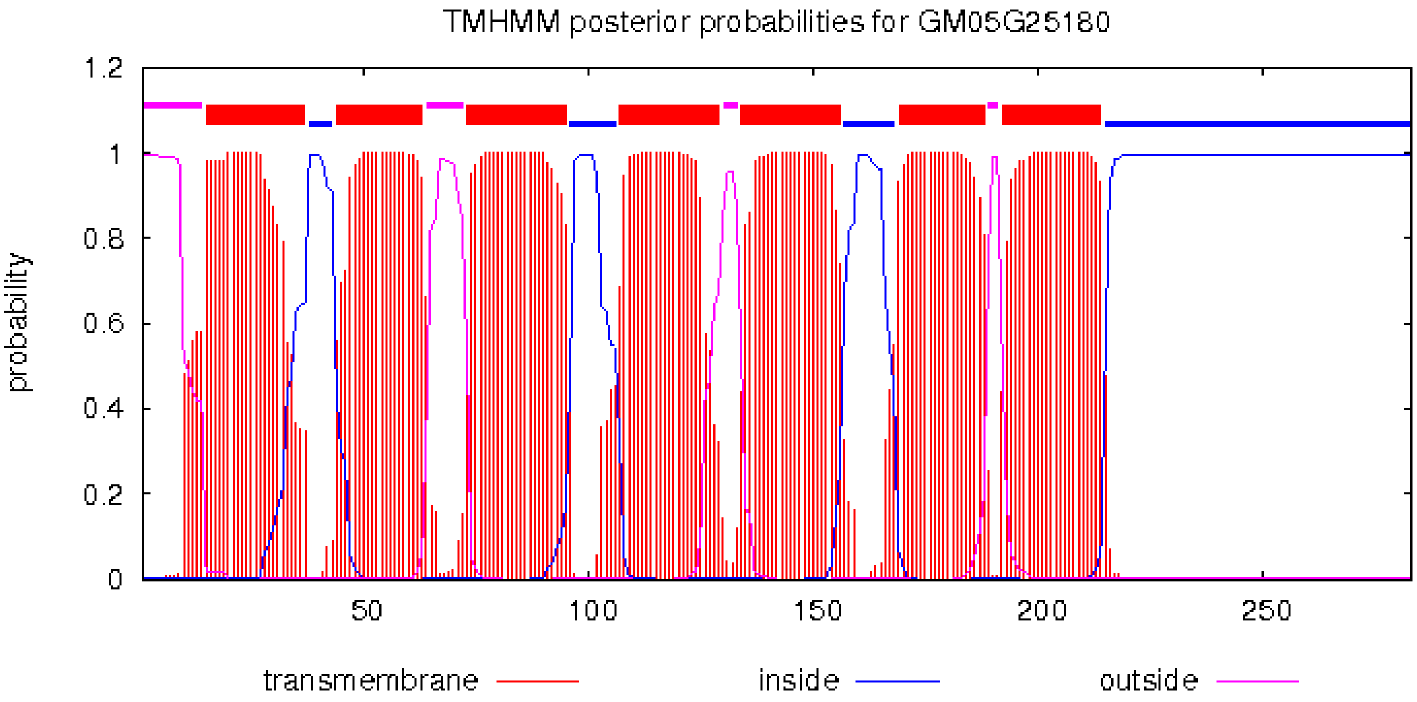

[plot](#) in postscript, [script](#) for making the plot in gnuplot, [data](#) for plot

---

```
# GM05G38340 Length: 257
# GM05G38340 Number of predicted TMHs:  7
# GM05G38340 Exp number of AAs in TMHs: 151.98541
# GM05G38340 Exp number, first 60 AAs:  39.49249
# GM05G38340 Total prob of N-in:        0.87945
# GM05G38340 POSSIBLE N-term signal sequence
GM05G38340      TMHMM2.0        inside       1     6
GM05G38340      TMHMM2.0        TMhelix      7    29
GM05G38340      TMHMM2.0        outside     30    43
GM05G38340      TMHMM2.0        TMhelix     44    63
GM05G38340      TMHMM2.0        inside      64    69
GM05G38340      TMHMM2.0        TMhelix     70    92
GM05G38340      TMHMM2.0        outside     93   101
GM05G38340      TMHMM2.0        TMhelix    102   124
GM05G38340      TMHMM2.0        inside     125   130
GM05G38340      TMHMM2.0        TMhelix    131   153
GM05G38340      TMHMM2.0        outside    154   162
GM05G38340      TMHMM2.0        TMhelix    163   185
GM05G38340      TMHMM2.0        inside     186   189
GM05G38340      TMHMM2.0        TMhelix    190   212
GM05G38340      TMHMM2.0        outside    213   257
```

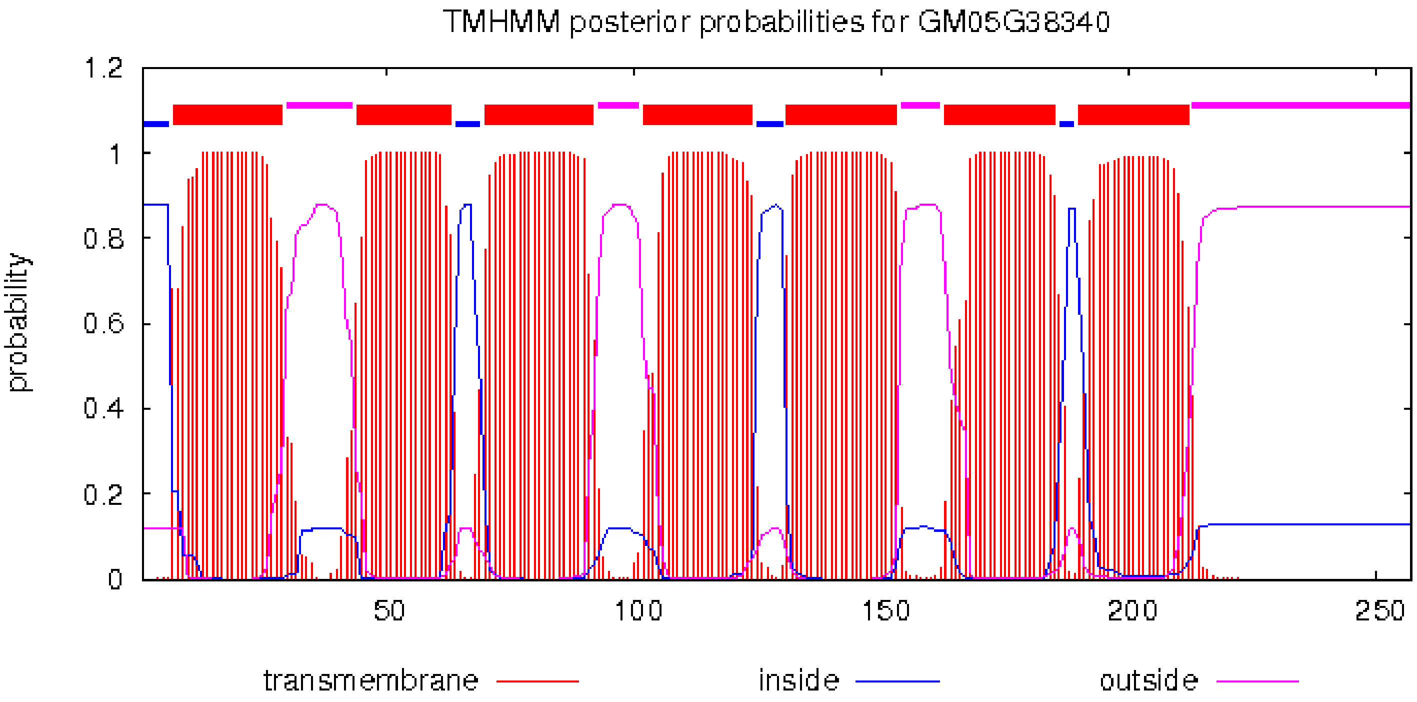

plot in postscript, script for making the plot in gnuplot, data for plot

---

```
# GM05G38351 Length: 236
# GM05G38351 Number of predicted TMHs:  5
# GM05G38351 Exp number of AAs in TMHs: 109.82419
# GM05G38351 Exp number, first 60 AAs:  35.49922
# GM05G38351 Total prob of N-in:        0.00179
# GM05G38351 POSSIBLE N-term signal sequence
GM05G38351      TMHMM2.0        outside      1    14
GM05G38351      TMHMM2.0        TMhelix     15    37
GM05G38351      TMHMM2.0        inside      38    43
GM05G38351      TMHMM2.0        TMhelix     44    66
GM05G38351      TMHMM2.0        outside     67    75
GM05G38351      TMHMM2.0        TMhelix     76    98
GM05G38351      TMHMM2.0        inside      99   109
GM05G38351      TMHMM2.0        TMhelix    110   132
GM05G38351      TMHMM2.0        outside    133   135
GM05G38351      TMHMM2.0        TMhelix    136   158
GM05G38351      TMHMM2.0        inside     159   236
```

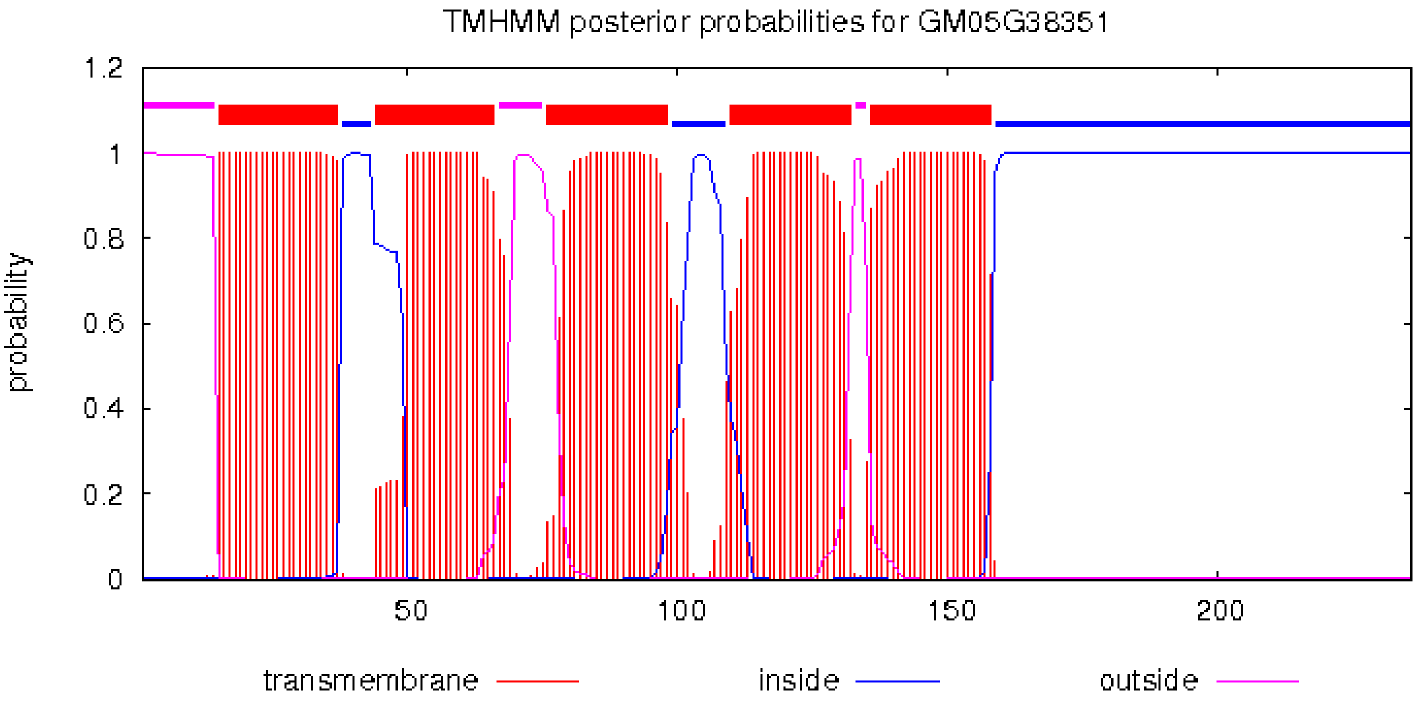

plot in postscript, script for making the plot in gnuplot, data for plot

---

```
# GM06G12740 Length: 258
# GM06G12740 Number of predicted TMHs:  7
# GM06G12740 Exp number of AAs in TMHs: 156.90844
# GM06G12740 Exp number, first 60 AAs:  32.84628
# GM06G12740 Total prob of N-in:        0.00342
# GM06G12740 POSSIBLE N-term signal sequence
GM06G12740      TMHMM2.0        outside      1     9
GM06G12740      TMHMM2.0        TMhelix     10    32
GM06G12740      TMHMM2.0        inside      33    51
GM06G12740      TMHMM2.0        TMhelix     52    74
GM06G12740      TMHMM2.0        outside     75    78
GM06G12740      TMHMM2.0        TMhelix     79   101
GM06G12740      TMHMM2.0        inside     102   109
GM06G12740      TMHMM2.0        TMhelix    110   132
GM06G12740      TMHMM2.0        outside    133   141
GM06G12740      TMHMM2.0        TMhelix    142   164
GM06G12740      TMHMM2.0        inside     165   170
GM06G12740      TMHMM2.0        TMhelix    171   193
GM06G12740      TMHMM2.0        outside    194   197
GM06G12740      TMHMM2.0        TMhelix    198   220
GM06G12740      TMHMM2.0        inside     221   258
```

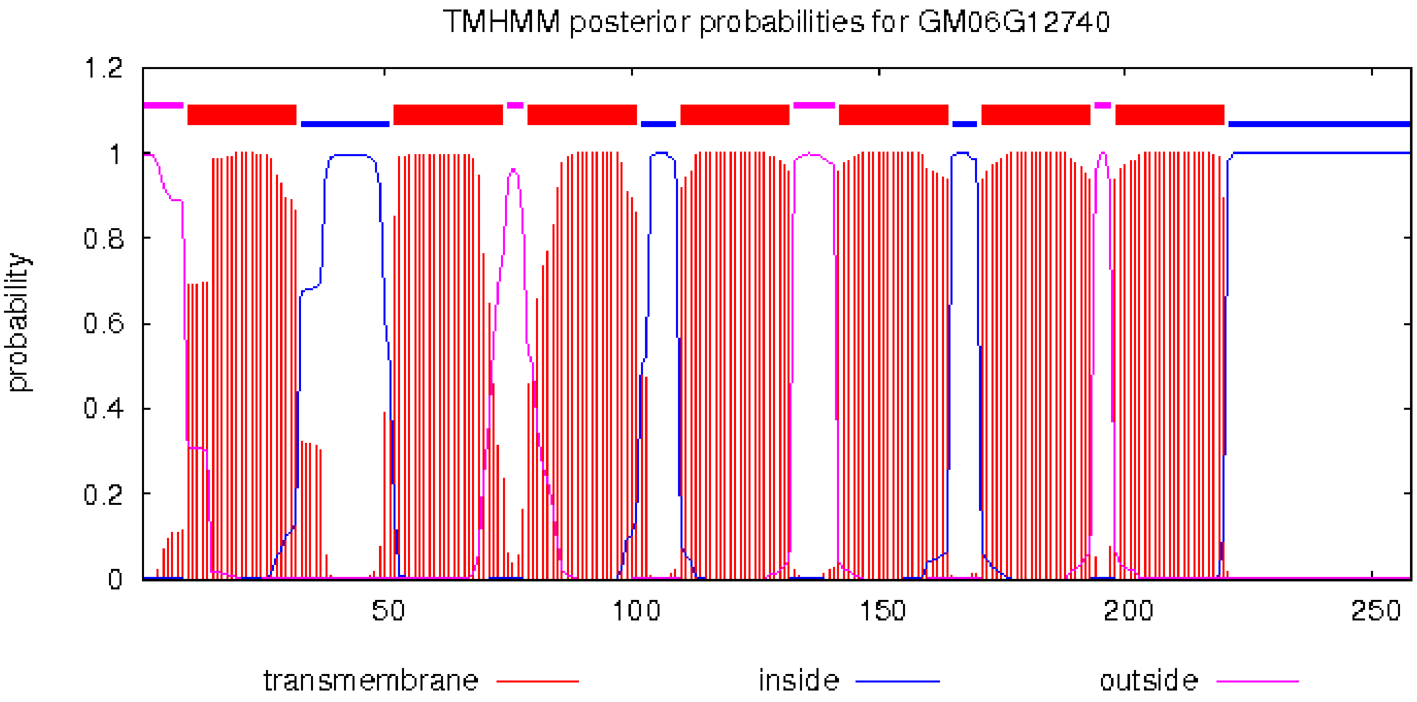

plot in postscript, script for making the plot in gnuplot, data for plot

---

```
# GM06G13110 Length: 254
# GM06G13110 Number of predicted TMHs:  7
# GM06G13110 Exp number of AAs in TMHs: 146.37238
# GM06G13110 Exp number, first 60 AAs:  41.04657
# GM06G13110 Total prob of N-in:        0.34188
# GM06G13110 POSSIBLE N-term signal sequence
GM06G13110      TMHMM2.0        outside      1     4
GM06G13110      TMHMM2.0        TMhelix      5    27
GM06G13110      TMHMM2.0        inside      28    39
GM06G13110      TMHMM2.0        TMhelix     40    62
GM06G13110      TMHMM2.0        outside     63    71
GM06G13110      TMHMM2.0        TMhelix     72    94
GM06G13110      TMHMM2.0        inside      95   100
GM06G13110      TMHMM2.0        TMhelix    101   123
GM06G13110      TMHMM2.0        outside    124   132
GM06G13110      TMHMM2.0        TMhelix    133   152
GM06G13110      TMHMM2.0        inside     153   163
GM06G13110      TMHMM2.0        TMhelix    164   186
GM06G13110      TMHMM2.0        outside    187   189
GM06G13110      TMHMM2.0        TMhelix    190   209
GM06G13110      TMHMM2.0        inside     210   254
```

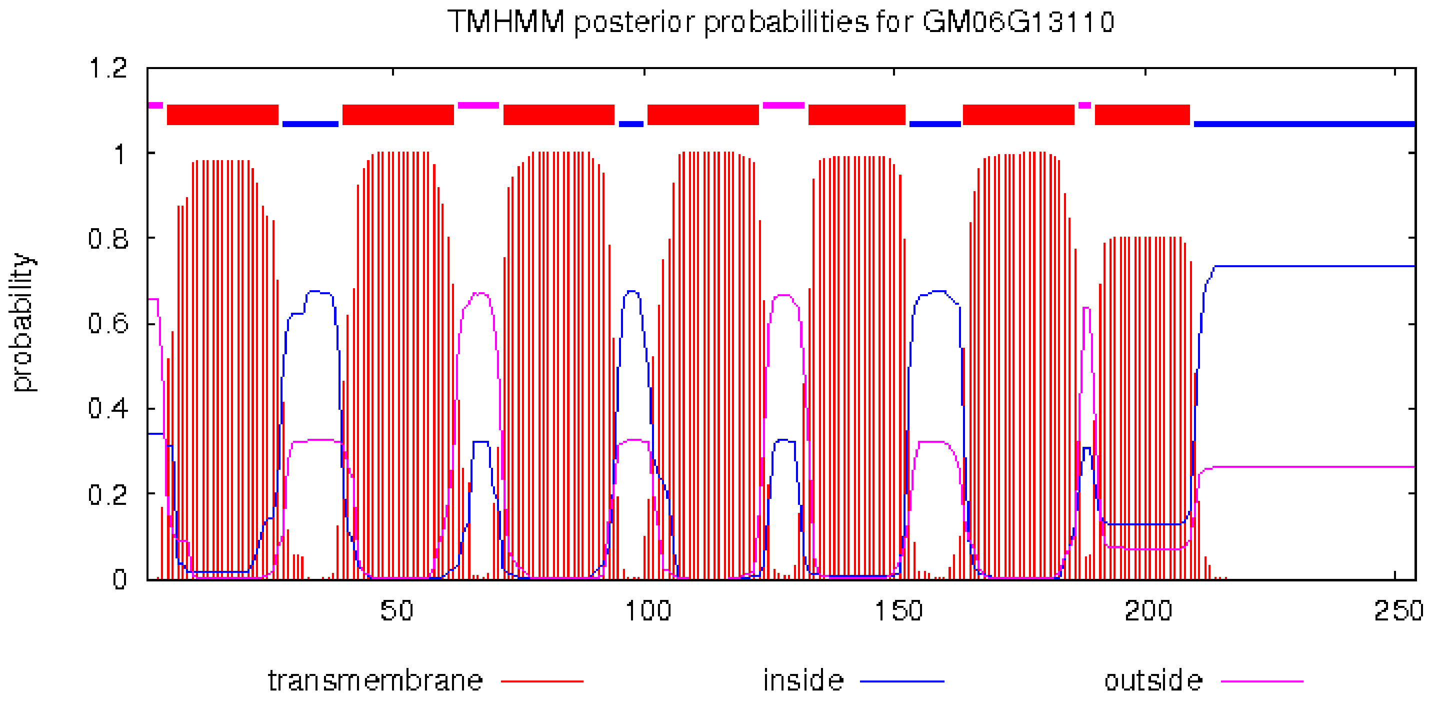

plot in postscript, script for making the plot in gnuplot, data for plot

---

```
# GM06G17520 Length: 309
# GM06G17520 Number of predicted TMHs:  7
# GM06G17520 Exp number of AAs in TMHs: 152.9282
# GM06G17520 Exp number, first 60 AAs:  41.56224
# GM06G17520 Total prob of N-in:        0.01387
# GM06G17520 POSSIBLE N-term signal sequence
GM06G17520      TMHMM2.0        outside      1     4
GM06G17520      TMHMM2.0        TMhelix      5    27
GM06G17520      TMHMM2.0        inside      28    39
GM06G17520      TMHMM2.0        TMhelix     40    62
GM06G17520      TMHMM2.0        outside     63    67
GM06G17520      TMHMM2.0        TMhelix     68    90
GM06G17520      TMHMM2.0        inside      91   102
GM06G17520      TMHMM2.0        TMhelix    103   122
GM06G17520      TMHMM2.0        outside    123   131
GM06G17520      TMHMM2.0        TMhelix    132   154
GM06G17520      TMHMM2.0        inside     155   160
GM06G17520      TMHMM2.0        TMhelix    161   183
GM06G17520      TMHMM2.0        outside    184   187
GM06G17520      TMHMM2.0        TMhelix    188   210
GM06G17520      TMHMM2.0        inside     211   309
```

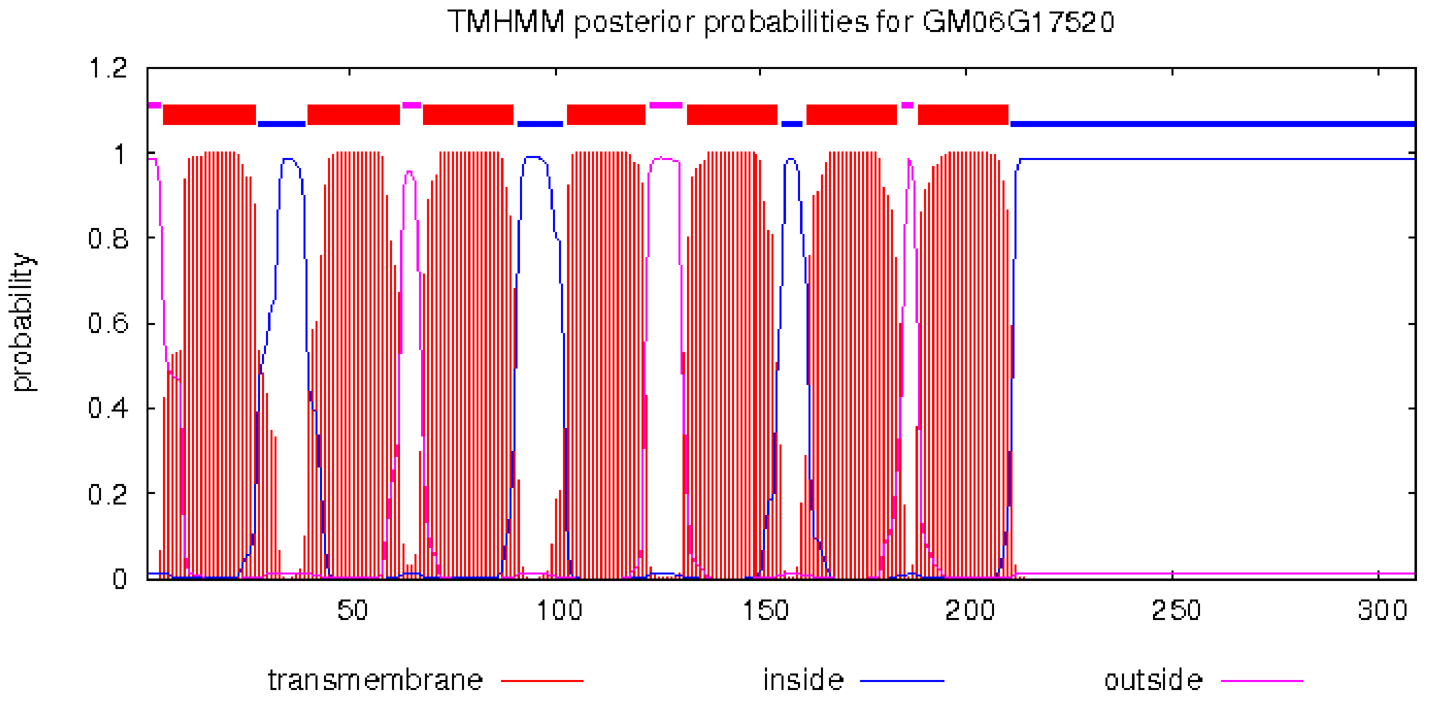

plot in postscript, script for making the plot in gnuplot, data for plot

```
# GM06G17530 Length: 260
# GM06G17530 Number of predicted TMHs:  7
# GM06G17530 Exp number of AAs in TMHs: 153.86303
# GM06G17530 Exp number, first 60 AAs:  38.85656
# GM06G17530 Total prob of N-in:        0.03660
# GM06G17530 POSSIBLE N-term signal sequence
GM06G17530      TMHMM2.0        outside      1     9
GM06G17530      TMHMM2.0        TMhelix     10    32
GM06G17530      TMHMM2.0        inside      33    44
GM06G17530      TMHMM2.0        TMhelix     45    64
GM06G17530      TMHMM2.0        outside     65    69
GM06G17530      TMHMM2.0        TMhelix     70    92
GM06G17530      TMHMM2.0        inside      93   104
GM06G17530      TMHMM2.0        TMhelix    105   127
GM06G17530      TMHMM2.0        outside    128   130
GM06G17530      TMHMM2.0        TMhelix    131   153
GM06G17530      TMHMM2.0        inside     154   164
GM06G17530      TMHMM2.0        TMhelix    165   187
GM06G17530      TMHMM2.0        outside    188   190
GM06G17530      TMHMM2.0        TMhelix    191   213
GM06G17530      TMHMM2.0        inside     214   260
```

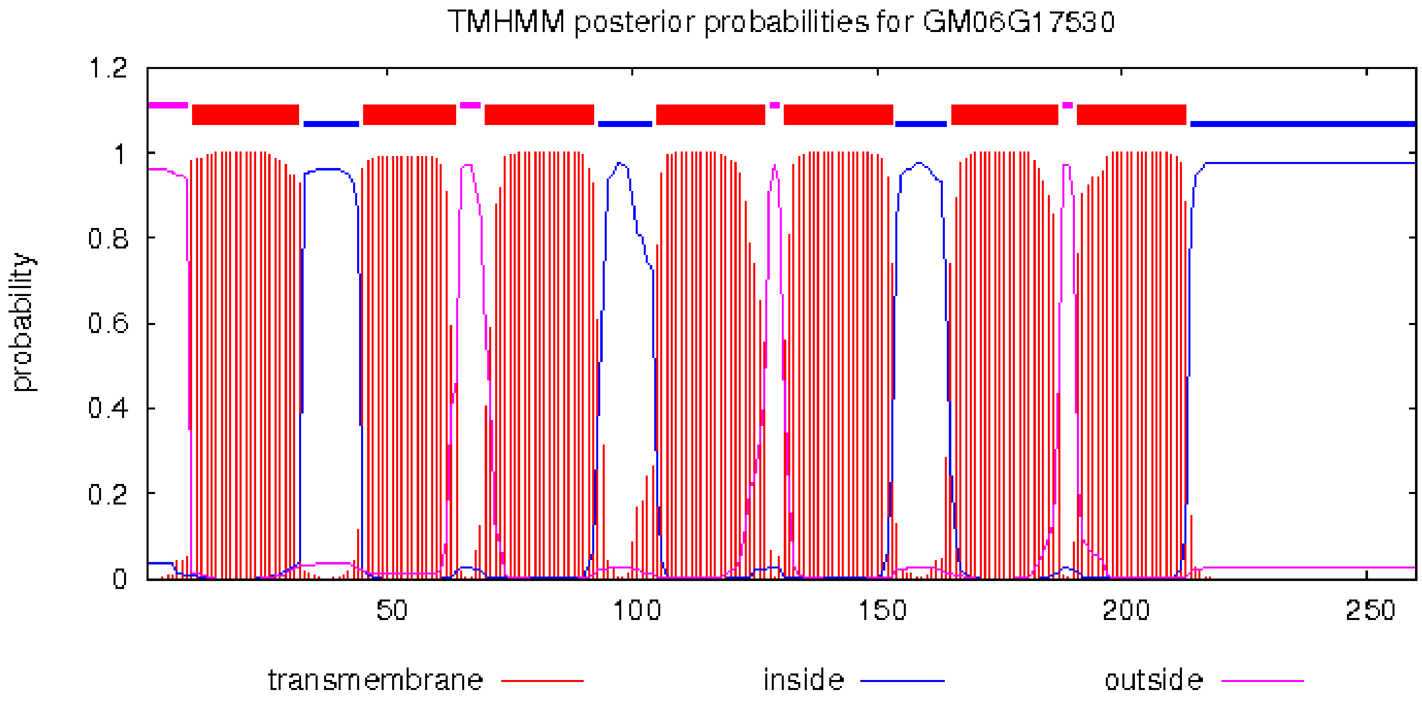

plot in postscript, script for making the plot in gnuplot, data for plot

---

```
# GM06G17540 Length: 258
# GM06G17540 Number of predicted TMHs:  7
# GM06G17540 Exp number of AAs in TMHs: 153.1661
# GM06G17540 Exp number, first 60 AAs:  39.25283
# GM06G17540 Total prob of N-in:        0.06749
# GM06G17540 POSSIBLE N-term signal sequence
GM06G17540      TMHMM2.0        outside      1     9
GM06G17540      TMHMM2.0        TMhelix     10    32
GM06G17540      TMHMM2.0        inside      33    44
GM06G17540      TMHMM2.0        TMhelix     45    62
GM06G17540      TMHMM2.0        outside     63    71
GM06G17540      TMHMM2.0        TMhelix     72    94
GM06G17540      TMHMM2.0        inside      95   100
GM06G17540      TMHMM2.0        TMhelix    101   123
GM06G17540      TMHMM2.0        outside    124   132
GM06G17540      TMHMM2.0        TMhelix    133   152
GM06G17540      TMHMM2.0        inside     153   163
GM06G17540      TMHMM2.0        TMhelix    164   186
GM06G17540      TMHMM2.0        outside    187   190
GM06G17540      TMHMM2.0        TMhelix    191   213
GM06G17540      TMHMM2.0        inside     214   258
```

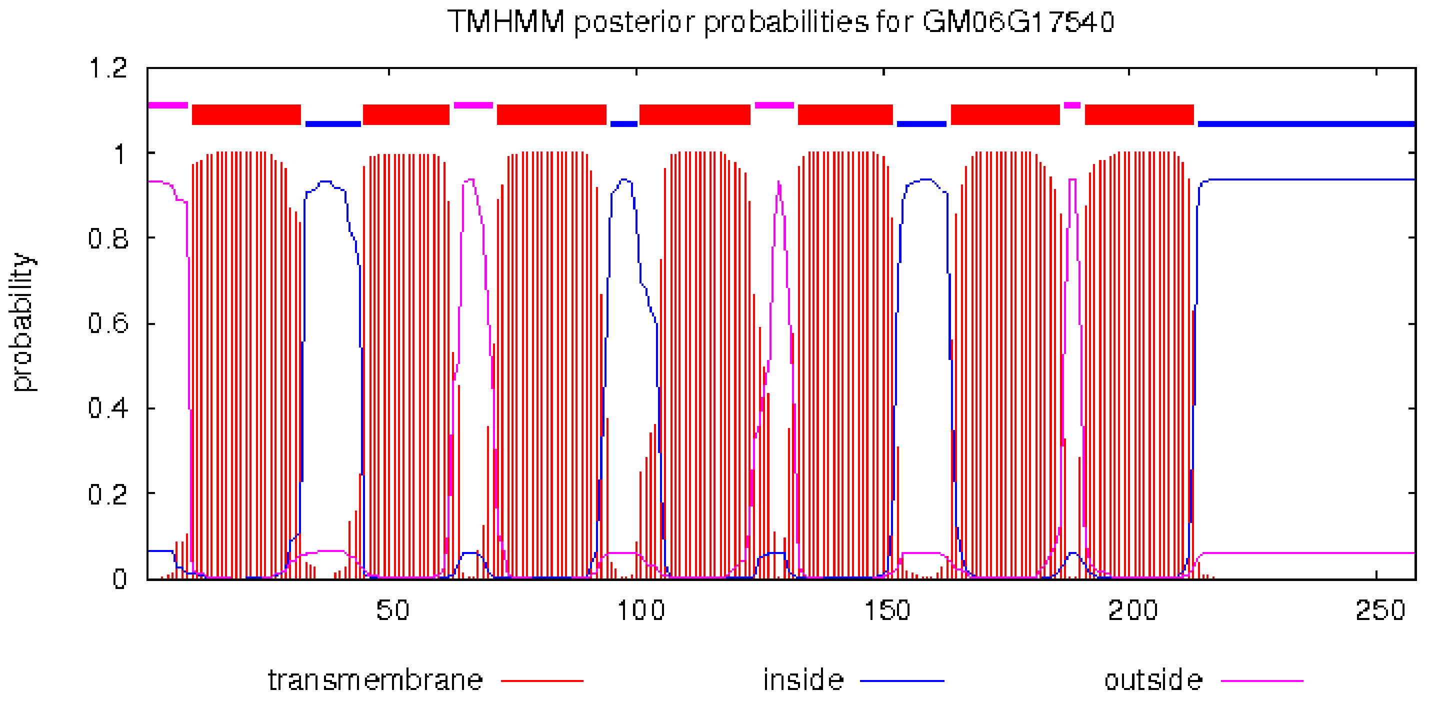

plot in postscript, script for making the plot in gnuplot, data for plot

---

```
# GM06G21572 Length: 149
# GM06G21572 Number of predicted TMHs:  5
# GM06G21572 Exp number of AAs in TMHs: 103.26155
# GM06G21572 Exp number, first 60 AAs:  38.84917
# GM06G21572 Total prob of N-in:        0.17068
# GM06G21572 POSSIBLE N-term signal sequence
GM06G21572      TMHMM2.0        outside      1     3
GM06G21572      TMHMM2.0        TMhelix      4    23
GM06G21572      TMHMM2.0        inside      24    35
GM06G21572      TMHMM2.0        TMhelix     36    55
GM06G21572      TMHMM2.0        outside     56    59
GM06G21572      TMHMM2.0        TMhelix     60    82
GM06G21572      TMHMM2.0        inside      83    94
GM06G21572      TMHMM2.0        TMhelix     95   117
GM06G21572      TMHMM2.0        outside    118   120
GM06G21572      TMHMM2.0        TMhelix    121   143
GM06G21572      TMHMM2.0        inside     144   149
```

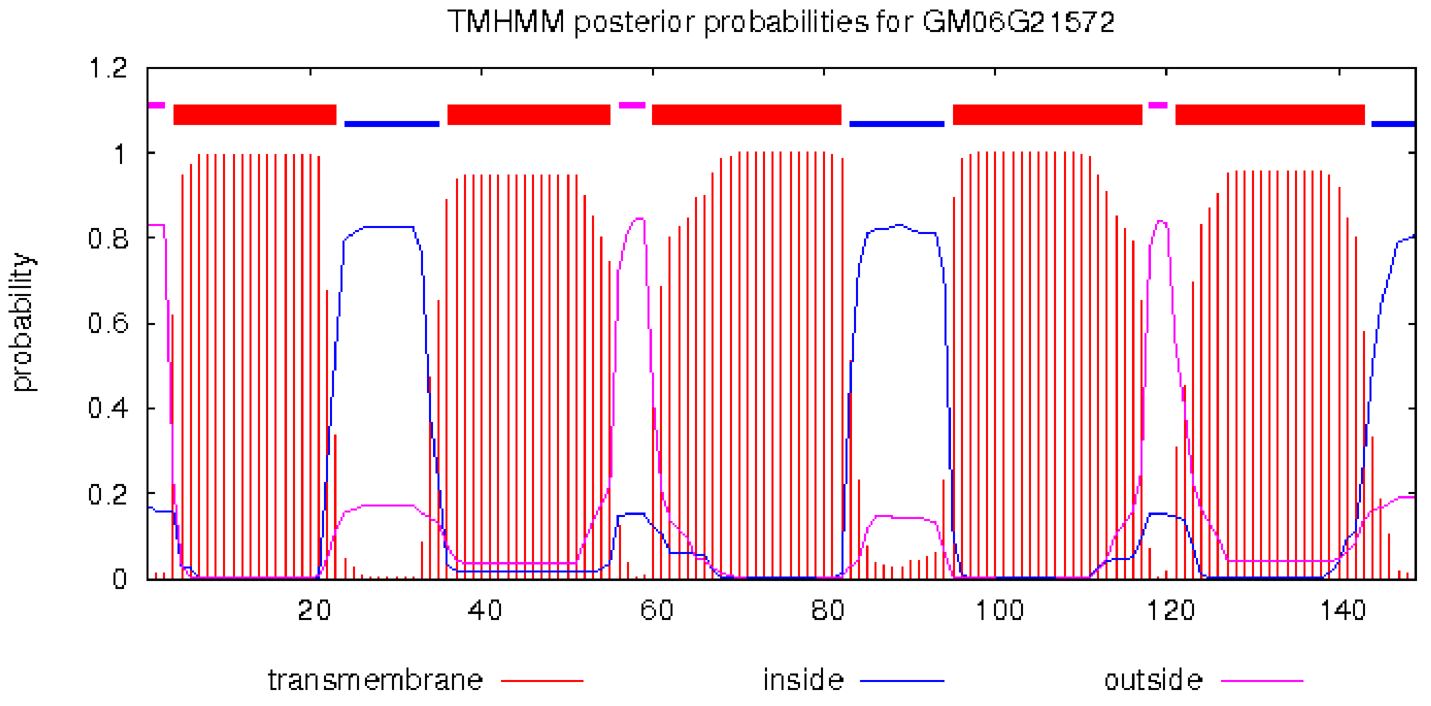

plot in postscript, script for making the plot in gnuplot, data for plot

---

```
# GM06G21665 Length: 184
# GM06G21665 Number of predicted TMHs:  5
# GM06G21665 Exp number of AAs in TMHs: 93.23374
# GM06G21665 Exp number, first 60 AAs:  15.21172
# GM06G21665 Total prob of N-in:        0.28636
# GM06G21665 POSSIBLE N-term signal sequence
GM06G21665      TMHMM2.0        outside      1    41
GM06G21665      TMHMM2.0        TMhelix     42    61
GM06G21665      TMHMM2.0        inside      62    65
GM06G21665      TMHMM2.0        TMhelix     66    88
GM06G21665      TMHMM2.0        outside     89    92
GM06G21665      TMHMM2.0        TMhelix     93   115
GM06G21665      TMHMM2.0        inside     116   135
GM06G21665      TMHMM2.0        TMhelix    136   155
GM06G21665      TMHMM2.0        outside    156   158
GM06G21665      TMHMM2.0        TMhelix    159   181
GM06G21665      TMHMM2.0        inside     182   184
```

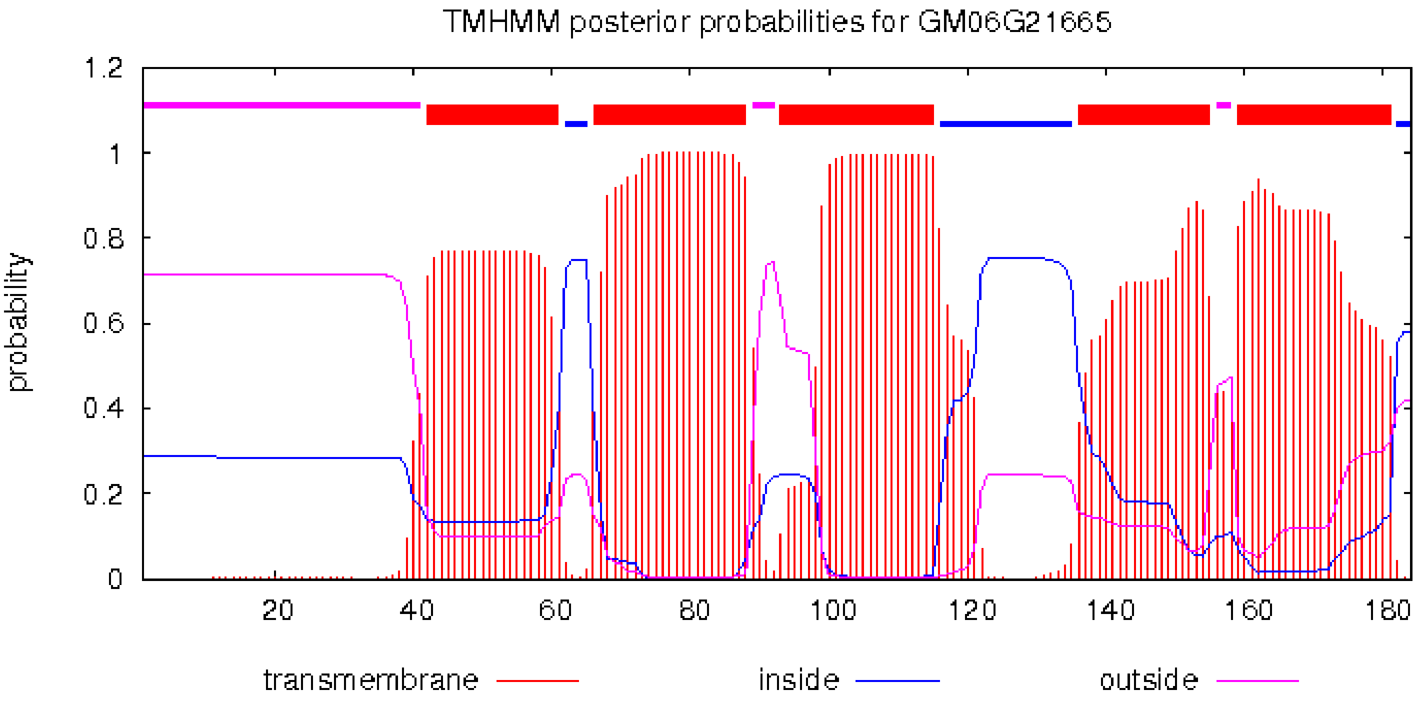

plot in postscript, script for making the plot in gnuplot, data for plot

---

```
# GM08G01300 Length: 294
# GM08G01300 Number of predicted TMHs:  7
# GM08G01300 Exp number of AAs in TMHs: 153.18095
# GM08G01300 Exp number, first 60 AAs:  40.70954
# GM08G01300 Total prob of N-in:        0.05982
# GM08G01300 POSSIBLE N-term signal sequence
GM08G01300      TMHMM2.0        outside      1     5
GM08G01300      TMHMM2.0        TMhelix      6    28
GM08G01300      TMHMM2.0        inside      29    40
GM08G01300      TMHMM2.0        TMhelix     41    63
GM08G01300      TMHMM2.0        outside     64    67
GM08G01300      TMHMM2.0        TMhelix     68    90
GM08G01300      TMHMM2.0        inside      91   102
GM08G01300      TMHMM2.0        TMhelix    103   122
GM08G01300      TMHMM2.0        outside    123   131
GM08G01300      TMHMM2.0        TMhelix    132   154
GM08G01300      TMHMM2.0        inside     155   165
GM08G01300      TMHMM2.0        TMhelix    166   185
GM08G01300      TMHMM2.0        outside    186   189
GM08G01300      TMHMM2.0        TMhelix    190   212
GM08G01300      TMHMM2.0        inside     213   294
```

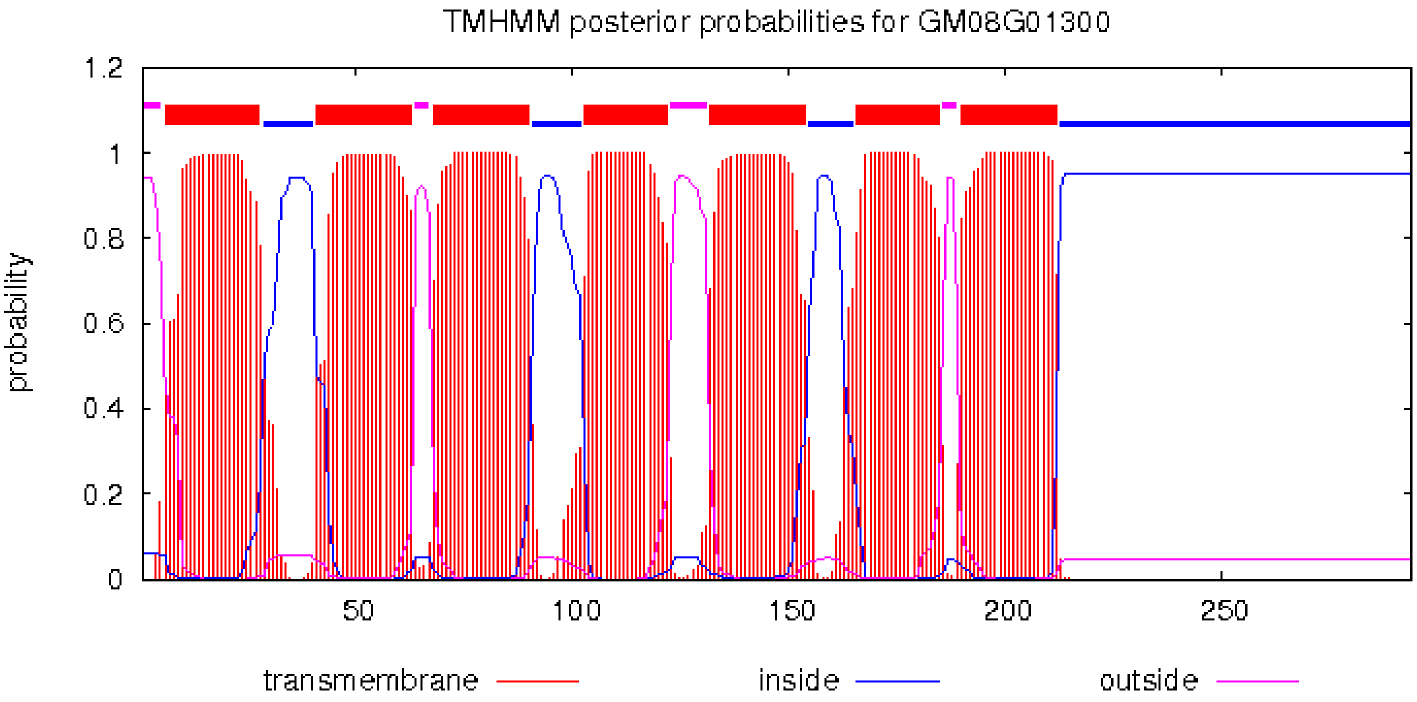

plot in postscript, script for making the plot in gnuplot, data for plot

---

```
# GM08G01310 Length: 254
# GM08G01310 Number of predicted TMHs:  7
# GM08G01310 Exp number of AAs in TMHs: 152.59659
# GM08G01310 Exp number, first 60 AAs:  39.39516
# GM08G01310 Total prob of N-in:        0.86418
# GM08G01310 POSSIBLE N-term signal sequence
GM08G01310      TMHMM2.0        inside       1     6
GM08G01310      TMHMM2.0        TMhelix      7    29
GM08G01310      TMHMM2.0        outside     30    43
GM08G01310      TMHMM2.0        TMhelix     44    63
GM08G01310      TMHMM2.0        inside      64    69
GM08G01310      TMHMM2.0        TMhelix     70    92
GM08G01310      TMHMM2.0        outside     93   101
GM08G01310      TMHMM2.0        TMhelix    102   124
GM08G01310      TMHMM2.0        inside     125   130
GM08G01310      TMHMM2.0        TMhelix    131   153
GM08G01310      TMHMM2.0        outside    154   167
GM08G01310      TMHMM2.0        TMhelix    168   187
GM08G01310      TMHMM2.0        inside     188   191
GM08G01310      TMHMM2.0        TMhelix    192   214
GM08G01310      TMHMM2.0        outside    215   254
```

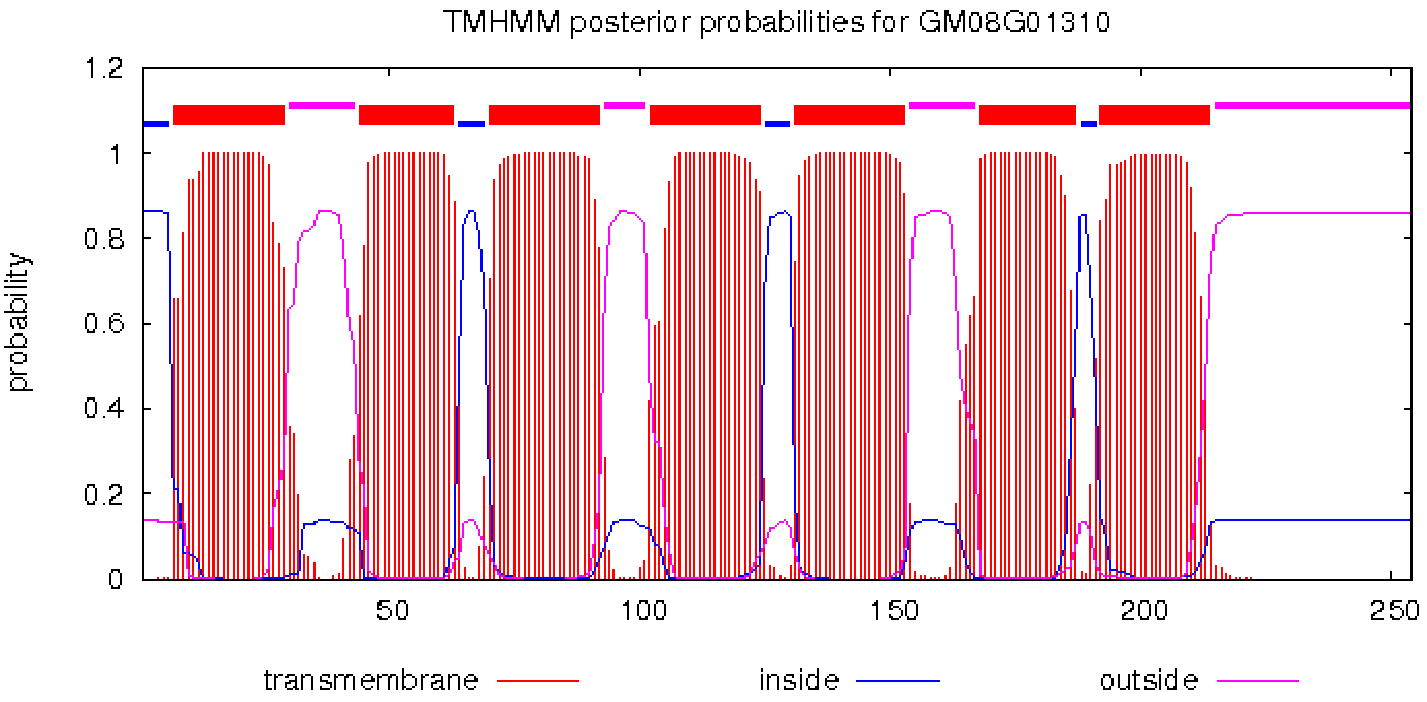

plot in postscript, script for making the plot in gnuplot, data for plot

---

```
# GM08G02890 Length: 247
# GM08G02890 Number of predicted TMHs:  7
# GM08G02890 Exp number of AAs in TMHs: 145.67864
# GM08G02890 Exp number, first 60 AAs:  33.88024
# GM08G02890 Total prob of N-in:        0.44187
# GM08G02890 POSSIBLE N-term signal sequence
GM08G02890      TMHMM2.0        outside      1     9
GM08G02890      TMHMM2.0        TMhelix     10    32
GM08G02890      TMHMM2.0        inside      33    43
GM08G02890      TMHMM2.0        TMhelix     44    66
GM08G02890      TMHMM2.0        outside     67    70
GM08G02890      TMHMM2.0        TMhelix     71    93
GM08G02890      TMHMM2.0        inside      94    99
GM08G02890      TMHMM2.0        TMhelix    100   122
GM08G02890      TMHMM2.0        outside    123   131
GM08G02890      TMHMM2.0        TMhelix    132   151
GM08G02890      TMHMM2.0        inside     152   163
GM08G02890      TMHMM2.0        TMhelix    164   186
GM08G02890      TMHMM2.0        outside    187   190
GM08G02890      TMHMM2.0        TMhelix    191   213
GM08G02890      TMHMM2.0        inside     214   247
```

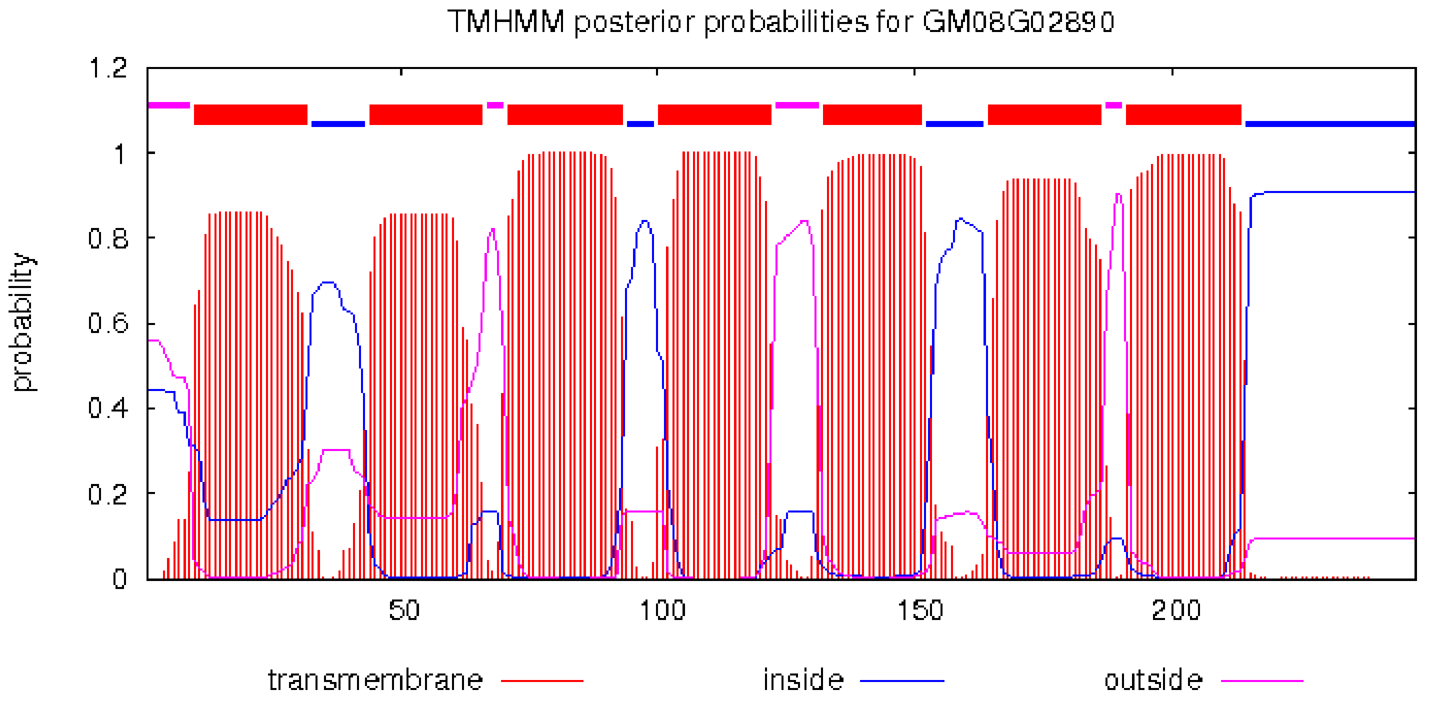

plot in postscript, script for making the plot in gnuplot, data for plot

---

```
# GM08G08200 Length: 274
# GM08G08200 Number of predicted TMHs:  7
# GM08G08200 Exp number of AAs in TMHs: 153.05348
# GM08G08200 Exp number, first 60 AAs:  38.80847
# GM08G08200 Total prob of N-in:        0.01384
# GM08G08200 POSSIBLE N-term signal sequence
GM08G08200      TMHMM2.0        outside      1     9
GM08G08200      TMHMM2.0        TMhelix     10    32
GM08G08200      TMHMM2.0        inside      33    43
GM08G08200      TMHMM2.0        TMhelix     44    63
GM08G08200      TMHMM2.0        outside     64    72
GM08G08200      TMHMM2.0        TMhelix     73    95
GM08G08200      TMHMM2.0        inside      96   106
GM08G08200      TMHMM2.0        TMhelix    107   126
GM08G08200      TMHMM2.0        outside    127   135
GM08G08200      TMHMM2.0        TMhelix    136   158
GM08G08200      TMHMM2.0        inside     159   164
GM08G08200      TMHMM2.0        TMhelix    165   187
GM08G08200      TMHMM2.0        outside    188   190
GM08G08200      TMHMM2.0        TMhelix    191   213
GM08G08200      TMHMM2.0        inside     214   274
```

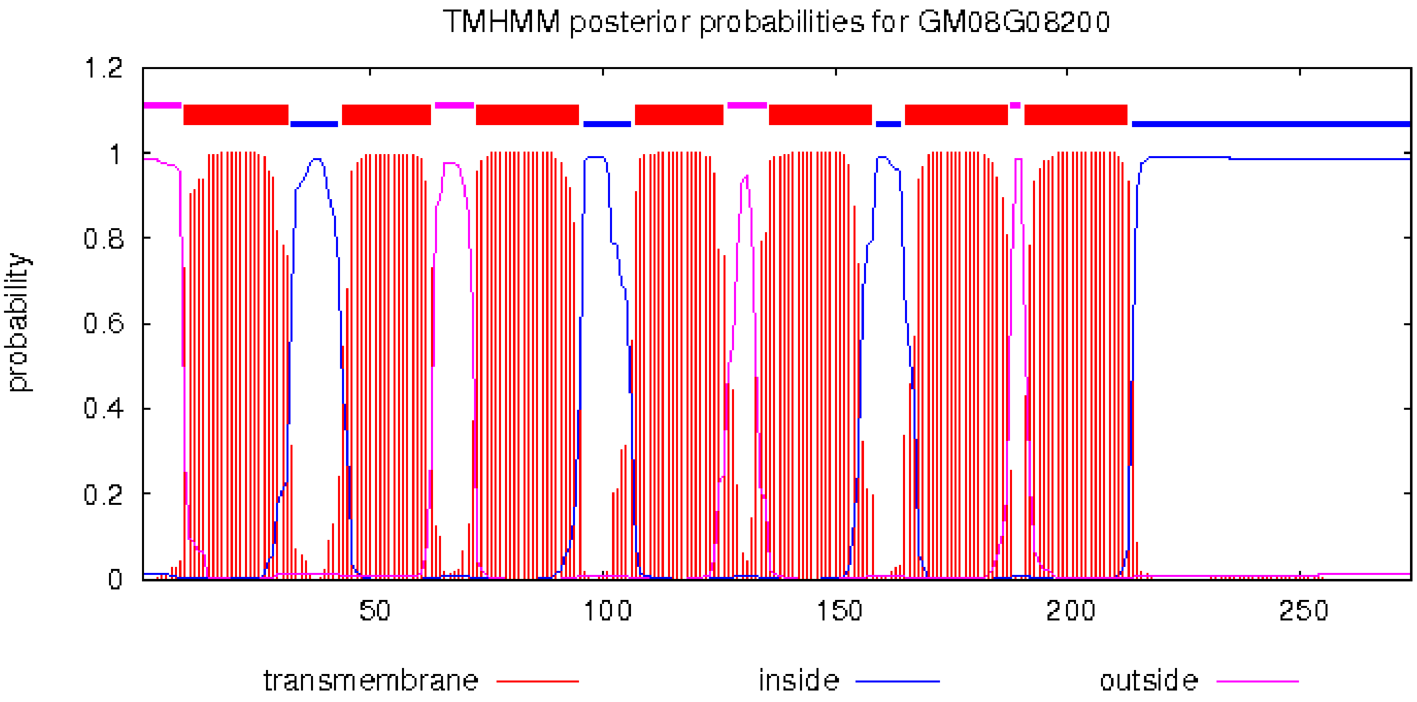

plot in postscript, script for making the plot in gnuplot, data for plot

---

```
# GM08G19580 Length: 280
# GM08G19580 Number of predicted TMHs:  7
# GM08G19580 Exp number of AAs in TMHs: 150.84644
# GM08G19580 Exp number, first 60 AAs:  40.74663
# GM08G19580 Total prob of N-in:        0.44491
# GM08G19580 POSSIBLE N-term signal sequence
GM08G19580      TMHMM2.0        outside      1     5
GM08G19580      TMHMM2.0        TMhelix      6    28
GM08G19580      TMHMM2.0        inside      29    40
GM08G19580      TMHMM2.0        TMhelix     41    63
GM08G19580      TMHMM2.0        outside     64    67
GM08G19580      TMHMM2.0        TMhelix     68    90
GM08G19580      TMHMM2.0        inside      91   101
GM08G19580      TMHMM2.0        TMhelix    102   124
GM08G19580      TMHMM2.0        outside    125   133
GM08G19580      TMHMM2.0        TMhelix    134   156
GM08G19580      TMHMM2.0        inside     157   162
GM08G19580      TMHMM2.0        TMhelix    163   185
GM08G19580      TMHMM2.0        outside    186   189
GM08G19580      TMHMM2.0        TMhelix    190   212
GM08G19580      TMHMM2.0        inside     213   280
```

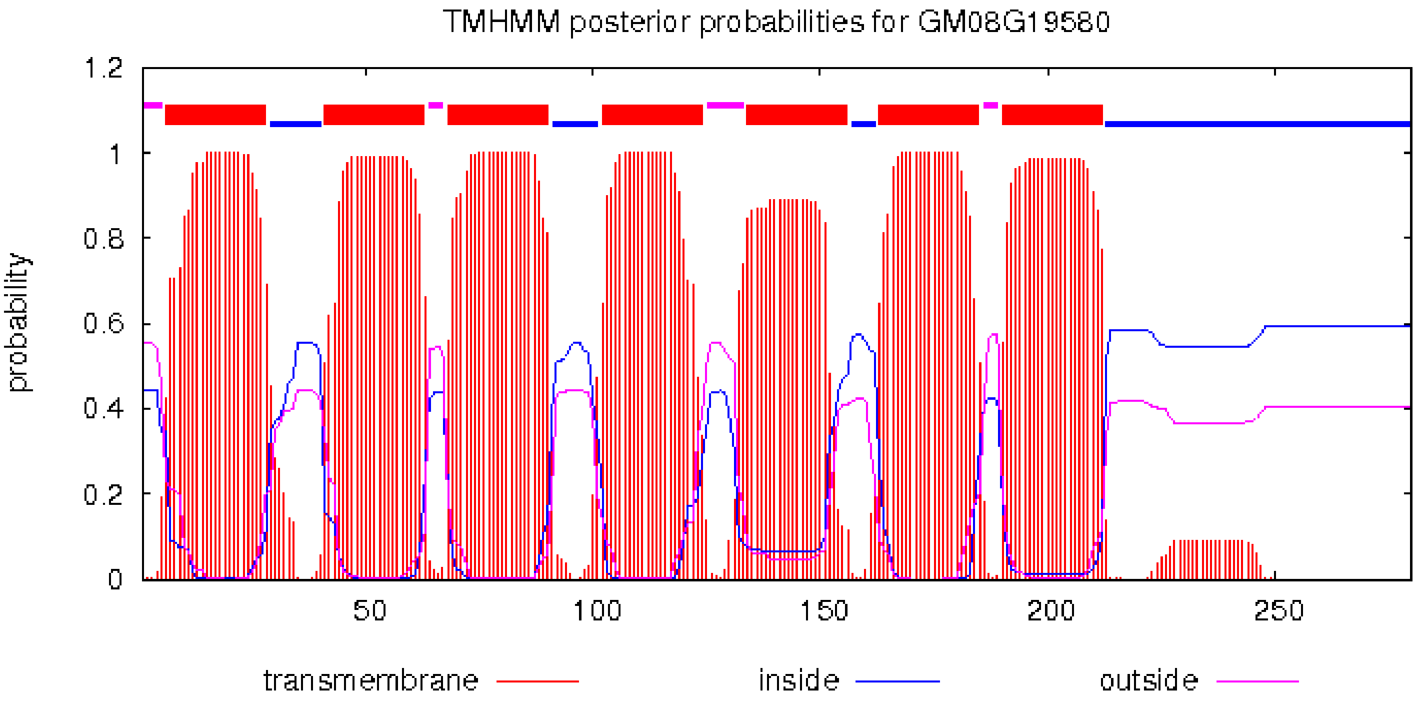

plot in postscript, script for making the plot in gnuplot, data for plot

---

```
# GM08G47550 Length: 272
# GM08G47550 Number of predicted TMHs:  7
# GM08G47550 Exp number of AAs in TMHs: 154.14632
# GM08G47550 Exp number, first 60 AAs:  39.01664
# GM08G47550 Total prob of N-in:        0.00524
# GM08G47550 POSSIBLE N-term signal sequence
GM08G47550      TMHMM2.0        outside      1     9
GM08G47550      TMHMM2.0        TMhelix     10    32
GM08G47550      TMHMM2.0        inside      33    44
GM08G47550      TMHMM2.0        TMhelix     45    64
GM08G47550      TMHMM2.0        outside     65    67
GM08G47550      TMHMM2.0        TMhelix     68    90
GM08G47550      TMHMM2.0        inside      91   101
GM08G47550      TMHMM2.0        TMhelix    102   124
GM08G47550      TMHMM2.0        outside    125   133
GM08G47550      TMHMM2.0        TMhelix    134   156
GM08G47550      TMHMM2.0        inside     157   162
GM08G47550      TMHMM2.0        TMhelix    163   185
GM08G47550      TMHMM2.0        outside    186   188
GM08G47550      TMHMM2.0        TMhelix    189   211
GM08G47550      TMHMM2.0        inside     212   272
```

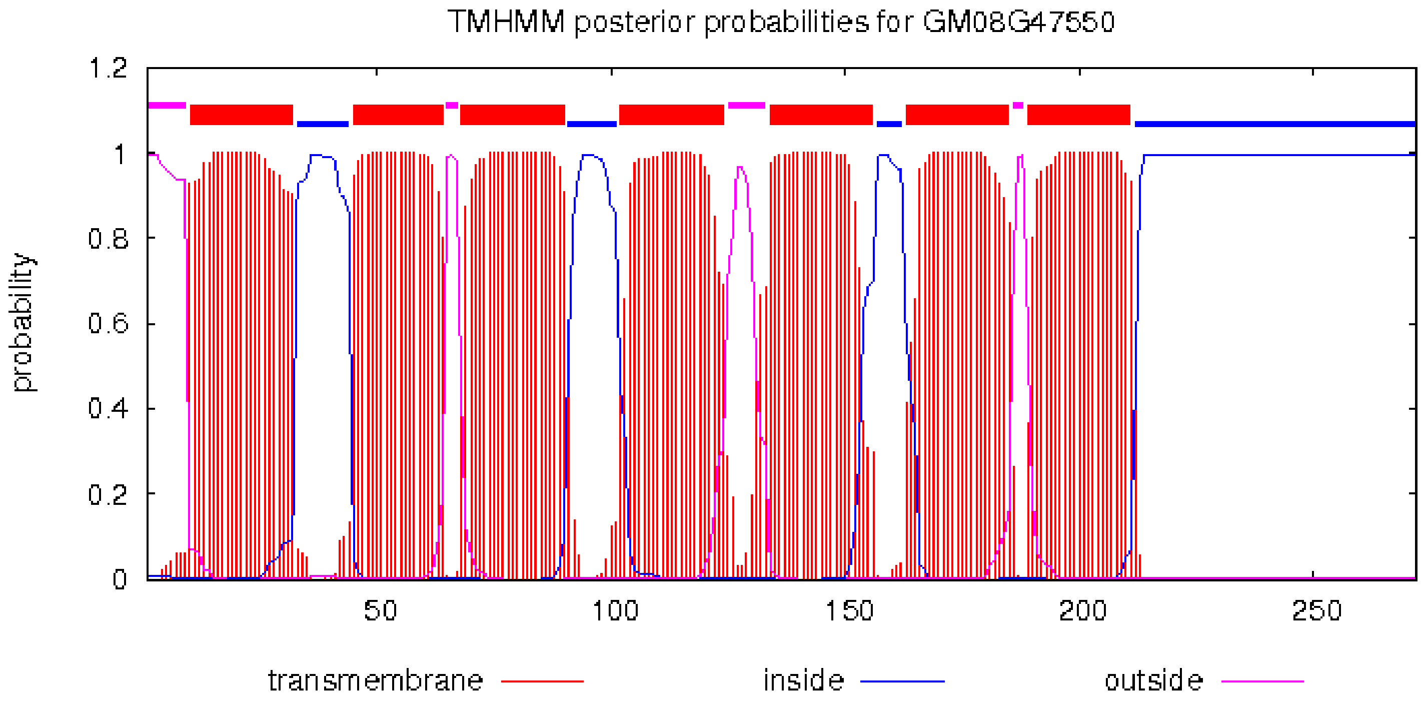

plot in postscript, script for making the plot in gnuplot, data for plot

---

```
# GM08G47561 Length: 273
# GM08G47561 Number of predicted TMHs:  7
# GM08G47561 Exp number of AAs in TMHs: 154.61518
# GM08G47561 Exp number, first 60 AAs:  39.43463
# GM08G47561 Total prob of N-in:        0.04448
# GM08G47561 POSSIBLE N-term signal sequence
GM08G47561      TMHMM2.0        outside      1     9
GM08G47561      TMHMM2.0        TMhelix     10    32
GM08G47561      TMHMM2.0        inside      33    44
GM08G47561      TMHMM2.0        TMhelix     45    64
GM08G47561      TMHMM2.0        outside     65    67
GM08G47561      TMHMM2.0        TMhelix     68    90
GM08G47561      TMHMM2.0        inside      91   101
GM08G47561      TMHMM2.0        TMhelix    102   124
GM08G47561      TMHMM2.0        outside    125   133
GM08G47561      TMHMM2.0        TMhelix    134   156
GM08G47561      TMHMM2.0        inside     157   162
GM08G47561      TMHMM2.0        TMhelix    163   185
GM08G47561      TMHMM2.0        outside    186   188
GM08G47561      TMHMM2.0        TMhelix    189   211
GM08G47561      TMHMM2.0        inside     212   273
```

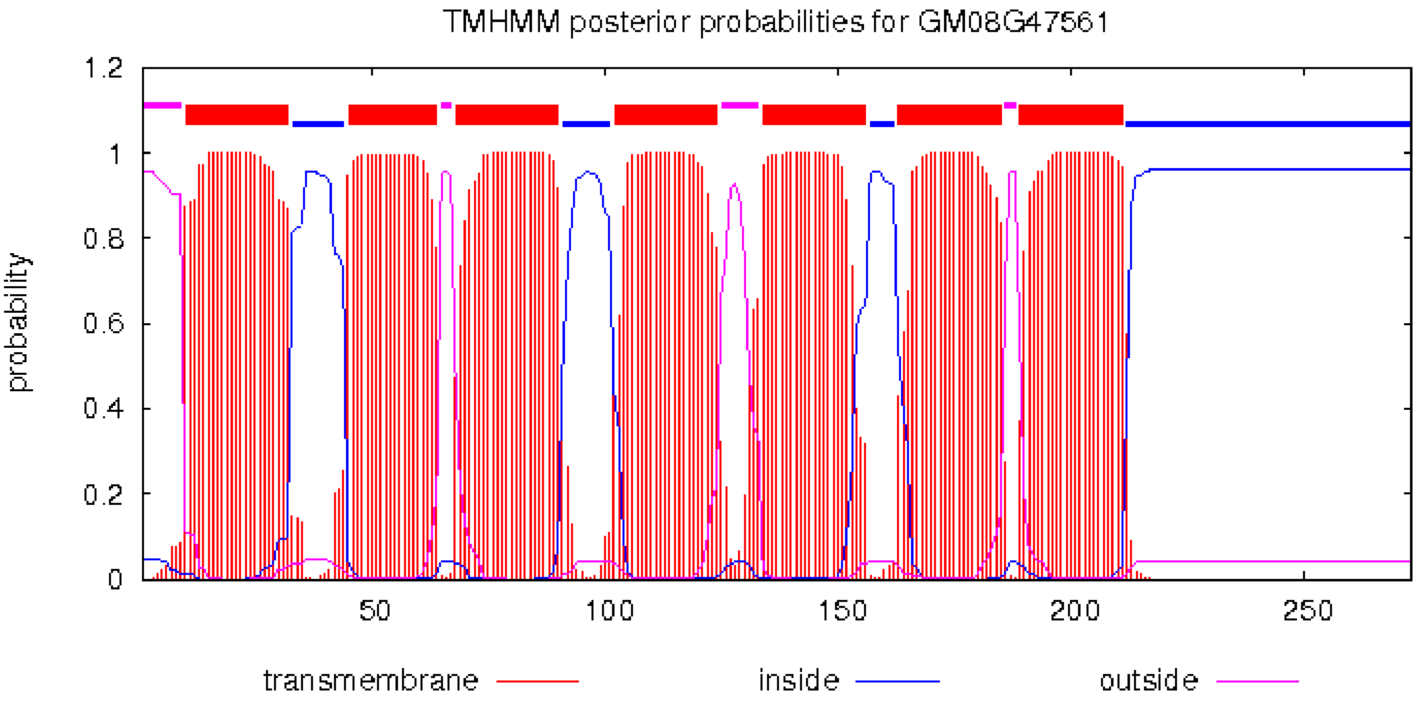

plot in postscript, script for making the plot in gnuplot, data for plot

---

```
# GM08G48281 Length: 223
# GM08G48281 Number of predicted TMHs:  6
# GM08G48281 Exp number of AAs in TMHs: 129.13225
# GM08G48281 Exp number, first 60 AAs:  43.78571
# GM08G48281 Total prob of N-in:        0.94860
# GM08G48281 POSSIBLE N-term signal sequence
GM08G48281      TMHMM2.0        inside       1     1
GM08G48281      TMHMM2.0        TMhelix      2    21
GM08G48281      TMHMM2.0        outside     22    24
GM08G48281      TMHMM2.0        TMhelix     25    47
GM08G48281      TMHMM2.0        inside      48    58
GM08G48281      TMHMM2.0        TMhelix     59    81
GM08G48281      TMHMM2.0        outside     82    90
GM08G48281      TMHMM2.0        TMhelix     91   108
GM08G48281      TMHMM2.0        inside     109   120
GM08G48281      TMHMM2.0        TMhelix    121   143
GM08G48281      TMHMM2.0        outside    144   147
GM08G48281      TMHMM2.0        TMhelix    148   170
GM08G48281      TMHMM2.0        inside     171   223
```

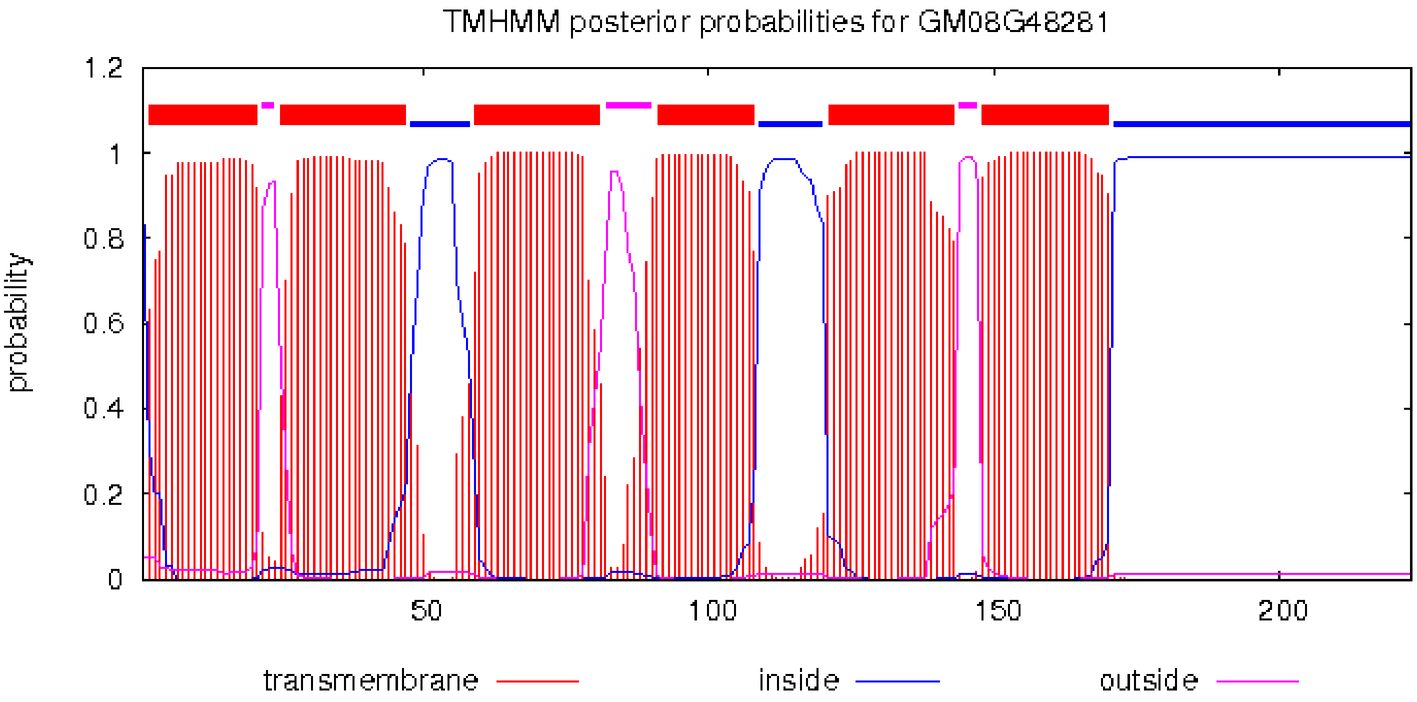

plot in postscript, script for making the plot in gnuplot, data for plot

---

```
# GM09G04840 Length: 244
# GM09G04840 Number of predicted TMHs:  7
# GM09G04840 Exp number of AAs in TMHs: 148.51233
# GM09G04840 Exp number, first 60 AAs:  37.56395
# GM09G04840 Total prob of N-in:        0.28399
# GM09G04840 POSSIBLE N-term signal sequence
GM09G04840      TMHMM2.0        outside      1     5
GM09G04840      TMHMM2.0        TMhelix      6    28
GM09G04840      TMHMM2.0        inside      29    40
GM09G04840      TMHMM2.0        TMhelix     41    60
GM09G04840      TMHMM2.0        outside     61    64
GM09G04840      TMHMM2.0        TMhelix     65    87
GM09G04840      TMHMM2.0        inside      88    98
GM09G04840      TMHMM2.0        TMhelix     99   118
GM09G04840      TMHMM2.0        outside    119   127
GM09G04840      TMHMM2.0        TMhelix    128   147
GM09G04840      TMHMM2.0        inside     148   159
GM09G04840      TMHMM2.0        TMhelix    160   182
GM09G04840      TMHMM2.0        outside    183   185
GM09G04840      TMHMM2.0        TMhelix    186   208
GM09G04840      TMHMM2.0        inside     209   244
```

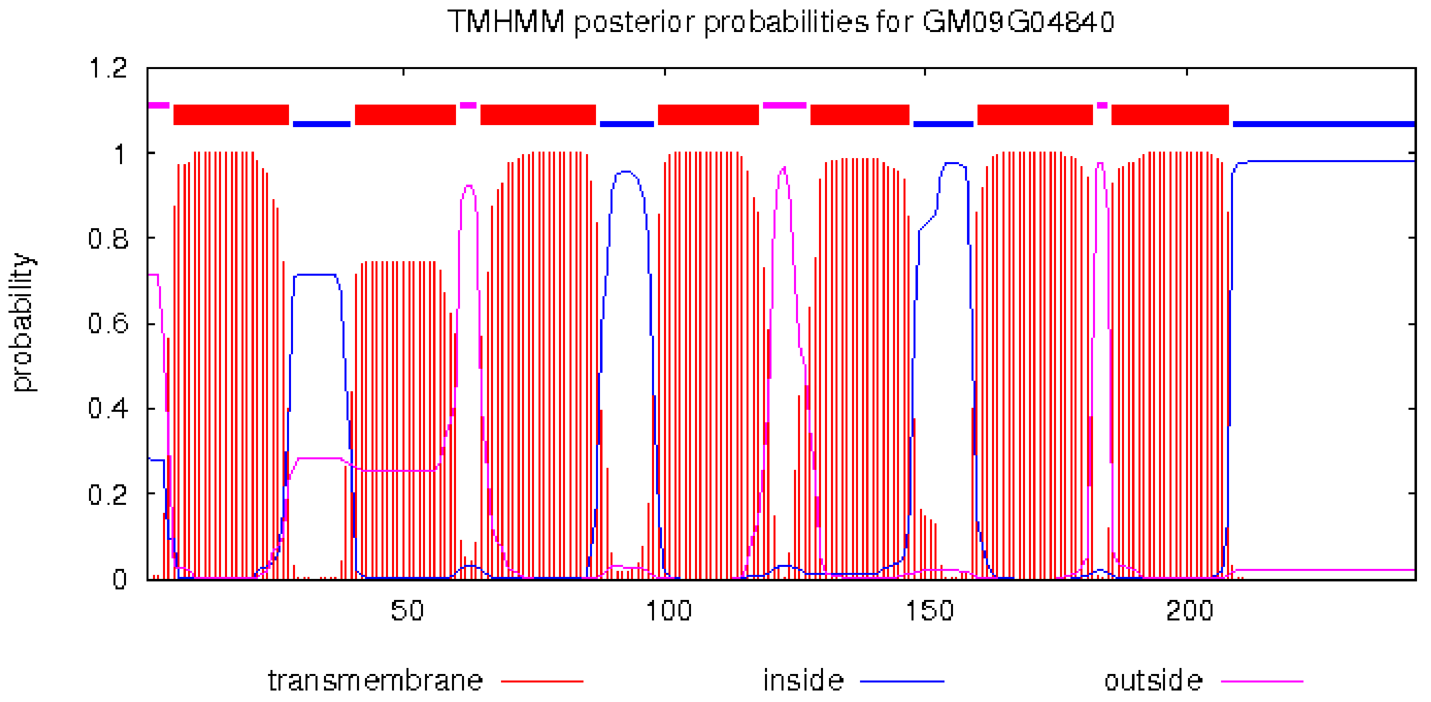

plot in postscript, script for making the plot in gnuplot, data for plot

---

```
# GM11G27070 Length: 128
# GM11G27070 Number of predicted TMHs:  3
# GM11G27070 Exp number of AAs in TMHs: 53.21175
# GM11G27070 Exp number, first 60 AAs:  22.7921
# GM11G27070 Total prob of N-in:        0.14004
# GM11G27070 POSSIBLE N-term signal sequence
GM11G27070      TMHMM2.0        outside      1    35
GM11G27070      TMHMM2.0        TMhelix     36    58
GM11G27070      TMHMM2.0        inside      59    64
GM11G27070      TMHMM2.0        TMhelix     65    84
GM11G27070      TMHMM2.0        outside     85    98
GM11G27070      TMHMM2.0        TMhelix     99   118
GM11G27070      TMHMM2.0        inside     119   128
```

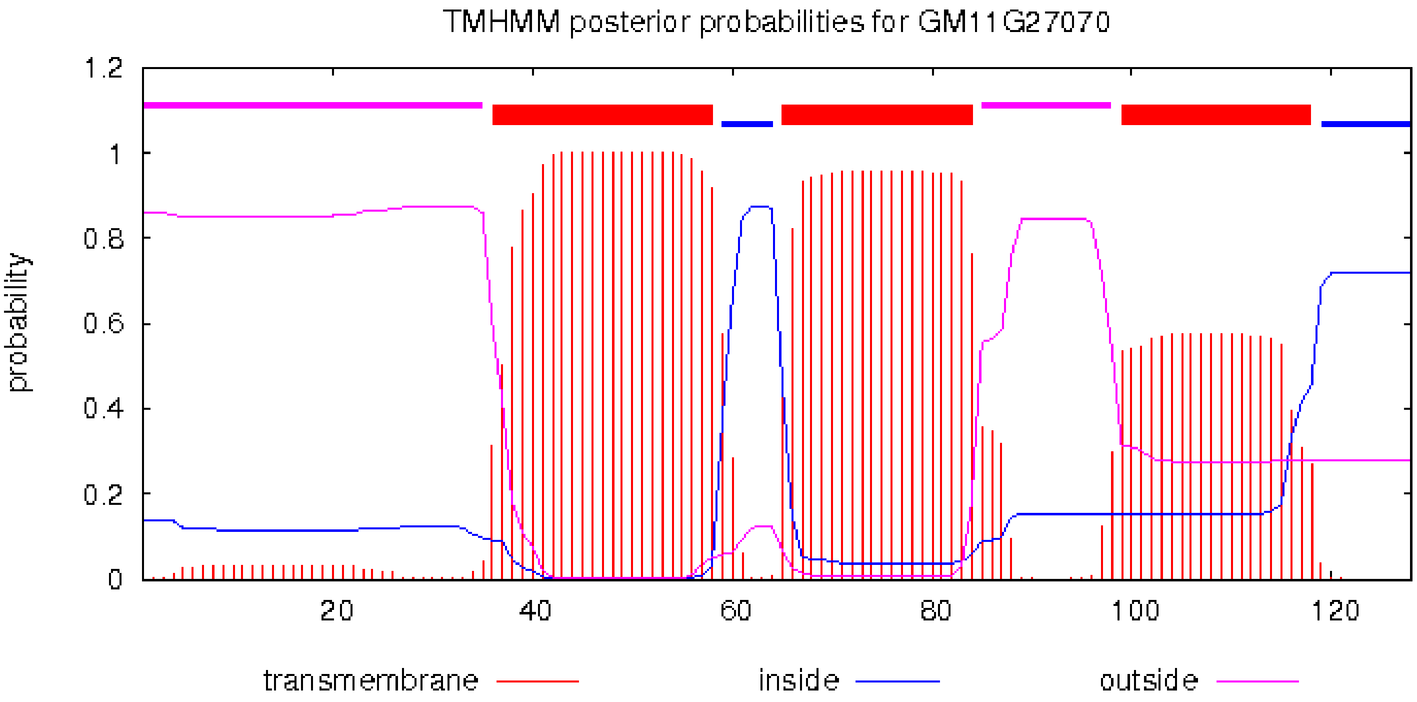

plot in postscript, script for making the plot in gnuplot, data for plot

---

```
# GM12G36300 Length: 235
# GM12G36300 Number of predicted TMHs:  7
# GM12G36300 Exp number of AAs in TMHs: 152.07555
# GM12G36300 Exp number, first 60 AAs:  31.96467
# GM12G36300 Total prob of N-in:        0.06212
# GM12G36300 POSSIBLE N-term signal sequence
GM12G36300      TMHMM2.0        outside      1    14
GM12G36300      TMHMM2.0        TMhelix     15    37
GM12G36300      TMHMM2.0        inside      38    49
GM12G36300      TMHMM2.0        TMhelix     50    72
GM12G36300      TMHMM2.0        outside     73    76
GM12G36300      TMHMM2.0        TMhelix     77    99
GM12G36300      TMHMM2.0        inside     100   105
GM12G36300      TMHMM2.0        TMhelix    106   128
GM12G36300      TMHMM2.0        outside    129   137
GM12G36300      TMHMM2.0        TMhelix    138   157
GM12G36300      TMHMM2.0        inside     158   168
GM12G36300      TMHMM2.0        TMhelix    169   191
GM12G36300      TMHMM2.0        outside    192   195
GM12G36300      TMHMM2.0        TMhelix    196   218
GM12G36300      TMHMM2.0        inside     219   235
```

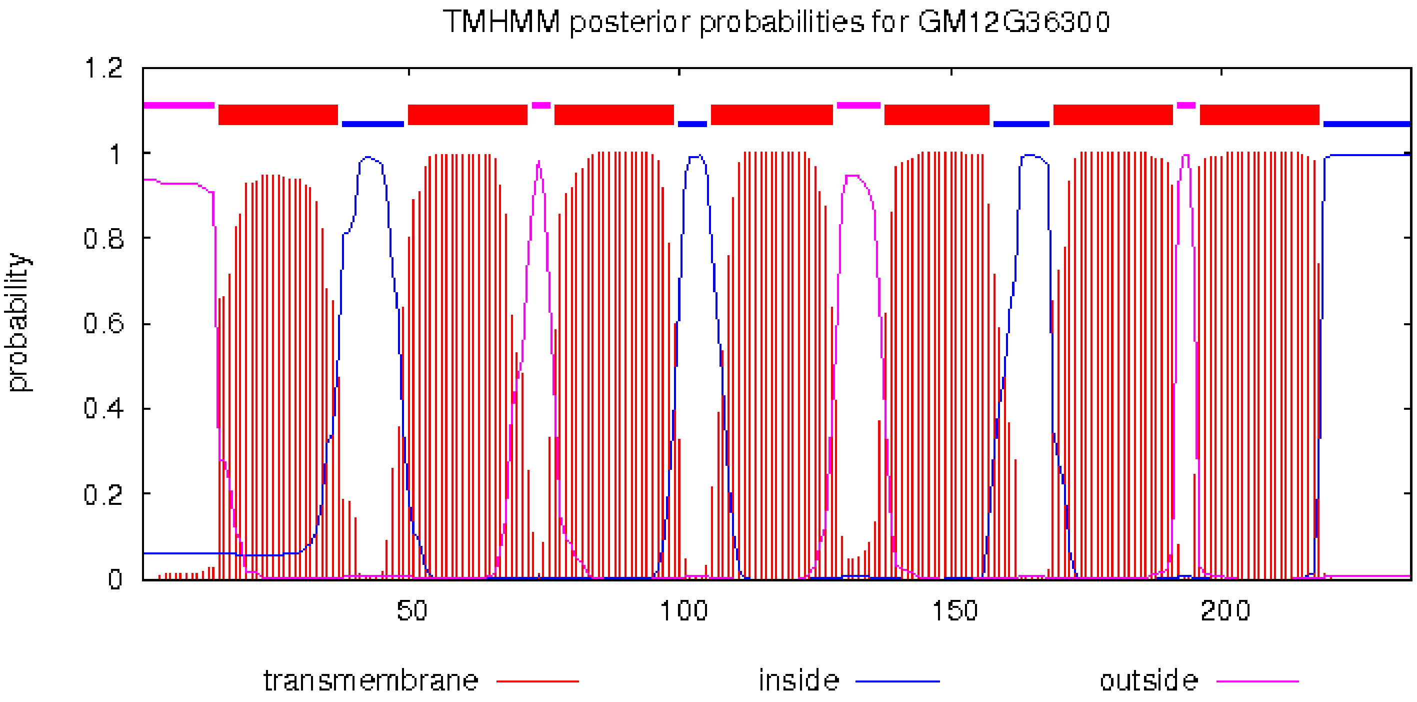

plot in postscript, script for making the plot in gnuplot, data for plot

---

```
# GM13G08190 Length: 255
# GM13G08190 Number of predicted TMHs:  7
# GM13G08190 Exp number of AAs in TMHs: 145.9615
# GM13G08190 Exp number, first 60 AAs:  39.68032
# GM13G08190 Total prob of N-in:        0.26391
# GM13G08190 POSSIBLE N-term signal sequence
GM13G08190      TMHMM2.0        outside      1     4
GM13G08190      TMHMM2.0        TMhelix      5    27
GM13G08190      TMHMM2.0        inside      28    39
GM13G08190      TMHMM2.0        TMhelix     40    62
GM13G08190      TMHMM2.0        outside     63    71
GM13G08190      TMHMM2.0        TMhelix     72    94
GM13G08190      TMHMM2.0        inside      95   100
GM13G08190      TMHMM2.0        TMhelix    101   123
GM13G08190      TMHMM2.0        outside    124   132
GM13G08190      TMHMM2.0        TMhelix    133   152
GM13G08190      TMHMM2.0        inside     153   163
GM13G08190      TMHMM2.0        TMhelix    164   186
GM13G08190      TMHMM2.0        outside    187   189
GM13G08190      TMHMM2.0        TMhelix    190   209
GM13G08190      TMHMM2.0        inside     210   255
```

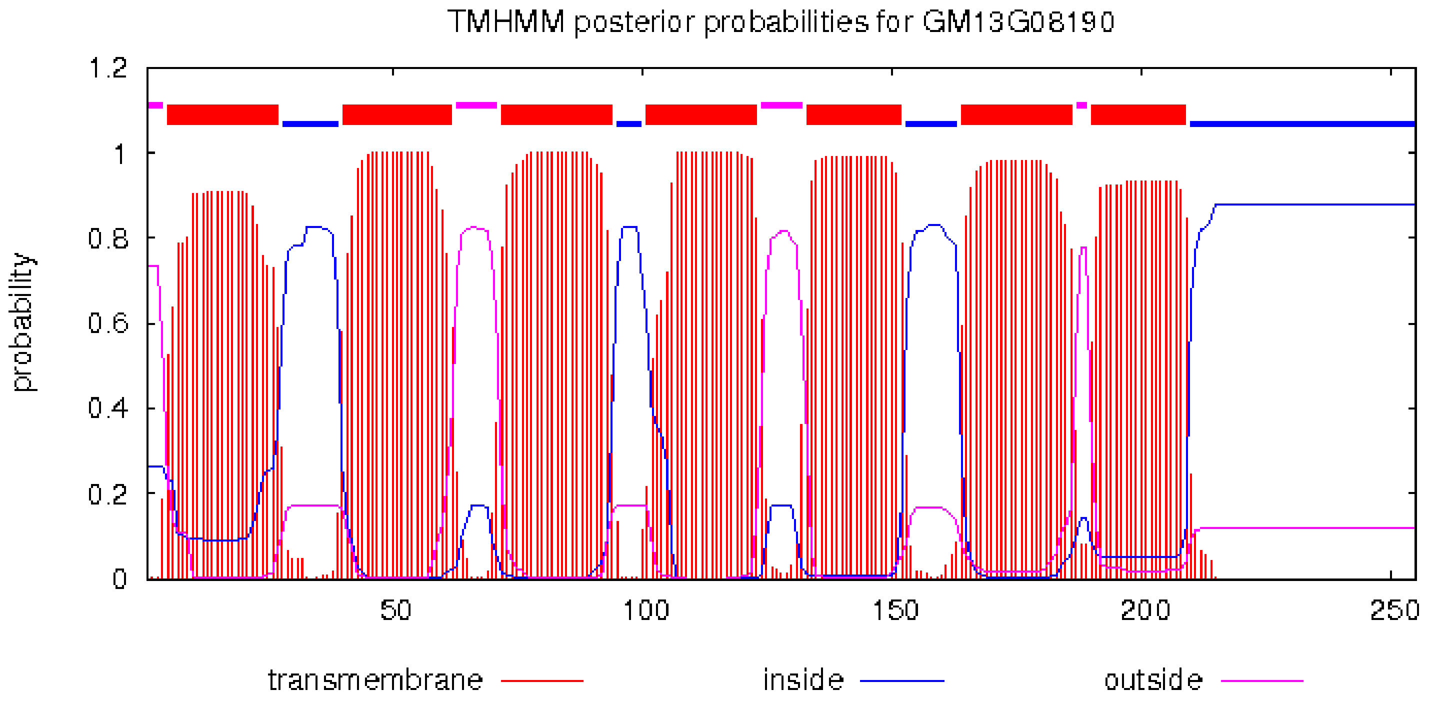

plot in postscript, script for making the plot in gnuplot, data for plot

---

```
# GM13G09140 Length: 248
# GM13G09140 Number of predicted TMHs:  7
# GM13G09140 Exp number of AAs in TMHs: 156.05491
# GM13G09140 Exp number, first 60 AAs:  42.03717
# GM13G09140 Total prob of N-in:        0.06547
# GM13G09140 POSSIBLE N-term signal sequence
GM13G09140      TMHMM2.0        outside      1     9
GM13G09140      TMHMM2.0        TMhelix     10    32
GM13G09140      TMHMM2.0        inside      33    38
GM13G09140      TMHMM2.0        TMhelix     39    58
GM13G09140      TMHMM2.0        outside     59    67
GM13G09140      TMHMM2.0        TMhelix     68    90
GM13G09140      TMHMM2.0        inside      91    98
GM13G09140      TMHMM2.0        TMhelix     99   121
GM13G09140      TMHMM2.0        outside    122   130
GM13G09140      TMHMM2.0        TMhelix    131   153
GM13G09140      TMHMM2.0        inside     154   159
GM13G09140      TMHMM2.0        TMhelix    160   182
GM13G09140      TMHMM2.0        outside    183   186
GM13G09140      TMHMM2.0        TMhelix    187   209
GM13G09140      TMHMM2.0        inside     210   248
```

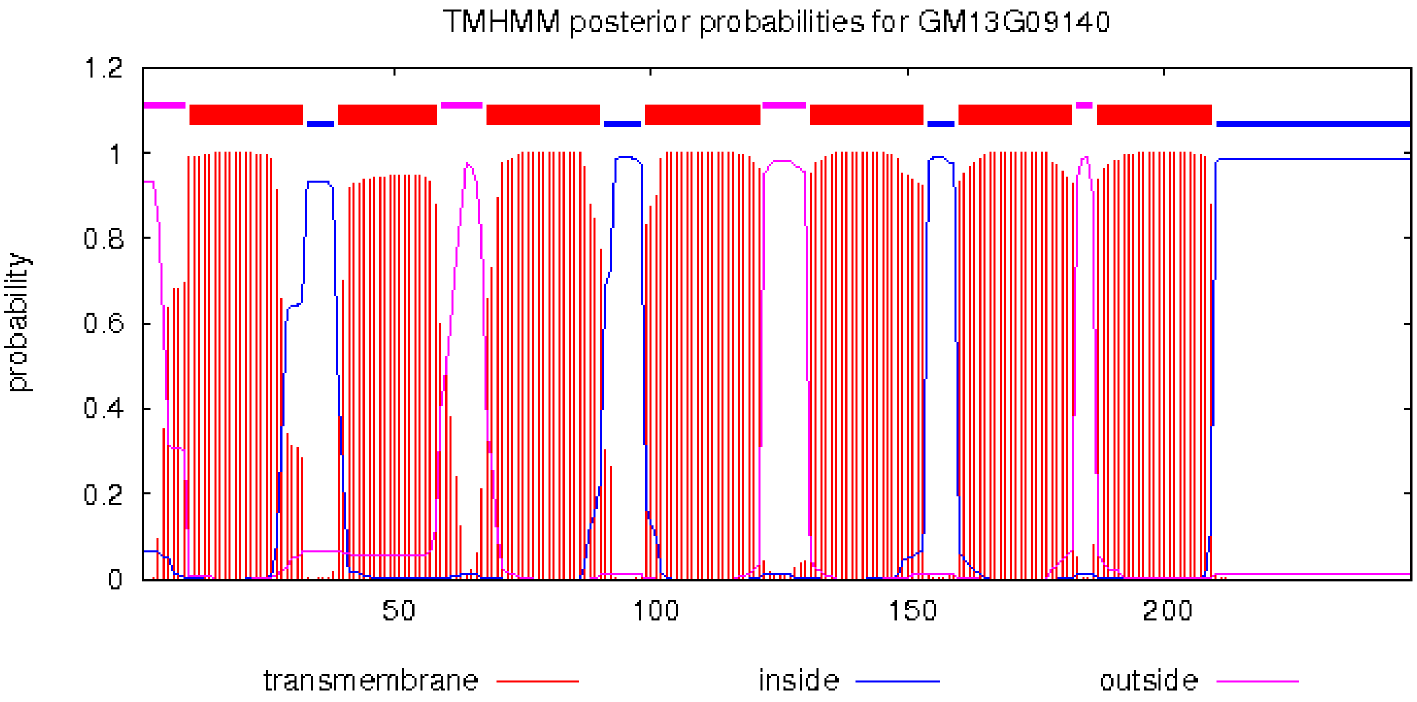

plot in postscript, script for making the plot in gnuplot, data for plot

---

```
# GM13G10560 Length: 257
# GM13G10560 Number of predicted TMHs:  7
# GM13G10560 Exp number of AAs in TMHs: 152.79174
# GM13G10560 Exp number, first 60 AAs:  37.63561
# GM13G10560 Total prob of N-in:        0.13081
# GM13G10560 POSSIBLE N-term signal sequence
GM13G10560      TMHMM2.0        outside      1     9
GM13G10560      TMHMM2.0        TMhelix     10    32
GM13G10560      TMHMM2.0        inside      33    43
GM13G10560      TMHMM2.0        TMhelix     44    66
GM13G10560      TMHMM2.0        outside     67    70
GM13G10560      TMHMM2.0        TMhelix     71    93
GM13G10560      TMHMM2.0        inside      94    99
GM13G10560      TMHMM2.0        TMhelix    100   122
GM13G10560      TMHMM2.0        outside    123   131
GM13G10560      TMHMM2.0        TMhelix    132   151
GM13G10560      TMHMM2.0        inside     152   163
GM13G10560      TMHMM2.0        TMhelix    164   186
GM13G10560      TMHMM2.0        outside    187   190
GM13G10560      TMHMM2.0        TMhelix    191   213
GM13G10560      TMHMM2.0        inside     214   257
```

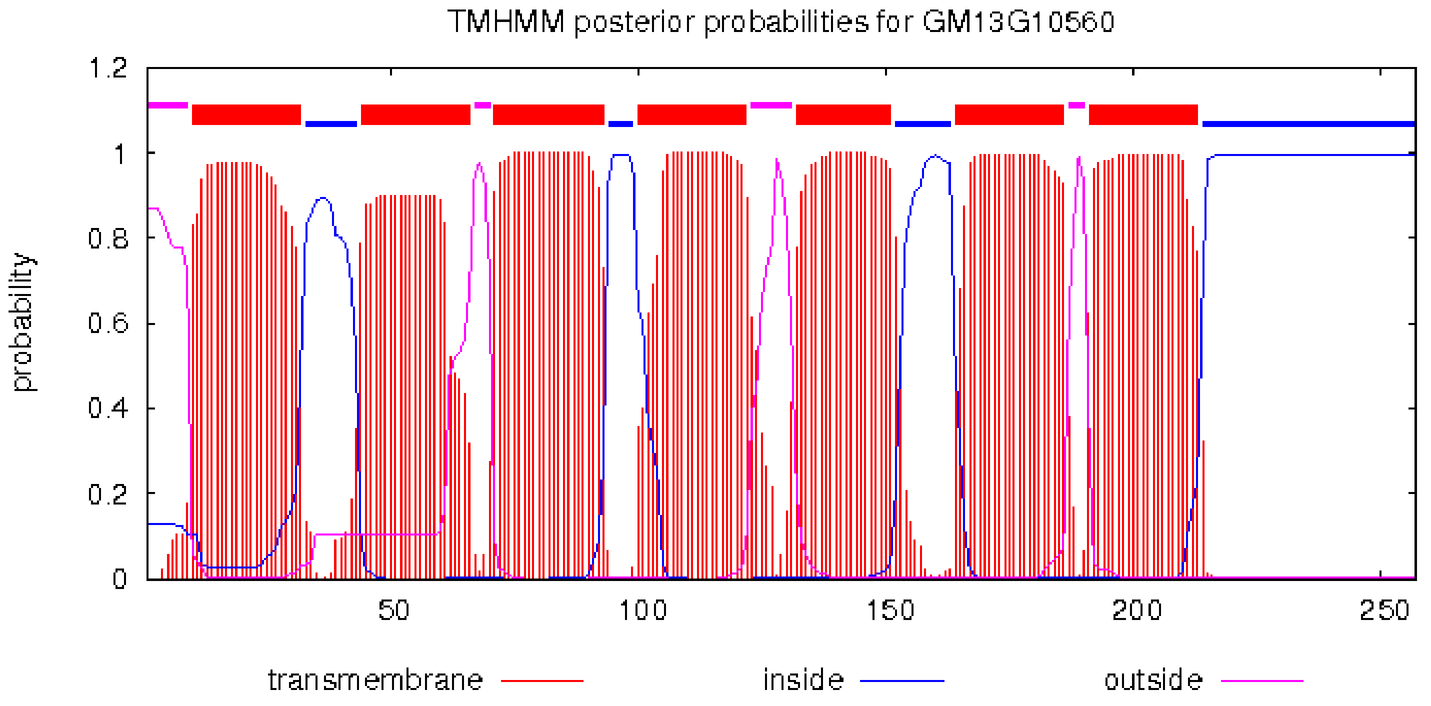

plot in postscript, script for making the plot in gnuplot, data for plot

---

```
# GM13G23860 Length: 245
# GM13G23860 Number of predicted TMHs:  6
# GM13G23860 Exp number of AAs in TMHs: 144.83132
# GM13G23860 Exp number, first 60 AAs:  31.91567
# GM13G23860 Total prob of N-in:        0.53166
# GM13G23860 POSSIBLE N-term signal sequence
GM13G23860      TMHMM2.0        inside       1    11
GM13G23860      TMHMM2.0        TMhelix     12    34
GM13G23860      TMHMM2.0        outside     35    68
GM13G23860      TMHMM2.0        TMhelix     69    91
GM13G23860      TMHMM2.0        inside      92   102
GM13G23860      TMHMM2.0        TMhelix    103   125
GM13G23860      TMHMM2.0        outside    126   134
GM13G23860      TMHMM2.0        TMhelix    135   157
GM13G23860      TMHMM2.0        inside     158   163
GM13G23860      TMHMM2.0        TMhelix    164   186
GM13G23860      TMHMM2.0        outside    187   190
GM13G23860      TMHMM2.0        TMhelix    191   213
GM13G23860      TMHMM2.0        inside     214   245
```

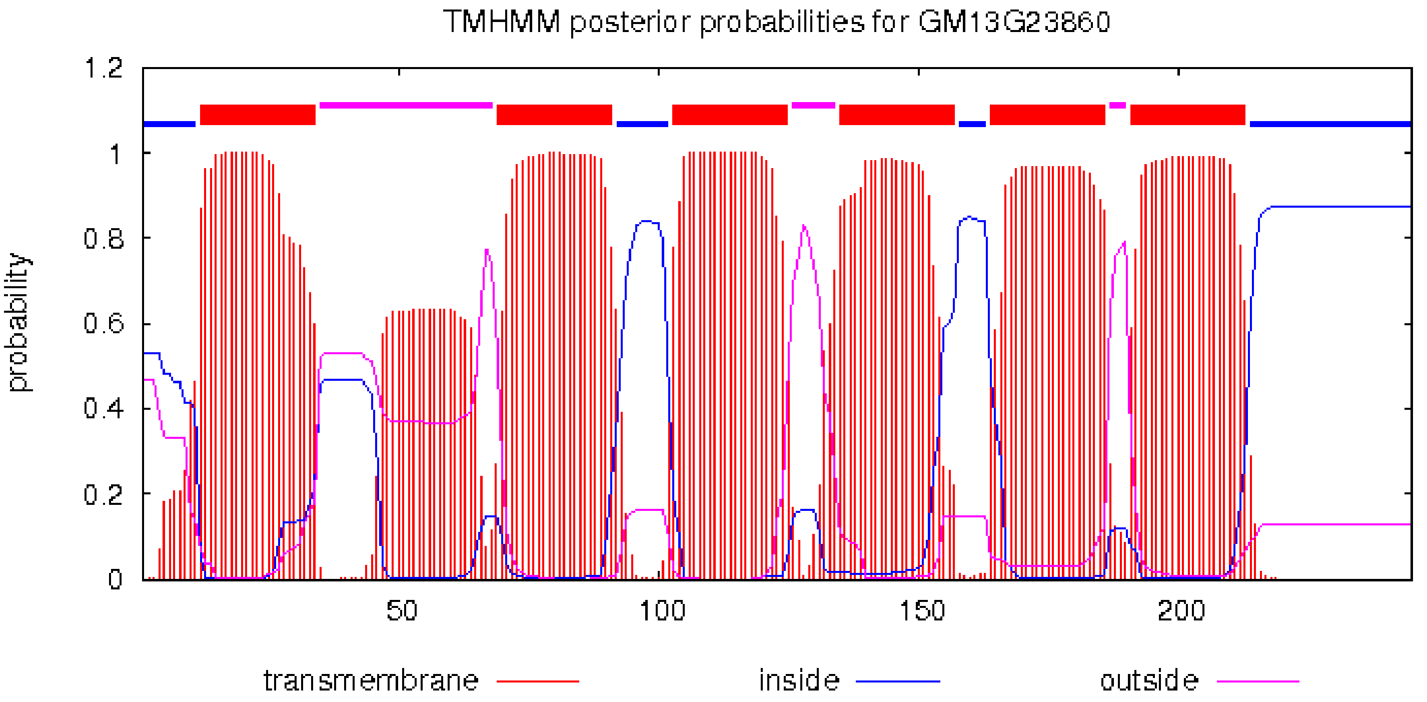

plot in postscript, script for making the plot in gnuplot, data for plot

---

```
# GM13G33950 Length: 235
# GM13G33950 Number of predicted TMHs:  7
# GM13G33950 Exp number of AAs in TMHs: 151.65951
# GM13G33950 Exp number, first 60 AAs:  31.61705
# GM13G33950 Total prob of N-in:        0.08310
# GM13G33950 POSSIBLE N-term signal sequence
GM13G33950      TMHMM2.0        outside      1    14
GM13G33950      TMHMM2.0        TMhelix     15    37
GM13G33950      TMHMM2.0        inside      38    49
GM13G33950      TMHMM2.0        TMhelix     50    72
GM13G33950      TMHMM2.0        outside     73    76
GM13G33950      TMHMM2.0        TMhelix     77    99
GM13G33950      TMHMM2.0        inside     100   105
GM13G33950      TMHMM2.0        TMhelix    106   128
GM13G33950      TMHMM2.0        outside    129   137
GM13G33950      TMHMM2.0        TMhelix    138   157
GM13G33950      TMHMM2.0        inside     158   168
GM13G33950      TMHMM2.0        TMhelix    169   191
GM13G33950      TMHMM2.0        outside    192   195
GM13G33950      TMHMM2.0        TMhelix    196   218
GM13G33950      TMHMM2.0        inside     219   235
```

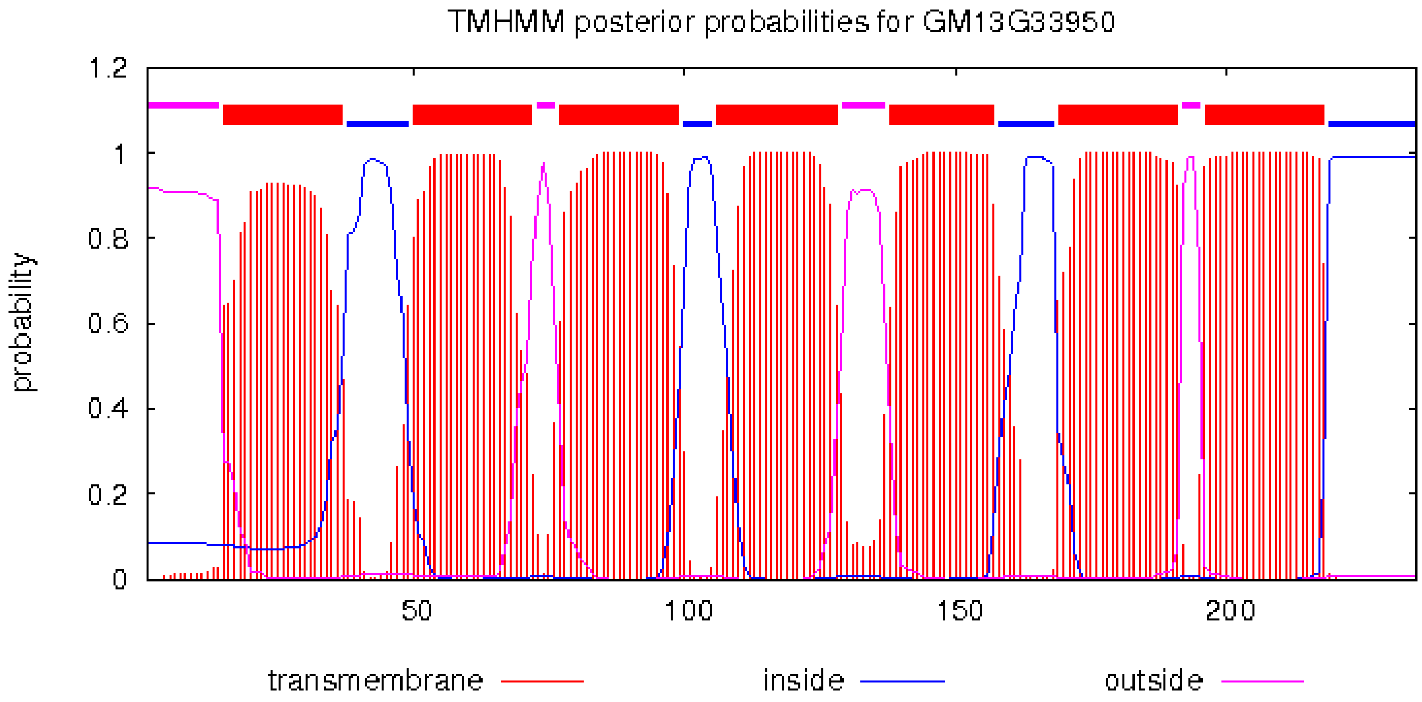

plot in postscript, script for making the plot in gnuplot, data for plot

---

```
# GM14G17811 Length: 181
# GM14G17811 Number of predicted TMHs:  5
# GM14G17811 Exp number of AAs in TMHs: 109.98662
# GM14G17811 Exp number, first 60 AAs:  39.34855
# GM14G17811 Total prob of N-in:        0.03399
# GM14G17811 POSSIBLE N-term signal sequence
GM14G17811      TMHMM2.0        outside      1     9
GM14G17811      TMHMM2.0        TMhelix     10    32
GM14G17811      TMHMM2.0        inside      33    44
GM14G17811      TMHMM2.0        TMhelix     45    62
GM14G17811      TMHMM2.0        outside     63    71
GM14G17811      TMHMM2.0        TMhelix     72    94
GM14G17811      TMHMM2.0        inside      95   106
GM14G17811      TMHMM2.0        TMhelix    107   126
GM14G17811      TMHMM2.0        outside    127   135
GM14G17811      TMHMM2.0        TMhelix    136   158
GM14G17811      TMHMM2.0        inside     159   181
```

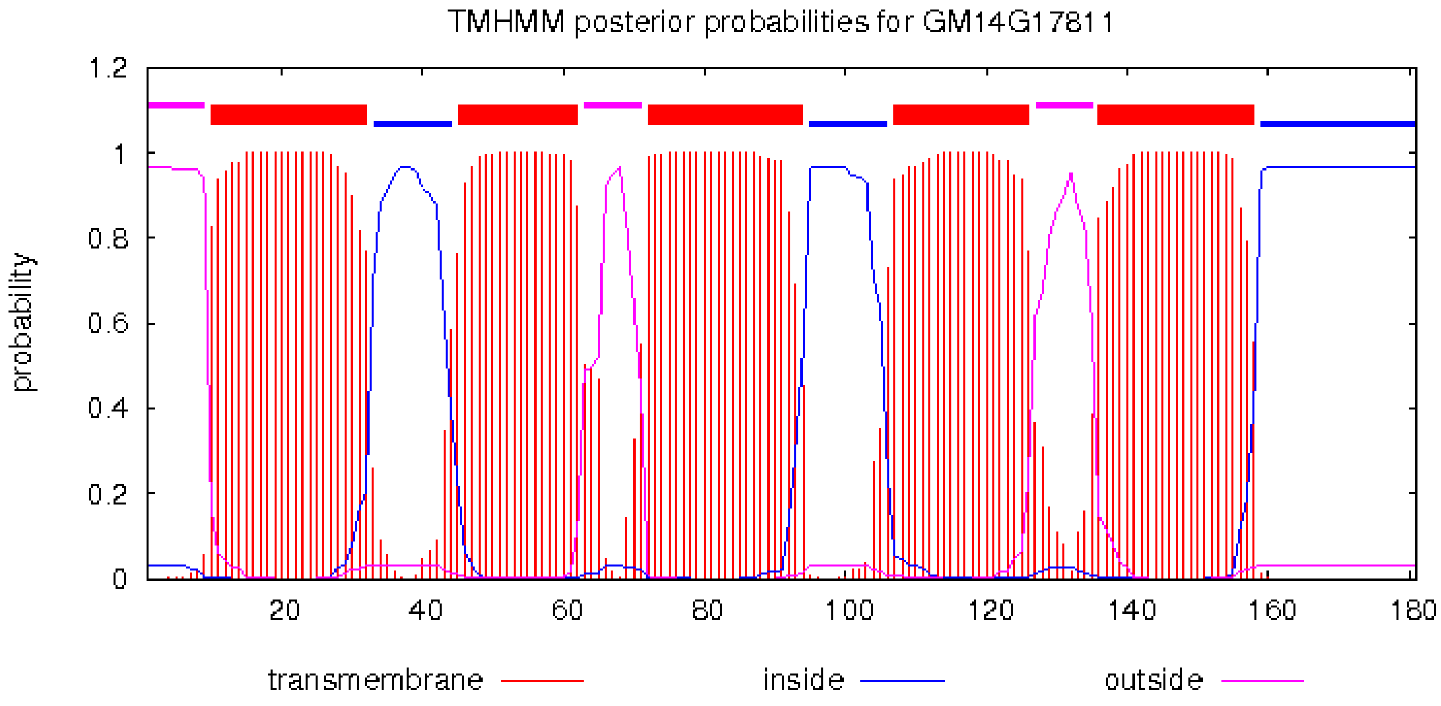

plot in postscript, script for making the plot in gnuplot, data for plot

---

```
# GM14G27610 Length: 249
# GM14G27610 Number of predicted TMHs:  7
# GM14G27610 Exp number of AAs in TMHs: 156.82827
# GM14G27610 Exp number, first 60 AAs:  42.5239
# GM14G27610 Total prob of N-in:        0.03137
# GM14G27610 POSSIBLE N-term signal sequence
GM14G27610      TMHMM2.0        outside      1     5
GM14G27610      TMHMM2.0        TMhelix      6    28
GM14G27610      TMHMM2.0        inside      29    40
GM14G27610      TMHMM2.0        TMhelix     41    63
GM14G27610      TMHMM2.0        outside     64    67
GM14G27610      TMHMM2.0        TMhelix     68    90
GM14G27610      TMHMM2.0        inside      91    98
GM14G27610      TMHMM2.0        TMhelix     99   121
GM14G27610      TMHMM2.0        outside    122   130
GM14G27610      TMHMM2.0        TMhelix    131   153
GM14G27610      TMHMM2.0        inside     154   159
GM14G27610      TMHMM2.0        TMhelix    160   182
GM14G27610      TMHMM2.0        outside    183   186
GM14G27610      TMHMM2.0        TMhelix    187   209
GM14G27610      TMHMM2.0        inside     210   249
```

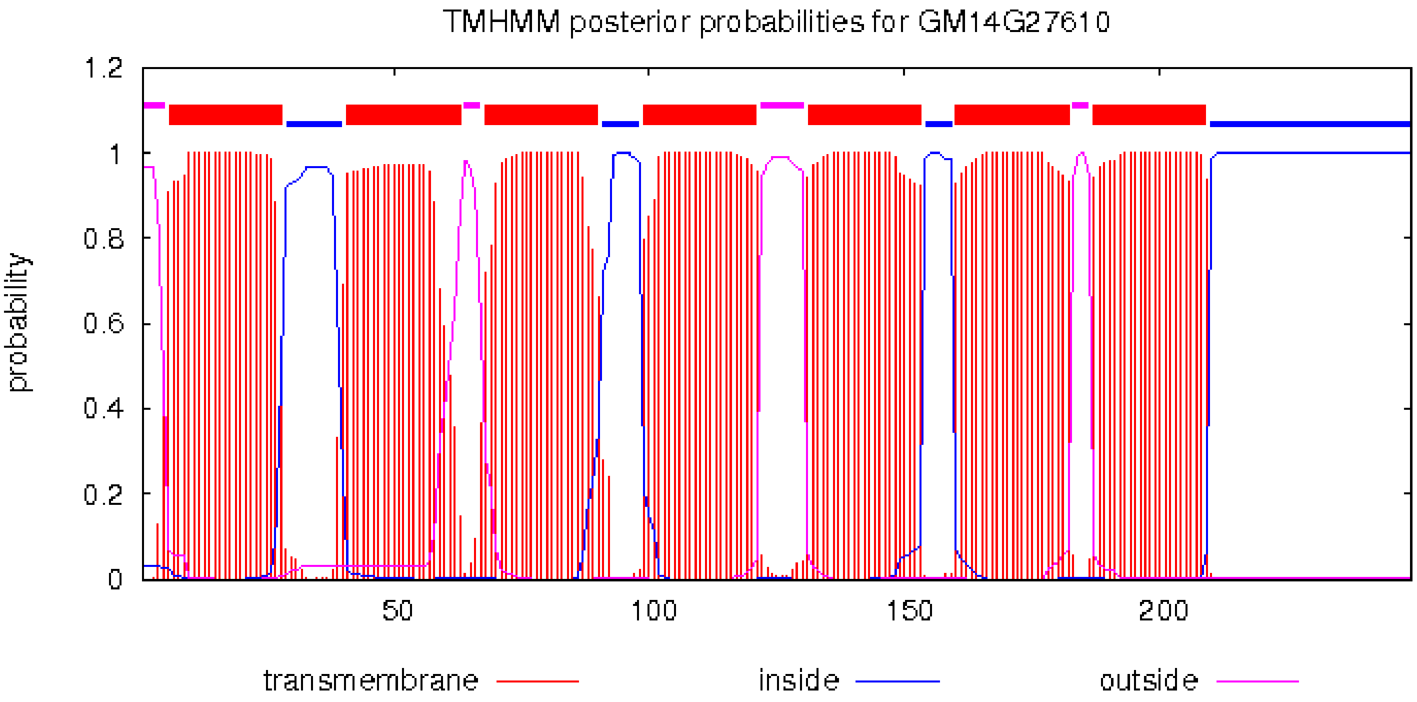

plot in postscript, script for making the plot in gnuplot, data for plot

---

```
# GM14G30740 Length: 260
# GM14G30740 Number of predicted TMHs:  6
# GM14G30740 Exp number of AAs in TMHs: 139.59389
# GM14G30740 Exp number, first 60 AAs:  38.79296
# GM14G30740 Total prob of N-in:        0.64587
# GM14G30740 POSSIBLE N-term signal sequence
GM14G30740      TMHMM2.0        inside       1     6
GM14G30740      TMHMM2.0        TMhelix      7    26
GM14G30740      TMHMM2.0        outside     27    40
GM14G30740      TMHMM2.0        TMhelix     41    63
GM14G30740      TMHMM2.0        inside      64    69
GM14G30740      TMHMM2.0        TMhelix     70    92
GM14G30740      TMHMM2.0        outside     93   101
GM14G30740      TMHMM2.0        TMhelix    102   124
GM14G30740      TMHMM2.0        inside     125   130
GM14G30740      TMHMM2.0        TMhelix    131   153
GM14G30740      TMHMM2.0        outside    154   186
GM14G30740      TMHMM2.0        TMhelix    187   209
GM14G30740      TMHMM2.0        inside     210   260
```

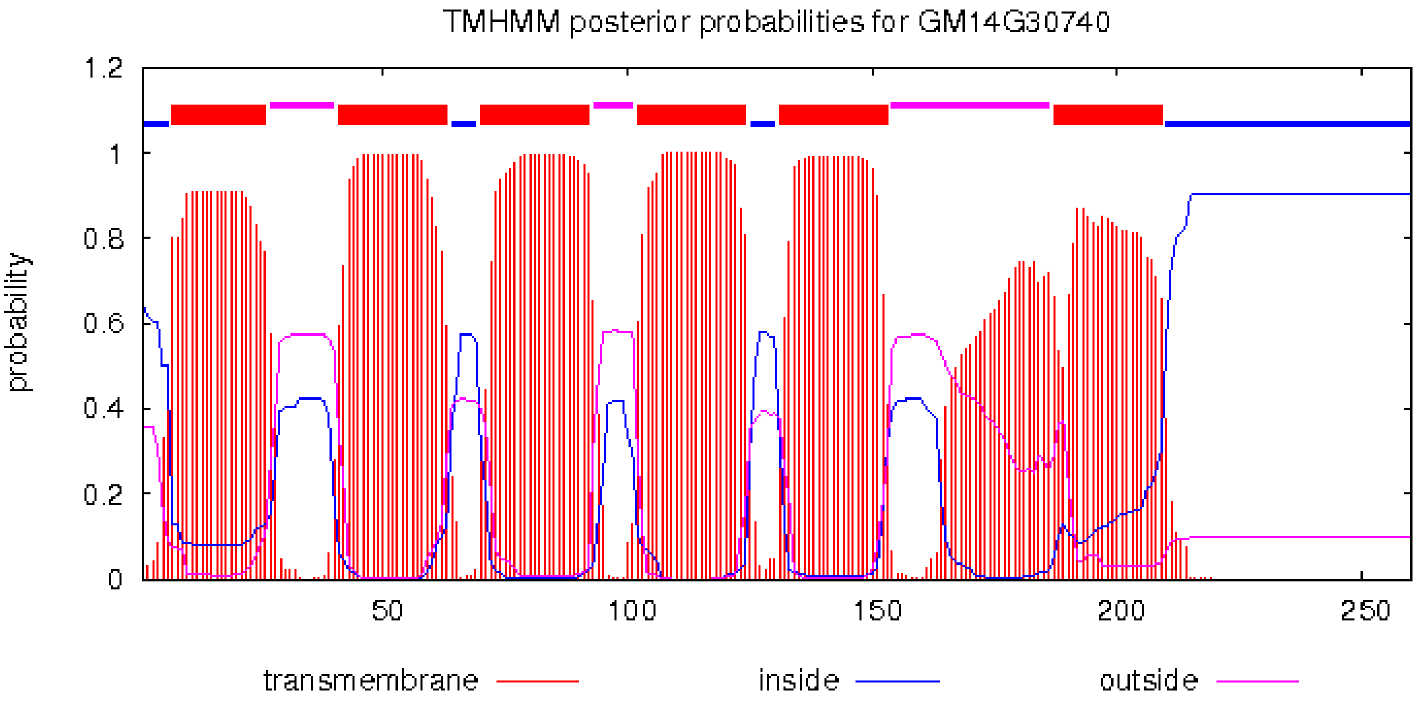

plot in postscript, script for making the plot in gnuplot, data for plot

---

```
# GM14G30940 Length: 254
# GM14G30940 Number of predicted TMHs:  7
# GM14G30940 Exp number of AAs in TMHs: 145.98069
# GM14G30940 Exp number, first 60 AAs:  39.3873
# GM14G30940 Total prob of N-in:        0.31555
# GM14G30940 POSSIBLE N-term signal sequence
GM14G30940      TMHMM2.0        outside      1     4
GM14G30940      TMHMM2.0        TMhelix      5    27
GM14G30940      TMHMM2.0        inside      28    39
GM14G30940      TMHMM2.0        TMhelix     40    62
GM14G30940      TMHMM2.0        outside     63    71
GM14G30940      TMHMM2.0        TMhelix     72    94
GM14G30940      TMHMM2.0        inside      95   100
GM14G30940      TMHMM2.0        TMhelix    101   123
GM14G30940      TMHMM2.0        outside    124   132
GM14G30940      TMHMM2.0        TMhelix    133   152
GM14G30940      TMHMM2.0        inside     153   163
GM14G30940      TMHMM2.0        TMhelix    164   186
GM14G30940      TMHMM2.0        outside    187   189
GM14G30940      TMHMM2.0        TMhelix    190   209
GM14G30940      TMHMM2.0        inside     210   254
```

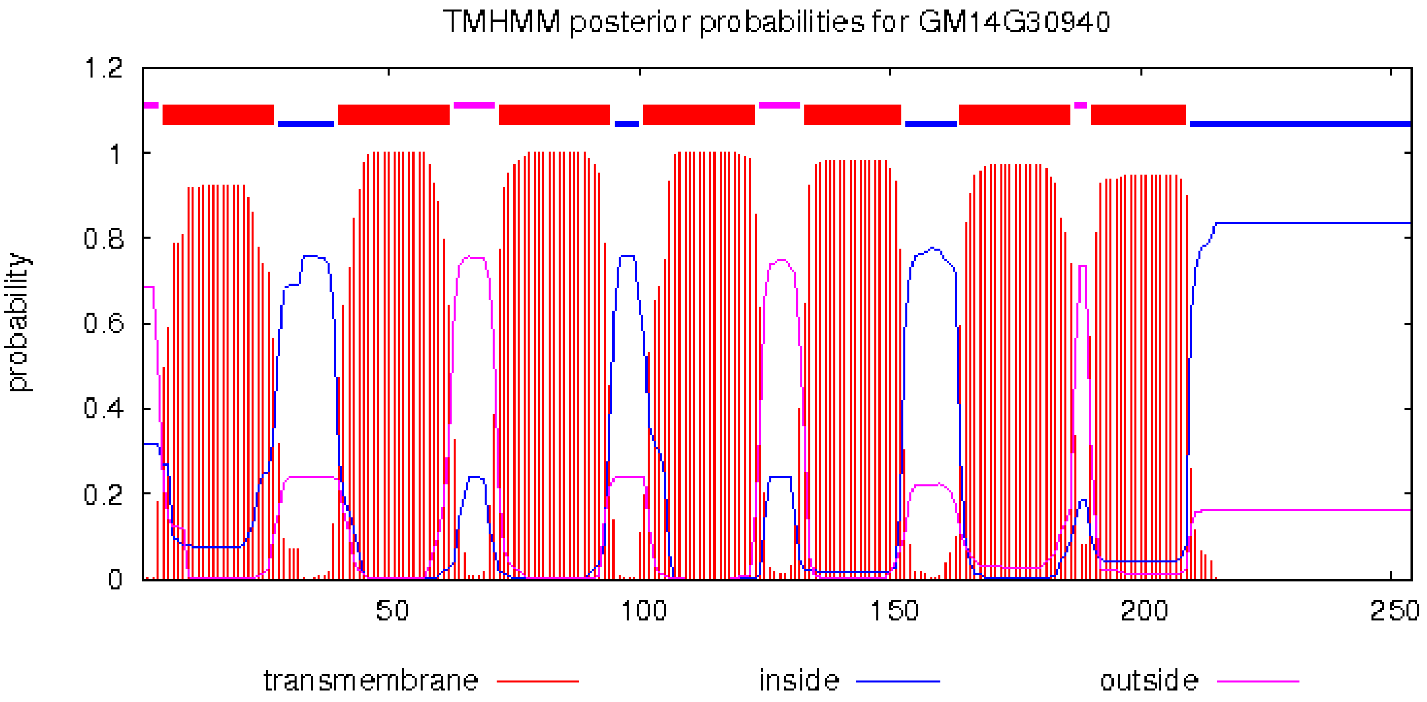

plot in postscript, script for making the plot in gnuplot, data for plot

---

```
# GM15G05470 Length: 249
# GM15G05470 Number of predicted TMHs:  7
# GM15G05470 Exp number of AAs in TMHs: 150.01361
# GM15G05470 Exp number, first 60 AAs:  40.5933
# GM15G05470 Total prob of N-in:        0.38150
# GM15G05470 POSSIBLE N-term signal sequence
GM15G05470      TMHMM2.0        outside      1     5
GM15G05470      TMHMM2.0        TMhelix      6    28
GM15G05470      TMHMM2.0        inside      29    40
GM15G05470      TMHMM2.0        TMhelix     41    63
GM15G05470      TMHMM2.0        outside     64    67
GM15G05470      TMHMM2.0        TMhelix     68    90
GM15G05470      TMHMM2.0        inside      91   102
GM15G05470      TMHMM2.0        TMhelix    103   122
GM15G05470      TMHMM2.0        outside    123   131
GM15G05470      TMHMM2.0        TMhelix    132   151
GM15G05470      TMHMM2.0        inside     152   163
GM15G05470      TMHMM2.0        TMhelix    164   186
GM15G05470      TMHMM2.0        outside    187   189
GM15G05470      TMHMM2.0        TMhelix    190   212
GM15G05470      TMHMM2.0        inside     213   249
```

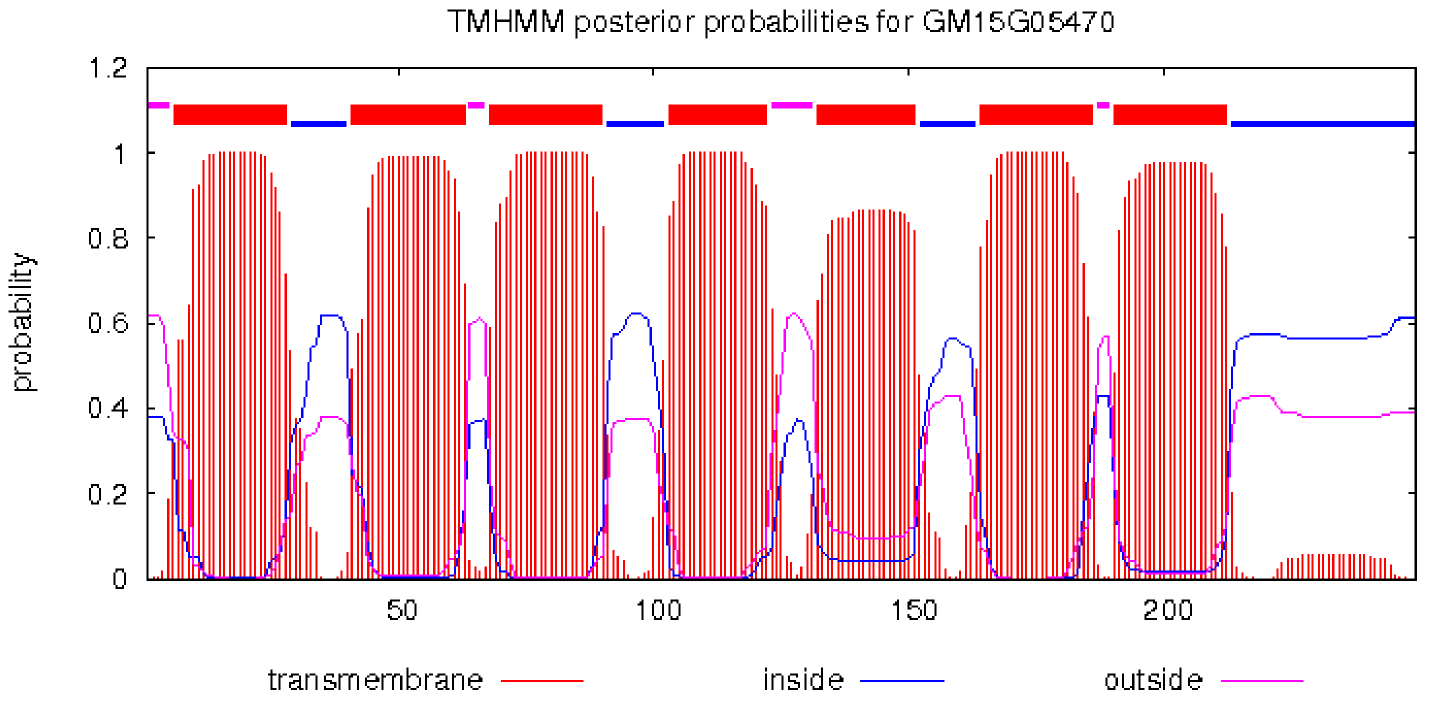

plot in postscript, script for making the plot in gnuplot, data for plot

---

```
# GM15G16030 Length: 245
# GM15G16030 Number of predicted TMHs:  7
# GM15G16030 Exp number of AAs in TMHs: 152.75624
# GM15G16030 Exp number, first 60 AAs:  41.15404
# GM15G16030 Total prob of N-in:        0.18700
# GM15G16030 POSSIBLE N-term signal sequence
GM15G16030      TMHMM2.0        outside      1     5
GM15G16030      TMHMM2.0        TMhelix      6    28
GM15G16030      TMHMM2.0        inside      29    40
GM15G16030      TMHMM2.0        TMhelix     41    60
GM15G16030      TMHMM2.0        outside     61    64
GM15G16030      TMHMM2.0        TMhelix     65    87
GM15G16030      TMHMM2.0        inside      88    98
GM15G16030      TMHMM2.0        TMhelix     99   121
GM15G16030      TMHMM2.0        outside    122   125
GM15G16030      TMHMM2.0        TMhelix    126   148
GM15G16030      TMHMM2.0        inside     149   159
GM15G16030      TMHMM2.0        TMhelix    160   182
GM15G16030      TMHMM2.0        outside    183   185
GM15G16030      TMHMM2.0        TMhelix    186   208
GM15G16030      TMHMM2.0        inside     209   245
```

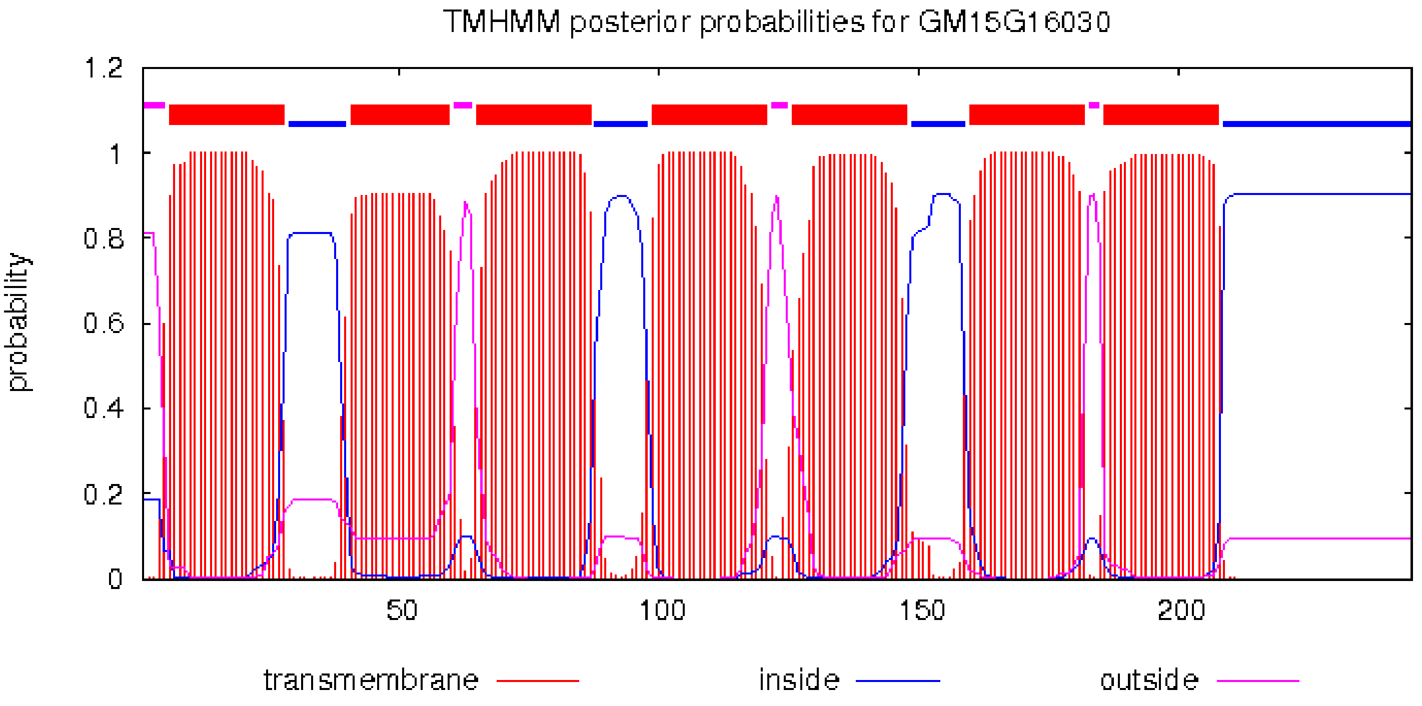

plot in postscript, script for making the plot in gnuplot, data for plot

---

```
# GM15G27530 Length: 223
# GM15G27530 Number of predicted TMHs:  6
# GM15G27530 Exp number of AAs in TMHs: 131.53331
# GM15G27530 Exp number, first 60 AAs:  21.66482
# GM15G27530 Total prob of N-in:        0.99698
# GM15G27530 POSSIBLE N-term signal sequence
GM15G27530      TMHMM2.0        inside       1    35
GM15G27530      TMHMM2.0        TMhelix     36    55
GM15G27530      TMHMM2.0        outside     56    64
GM15G27530      TMHMM2.0        TMhelix     65    87
GM15G27530      TMHMM2.0        inside      88    93
GM15G27530      TMHMM2.0        TMhelix     94   116
GM15G27530      TMHMM2.0        outside    117   125
GM15G27530      TMHMM2.0        TMhelix    126   145
GM15G27530      TMHMM2.0        inside     146   156
GM15G27530      TMHMM2.0        TMhelix    157   179
GM15G27530      TMHMM2.0        outside    180   183
GM15G27530      TMHMM2.0        TMhelix    184   206
GM15G27530      TMHMM2.0        inside     207   223
```

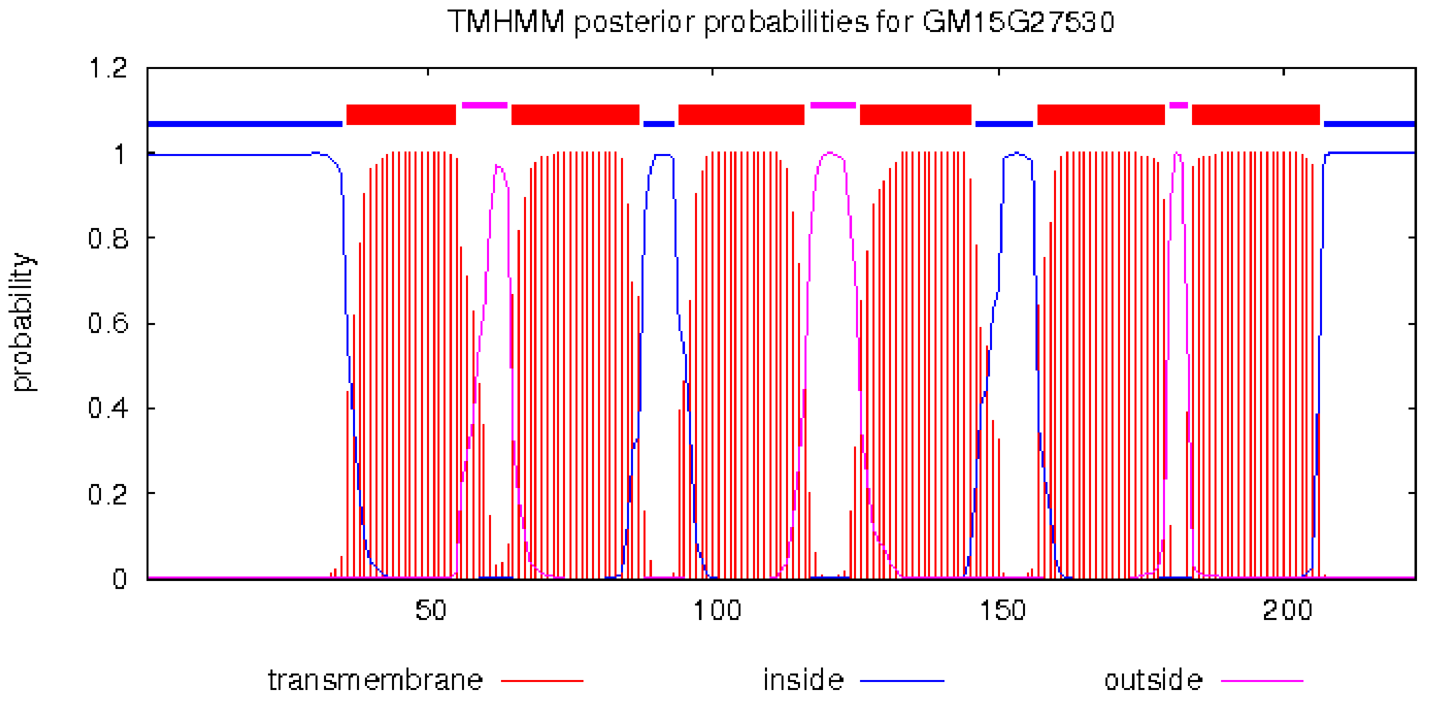

plot in postscript, script for making the plot in gnuplot, data for plot

---

```
# GM15G27750 Length: 235
# GM15G27750 Number of predicted TMHs:  7
# GM15G27750 Exp number of AAs in TMHs: 151.96939
# GM15G27750 Exp number, first 60 AAs:  32.28583
# GM15G27750 Total prob of N-in:        0.04652
# GM15G27750 POSSIBLE N-term signal sequence
GM15G27750      TMHMM2.0        outside      1    14
GM15G27750      TMHMM2.0        TMhelix     15    37
GM15G27750      TMHMM2.0        inside      38    49
GM15G27750      TMHMM2.0        TMhelix     50    72
GM15G27750      TMHMM2.0        outside     73    76
GM15G27750      TMHMM2.0        TMhelix     77    99
GM15G27750      TMHMM2.0        inside     100   105
GM15G27750      TMHMM2.0        TMhelix    106   128
GM15G27750      TMHMM2.0        outside    129   137
GM15G27750      TMHMM2.0        TMhelix    138   157
GM15G27750      TMHMM2.0        inside     158   168
GM15G27750      TMHMM2.0        TMhelix    169   191
GM15G27750      TMHMM2.0        outside    192   195
GM15G27750      TMHMM2.0        TMhelix    196   218
GM15G27750      TMHMM2.0        inside     219   235
```

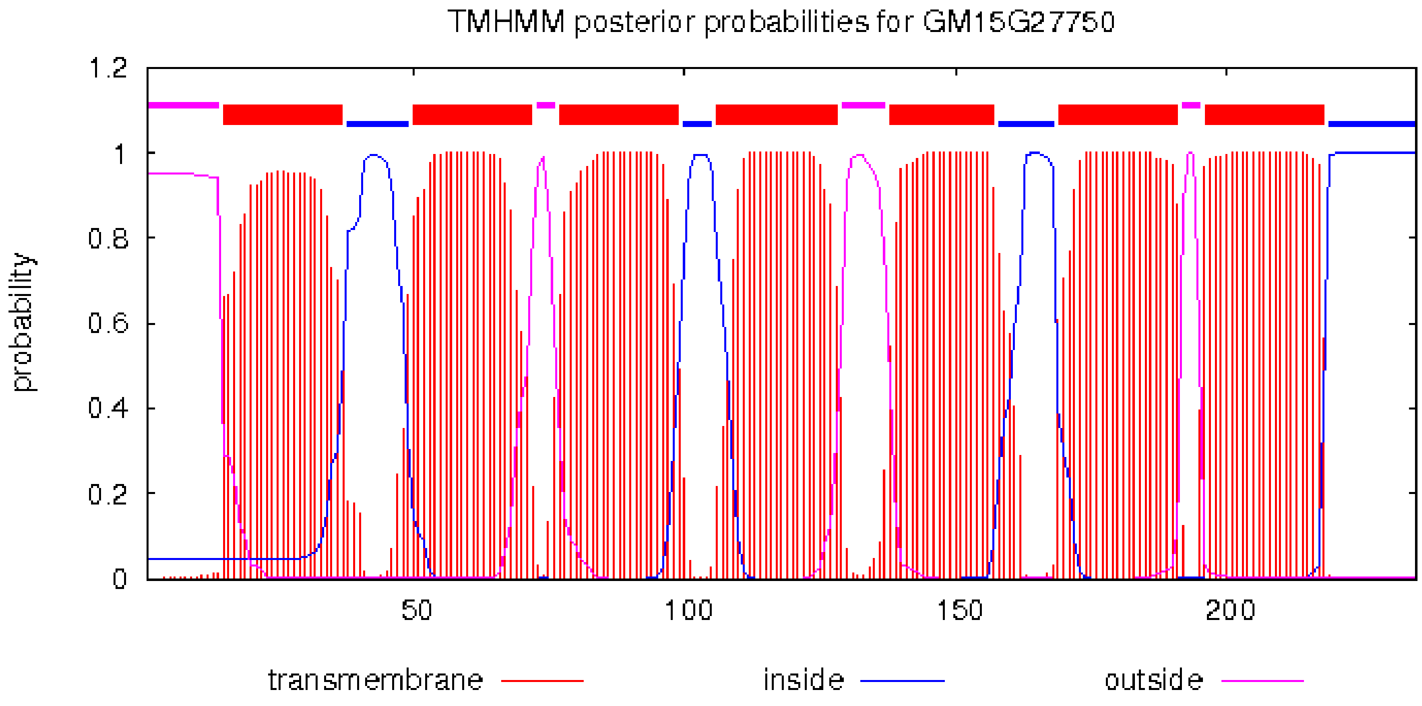

plot in postscript, script for making the plot in gnuplot, data for plot

---

```
# GM17G09840 Length: 226
# GM17G09840 Number of predicted TMHs:  6
# GM17G09840 Exp number of AAs in TMHs: 137.84792
# GM17G09840 Exp number, first 60 AAs:  27.12712
# GM17G09840 Total prob of N-in:        0.74021
# GM17G09840 POSSIBLE N-term signal sequence
GM17G09840      TMHMM2.0        inside       1    11
GM17G09840      TMHMM2.0        TMhelix     12    34
GM17G09840      TMHMM2.0        outside     35    71
GM17G09840      TMHMM2.0        TMhelix     72    94
GM17G09840      TMHMM2.0        inside      95   100
GM17G09840      TMHMM2.0        TMhelix    101   123
GM17G09840      TMHMM2.0        outside    124   132
GM17G09840      TMHMM2.0        TMhelix    133   152
GM17G09840      TMHMM2.0        inside     153   163
GM17G09840      TMHMM2.0        TMhelix    164   186
GM17G09840      TMHMM2.0        outside    187   190
GM17G09840      TMHMM2.0        TMhelix    191   213
GM17G09840      TMHMM2.0        inside     214   226
```

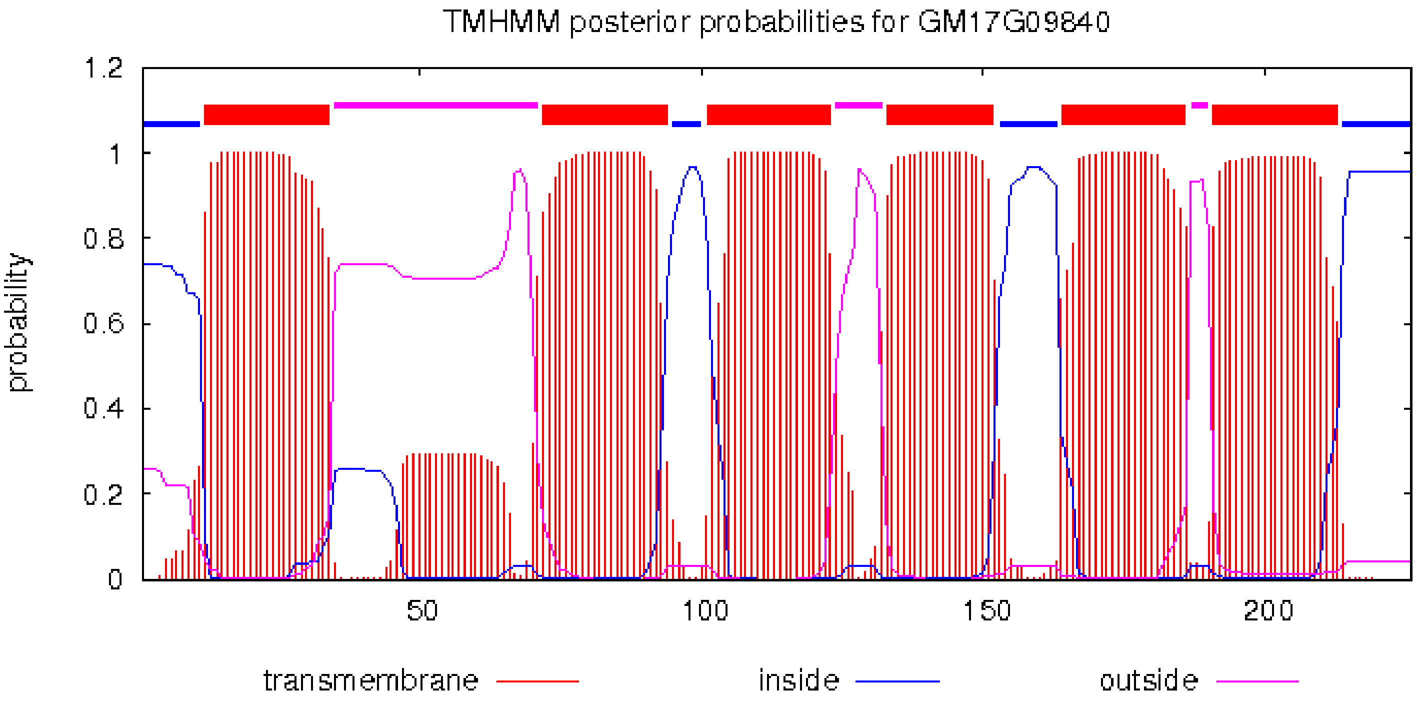

plot in postscript, script for making the plot in gnuplot, data for plot

---

```
# GM18G53250 Length: 297
# GM18G53250 Number of predicted TMHs:  7
# GM18G53250 Exp number of AAs in TMHs: 158.30202
# GM18G53250 Exp number, first 60 AAs:  19.20872
# GM18G53250 Total prob of N-in:        0.34997
# GM18G53250 POSSIBLE N-term signal sequence
GM18G53250      TMHMM2.0        outside      1    45
GM18G53250      TMHMM2.0        TMhelix     46    68
GM18G53250      TMHMM2.0        inside      69    80
GM18G53250      TMHMM2.0        TMhelix     81   100
GM18G53250      TMHMM2.0        outside    101   103
GM18G53250      TMHMM2.0        TMhelix    104   126
GM18G53250      TMHMM2.0        inside     127   137
GM18G53250      TMHMM2.0        TMhelix    138   160
GM18G53250      TMHMM2.0        outside    161   164
GM18G53250      TMHMM2.0        TMhelix    165   187
GM18G53250      TMHMM2.0        inside     188   198
GM18G53250      TMHMM2.0        TMhelix    199   221
GM18G53250      TMHMM2.0        outside    222   225
GM18G53250      TMHMM2.0        TMhelix    226   248
GM18G53250      TMHMM2.0        inside     249   297
```

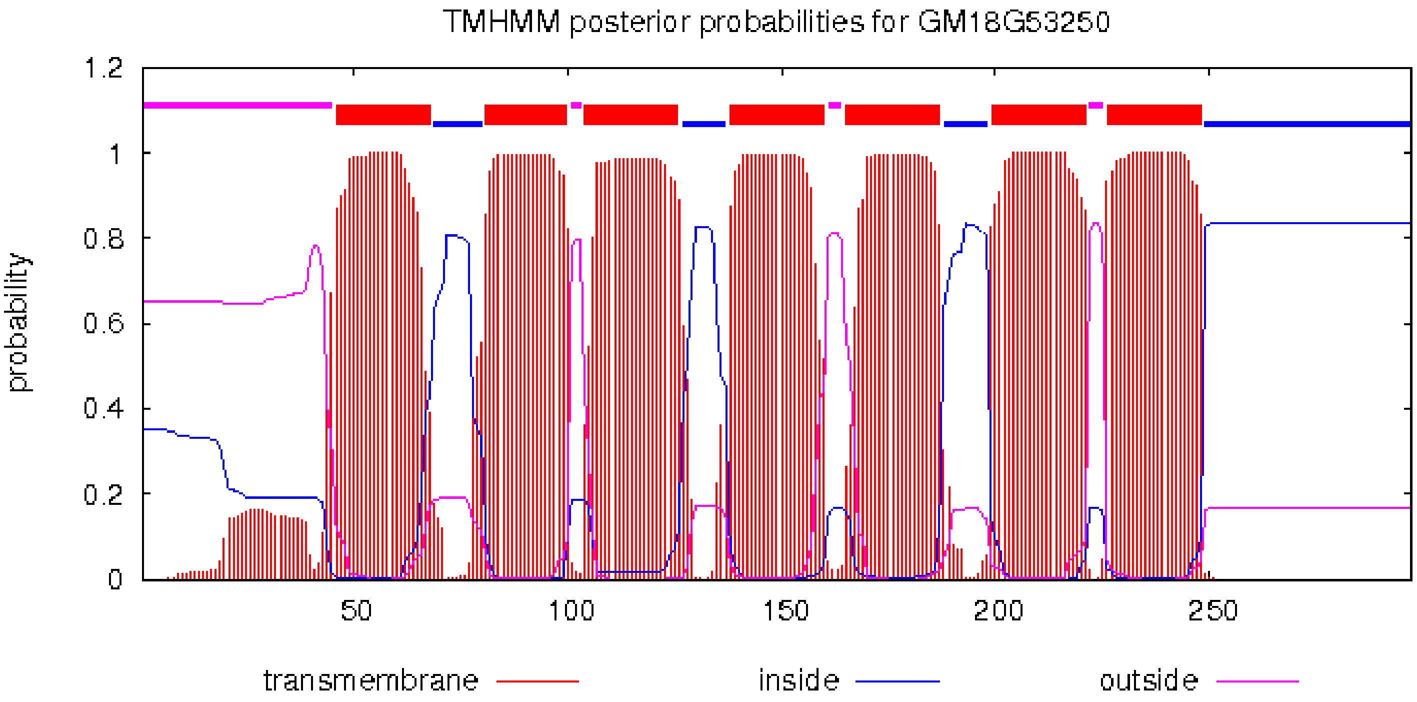

plot in postscript, script for making the plot in gnuplot, data for plot

---

```
# GM18G53930 Length: 268
# GM18G53930 Number of predicted TMHs:  7
# GM18G53930 Exp number of AAs in TMHs: 154.6457
# GM18G53930 Exp number, first 60 AAs:  39.42369
# GM18G53930 Total prob of N-in:        0.01374
# GM18G53930 POSSIBLE N-term signal sequence
GM18G53930      TMHMM2.0        outside      1     9
GM18G53930      TMHMM2.0        TMhelix     10    32
GM18G53930      TMHMM2.0        inside      33    44
GM18G53930      TMHMM2.0        TMhelix     45    64
GM18G53930      TMHMM2.0        outside     65    67
GM18G53930      TMHMM2.0        TMhelix     68    90
GM18G53930      TMHMM2.0        inside      91   101
GM18G53930      TMHMM2.0        TMhelix    102   124
GM18G53930      TMHMM2.0        outside    125   133
GM18G53930      TMHMM2.0        TMhelix    134   156
GM18G53930      TMHMM2.0        inside     157   162
GM18G53930      TMHMM2.0        TMhelix    163   185
GM18G53930      TMHMM2.0        outside    186   188
GM18G53930      TMHMM2.0        TMhelix    189   211
GM18G53930      TMHMM2.0        inside     212   268
```

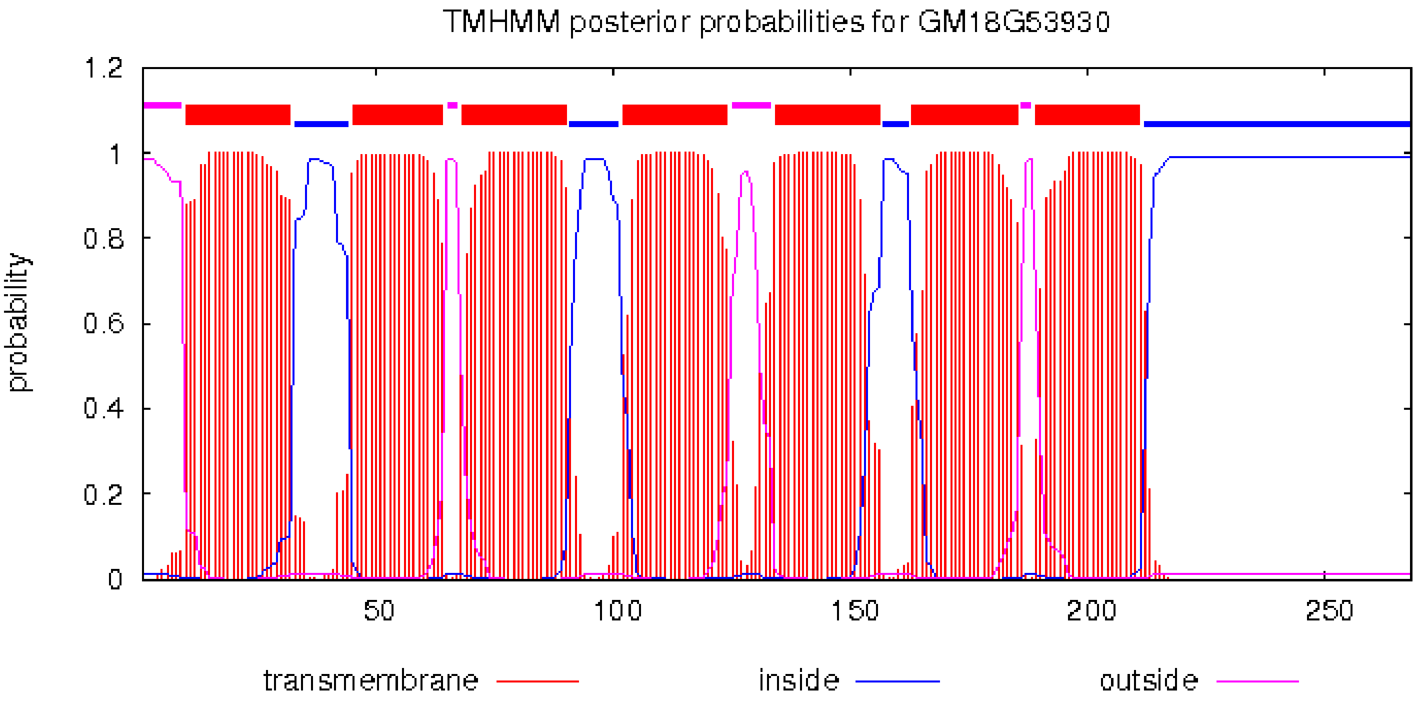

plot in postscript, script for making the plot in gnuplot, data for plot

---

```
# GM18G53940 Length: 271
# GM18G53940 Number of predicted TMHs:  7
# GM18G53940 Exp number of AAs in TMHs: 154.37561
# GM18G53940 Exp number, first 60 AAs:  39.03152
# GM18G53940 Total prob of N-in:        0.00322
# GM18G53940 POSSIBLE N-term signal sequence
GM18G53940      TMHMM2.0        outside      1     9
GM18G53940      TMHMM2.0        TMhelix     10    32
GM18G53940      TMHMM2.0        inside      33    44
GM18G53940      TMHMM2.0        TMhelix     45    64
GM18G53940      TMHMM2.0        outside     65    67
GM18G53940      TMHMM2.0        TMhelix     68    90
GM18G53940      TMHMM2.0        inside      91   101
GM18G53940      TMHMM2.0        TMhelix    102   124
GM18G53940      TMHMM2.0        outside    125   133
GM18G53940      TMHMM2.0        TMhelix    134   156
GM18G53940      TMHMM2.0        inside     157   162
GM18G53940      TMHMM2.0        TMhelix    163   185
GM18G53940      TMHMM2.0        outside    186   188
GM18G53940      TMHMM2.0        TMhelix    189   211
GM18G53940      TMHMM2.0        inside     212   271
```

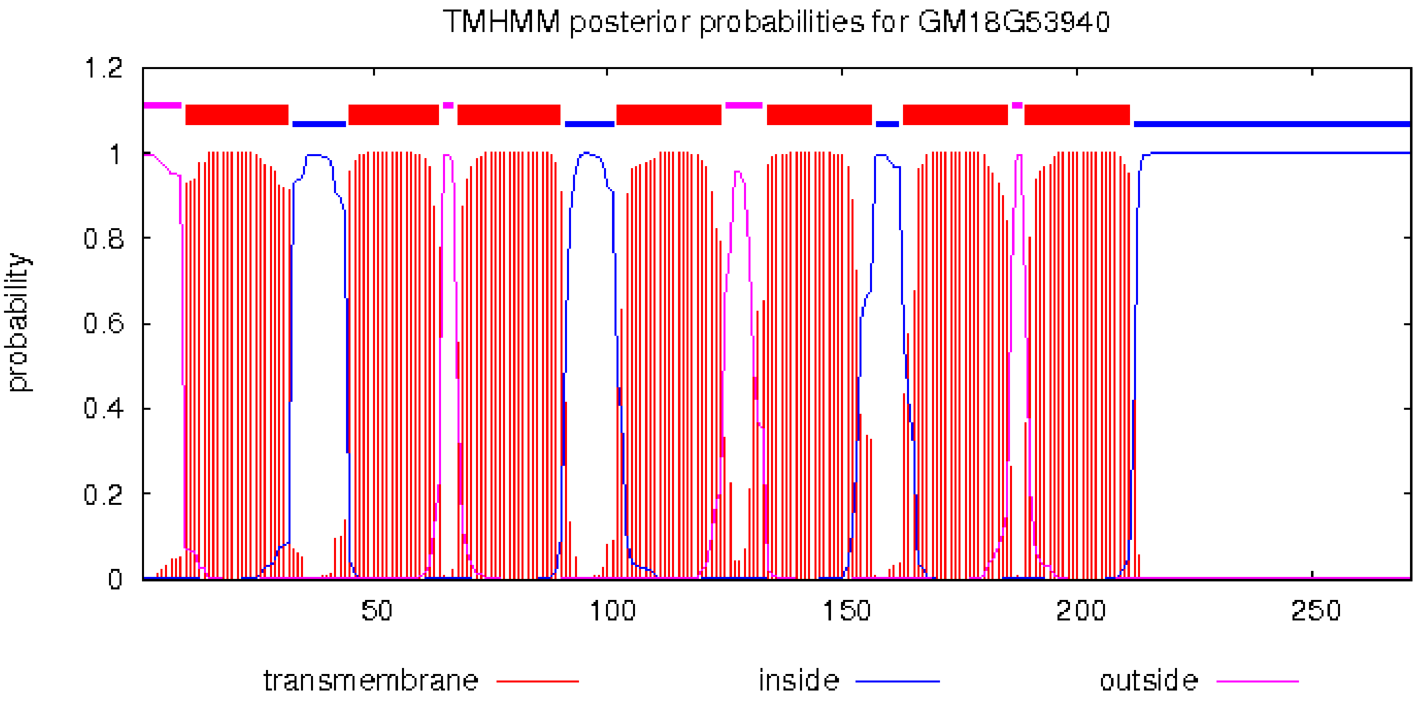

plot in postscript, script for making the plot in gnuplot, data for plot

---

```
# GM19G01270 Length: 246
# GM19G01270 Number of predicted TMHs:  7
# GM19G01270 Exp number of AAs in TMHs: 148.77888
# GM19G01270 Exp number, first 60 AAs:  33.65065
# GM19G01270 Total prob of N-in:        0.20573
# GM19G01270 POSSIBLE N-term signal sequence
GM19G01270      TMHMM2.0        outside      1     3
GM19G01270      TMHMM2.0        TMhelix      4    26
GM19G01270      TMHMM2.0        inside      27    46
GM19G01270      TMHMM2.0        TMhelix     47    66
GM19G01270      TMHMM2.0        outside     67    70
GM19G01270      TMHMM2.0        TMhelix     71    93
GM19G01270      TMHMM2.0        inside      94   101
GM19G01270      TMHMM2.0        TMhelix    102   124
GM19G01270      TMHMM2.0        outside    125   133
GM19G01270      TMHMM2.0        TMhelix    134   156
GM19G01270      TMHMM2.0        inside     157   162
GM19G01270      TMHMM2.0        TMhelix    163   185
GM19G01270      TMHMM2.0        outside    186   189
GM19G01270      TMHMM2.0        TMhelix    190   212
GM19G01270      TMHMM2.0        inside     213   246
```

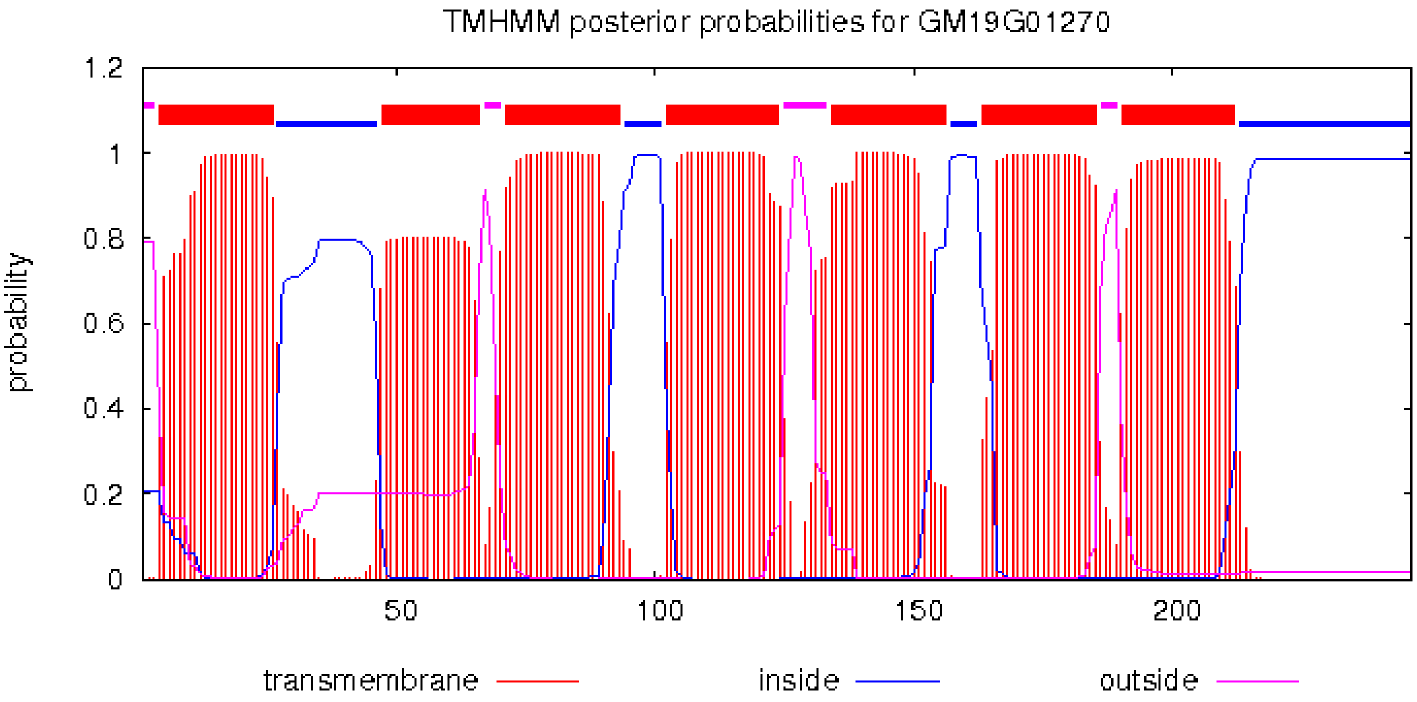

plot in postscript, script for making the plot in gnuplot, data for plot

---

```
# GM19G01280 Length: 246
# GM19G01280 Number of predicted TMHs:  6
# GM19G01280 Exp number of AAs in TMHs: 144.76818
# GM19G01280 Exp number, first 60 AAs:  31.39795
# GM19G01280 Total prob of N-in:        0.48361
# GM19G01280 POSSIBLE N-term signal sequence
GM19G01280      TMHMM2.0        inside       1    11
GM19G01280      TMHMM2.0        TMhelix     12    34
GM19G01280      TMHMM2.0        outside     35    68
GM19G01280      TMHMM2.0        TMhelix     69    91
GM19G01280      TMHMM2.0        inside      92   102
GM19G01280      TMHMM2.0        TMhelix    103   125
GM19G01280      TMHMM2.0        outside    126   134
GM19G01280      TMHMM2.0        TMhelix    135   157
GM19G01280      TMHMM2.0        inside     158   163
GM19G01280      TMHMM2.0        TMhelix    164   186
GM19G01280      TMHMM2.0        outside    187   190
GM19G01280      TMHMM2.0        TMhelix    191   213
GM19G01280      TMHMM2.0        inside     214   246
```

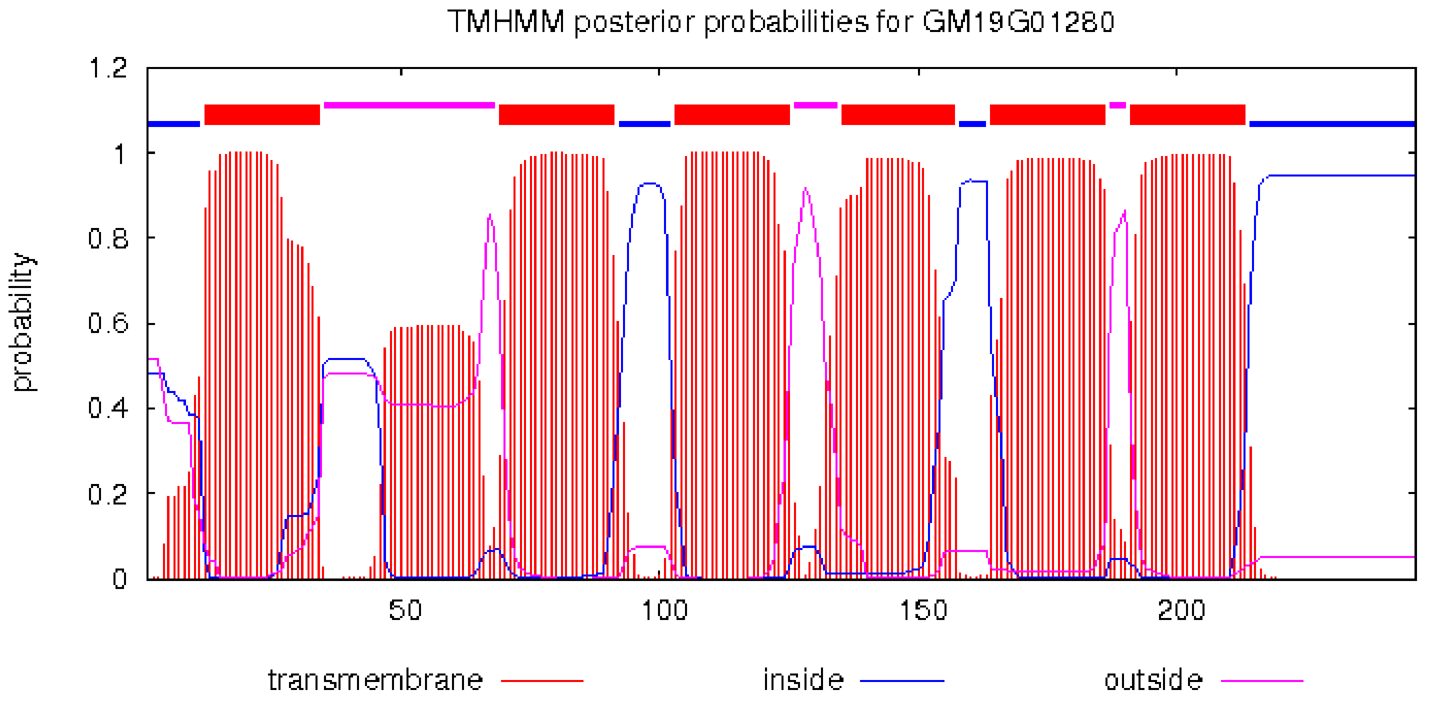

plot in postscript, script for making the plot in gnuplot, data for plot

---

```
# GM19G42040 Length: 307
# GM19G42040 Number of predicted TMHs:  7
# GM19G42040 Exp number of AAs in TMHs: 151.86805
# GM19G42040 Exp number, first 60 AAs:  34.77714
# GM19G42040 Total prob of N-in:        0.15649
# GM19G42040 POSSIBLE N-term signal sequence
GM19G42040      TMHMM2.0        outside      1     3
GM19G42040      TMHMM2.0        TMhelix      4    26
GM19G42040      TMHMM2.0        inside      27    45
GM19G42040      TMHMM2.0        TMhelix     46    65
GM19G42040      TMHMM2.0        outside     66    68
GM19G42040      TMHMM2.0        TMhelix     69    91
GM19G42040      TMHMM2.0        inside      92   102
GM19G42040      TMHMM2.0        TMhelix    103   125
GM19G42040      TMHMM2.0        outside    126   128
GM19G42040      TMHMM2.0        TMhelix    129   151
GM19G42040      TMHMM2.0        inside     152   163
GM19G42040      TMHMM2.0        TMhelix    164   186
GM19G42040      TMHMM2.0        outside    187   190
GM19G42040      TMHMM2.0        TMhelix    191   213
GM19G42040      TMHMM2.0        inside     214   307
```

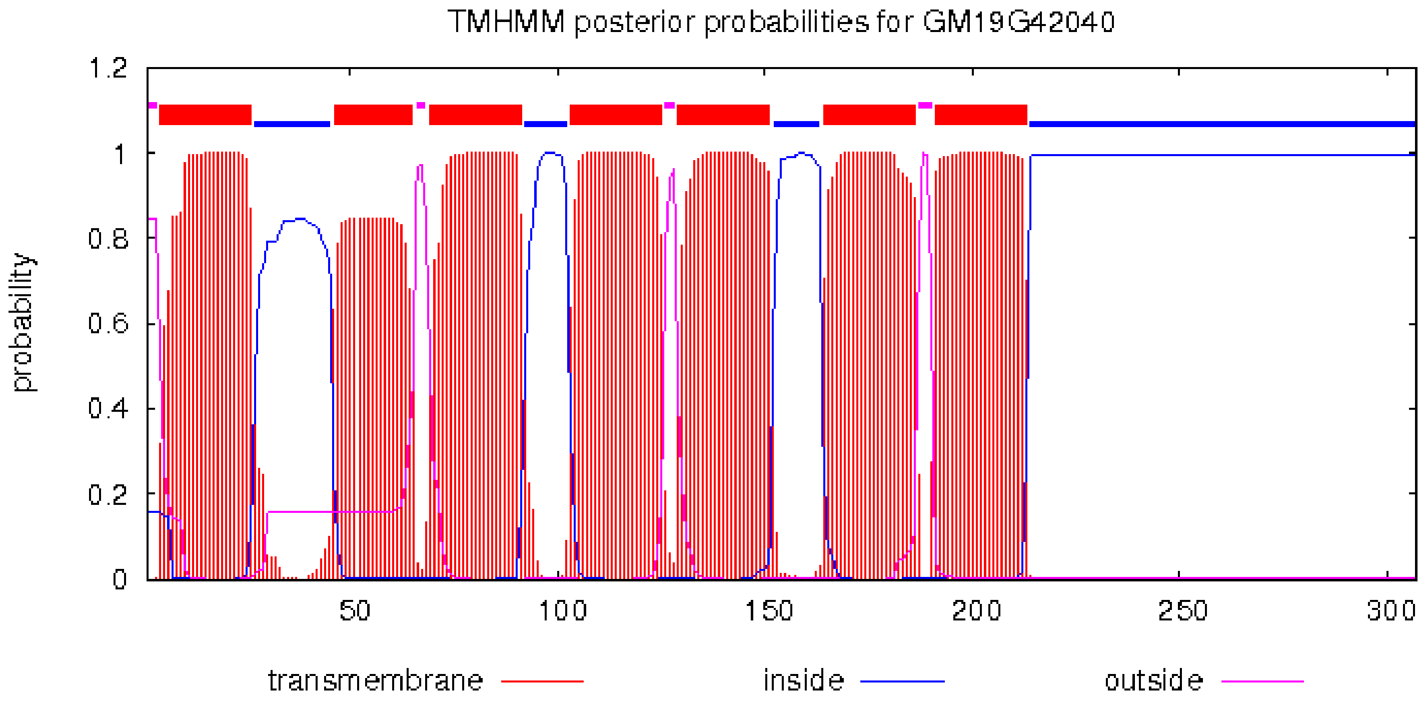

plot in postscript, script for making the plot in gnuplot, data for plot

---

```
# GM20G01895 Length: 192
# GM20G01895 Number of predicted TMHs:  3
# GM20G01895 Exp number of AAs in TMHs: 59.64746
# GM20G01895 Exp number, first 60 AAs:  18.01466
# GM20G01895 Total prob of N-in:        0.85934
# GM20G01895 POSSIBLE N-term signal sequence
GM20G01895      TMHMM2.0        inside       1    20
GM20G01895      TMHMM2.0        TMhelix     21    43
GM20G01895      TMHMM2.0        outside     44    97
GM20G01895      TMHMM2.0        TMhelix     98   117
GM20G01895      TMHMM2.0        inside     118   123
GM20G01895      TMHMM2.0        TMhelix    124   146
GM20G01895      TMHMM2.0        outside    147   192
```

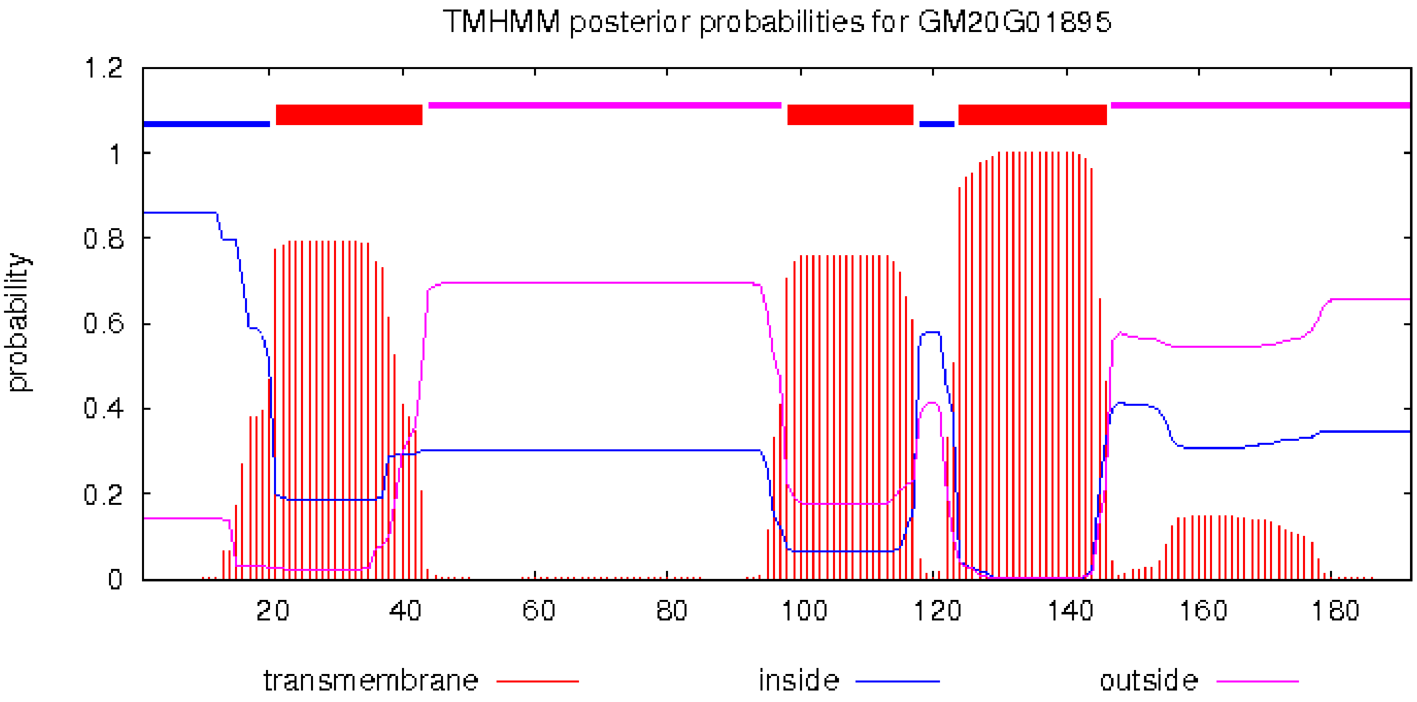

plot in postscript, script for making the plot in gnuplot, data for plot

---

```
# GM20G16160 Length: 256
# GM20G16160 Number of predicted TMHs:  6
# GM20G16160 Exp number of AAs in TMHs: 151.98856
# GM20G16160 Exp number, first 60 AAs:  37.56298
# GM20G16160 Total prob of N-in:        0.17768
# GM20G16160 POSSIBLE N-term signal sequence
GM20G16160      TMHMM2.0        inside       1    11
GM20G16160      TMHMM2.0        TMhelix     12    34
GM20G16160      TMHMM2.0        outside     35    43
GM20G16160      TMHMM2.0        TMhelix     44    63
GM20G16160      TMHMM2.0        inside      64    69
GM20G16160      TMHMM2.0        TMhelix     70    92
GM20G16160      TMHMM2.0        outside     93   101
GM20G16160      TMHMM2.0        TMhelix    102   124
GM20G16160      TMHMM2.0        inside     125   130
GM20G16160      TMHMM2.0        TMhelix    131   153
GM20G16160      TMHMM2.0        outside    154   191
GM20G16160      TMHMM2.0        TMhelix    192   214
GM20G16160      TMHMM2.0        inside     215   256
```

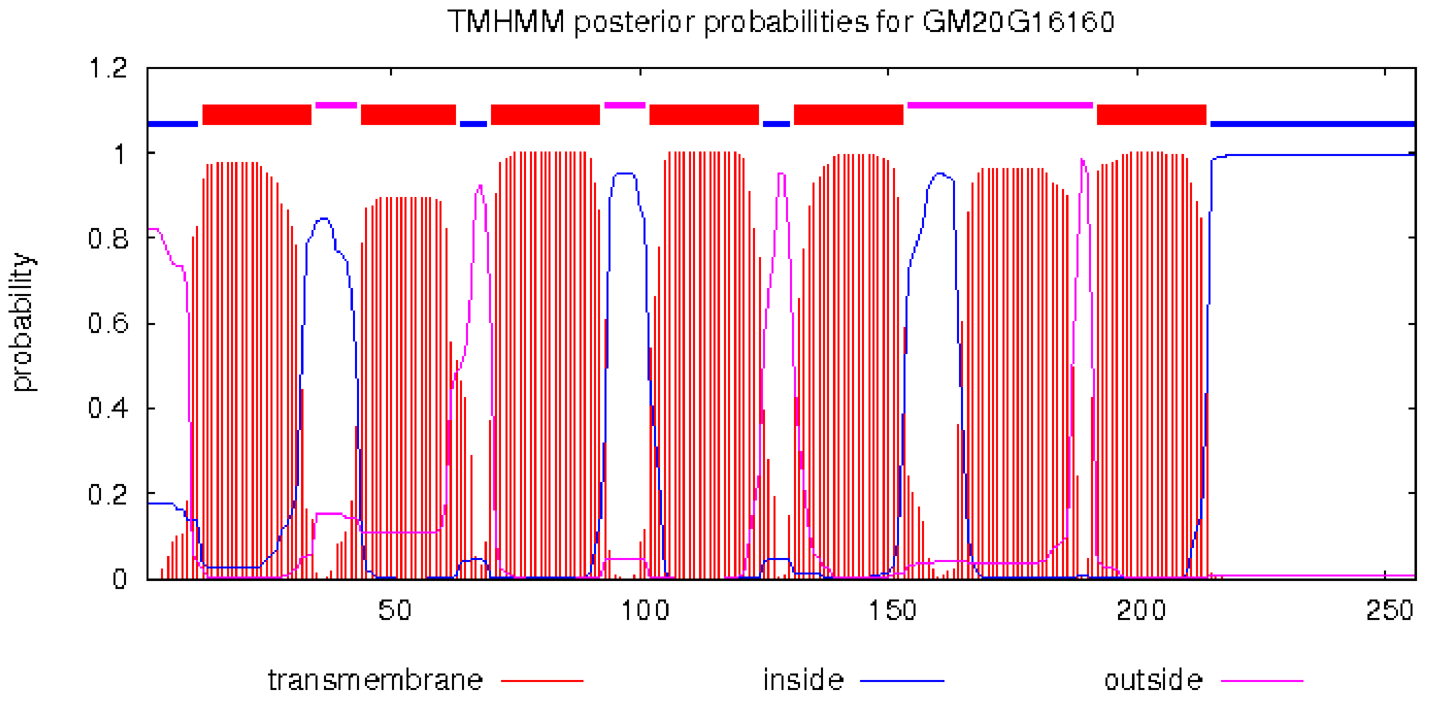

plot in postscript, script for making the plot in gnuplot, data for plot

---

```
# GM20G21064 Length: 179
# GM20G21064 Number of predicted TMHs:  3
# GM20G21064 Exp number of AAs in TMHs: 80.41464
# GM20G21064 Exp number, first 60 AAs:  13.8797
# GM20G21064 Total prob of N-in:        0.43149
# GM20G21064 POSSIBLE N-term signal sequence
GM20G21064      TMHMM2.0        outside      1    67
GM20G21064      TMHMM2.0        TMhelix     68    90
GM20G21064      TMHMM2.0        inside      91    98
GM20G21064      TMHMM2.0        TMhelix     99   121
GM20G21064      TMHMM2.0        outside    122   124
GM20G21064      TMHMM2.0        TMhelix    125   147
GM20G21064      TMHMM2.0        inside     148   179
```

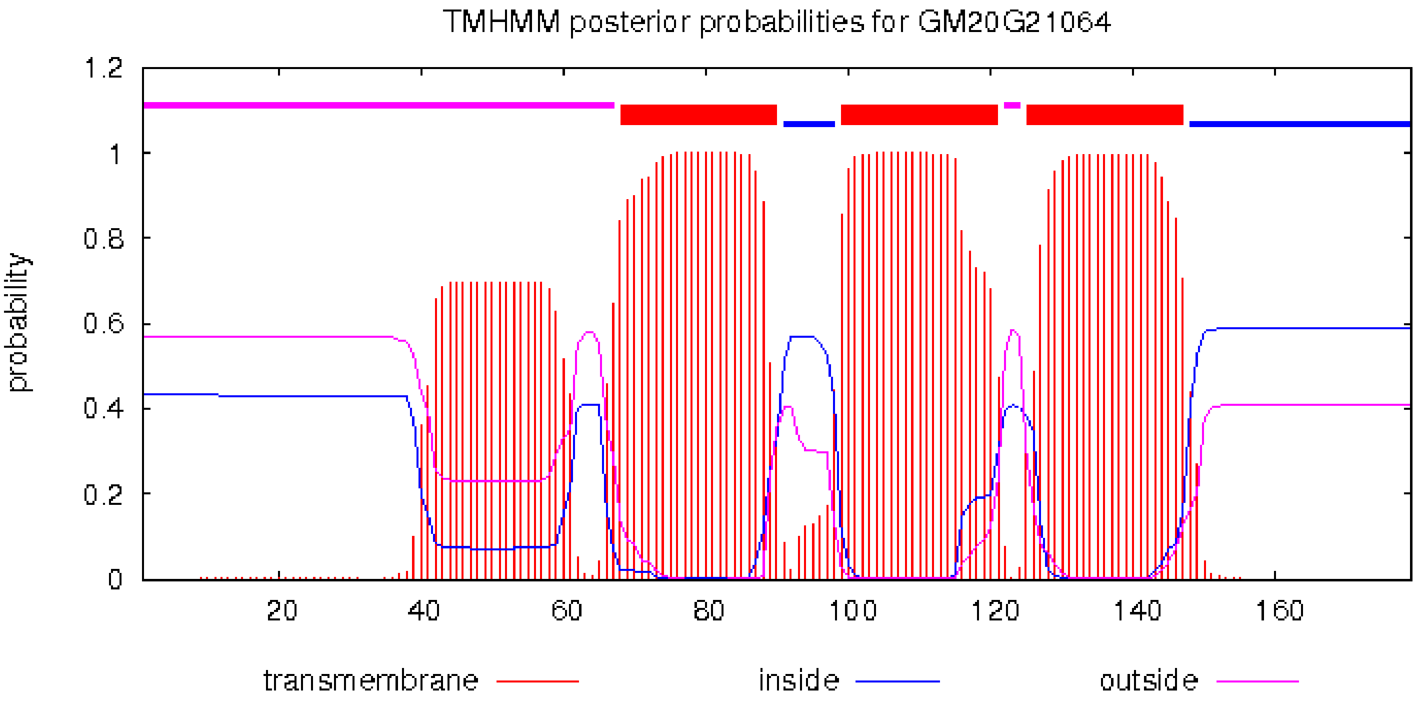

plot in postscript, script for making the plot in gnuplot, data for plot

---
