# Supplementary material for: Soybean (Glycine max) SWEET gene family: insights through comparative genomics, transcriptome profiling and whole genome re-sequence analysis
Source: BMC Genomics. 2015 Jul 11;16(1):520. doi: 10.1186/s12864-015-1730-y (PMC4499210; doi:10.1186/s12864-015-1730-y)
Supplement: Additional file 6: — cis -motif analysis of soybean SWEET genes. Conserved motifs identified in proximal promoter region of SWEET gene family using INCLUSive MotifSampler and its similarity with known motifs available in Athmap database. Similarity search performed using STAM tool (www.benoslab.pitt.edu/stamp). [file 12864_2015_1730_MOESM6_ESM.pdf]

Additional file 6 – *cis*-motif analysis of soybean SWEET genes. Conserved motifs identified in proximal promoter region of SWEET gene family using INCLUSive MotifSampler and its similarity with known motifs available in Athmap database. Similarity search performed using STAM tool (www.benoslab.pitt.edu/stamp).

Sr. No	Motif identified in SWEET Gene promoter	Significant match with Athmap motif	Athmap similarity	Annotation
1	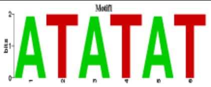	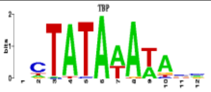	TBP<br>(E val: 9.2143e-05)	TATA box binding protein
2	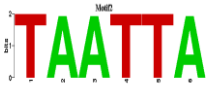	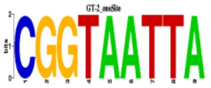	GT-2_oneSite<br>(E val: 1.1768e-08)	Grass Transcription factor 2
3	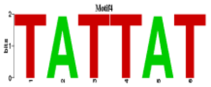	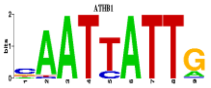	ATHB1<br>(E val: 4.8179e-05)	*Arabidopsis thaliana* homeobox 1
4	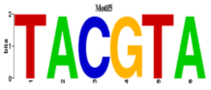	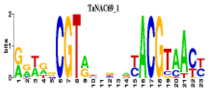	TaNAC69_1<br>(E val: 1.0461e-05)	*Triticum aestivum* NAC transcription factors
5	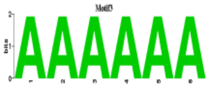	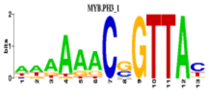	MYB.PH3_1<br>(E val: 1.6064e-06)	*Petunia hybrida* petal epidermis-specific MYB transcription factor (MYB.Ph3)
6	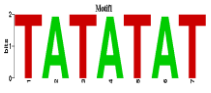	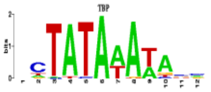	TBP<br>(E val: 2.2256e-06)	TATA box binding protein
7	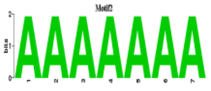	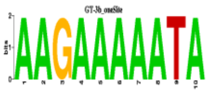	GT-3b_oneSite<br>(E val: 1.2349e-05)	Trihelix transcription factor GT-3b
8	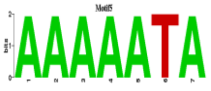	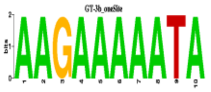	GT-3b_oneSite<br>(E val: 2.2828e-10)	Trihelix transcription factor GT-3b
9	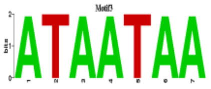	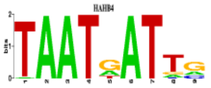	HAHB4<br>(E val: 2.3891e-04)	*Helianthus annuus* homeobox-4
10	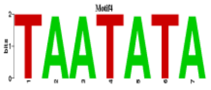	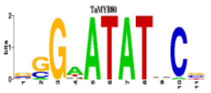	TaMYB80<br>(E val: 1.1679e-03)	*Triticum aestivum* MYB80
11	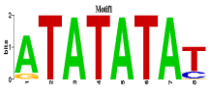	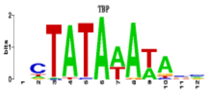	TBP<br>(E val: 2.9772e-05)	TATA box binding protein
12	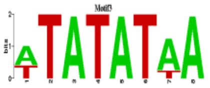	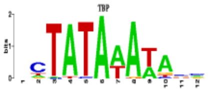	TBP<br>(E val: 9.4475e-06)	TATA box binding protein
13	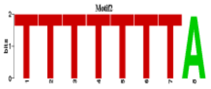	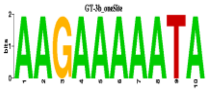	GT-3b_oneSite<br>(E val: 9.3255e-05)	Trihelix transcription factor GT-3b
14	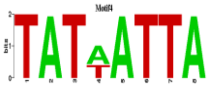	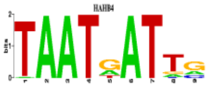	HAHB4<br>(E val: 1.7135e-05)	*Helianthus annuus* homeobox-4
15	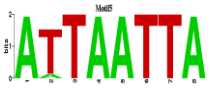	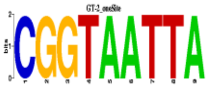	GT-2_oneSite<br>(E val: 4.8477e-06)	Grass Transcription factor 2
