# Supplementary material for: Soybean (Glycine max) SWEET gene family: insights through comparative genomics, transcriptome profiling and whole genome re-sequence analysis
Source: BMC Genomics. 2015 Jul 11;16(1):520. doi: 10.1186/s12864-015-1730-y (PMC4499210; doi:10.1186/s12864-015-1730-y)
Supplement: Additional file 10: — SNP-Haplotype analysis of SWEET gene cluster on chromosome 6. Hierarchical clustering showed the association between average sucrose content and SNPs haplogroups in 106 lines. Base position identical to reference (Williams 82) are light sky blue, black – different, gray-missing data. Blue colored bar on top showing approximate position of GmSWEET15, −16 and −17. [file 12864_2015_1730_MOESM10_ESM.pptx]

## Slide 1
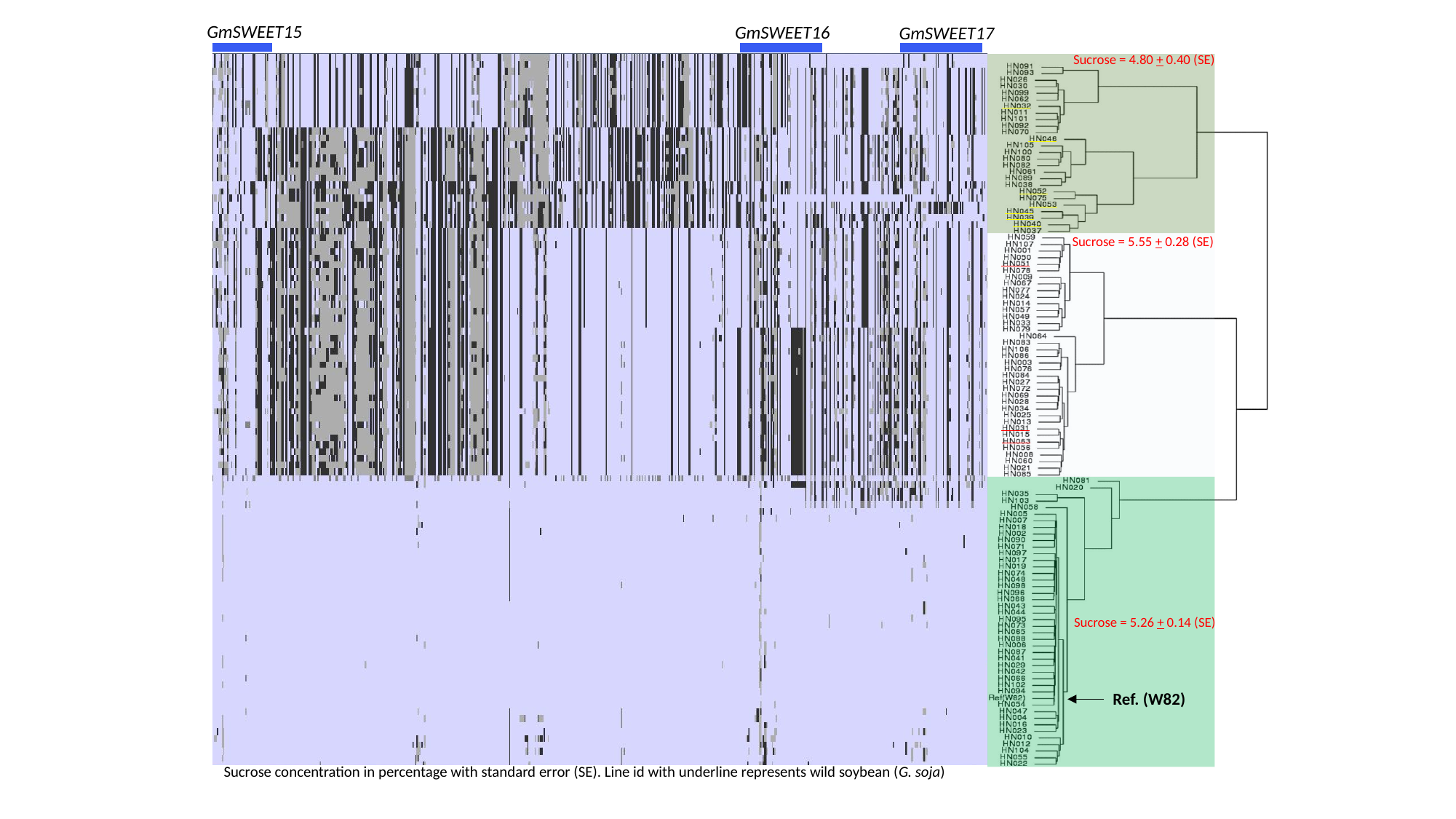

GmSWEET15
GmSWEET16
GmSWEET17
Sucrose = 4.80 + 0.40 (SE)
Sucrose = 5.55 + 0.28 (SE)
Sucrose = 5.26 + 0.14 (SE)
Ref. (W82)
Sucrose concentration in percentage with standard error (SE). Line id with underline represents wild soybean (G. soja)
