# Supplementary material for: Soybean (Glycine max) SWEET gene family: insights through comparative genomics, transcriptome profiling and whole genome re-sequence analysis
Source: BMC Genomics. 2015 Jul 11;16(1):520. doi: 10.1186/s12864-015-1730-y (PMC4499210; doi:10.1186/s12864-015-1730-y)
Supplement: Additional file 11: — Primer sequences of 23 selected GmSWEET genes for RT-qPCR analysis. [file 12864_2015_1730_MOESM11_ESM.pdf]

Additional file 11: Primer sequences of 23 selected *Soybean* SWEET genes for RT-qPCR analysis

Unique ID	GmSWEET ID	Primer Seq (5' - 3')
Glyma02g09710.1	*GmSWEET1_F*	TGGTGTTCTTAGCACCCTTGCC
	GmSWEET1_R	TGCGCTAAGAAGTGCAACCG
Glyma04g37510.1	*GmSWEET4_F*	TTTGGCATAATCCAGATGGTGCTG
	GmSWEET4_R	GCTCCTCCAATGTTTGTGGCTTG
Glyma04g37520.1	*GmSWEET5_F*	ACGCCAAGAAAGATGAGCCAATG
	GmSWEET5_R	TGTTGCATGATTCGGCTCAGAGG
Glyma04g42040.1	*GmSWEET8_F*	AGGGATACCTTCTCCATCCGCTAC
	GmSWEET8_R	GCGGAAGCATTCCCAAAGATGC
Glyma05g25180.1	*GmSWEET10_F*	TCACTCAAGCCAGCTGATGCTAC
	GmSWEET10_R	ACGGTTAAGTTCCGGGCATCTTTG
Glyma05g38340.1	*GmSWEET12_F*	GTGAAGGCTCAAGAATTGAATGGC
	GmSWEET12_R	AGTAGCTGCGTGATTCGGTTCC
Glyma06g12740.1	*GmSWEET13_F*	GAATGCTTCCGCTCTGTTCCTC
	GmSWEET13_R	GGGAAGGAAGCAGCTATGTAATGC
Glyma06g13110.1	*GmSWEET14_F*	GGTACGGTTTGCCTGTTGTGAG
	GmSWEET14_R	ACCAACTCCATTAACTGTGACGAG
Glyma06g17520.1	*GmSWEET15_F*	TGCCCGAAGAAAGCTAGGATGTC
	GmSWEET15_R	AGCTTGACACGGGCTTCTTCTC
Glyma06g17530.1	*GmSWEET16_F*	AACGCCAAGACACCTGATCTGC
	GmSWEET16_R	TTCTGCTCAGCTTCCCAACGTC
Glyma06g17540.1	*GmSWEET17_F*	GGCCTCATTCAACGCAAGAACG
	GmSWEET17_R	AGCTACCCGTTCAGGCAAATACG
Glyma08g01310.1	*GmSWEET21_F*	CTACCTGTCAAAGGGAGCAAAGCG
	GmSWEET21_R	ACGCTCCTCGTCTTTATGACTCG
Glyma08g02890.1	*GmSWEET22_F*	AGAAACGCTCTGCCATTGTTGG
	GmSWEET22_R	TTCATGATAGCCAAGGGTGAAGC
Glyma08g08200.1	*GmSWEET23_F*	CTTTGCTATACATGGCTCCCTACG
	GmSWEET23_R	TCGAATAACCTGAGCCACAATGC
Glyma08g47560.1	*GmSWEET26_F*	TGCCTTACCTGGTGGCATTGTTC
	GmSWEET26_R	GAGAAGCATGGCGTCTTTCTTGAG
Glyma09g04840.1	*GmSWEET28_F*	GCGATCTACAGAAATGGGAAGCG
	GmSWEET28_R	TGGGCCTGTCTTCACTCTGATG
Glyma12g36300.1	*GmSWEET29_F*	GCTGAAGAATCAGAGTCAGGAACG
	GmSWEET29_R	AACTCCAGTCTCAGCCAGCAAC
Glyma13g33950.1	*GmSWEET34_F*	TCAAGAAAGGCACATCGCACCTC
	GmSWEET34_R	AAGAGAAGCCAGCCTAGAGCAC
Glyma14g30940.1	*GmSWEET38_F*	TTTCGTTGCGGGACCAAGTCTG
	GmSWEET38_R	TTGCAGTGGAGAACGAGTTGGAG
Glyma15g16030.1	*GmSWEET40_F*	ACATGAGCCTCTCATCTCACAGC
	GmSWEET40_R	AACTAGATGGGCCTGTCTTCCC
Glyma18g53930.1	*GmSWEET45_F*	TGTGGTTGCCCAAAGTCAGAAG
	GmSWEET45_R	TGCCGAAGGCAAATGCCAAAG
Glyma18g53940.1	*GmSWEET46_F*	TCATTCTTGTGGTTGCCAAAGTCG

	GmSWEET46_R	TGGCACTGGAGCCAAGAATACC
Glyma19g01280.1	*GmSWEET48_F*	AACCAAGTGGCTTTCAGCTCTC
	GmSWEET48_R	TGTGACATCAGACTACTGCAGCTC
